# A phase I/Ib trial and biological correlate analysis of neoadjuvant SBRT with single-dose durvalumab in HPV-unrelated locally advanced HNSCC

**DOI:** 10.1038/s43018-022-00450-6

**Published:** 2022-11-25

**Authors:** Laurel B. Darragh, Michael M. Knitz, Junxiao Hu, Eric T. Clambey, Jennifer Backus, Andrew Dumit, Von Samedi, Andrew Bubak, Casey Greene, Timothy Waxweiler, Sanjana Mehrotra, Shilpa Bhatia, Jacob Gadwa, Thomas Bickett, Miles Piper, Kareem Fakhoury, Arthur Liu, Joshua Petit, Daniel Bowles, Ashesh Thaker, Kimberly Atiyeh, Julie Goddard, Robert Hoyer, Adrie Van Bokhoven, Kimberly Jordan, Antonio Jimeno, Angelo D’Alessandro, David Raben, Jessica D. McDermott, Sana D. Karam

**Affiliations:** 1 Radiation Oncology, University of Colorado Denver at Anschutz Medical Campus, Aurora, CO USA; 2 Department of Immunology, University of Colorado Denver at Anschutz Medical Campus, Aurora, CO USA; 3 Department of Biostatistics and Informatics, Colorado School of Public Health, University of Colorado Denver, Aurora, CO USA; 4 Department of Anesthesiology, University of Colorado Anschutz Medical Campus, Aurora, CO USA; 5 Department of Pathology, University of Colorado Denver at Anschutz Medical Campus, Aurora, CO USA; 6 Department of Neurology, University of Colorado Denver at Anschutz Medical Campus, Aurora, CO USA; 7 Department of Biochemistry and Molecular Genetics, University of Colorado Denver at Anschutz Medical Campus, Aurora, CO USA; 8 Department of Radiation Oncology, University of Colorado, Poudre Valley Hospital, Fort Collins, CO USA; 9 Division of Medical Oncology, University of Colorado School of Medicine, Aurora, CO USA; 10 Department of Radiology, University of Colorado Denver at Anschutz Medical Campus, Aurora, CO USA; 11 Department of Otolaryngology Head and Neck Surgery, University of Colorado, Memorial South Hospital, Colorado Springs, CO USA; 12 Department of Otolaryngology Head and Neck Surgery, University of Colorado Denver at Anschutz Medical Campus, Aurora, CO USA

**Keywords:** Oral cancer, Tumour immunology, Cancer, Immunotherapy

## Abstract

Five-year survival for human papilloma virus-unrelated head and neck squamous cell carcinomas remain below 50%. We assessed the safety of administering combination hypofractionated stereotactic body radiation therapy with single-dose durvalumab (anti-PD-L1) neoadjuvantly (*n* = 21) (NCT03635164). The primary endpoint of the study was safety, which was met. Secondary endpoints included radiographic, pathologic and objective response; locoregional control; progression-free survival; and overall survival. Among evaluable patients at an early median follow-up of 16 months (448 d or 64 weeks), overall survival was 80.1% with 95% confidence interval (95% CI) (62.0%, 100.0%), locoregional control and progression-free survival were 75.8% with 95% CI (57.5%, 99.8%), and major pathological response or complete response was 75% with 95% exact CI (51.6%, 100.0%). For patients treated with 24 Gy, 89% with 95% CI (57.1%, 100.0%) had MPR or CR. Using high-dimensional multi-omics and spatial data as well as biological correlatives, we show that responders had: (1) an increase in effector T cells; (2) a decrease in immunosuppressive cells; and (3) an increase in antigen presentation post-treatment.

## Abstract (web summary)

Karam and colleagues report a phase I/Ib trial in patients with HNSCC treated with neoadjuvant SBRT and anti-PD-L1, and perform high-dimensional analyses of immune correlates of response in the tissue microenvironment and peripheral blood.

## Main

Patients with human papilloma virus (HPV)-unrelated head and neck squamous cell carcinoma (HNSCC) are at high risk for poor survival outcomes and high morbidity^1^. Locally advanced HPV-unrelated HNSCC of the oral cavity or larynx is still primarily treated with a combination of surgery, radiation and chemotherapy^2^. Lacking effective therapeutics targeting oncogenic drivers^3^, the advent of immunotherapy has provided a new modality for treating these historically unresponsive tumors. Incorporating anti-PD-1 or anti-PD-L1, immune checkpoint inhibitors, into therapy has the potential to improve survival outcomes and decrease the treatment’s overall morbidity. Despite these advances, response rates of HPV-unrelated HNSCC to immune checkpoint blockade have been low, both pre-clinically and in recent clinical trials^4–8^.

Preclinical and clinical trials have shown that patients with increased T cell infiltration at the time of treatment for HPV-unrelated HNSCC have improved outcomes in response to immune checkpoint therapy^9,10^. Combining immunotherapies with radiation therapy to increase immune cell infiltration into the tumor microenvironment (TME) may improve response rates^11–13^. Hypofractionated stereotactic body radiation therapy (SBRT) may improve anti-tumor immune function instead of blunting it^13^. Dose-escalation studies have shown that hypofractionation is optimal for stimulating an anti-tumor immune response while minimizing an immune wound-healing phenotype^14–17^. Preclinical studies investigating how to overcome resistance to immunotherapy in HPV-unrelated HNSCC tumor models have shown that combining immunotherapy with SBRT can invigorate the immune system and drive tumor eradication^11,13^.

Based on these previous findings, we hypothesized that the addition of SBRT to treatment comprising neoadjuvant checkpoint inhibition would be safe and would prime the immune system, thereby improving the chances of successful surgeries and disease outcomes for patients with HPV-unrelated HNSCC. We tested this hypothesis in a phase I/Ib clinical trial combining neoadjuvant SBRT with anti-PD-L1.

## Results

### Patient population and trial design

Twenty-one patients with locally advanced HPV-unrelated oral cavity or larynx HNSCC participated in a phase I/Ib dose-escalation study (NCT03635164). General patient characteristics are summarized in Table 1. There were 14 men (67%) and 7 women (33%); median age was 61 yr (43–84, median interquartile range (IQR) 55–69); the majority were heavy smokers (*n* = 14; 66.7%). The most common subsite was oral cavity cancer (*n* = 18, 85.7%), and most patients had ≥T3 disease (*n* = 19, 90.5%). Most patients (*n* = 14; 67%) had node-positive disease. Five patients (24%) had received previous irradiation. This study’s primary objective was to determine the maximum tolerated dose (MTD) of neoadjuvant SBRT in combination with concurrent and postoperative durvalumab.

**Table 1:** Patient demographics

All patients		*N* (%)
Sex	Female	7 (33.3)
	Male	14 (66.7)
Age (yr)	Median (IQR)	61.0 (55.0 to 69.0)
Smoking	Never smoker	7 (33.3)
	Smoker	14 (66.7)
Alcohol history	≤20 yr	4 (19.0)
	>20 yr	8 (38.1)
	No alcohol	5 (23.8)
	(Missing)	4 (19.0)
Time to surgery (d)	Median (IQR)	46.0 (39.0 to 55.0)
Radiation dosage	12	3 (14.3)
	18	9 (42.9)
	24	9 (42.9)
T stage	T2	2 (9.5)
	T3	4 (19.0)
	T4	15 (71.4)
N stage	N0	7 (33.3)
	N1	5 (23.8)
	N2	9 (42.9)
Location	Gingiva	3 (14.3)
	Hard palate	1 (4.8)
	Larynx	3 (14.3)
	Retromolar Trigone	3 (14.3)
	Tongue	11 (52.4)
Previous RT (yes/no)	No	16 (76.2)
	Yes	5 (23.8)

**Fig. 1:**
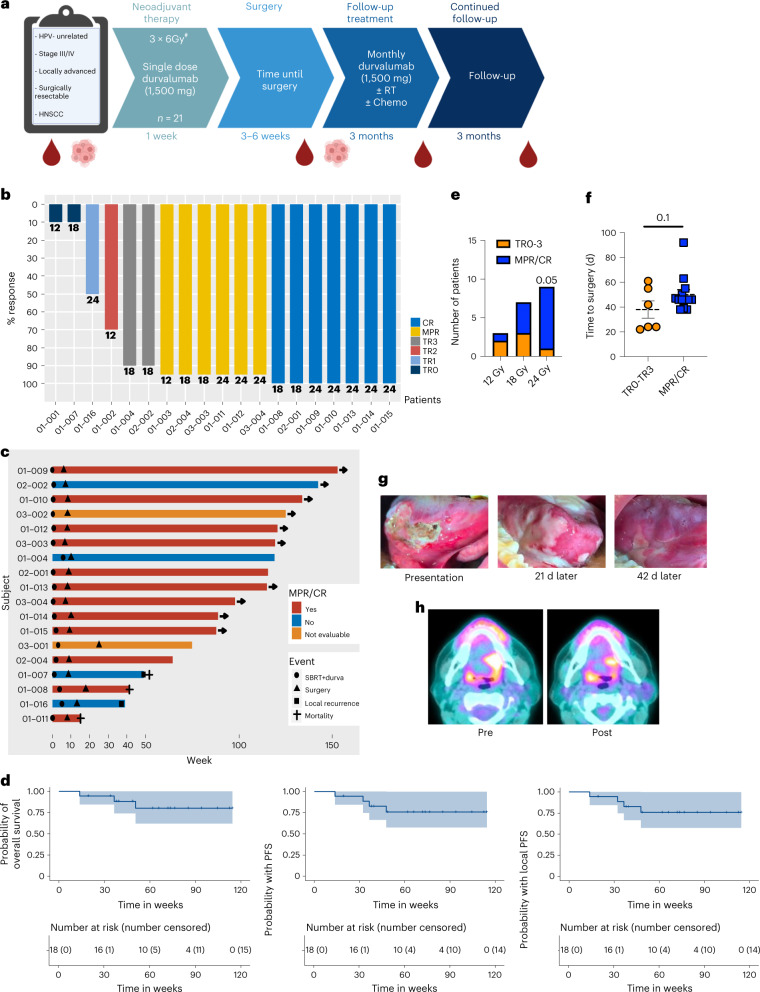
Summary of clinical outcomes. **a**, Diagram of trial design. **b**, Summary of pathological outcomes of all evaluable patients (*n* = 19 patients). **c**, Summary of clinical outcomes of patients treated at MTD (*n* = 18). **d**, Kaplan–Meier survival curves for overall survival, PFS and local PFS survival for patients treated at MTD (*n* = 18 patients). **e**, A plot of the relationship between radiation dose and pathologic outcome at time of surgery (*P* = 0.05), spearman correlation coefficient 0.45, CI (−0.01909, 0.7573). 12 Gy *n* = 3 patients, 18 Gy *n* = 7 patients and 24 Gy *n* = 9 patients. **f**, Analysis of the relationship between time to surgery and pathological response (mean ± s.e.m.). Pathologic tumor response (pTR) pTR0–3 *n* = 6 patients and MPR/CR *n* = 13 patients. **g**, Representative images of a patient’s tumor response to treatment. **h**, Representative standardized uptake value (SUV) images showing a decrease in signal intensity post-treatment. A two-sided Fisher’s exact test was used in **e**. Statistical significance was determined by an unpaired two-sided Student’s *t*-test for **f**. Significance was concluded if *P* < 0.05. ^#^For the last 9 patients the GTV was dosimetrically calculated at 24 Gy. Source data

**Extended Data Fig. 1:**
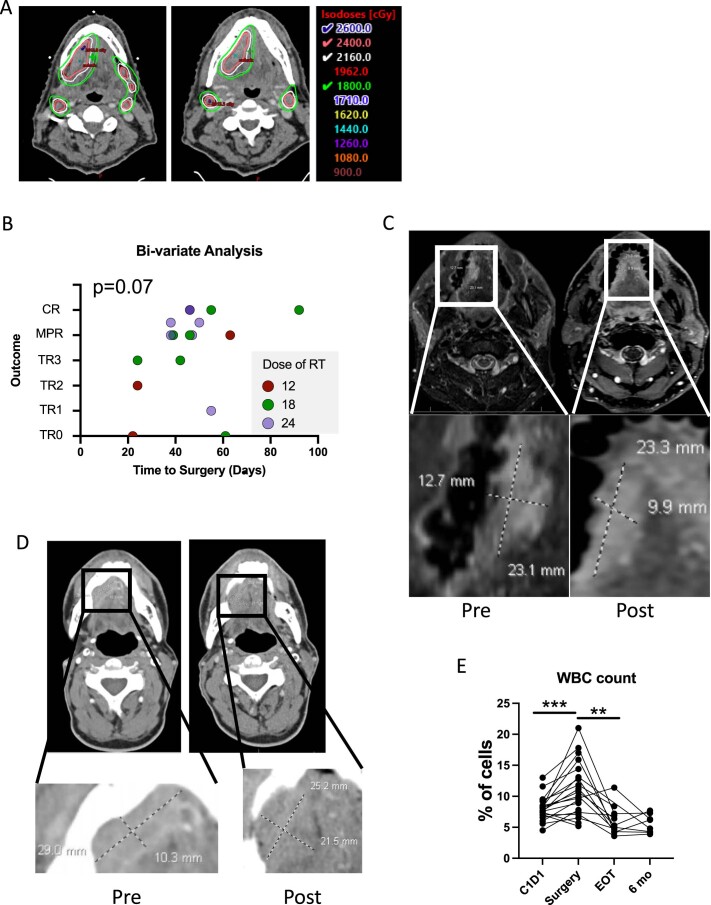
Representative images of SBRT planning and response to treatment. (**A**) Representative image of volumetric contouring of gross tumor only to determine the specific doses of SBRT delivered. (**B**) A multiple logistic regression analysis was used to determine if time to surgery or dose of radiation can independently account for pathological response at time of surgery (n = 19 patients). *P* = 0.07 (coefficient estimate per Gy of RT = 0.2253, s.e.m. = 0.1429, CI (−0.03648,0.5582); intercept for the logistic regression was −6.207). (**C**) Representative MRI of a patient pre- and post-treatment with tumor volume measurements magnified. (**D**) Representative CT image of a patient pre- and post-treatment with tumor volume measurements magnified. (**E**) White blood cell (WBC) count for each patient at four time points (C1D1, surgery, end of treatment (EOT) and 6-month follow-up). C1D1 n = 21 patients, surgery n = 20 patients, EOT n = 12 patients and 6-month follow-up n = 8 patients (*P* = 0.0006*** and *P* = 0.0019**). A multiple linear regression test was used to determine interdependence of time to surgery and dose of radiation for **B**. Statistical significance was determined by a paired two-sided Student’s t-test for **E**. Significance was concluded if *P*<0.05 (*P<0.05, **P<0.01, ***P<0.001). **These are representative images of 21 patients**.

Following enrollment, patients received one dose of neoadjuvant durvalumab (1,500 mg) approximately 3–6 weeks before standard-of-care surgery, given concurrently with neoadjuvant SBRT. This SBRT dose adjustment was escalated using a 3 + 3 model. The starting SBRT dose level was given as 6 Gy for two fractions (12 Gy total) every other day over approximately 1 week to sites of gross disease only to minimize exposure to normal tissue. Representative contouring images are depicted in Extended Data Fig. 1a. As no toxicity developed that delayed surgery by more than 8 weeks, the SBRT dose was increased to 6 Gy for three fractions (18 Gy total). With dosimetric dose painting, the gross tumor volume (GTV) dose for the last nine patients on trial was calculated at 24 Gy and will be reported as such. Patients underwent surgical resection approximately 3–6 weeks after radiation concluded to the initial planned resection margins, which were not adjusted in the case of a clinical response. For the first eight patients, adjuvant therapy (radiation or cisplatin–radiation therapy) was used post-surgery as per standard of care based on pathologic analysis and initial staging. All patients were required to receive adjuvant durvalumab, initiated at approximately 6–12 weeks after surgery, at 1,500 mg intravenously once every 4 weeks for a maximum of six doses, or until progression, toxicity or withdrawal from study. Durvalumab was given either as monotherapy or concurrently with adjuvant radiation with or without systemic therapy for high-risk patients. Following the eighth patient, the protocol was updated after discussion at the multi-disciplinary tumor board to allow omission of adjuvant radiation or chemoradiation for any patient who had a complete response (CR) or major pathological response (MPR) with no positive lymph nodes detected at time of surgery or on preoperative (post durvalumab–SBRT) imaging. All patients, however, were still required to receive adjuvant durvalumab. The trial design and each patient’s pathological outcome are summarized in Fig. 1a,b. Each patient’s treatment course is summarized in Fig. 1c.

### Summary of clinical outcomes

Among patients treated at MTD with 18 or 24 Gy (*n* = 18), at early median follow-up of 16 months (448 days), overall survival was 80.1% with 95% CI (62.0%, 100%), progression-free survival (PFS) and locoregional control were 75% with 95% CI (57%, 99.8%) as there were no distant recurrences, and MPR or CR was 75% with 95% CI (51%, 100%), and median survival was not reached for either endpoint (Fig. 1d). Demographic and clinical characteristics of patients who received ≥18 Gy are summarized in Table 2 and stratified by level of response. Of the 16 evaluable patients treated at MTD, 75% (*n* = 12) had MPR, including 7 patients (44%) with CR. For patients treated with 24 Gy, 89% with 95% CI (57.1%, 100%) had MPR or CR. All but one evaluable patient who received a dosimetric dose of 24 Gy (*n* = 9) covering the neoadjuvant GTV achieved MPR or CR. Radiation dose and time to surgery correlated with pathologic response to therapy (Fig. 1e,f). A visual representation of tumor response with this treatment regimen is provided in Fig. 1g. Using a multivariable logistic regression analysis to account for time to surgery, a dose of 24 Gy was positively associated with a better response, *P* = 0.07 (Extended Data Fig. 1b). One patient, treated below the MTD, recurred out of field, despite having received adjuvant radiation and durvalumab. Two other patients failed in field, one who refused adjuvant radiation but received adjuvant durvalumab (patient 01-007), and another who received adjuvant chemoradiation and durvalumab (patient 01-016). Two other patients died from unrelated causes. None of the patients who recurred had an MPR or CR. Decreased SUV or enhancement on magnetic resonance imaging (MRI) or computed tomography (CT) scan appeared to correlate with response (Fig. 1h and Extended Data Fig. 1c,d). Unlike traditional curative concurrent radioimmunotherapy, which induces lymphopenia^18^, SBRT combined with durvalumab increased white blood cell count from the first day of treatment (cycle 1, day 1 or C1D1) to time of surgery (Extended Data Fig. 1e).

**Table 2:** Demographic and clinical characteristics stratified by MPR

MPR (Yes/No)		No	Yes	*P*
Sex	Female	0 (0.0)	6 (50.0)	0.234
	Male	4 (100.0)	6 (50.0)	
Age (yr)	Median (IQR)	58.0 (53.8 to 66.8)	62.5 (57.2 to 69.0)	0.952
Smoking	Never smoker	1 (25.0)	3 (25.0)	1.000
	Smoker	3 (75.0)	9 (75.0)	
Time to surgery (d)	Median (IQR)	48.5 (37.5 to 56.5)	46.0 (44.2 to 47.8)	0.951
Radiation dosage	18	3 (75.0)	4 (33.3)	0.262
	24	1 (25.0)	8 (66.7)	
T stage	T2	0 (0.0)	0 (0.0)	0.529
	T3	0 (0.0)	3 (25.0)	
	T4	4 (100.0)	9 (75.0)	
N stage	N0	0 (0.0)	3 (25.0)	0.769
	N1	1 (25.0)	4 (33.3)	
	N2	3 (75.0)	5 (41.7)	
Alcohol history	≤20 yr	0 (0.0)	4 (33.3)	0.209
	>20 yr	3 (75.0)	3 (25.0)	
	No alcohol	0 (0.0)	2 (16.7)	
	(Missing)	1 (25.0)	3 (25.0)	
Location	Gingiva	0 (0.0)	3 (25.0)	0.140
	Hard palate	0 (0.0)	1 (8.3)	
	Larynx	0 (0.0)	2 (16.7)	
	Retromolar Trigone	2 (50.0)	0 (0.0)	
	Tongue	2 (50.0)	6 (50.0)	
Previous RT (yes/no)	No	3 (75.0)	9 (75.0)	1.000
	Yes	1 (25.0)	3 (25.0)	

The associations between MPR and demographic and clinical characteristics were evaluated with two-sided nonparametric Wilcoxon rank sum test for continuous variables, and with two-sided Fisher’s exact test for categorical variables. No multiple comparison adjustment.

### Safety, tolerability and quality of life

SBRT at a dose of 18 Gy in three fractions, with dosimetric escalation to 24 Gy, combined with durvalumab was deemed safe. The adverse events related to SBRT, durvalumab or the combination of the two treatments together are summarized in Extended Data Table 1. The most common adverse event of grade 3 or above was treatment-related oral mucositis, which was experienced by 4 of 21 patients (19%) in the study. A common side effect, less than grade 3, was hypothyroidism due to durvalumab (*n* = 5, 23.8%). Most of the adverse events were related to SBRT treatment, but the frequency of these events did not increase with the dose of SBRT (Extended Data Table 1). Notably, no adverse events associated with surgical complications or delays were attributable to either SBRT or durvalumab.

**Extended Data Fig. 2:**
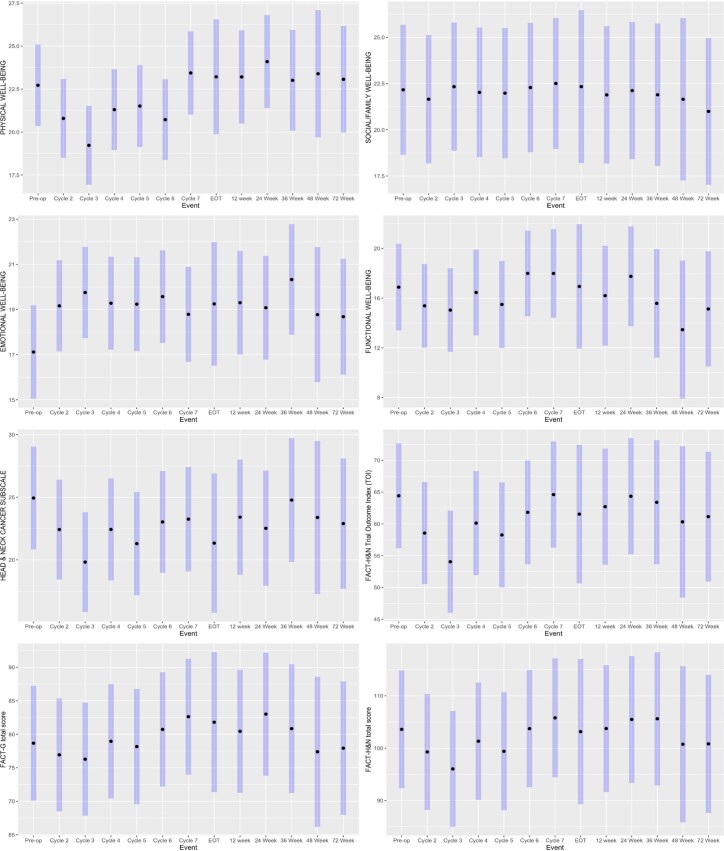
Longitudinal Assessment of Quality of Life. QOL data for each patient was collected using the FACT-H&N Version 4 Questionnaires. Sub scale scores and total scores were derived following the FACT-H&N Scoring Guidelines. This data is depicting information from 21 patients. A linear mixed model with random patient effect were used to test if the QOL scores changed by event timepoint for each of the sub scale scores and the total scores, and the Wald test results were reported. Multiple comparisons were adjusted using Benjamini and Hochberg’s method. Source data

We evaluated how this treatment affected our patients’ quality of life (QOL). QOL measures using the Functional Assessment of Cancer Therapy - Head and Neck (FACT-H&N) guidelines based on questionnaires given on the first day of SBRT and durvalumab and at each subsequent follow-up visit are summarized in Extended Data Table 2 and are longitudinally represented in Extended Data Fig. 2. To determine if QOL assessments changed over time, we used a linear mixed model with random patient effect to test if the QOL scores changed by event time for each point for each of the subscale scores and the total scores. Using a Wald test for significance and Benjamini and Hochberg’s method to adjust for multiple comparisons, on all evaluable summary scales, except physical well-being, the quality-of-life assessments showed a nonsignificant change in QOL from time of initial treatment to time of surgery. Physical well-being significantly declined post-surgery and did not recover post-operatively (Extended Data Table 2).

### Responders had an increase in effector T cells within the TME

Data from previous trials have shed light on potential mechanisms for enhancing therapeutic response and efficacy. Notably, when examining the TME, patients with no pathologic response had few T cells^5^. These data underscore what has been established in preclinical models of orthotopic HPV-unrelated HNSCC: that without an inflammatory trigger, such as SBRT, there is limited infiltration of immune cells to allow for a meaningful response to checkpoint inhibition^11,19–21^. Hypofractionated radiation, SBRT, has been shown to stimulate the immune response by increasing T cell abundance and activation in the TME and to lead to significant tumor growth reduction when combined with anti-PD-1/PD-L1 inhibitors^22,23^ (NCT02383212) (refs. ^24,25^).

**Fig. 2:**
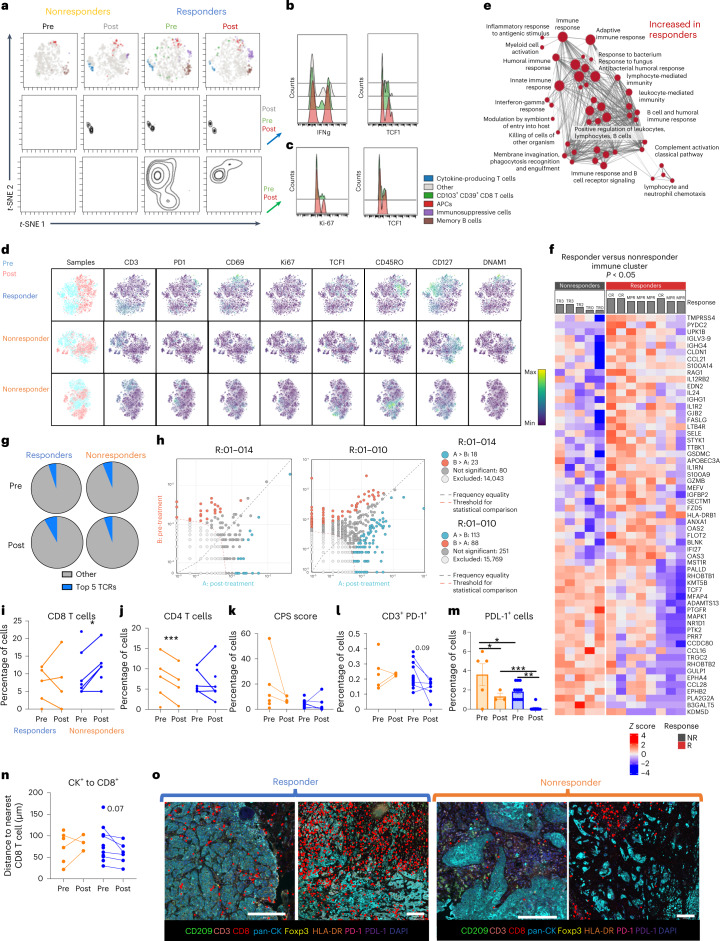
Responders had an increase in effector T cells within the TME. **a**, viSNE plot of CD45^+^ cells in the TME of patients pre- and post-treatment stratified by response and colored by cell type. Density plots of two clusters are included below the viSNE plot (nonresponders *n* = 6 patients, responders *n* = 10 patients). **b**, Histograms representing the proportion of cells, within the cytokine-producing T cell cluster, producing IFN-γ and expressing TCF1. **c**, Histograms representing the proportion of cells, within the CD103^+^CD39^+^ CD8 T cell cluster, expressing Ki-67 and TCF1. **d**, *t*-Distributed stochastic neighbor embedding (*t*-SNE) plots of CD45^+^ cells in a responder and two nonresponders. Blue represents TME samples taken before treatment, and pink represents TME samples taken after treatment. **e**, Magnification of the large cluster of pathways increased in responders pre-treatment represented in Extended Data Fig. 3b (nonresponders *n* = 5 patients, responders *n* = 8 patients). **f**, Significant genes determined using the GSEAPreranked module, based on an adjusted *P* value to account for multiple comparisons, which were used in the gene mapping identified pre-treatment (nonresponders *n* = 5 patients, responders *n* = 8 patients). The gray bars denote pathological response. **g**, Average of the top 5 TCR sequences (nonresponders *n* = 3 patients, responders *n* = 5 patients). **h**, Scatterplot with annotations depicting clones with more than 8 transcripts before and after treatment for patients 01-010 and 01-014. Red dots were clones significantly increased pre-treatment and blue dots were clones that were significantly increased post-treatment. **i**, Quantification of CD8^+^ T cells (CD3^+^CD8^+^) within the TME pre- and post-treatment (nonresponders *n* = 5 patients, responders *n* = 7 patients). **j**, Quantification of VECTRA images of CD4 T cells (CD3^+^CD8^−^Foxp3^−^) within the TME pre- and post-treatment (nonresponders *n* = 5 patients, responders *n* = 7 patients). **k**, CPS (no. PD-L1-expressing cells/no. CK^+^ cells) calculated from VECTRA images (nonresponders *n* = 6 patients, responders *n* = 12 patients). **l**, Quantification of VECTRA images of PD-1-expressing T cells (CD3^+^PD-1^+^) within the TME pre- and post-treatment (nonresponders *n* = 6 patients, responders *n* = 12 patients). **m**, The percentage of cells expressing PD-L1 from VECTRA images (mean ± s.e.m.) (nonresponders *n* = 6 patients, responders *n* = 12 patients). **n**, Quantification of the median proximity of a CD8^+^ cell to a CK^+^ cell from VECTRA images (nonresponders *n* = 6 patients, responders *n* = 12 patients). **o**, Representative images of the distance between cancer cells (CK^+^) and the nearest CD8 T cells (highlighted in red). Statistical significance was determined using a two-tailed paired Student’s *t*-test. *P < 0.05, **P < 0.01, ***P < 0.001. NR, non-responder; R, responder. Source data

To evaluate the immune cell response before and after treatment, we used cytometry by time of flight (CyTOF) on tumor biopsies and surgical resections on all evaluable patients (*n* = 19). We observed differences in CD45^+^ cells between responders and nonresponders within the TME at baseline and post-treatment (Fig. 2a). Within the cluster identified as cytokine-producing T cells (that is, INF-γ, IL-2, TNF-α), responders had an increase in mean florescent intensity of IFN-γ and TCF1 post-treatment (Fig. 2b). As reported in previous HNSCC clinical trials^26^, we observed an increase in CD103^+^CD39^+^ CD8^+^ T cells in responders both pre- and post-treatment, and increased Ki-67 and TCF1 expression, in this cell population, post-treatment (Fig. 2c). Post-treatment, responders had an increase in activated T cells (PD-1, CD69, Ki-67 and DNAM-1) as well as decreased expression of CD127 on CD45^+^CD3^−^ cells (Fig. 2d). Additionally, while a consistent increase in T cell memory (defined by CD45RO expression) or TCF1 expression was noted post-treatment in responders, nonresponders displayed more heterogeneity in these markers with no consistent pattern noted (Fig. 2d). Together, these data suggest that responders consistently have higher levels of CD103^+^CD39^+^ CD8^+^ T cells while nonresponders do not (pre- or post-treatment). Responders also had increases in T cell activation markers and T cell memory markers, while nonresponders exhibited variable expression of these markers.

**Extended Data Fig. 3:**
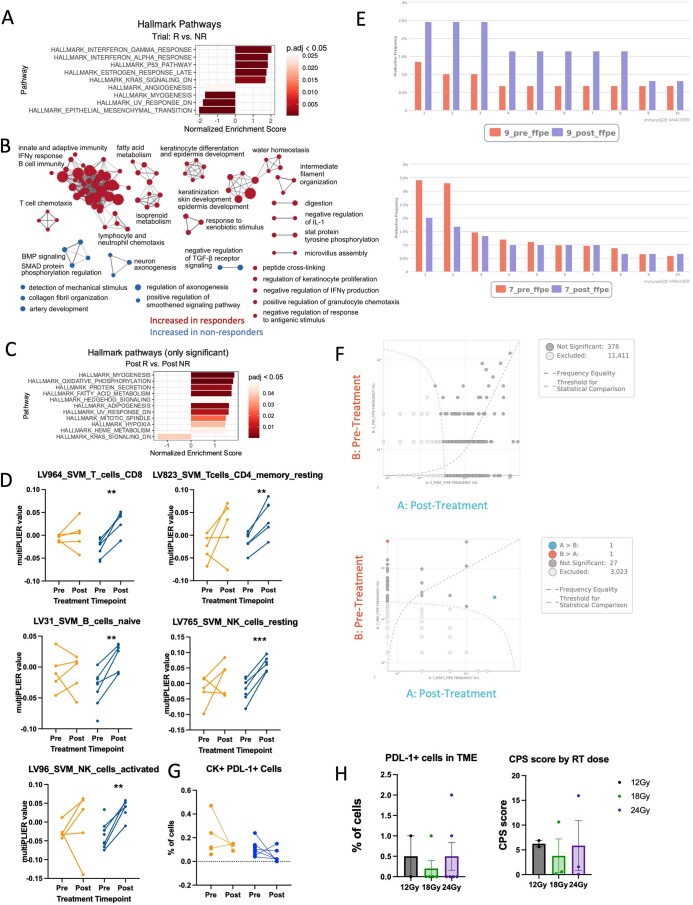
Responders have an increase in genes and pathways associated with inflammation. (**A**) Significant HALLMARK pathways that differed between responders and non-responders at baseline (non-responders n = 5 patients, responders n = 8 patients). (**B**) Gene mapping analysis of significant pathways increased in responders and non-responders at baseline using significant genes identified in GO biological and KEGG pathway analysis. Red clusters are increased in responders at baseline and blue clusters are increased in non-responders at baseline (non-responders n = 5 patients, responders n = 8 patients). (**C**) Significant HALLMARK pathways that differed between responders and non-responders post-treatment (non-responders n = 5 patients, responders n = 8 patients). (**D**) MultiPLIER cell type analysis of the RNA sequencing data (non-responders n = 5 patients, responders n = 8 patients) (LV964 non-responders p = 0.63 and responders p = 0.0024; LV823 non-responders p = 0.07 and responders p = 0.0059; LV31 non-responders p = 0.88 and responders p = 0.007; LV765 non-responders p = 0.28 and responders p = 0.001; LV96 non-responders p = 0.59 and responders = 0.0025). (**E**) Representative graphs depicting TCR clone expansion of the top 10 clones for two patients, a responder (01-009) and a non-responder (01-007). (**F**) Scatterplot with annotations depicting clones with more than 8 transcripts before and after treatment for patients 01-002 and 01-007. Light grey dots were not included in the analysis because they had less than 8 sequences. Dark grey dots are clones that were not significantly different between pre- and post-samples. Red dots were clones significantly increased pre-treatment and blue dots were clones that were significantly increased post-treatment. Dots along the Y and X axis are clones not present in the pre-sample or post-sample, respectively. Analysis was conducted using the ImmunoSEQ analyzer. (**G**) Quantification of PD-L1 expressing cancer cells (CK+) within the TME pre- and post-treatment (non-responders n = 6 patients, responders n = 12 patients). Non-responders p = 0.36 and responders p = 0.26. (**H**) Quantification of CPS score (12 Gy n = 2 patients, 18 Gy n = 3 patients, and 24 Gy n = 3 patients) and PD-L1+ cells (12 Gy n = 2 patients, 18 Gy n = 5 patients, 24 Gy n = 6 patients) in the TME post-treatment by VECTRA categorized by dose of SBRT given to the patient. Significance was determined by a two-way paired student’s t-test, *p < 0.05, **p < 0.01, and ***p < 0.001. The error bars represent the standard error of the mean (± SEM). Source data

To evaluate the underlying pathways that are driving this activation of T cells, we performed RNA sequencing on tumors pre- and post-treatment. Responders had an increase in expression of IFN-γ and IFN-α-associated genes pre-treatment (Extended Data Fig. 3a), and an increase in lymphocyte activation pathways (Extended Data Fig. 3b). At baseline, responders had an increase in expression of genes associated with an activated immune system: immune response to antigenic stimulation, humoral immune response, leukocyte-mediated immunity, IFN-γ response and innate immune response (Fig. 2e). A heatmap of genes that had significantly different expression levels between responders and nonresponders from this immune gene cluster reveals increases in genes that encode for proteins known to increase T cell trafficking to tumors (*CCL21*), transendothelial migration (*SELE*), tumor cell killing (*GZMB*) and antigen presentation (*HLA-DRB1*) (Fig. 2f). Responders also differed post-treatment from nonresponders by having a decrease in expression of genes associated with Kras signaling pathway, a known oncogenic pathway (Extended Data Fig. 3c). These findings confirm that an increase in immune infiltration and an immune activation signature pre-treatment correlated with treatment response. To validate these findings, we used a learned prediction model for cellular phenotyping based on RNA sequencing data, MultiPLIER^27^. MultiPLIER confirmed our findings from CyTOF. Relative to baseline, responders showed increased CD8^+^ T cells, CD4^+^ T cell memory cells, B cells and activated NK cells post-treatment (Extended Data Fig. 3d).

With multiple methodologies confirming increased tumor infiltrating lymphocytes (TILs) in the TME of responders post-treatment, we used T-cell receptor (TCR) sequencing to examine the effect of treatment on the TCR repertoire within the TME. Responders had an increase in expansion of the top five TCR sequences found in the TME compared with nonresponders (Fig. 2g). The individual expansions of the top ten TCR sequences for responder (01-009) and nonresponder (01-007) are shown in Extended Data Fig. 3e. TCR expansion has been associated with better outcomes in various cancer types, including HPV-unrelated HNSCC^26^. Given that it has been previously reported that within the TME, new T cells, not the preexisting ones, drive response to anti-PD-1 treatment^28^, we examined clones pre- and post-treatment in responders. We found that, although there are new clones that expand in responders post-treatment that were not detected at time of biopsy, preexisting T cells within the TME were also expanding post-treatment (Fig. 2h and Extended Data Fig. 3f).

To confirm that these effector T cells were truly infiltrating the TME and in proximity to cancer cells for the execution of cytotoxic T cell killing^29^, we used the multiplex multispectral spatial imaging platform VECTRA to determine the spatial relationship between TILs and cancer cells. VECTRA showed a significant increase in CD8^+^ T cells post-treatment in responders (Fig. 2i). Nonresponders also showed a decrease in CD4 T cells at time of surgery (Fig. 2j). There was no difference in baseline cancer cell-specific expression of PD-L1 levels (Extended Data Fig. 3g) or in combined positive score (CPS) (Fig. 2k) between responders and nonresponders or based on dose of radiation therapy (RT) (Extended Data Fig. 3h). While T cells expressing PD-1 tended to decrease post-treatment in responders (Fig. 2l), cumulative PD-L1 levels on all cells appeared to decrease in both responders and nonresponders, with the levels overall being lower in responders than nonresponders (Fig. 2m). Finally, evaluation of spatial proximity of T cells to cancer cells showed that the cancer cells were closer to CD8^+^ T cells after treatment in responders (Fig. 2n). Representative images of the distance between CD8^+^ T cells and cancer cells are shown in Fig. 2o.

### Multinucleated giant cells surround keratin pearls post-treatment in TME

**Extended Data Fig. 4:**
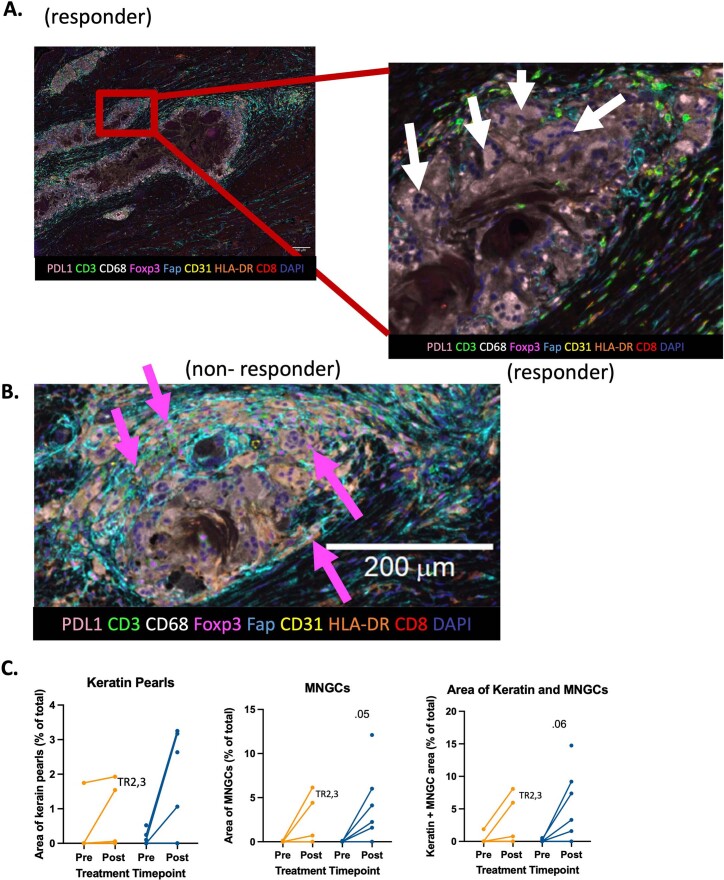
CD68+ Multinucleated giant cells surround keratin pearls post-treatment in responders’ TME. (**A**) Representative VECTRA image of the TME of a responder showing keratin pearls surrounded by MNGCs and CD3+ cells (highlighted in red). To the right, a zoom in on the image highlights MNGCs with white arrows. (**B**) Representative VECTRA image of the TME of a non-responder post-treatment showing Tregs surrounding a keratin pearl. Several Tregs highlighted by pink arrows. (**C**) Quantification of Keratin pearl area, MNGC area, and the combined area of both keratin pearls and MNGC (non-responder n = 5 patients, responder n = 7 patients). Image J was used for quantification and a paired two-sided student’s t-test was used to determine significance. Source data

In addition to cell type analysis, multispectral VECTRA imaging revealed differences in CD68^+^ multinucleated giant cells (MNGCs) within responders’ TME. Keratin pearls were often surrounded by TILs and MNGCs in responders (Extended Data Fig. 4a). Although the function of MNGCs is still unknown, MNGCs may be recruited by TILs to clear keratin pearls from the TME. Nonresponders appeared not to have developed keratin pearls post-treatment, or the keratin pearls were not surrounded by MNGCs. Only one nonresponder had keratin pearls surrounded by MNGCs, but the keratin pearls were also surrounded by large numbers of regulatory T cell (T_reg_) cells (Extended Data Fig. 4b). Quantification of the area of keratin pearls and MNGCs revealed a trend towards responders having an increase in keratin formation and subsequent MNGC density (Extended Data Fig. 4c). This suggests that MNGCs surrounding keratin pearls may be an indication that the immune system is in the process of clearing cancer cells and their presence at surgery, if cancer cells are still detectable, can potentially serve as a biologic correlative of response to neoadjuvant SBRT + anti-PD-L1.

### Responders increase antigen presentation and TCR expansion

**Fig. 3:**
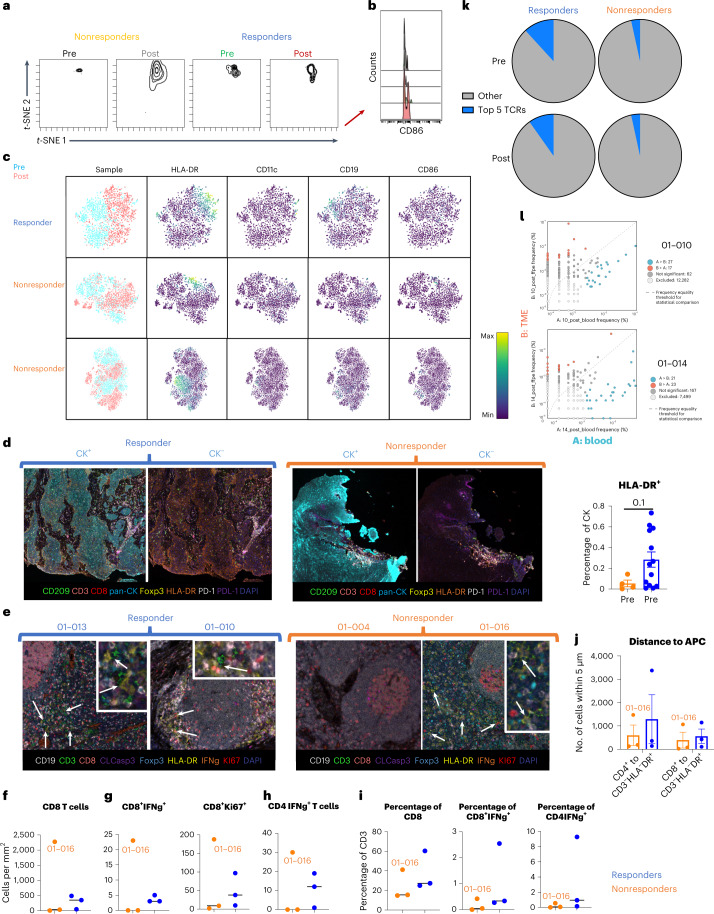
Responders increase antigen presentation and TCR expansion. **a**, Density plots of the antigen-presenting cluster from Fig. 2a (nonresponders *n* = 6 patients, responders *n* = 10 patients). **b**, Histogram representing the amount of CD86 expressed by cells within the antigen-presenting cluster. **c**, *t*-SNEs of CD45^+^ cells in a responder and two nonresponders. Blue represents TME samples taken before treatment, and pink represents TME samples taken after treatment. **d**, Left, representative VECTRA images of HLA-DR expression (left images with CK expression, right images without CK expression). Right, quantification of VECTRA images of HLA-DR expression on CK^+^ cells within the TME pre-treatment (mean ± s.e.m.) (nonresponders *n* = 4 patients, responders *n* = 12 patients). **e**, Representative VECTRA images of DLNs collected at time of surgery. DC–T cell interactions are highlighted with white arrows. **f**, Density of CD8^+^ T cells (CD3^+^CD8^+^) in the DLNs (nonresponders *n* = 3 patients, responders *n* = 3 patients). **g**, Density of activated CD8^+^ T cells (CD3^+^CD8^+^IFN-γ^+^) and replicating CD8^+^ T cells (CD3^+^CD8^+^Ki67^+^) in the DLNs (nonresponders *n* = 3 patients, responders *n* = 3 patients). **h**, Density of activated CD4^+^ T cells (CD3^+^CD8^−^Foxp3^−^IFN-γ^+^) in the DLNs (nonresponders *n* = 3 patients, responders *n* = 3 patients). **i**, Percentages of CD3^+^ cells that are CD8^+^ T cells, activated CD8^+^ T cells and activated CD4^+^ T cells (nonresponders *n* = 3 patients, responders *n* = 3 patients). **j**, Quantification of how many T cells were within 15 μm of APCs (CD3^−^HLA^−^DR^+^) (mean ± s.e.m.) (nonresponders *n* = 3 patients, responders *n* = 3 patients). **k**, Average of the top 5 TCR sequences pre- and post-treatment in the blood (nonresponders *n* = 3 patients, responders *n* = 5 patients). **l**, Scatterplot with annotations depicting clones with more than 8 transcripts after treatment in the TME and blood for patients 01-010 and 01-014. Red dots are clones significantly increased in the TME and blue dots are clones that were significantly increased in the blood. Dots along the *y* and *x* axes are clones not present in the TME or blood post-treatment, respectively. Statistical significance was determined using a two-tailed paired Student’s *t*-test. *P < 0.05, **P < 0.01, ***P < 0.001. Source data

**Extended Data Fig. 5:**
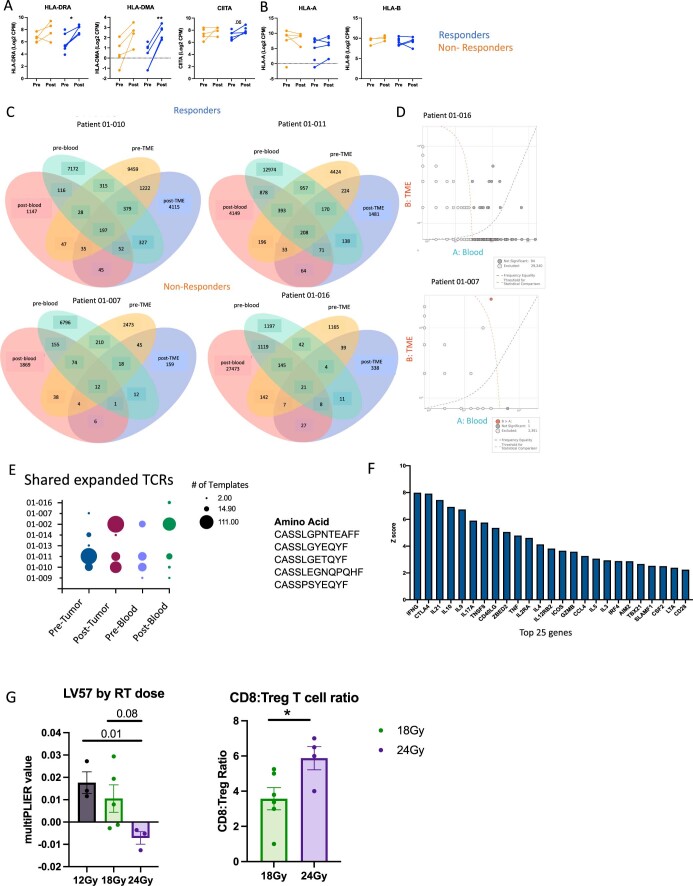
Antigen presentation is increased in responders while Tregs are decreased. (**A**) RNA expression of genes associated with MHC II expression (CIITA) and MHC II genes (HLA-DRA and HLA-DMA) (non-responders n = 5 patients, responders n = 8 patients) (HLA-DRA non-responders p = 0.25 and responders p = 0.02; HLA-DMA non-responders p = 0.07 and responders p = 0.0014; CIITA non-responders p = 0.19 and responders p = 0.06). (**B**) RNA expression of MHC I genes (HLA-A and HLA-B) (non-responders n = 5 patients, responders n = 8 patients). (**C**) Venn diagrams depicting TCR sequences shared between the blood and the TME pre- and post-treatment in representative responders and non-responders (non-responders n = 3 patients, responders n = 5 patients). (**D**) Scatterplot with annotations depicting clones with more than 8 transcripts after treatment in the TME and blood for patients 01-016 and 01-007. Light grey dots were not included in the analysis because they had less than 8 sequences. Dark grey dots are clones that were not significantly different between post-treatment TME and blood samples. Red dots were clones significantly increased the TME and blue dots were clones that were significantly increased in the blood. Dots along the Y and X axis are clones not present in the TME or blood post-treatment, respectively. (**E**) Dot plot of template expansion of the top 5 TCR amino acid sequences shared between the most samples. Any patient sample was required to have at least two templates to be included. The top 5 amino acid sequences found are on the right. Analysis was conducted using the ImmunoSEQ analyzer. (**F**) Top 25 genes for LV57 identified by MultiPLIER (non-responders n = 5 patients, responders n = 8 patients). (**G**) Quantification of LV57 by dose of RT (12 Gy n = 3 patients, 18 Gy n = 5 patients, 24 Gy n = 3 patients) and quantification of the CD8:Treg Ratio in tumor samples post-treatment by VECTRA (p = 0.04) (18 Gy n = 5 patients, 24 Gy n = 4 patients). Statistical significance was determined using a two-tailed unpaired student’s t-test, *p < 0.05 and **p < 0.01. The error bars represent the standard error of the mean (± SEM). Source data

To effectively activate T cells, tumor antigens must be presented to T cells via antigen-presenting cells (APCs) such as dendritic cells (DCs). Preclinical studies demonstrate the importance of T cell priming in the draining lymph nodes (DLNs) for response to SBRT combined with immunotherapies^21^. Although T cell priming is primarily in the DLNs, DCs acquire antigens in the TME and T cells require additional TCR stimulation within the TME to maintain activation and decrease efflux. Although we did not observe a difference in the amount of APCs in the TME (Fig. 2a), responders had increased expression of the costimulatory molecule CD86 in the APC cluster, indicating increased potential to activate T cells (Fig. 3b). Comparing responders with nonresponders revealed that the responders had an increase in HLA-DR expression post-treatment and an increase in B cells (CD19^+^) pre-treatment (Fig. 3c). Transcriptomic analysis also revealed an increase in expression of MHC II antigen presentation-related genes, but not an increase in expression of MHC I-associated genes in responders post-treatment (Extended Data Fig. 5a,b). Using VECTRA, we also evaluated the level of MHC II expression on cancer cells in the TME, as cancer cells represent one of the nonclassical APCs in the TME and have been shown to correlate with response in preclinical models and in clinical trial outcomes^30^. Similarly, we observed that response to treatment in this trial correlated with higher baseline levels of HLA-DR expression on cancer cells (CK^+^) in the TME (Fig. 3d). These data suggest that responders have an increase in antigen presentation machinery in the TME.

As the majority of antigen presentation and subsequent T cell expansion/activation occurs in the DLNs, we evaluated DLN tissue from responders and nonresponders collected at time of surgery with VECTRA. We evaluated antigen presentation by T cell division (Ki-67^+^), T cell cytokine production and the proximity between T cells and APCs. Representative images of DLNs from responders and nonresponders are presented in Fig. 3e. A noticeable difference was observed in patient 01-016 (nonresponder) compared with the other samples analyzed. Patient 01-016, with notable edema before surgery, had a higher density of cells in the DLNs compared with other patients (Fig. 3f–h). We observed an increase in density of CD8^+^ T cells in responders’ TME (Fig. 3f). There was also an increase in the density of IFN-γ^+^ CD8 T cells and replicating CD8^+^ T cells (Ki-67+) (Fig. 3g), as well as IFN-γ^+^ CD4 T cells (Fig. 3h). To highlight that these dense structures within DLNs were primarily composed of lymphocytes, plotted as a percentage of CD3^+^ cells, we observe enrichment of CD8^+^ T cells, activated CD8^+^ T cells and activated CD4^+^ T cells in responders (Fig. 3i). As T cell activation by APCs is mediated by cell-to-cell contact via MHC molecules and TCRs, we also evaluated the distance between APCs and T cells. We found that there were more CD4^+^ and CD8^+^ T cells near APCs in responders (Fig. 3j). DC–T cell interactions are highlighted by white arrows in Fig. 3e. As DC–T cell interactions in the DLNs classically result in an expansion of T cell clones that will leave the DLNs and enter circulation, we also sequenced T cells in the blood pre- and post-treatment to evaluate changes in the TCR repertoire. We found that responders already had a high percentage of clonal expansion before treatment and continued to maintain a high percentage of clonally expanded T cells post-treatment, while nonresponders did not (Fig. 3k). The clones in the blood had overlap with the clones present in the TME post-treatment (Fig. 3l and Extended Data Fig. 5c,d). To determine if patients with this HPV-unrelated HNSCC recognize similar tumor antigens, we explored the TCR sequencing for shared expansion of T cell clones, with the same amino acid sequences that define the antigen-binding pocket. We observed that the top five TCR clones shared by the most samples sequenced expanded in all patients sequenced either in the blood and/or tumor and that these clones were not specific to a virus (cytomegalovirus) (Extended Data Fig. 5e), suggesting that this patient population may have shared tumor antigens.

### Responders decrease immunosuppressive cells in the TME

**Fig. 4:**
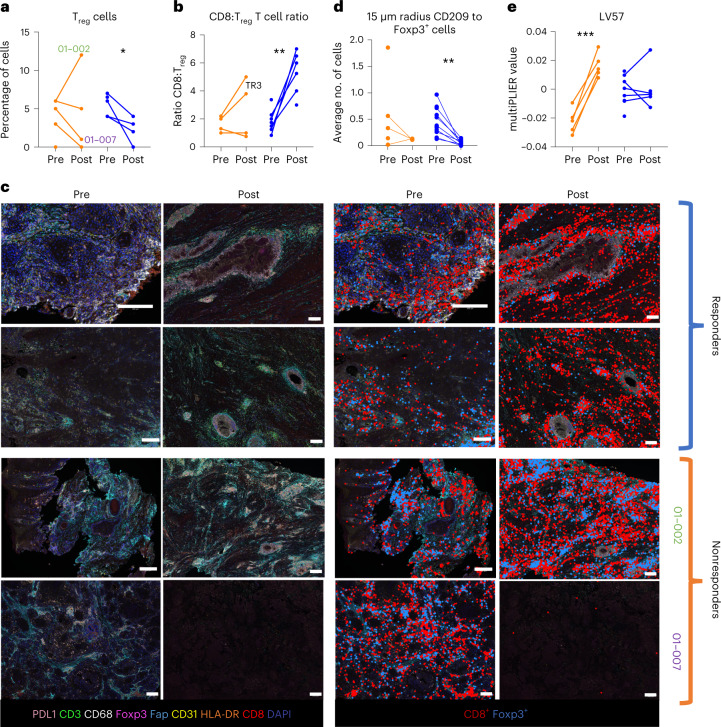
Responders decrease immunosuppressive cells in the TME. **a**, VECTRA image quantification of T_reg_ cells (CD3^+^CD8^−^Foxp3^+^) in patient tumors pre- and post-treatment (nonresponders *n* = 5, responders *n* = 7). Nonresponders *P* = 0.84 and responders *P* = 0.03. **b**, Ratio of CD8^+^ T cells (CD3^+^CD8^+^) to T_reg_ cells in the TME pre- and post-treatment (nonresponders *n* = 5 patients, responders *n* = 7 patients). Nonresponders *P* = 0.28 and responders *P* = 0.0032. **c**, Representative VECTRA image of the TME pre- and post-treatment. Patients 01-002 and 01-007 representing two different kinds of non-responders to treatment. To the right, CD8^+^ T cells (red) and T_reg_ cells (blue) are highlighted. **d**, VECTRA quantification of the distance between T_reg_ cells and DCs (CD209^+^) in the TME of responders and nonresponders pre- and post-treatment (nonresponders *n* = 5 patients, responders *n* = 7 patients). Nonresponders *P* = 0.38 and responders *P* = 0.0024. **e**, MultiPLIER quantification of LV57 (nonresponders *n* = 5, responders *n* = 8). Nonresponders *P* = 0.0006 and responders *P* = 0.99. Statistical significance was determined using a two-tailed paired student’s *t*-test. *P < 0.05, **P < 0.01, ***P < 0.001. Source data

A long-term, robust T cell-mediated response to tumor-specific antigens requires not only activation, but also maintenance of this activation within the TME. Suppression post-activation can be mediated by cells such as T_reg_ cells. Our VECTRA spatial imaging analysis revealed that while responders had an increase in T cells and a decrease in T_reg_ cells, the nonresponders had two main patterns of failure. Except for one nonresponder (patient 01-002), both responders and nonresponders consistently had decreased T_reg_ cells within the TME (Fig. 4a). However, in nonresponders, this was accompanied by a large decrease in T cells. Plotted as a ratio of CD8^+^ T cells:T_reg_ cells, a clear and significant difference can be observed between nonresponders and responders (Fig. 4b). Representative images highlighting the differences in T_reg_ and CD8^+^ T cell abundances between responders and the two types of nonresponders pre- and post-treatment are depicted in Fig. 4c. T_reg_ cells can act on effector T cells in three main ways: (1) by decreasing antigen presentation of DCs by cell-to-cell contact; (2) by releasing immunosuppressive signals into the TME such as IL-10; and (3) by sequestering the pro-survival factor IL-2 (ref. ^31^). We observed that the amount of T_reg_ cells near DCs decreased in responders while remaining unchanged in nonresponders (Fig. 4d). To examine indirect means by which T_reg_ cells are suppressing effector T cells within the TME, we used MultiPLIER to evaluate gene expression associated with T_reg_ activity. We observed a large increase in LV57, which represents an increase in expression of genes known to encode proteins associated with T_reg_ cell-mediated immunosuppression (CTLA-4, IL-10, IL2RA and ICOS), in nonresponders (Fig. 4e and Extended Data Fig. 5f). Next, we asked if these differences were based on the dose of RT given to each patient, independent of response, as we observed that a combined dosimetric dose of 24 Gy was associated with improved outcomes (Fig. 1e). We found that after treatment, patients that received 24 Gy had lower levels of LV57 (Extended Data Fig. 5g). Similarly, the ratio of CD8^+^ T cells to T_reg_ cells increased in patients that received 24 Gy (Extended Data Fig. 5g).

### Circulating lymphocytes and metabolites correlate with response

**Fig. 5:**
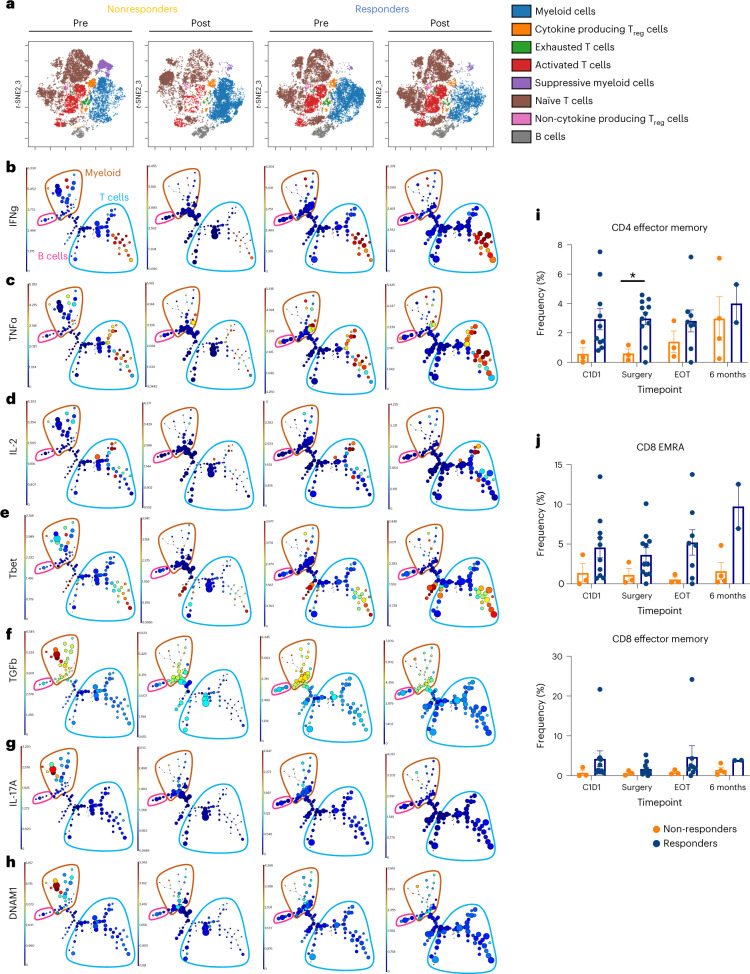
Circulating lymphocytes and metabolites correlate with response. **a**, viSNE depicting cell populations identified in the blood (nonresponders *n* = 3 patients, responders *n* = 9 patients). **b**, Expression of IFN-γ, **c**, TNF-α, **d**, IL-2, **e**, Tbet, **f**, TGF-β, **g**, IL-17A and **h**, DNAM in different populations in the blood. B cells are in pink, T cells are in blue and myeloids are in brown. **i**, Quantification of CD4^+^ effector memory T cells (CD3^+^CD19^−^CD56^−^CD14^−^CD4^+^CD8^−^Foxp3^−^CD45RA^−^CD27^−^) at baseline, time of surgery, EOT and 6-month follow-up (mean ± s.e.m.). C1D1 *P* = 0.57, surgery *P* = 0.016 and EOT *P* = 0.31. **j**, CD8^+^ EMRA T cells (CD3^+^CD19^−^CD56^−^CD14^−^CD4^−^CD8^+^CD45RA^+^, CD27^−^) (C1D1 *P* = 0.22, surgery *P* = 0.18, EOT *P* = 0.12 and 6-month follow-up *P* = 0.026) and CD8^+^ effector memory T cells (CD3^+^CD19^−^CD56^−^CD14^−^CD4^−^CD8^+^CD45RA^−^, CD27^−^) (C1D1 *P* = 0.37, surgery *P* = 0.28 and EOT *P* = 0.44) at baseline, time of surgery, EOT and 6-month follow-up (mean ± s.e.m.). For blood analysis at various time points in both **i** and **j**, C1D1 (nonresponders *n* = 3 patients, responders *n* = 10 patients), surgery (nonresponders *n* = 3 patients, responders *n* = 11 patients), EOT (nonresponders *n* = 3 patients, responders *n* = 8 patients) and for 6-month follow-up (nonresponders *n* = 4 patients, responders *n* = 2 patients). A two-tailed Student’s *t*-test was used to determine statistical significance, *P < 0.05. Source data

**Extended Data Fig. 6:**
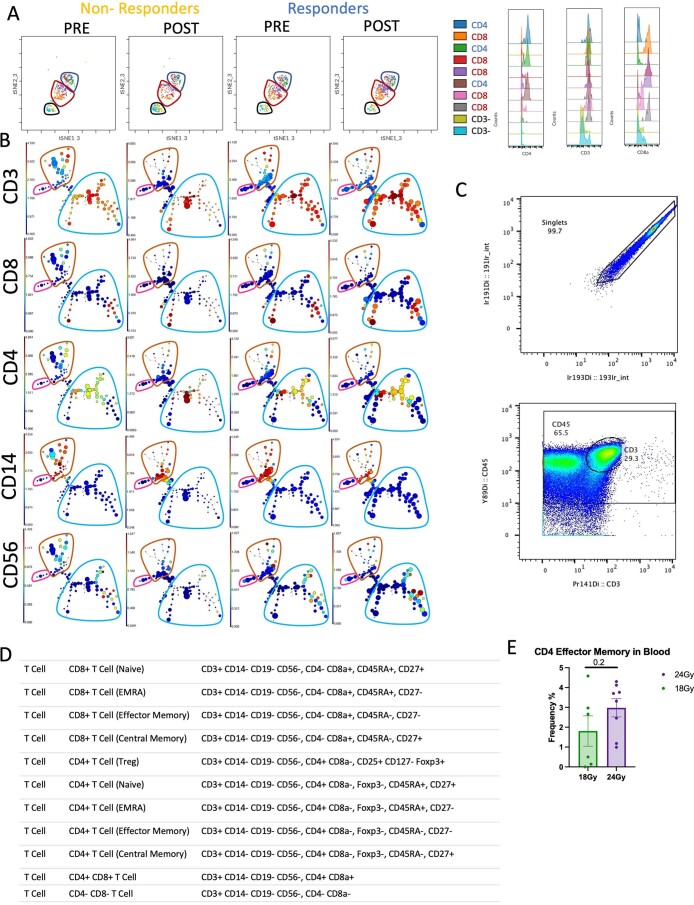
Clustering of cell populations in the blood. (**A**) Defining CD4^+^ and CD8^+^ T cells withing the activated T cell cluster (non-responders n = 3 patients, responders n = 9 patients). (**B**) CITRUS populations identified using differentiating markers (CD3, CD4, CD8, CD14, CD56). (**C**) Gating strategy for CD45^+^ cells used for clustering analysis. (**D**) Gating strategy for memory T cells. (**E**) Quantification of Effector Memory CD4 T cells post treatment by dose of radiation, at time of surgery (18 Gy n = 6 patients, 24 Gy n = 8 patients). Statistical significance was determined using a two-tailed unpaired student’s t-test. The error bars represent the standard error of the mean (± SEM). Source data

Undoubtedly, changes in the circulatory lymphocytes represent a prime opportunity for biomarker development, given that a blood draw is minimally invasive and relatively easy to process. Given that we observed similar TCR expansion in the blood to the TME, we sought to determine how changes in other circulatory lymphocytes compare with the TME. Similar to our findings within the tumor compartment, CyTOF analysis of the blood showed that responders had increases in activated T cells, decreases in suppressive immune cells and increases in naïve T cells (Fig. 5a). Within the activated T cells cluster, responders had increases in density of both CD4^+^ and CD8^+^ T cells (Extended Data Fig. 6a). We then used a clustering analysis to better understand the populations changing between responders and nonresponders. Cell populations were identified using differentiating markers (CD3, CD4, CD8, CD14, CD56) (Extended Data Fig. 6b) and the gating strategy for CD45^+^ cells input into the clustering analysis are provided in Extended Data Fig. 6c. Both CD4^+^ and CD8^+^ T cells expressed high levels of proinflammatory cytokines TNF-α and IFN-γ in responders (Fig. 5b,c). We also observed an increase in Th1-related proteins (Tbet and IL-2) in responders (Fig. 5d,e). A noticeable population that was elevated in nonresponders compared with responders pre-treatment was a myeloid population that expressed TGFβ, IL-17A and DNAM-1 (Fig. 5f–h). Importantly, we also observed an increase in memory T cells circulating in responders. Effector memory CD4^+^ T cells were significantly increased in responders at the time of surgery (Fig. 5i and Extended Data Fig. 6d). Post-surgery, effector memory cells re-expressing CD45RA (EMRA), CD8^+^ T cells and effector CD8^+^ memory T cells were increased in responders at the 6-month follow-up time point (Fig. 5j and Extended Data Fig. 6d). We also sought to determine if the circulatory effector memory CD4^+^ T cells were increased in patients given 24 Gy. Compared with 18 Gy, patients treated with 24 Gy tended to have an increase in this cell population (Extended Data Fig. 6e). These memory T cell populations may prove to be a correlate of long-term response in these patients upon further follow-up.

**Extended Data Fig. 7:**
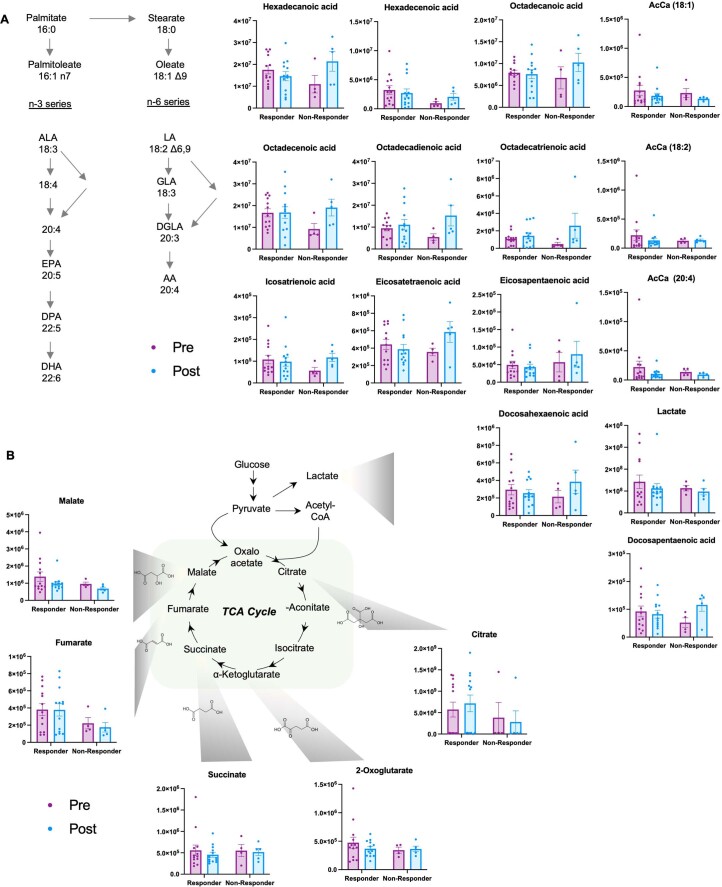
An increase in free-fatty acid metabolism correlated with response while decreased acyl-carnitines correlated with poor treatment response. (**A**) Quantification of circulating free-fatty acids from serum collected from patients pre- and post-treatment (mean+/− SEM) (pre non-responders n = 4 patients, post non-responders n = 5 patients, pre responders n = 13 patients, post responders n = 13 patients). (**B**) Quantification of circulating metabolites associated with the Krebs cycle pre- and post-treatment (mean +/− SEM) (pre non-responders n = 4 patients, post non-responders n = 5 patients, pre responders n = 13 patients, post responders n = 13 patients). Source data

To further identify circulating differences between responders and nonresponders, we performed plasma metabolomics. Consistent with our pathway analysis of RNA sequencing data (Extended Data Fig. 3c), plasma metabolomics analyses showed a differential treatment response in responders versus nonresponders with respect to the circulating levels of several free fatty acids (including saturated, monounsaturated and polyunsaturated fatty acids), all increasing in nonresponders following the treatment (Extended Data Fig. 7a). These changes were accompanied by decreases in the levels of acyl-carnitines (especially oleoyl-, linoleyl- and arachidonyl-carnitine) post-treatment, especially in nonresponders, suggestive of altered fatty acid oxidation, a hallmark of CD8^+^ memory T cell activation^32,33^. Indeed, analysis of circulating levels of carboxylic acids as a proxy for cellular mitochondrial metabolism suggests differential fluxes through the Krebs cycle in responders versus nonresponders, with significantly lower levels of fumarate and malate compared with responders, especially in response to the treatment (Extended Data Fig. 7b).

### Noninvasive markers that predict response to treatment

**Extended Data Fig. 8:**
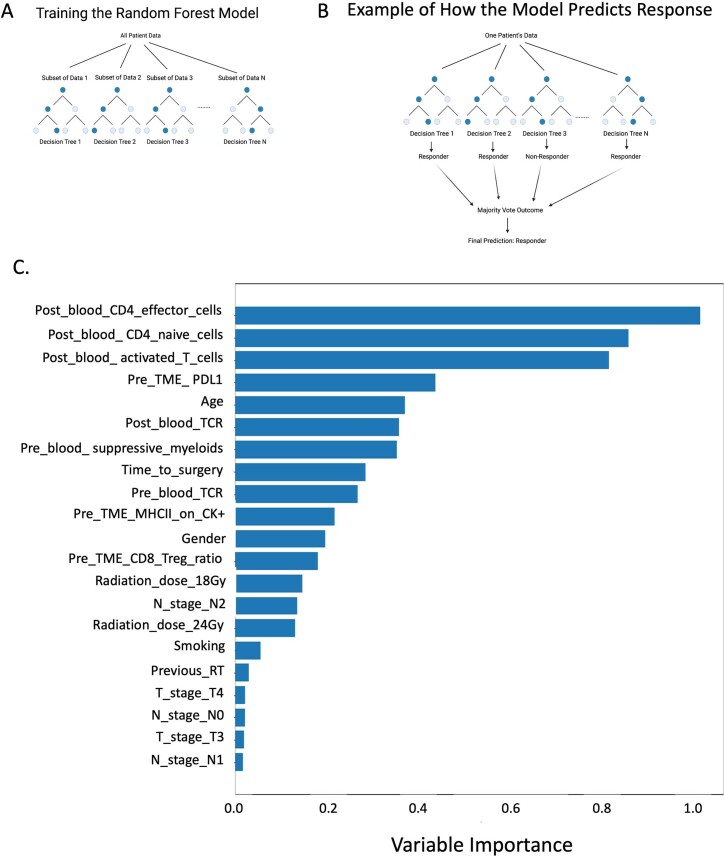
Predictor importance plot for the random forest model averaged across the random initializations. (**A**) Diagram depicting how the random forest model was trained. (**B**) Diagram depicting an example of how the model will determine if a patient responds to treatment. (**C**) Predictor importance is computed using the mean decrease in Gini index and plotted relative to the CD4^+^ effector T-cell importance, which had the maximum mean decrease in Gini index among the predictors. This figure was made with Python version 3.6.3 and Matplotlib version 3.2.2. Source data

Predicting response to therapy pre-treatment and at time of surgery could significantly improve patient outcomes and QOL and minimize morbid procedures and associated toxicities. Our goal was to determine whether response could be predicted exclusively from noninvasive means (blood) or biopsy samples taken at time of diagnosis. With a small sample size of 16 evaluable patients, we chose to narrow down our possible predictive variables to those that had already shown correlations with outcome in previous literature. This included clinical information such as age, sex, radiation dose, time to surgery, smoking history and previous radiation. We then added correlates in the blood that we identified throughout this manuscript, such as TCR expansion, activated T cells, memory T cells and immunosuppressive cells. From the tumor, we included variables such as CD8^+^ cells:T_reg_ ratio, PD-L1^+^ expression on all cells and MHC II expression on cancer cells. We then used a random forest machine learning algorithm to determine whether these variables could accurately predict response (Extended Data Fig. 8a,b). Importance weighting for each variable is represented in Extended Data Fig. 8c for this cohort. The variables that appeared to be most important for predicting response were from the blood taken pre-surgery (naïve T cells, activated T cells and CD4^+^ effector memory T cells). We also ran the model again, incorporating, directly, pre- and post-treatment differences (pre–post) into the model. This did not increase the accuracy of the model. Although preliminary, this predictive model was more accurate (82.5% accuracy) than the CPS, which showed no correlation with outcome or with randomly assuming that each patient would be a responder (75%). We plan to evaluate this prediction model in the ongoing phase II trial.

## Discussion

We report that the administration of neoadjuvant radiation in combination with durvalumab is safe for treating locally advanced HPV-unrelated HNSCCs. The safety was established not only in the context of lack of adverse events with surgical resection, but also in terms of long-term side effects. We report high rates of pathological MPR and CR for evaluable patients treated with MTD (75%) and a pathologic tumor response (pTR) pTR2 or greater of 83% among all evaluable study participants. In the expansion cohort, none of the patients who achieved pathological MPR or CR received adjuvant RT or CRT and none have recurred at the time of reporting of this publication. We observed an increase in survival when comparing our survival outcomes (80.1%) with historical outcomes (40–65%) at 16 months for standard of care described in American Joint Committee on Cancer (AJCC) 8th Edition^34^.

Although we are not able directly to compare our results with other recently published clinical trials in this space due to differences in patient population and dosing regimens, our trial design in HPV-unrelated HNSCC achieved the highest reported rates of pathological MPR and CR of 75% (89% for those treated with 24 Gy), despite having patients with more advanced disease. There were no pathological MPRs or CRs in either the neoadjuvant pembrolizumab trial^5^ or the neoadjuvant anti-OX40 trial^26^. In the neoadjuvant nivolumab or nivolumab plus ipilimumab trials, the pathological reported outcomes ranged from 8% (ref. ^6^) to 17% (ref. ^35^) in the nivolumab alone arm compared with 20% (ref. ^6^) or 35% (ref. ^35^) in the combination nivolumab plus ipilimumab arm. A landmark trial that has defined response rates to adding immunotherapy in the definitive setting is the JAVELIN trial^36^. Although it was not a neoadjuvant trial, JAVELIN’s disappointing results dampened the enthusiasm for integrating immunotherapy in patients with HNSCC. While it is conceivable that patient selection could have accounted for the lack of difference between the groups, one must wonder if the radiation fractionation and/or radiation treatment volumes could have negated any benefit immunotherapy would have offered^5,6,35^. Altogether, these results are consistent with those that we and others have reported in preclinical experiments: that concurrent administration of anti-PD-L1 with RT can significantly reduce tumor growth compared with either modality alone^23,37^.

Optimal RT dose and fractionation and optimal time to surgery remain unknown. Initially we administered 12 Gy in two fractions and escalated to 18 Gy in three fractions, but it was only when the GTV was heated to 24 Gy in three fractions that MPR or CR was consistently observed. Similarly, in a separate trial that included mostly HPV-positive patients with HNSCC, using a similar hypofractionated regimen, observed pathological response rates ranged between 67% and 86% (ref. ^38^). Leidner and colleagues^38^ reported that 40 Gy in five fractions did yield increased toxicity, but 24 Gy in three fractions was established to be a safe dose regimen. Additionally, maximal pathological response was observed after a minimum of 5 weeks from the end of radioimmunotherapy, consistent with data reported previously for p16^+^ locally advanced HNSCC^38^. This likely reflects the time required not only to clear the disease but also to develop systemic immune memory^39^. The latter is especially true in the context of anticipated surgical neck dissection and the need for immune priming to first develop in the DLNs before immune effector memory development^40^.

**Fig. 6:**
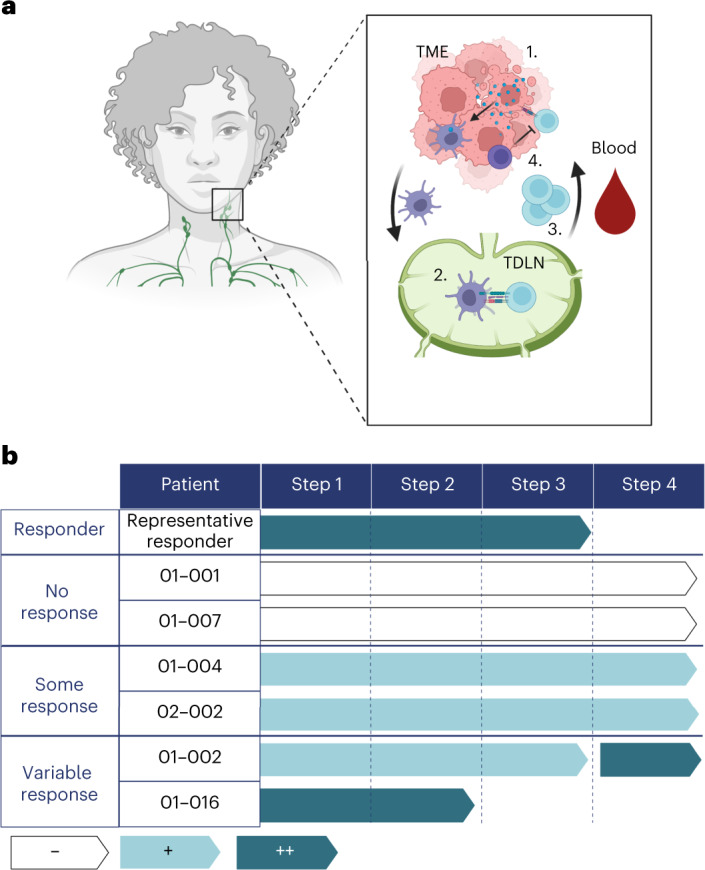
Summary of mechanisms underlying treatment failure. **a**, Diagram of the steps involved in a successful T cell-mediated anti-tumor response: (1) Initial TIL infiltration, antigen presentation and clonal expansion. (2) Antigen presentation in the lymph nodes and activation/replication of T cells. (3) Clonal expansion and T cell activation in the blood post-treatment. (4) Immune suppression by T_reg_ cells in the TME after treatment. **b**, Summary of each patient who failed therapy compared with the average responder. No evidence of a step in a patient is depicted with ‘−’, some evidence of a step is indicated with ‘+’ and a lot of evidence of a step is indicated with ‘++’. TDLN, tumor draining lymph node.

Four key steps for activating and maintaining a robust T cell-mediated anti-tumor response were consistently observed in the responders tested in this clinical trial. These steps, similar to those previously identified by others^41^, include: (1) initial TIL infiltration, antigen presentation and clonal expansion; (2) antigen presentation in the lymph nodes and activation/replication of T cells; (3) clonal expansion and T cell activation in the blood post-treatment; and (4) immune suppression in the TME post-treatment (Fig. 6).

Similar to what has been previously reported, PD-L1 expression on cancer cells did not correlate with response to SBRT and anti-PD-L1 in our cohort^42^. CPS also did not correlate with treatment response in the context of radiation and anti-PD-L1 therapy. Instead, (step1) responders’ pre-treatment TMEs were characterized by increases in expression of inflammatory gene pathways and in total number of CD103^+^CD39^+^ CD8 T cells. CD103^+^CD39^+^CD8^+^ T cells may serve as tumor antigen-specific T cells and have been associated with improved response to immunotherapy in HPV-unrelated HNSCC^26,43^. The influx of TILs and increased inflammation seen in responder TMEs pre-treatment may be the result of baseline increases in antigen presentation. This is corroborated by observed increases in responders’ MHC II cancer cell expression pre-treatment. Although CD8^+^ T cells are primed through interaction with MHC I, the importance of CD4^+^ T cell priming for an enhanced and sustained CD8^+^ T cell response is well-documented^44,45^.

The importance of antigen presentation was further substantiated by examining changes in the DLNs (step 2). As determined spatially by the distance from DCs to T cells and quantitatively by the number of replicating T cells, responders exhibited changes consistent with increased antigen presentation in the DLNs. Further evidence supporting enhanced antigen presentation and T cell priming in the DLNs was the observed increase in circulating clonal expansion, a direct consequence of priming in the DLNs^46^. Clonal expansion of TCRs in the blood of responders correlated with response, a finding that may serve as a minimally invasive surrogate of response, and a reflection of antigen presentation within the DLNs.

In the circulation, not only did TCR expansion serve as a correlate of response, but so did TIL activation post-treatment (step 3). In a manner that mirrored that of the TME, the increase in T cell activation was accompanied by increases in CD103^+^CD39^+^ CD8^+^ T cells. Also, the TCR clonal expansion observed in the blood of the responder population post-treatment was noted in the TME, with a high degree of overlap in TCR clonality. This suggests that in responders post-treatment, tumor-specific cytotoxic T cells are infiltrating the TME, and, as shown by our spatial analysis, are ultimately localized near the cancer cells. Past studies have shown that the numbers of CD8^+^ T cells close to cancer cells are more predictive of outcome than the overall numbers of CD8^+^ T cells, including CD8^+^ T cells within the stroma^47^.

Finally, maintaining a sustained immune response relies on minimizing immunosuppressive immune populations within the TME (step 4). Especially for poorly immunogenic tumors, once a T cell response develops, immunosuppression ensues as a result of a negative feedback control aimed at reducing effector T cell function^13^. In particular, the presence of T_reg_ cells has been shown, both in preclinical and clinical settings, to negatively influence response to immunotherapy^11^. The TME of responders in our study had low T_reg_ to CD8^+^ T cell ratios post-treatment. Although individual steps in activating and maintaining an effective anti-tumor response were observed, it is critical to emphasize that these interactions are interdependent^41^. The intimate interplay between these variables, rather than any individual component, will likely determine patient outcome^48^.

Although we observed excellent response rates to this treatment regimen, not all patients exhibited a CR. Based on extensive phenotyping of each patient profile at various time points using multiple analyses described in Figs. 2–5, four distinct patterns emerged that suggest why some patients failed to respond (Fig. 6). For example, patient 01-002 exhibited a large increase in T_reg_ cells in their TME although they had high levels of effector T cells (Fig. 4a,c). This patient may have benefited from combining this treatment with an immunotherapy targeting T_reg_ cells such as anti-CD25 (refs. ^11,49^). Patients 01-004 and 01-007 had low levels of antigen presentation and T cell replication in their DLNs (Fig. 3), possibly indicative of a lack of antigen for presentation, or a deficiency in the TCR repertoire capable of recognizing antigens being presented. As our data suggest that this patient population has shared tumor antigens, it holds promise for the development of a tumor vaccine, or a treatment to stimulate antigen presentation by DCs^26,50^. Finally, patient 01-016 did not respond despite mirroring many of the responder baseline traits. Patient 01-016 had notable edema pre-surgery. High interstitial pressure in the DLNs has been previously associated with decreased T cell activity and previous studies suggest that this patient may benefit from a therapy to relieve the interstitial pressure^51,52^.

The results of this phase I/Ib clinical trial highlight the importance of identifying the point at which a patient’s immune system is hindered in its recognition and elimination of cancer cells. To guide treatment selection, the peripheral/circulatory compartment was identified as a surrogate for the TME, and one containing putative biomarkers that may be predictive of early response to treatment. The patterns of failure in the nonresponders were uniformly determined using these circulatory markers, that is, indicators of circulating T cell activation, pre- and post-treatment, and the expansion of the circulatory TCR repertoire. It is important to note that lymphopenia, which has been commonly reported to be a consequence of conventionally fractionated radiation^18^, was not observed in the context of the hypofractionated SBRT used in this clinical trial. Finally, our metabolomic analysis revealed that circulating plasma fatty acids in circulatory plasma can act as a potential correlate of response. Exactly how these fatty acids affect T cell differentiation, functions and survival and are governed by lipid metabolism, however, requires further study^53,54^. Previous studies have shown a role for fatty acid metabolism^32,33^ and mitochondrial oxidative phosphorylation^55^ in dictating effector and memory T cell responses. Our data suggest that similar mechanisms could underlie responses to SBRT and warrant further investigation on the role of cell-intrinsic immunometabolic reprogramming in this context.

The positive response rate to the combination of radiation and immunotherapy observed in this trial suggests that this intervention represents a promising therapeutic strategy for treating locally advanced HPV-unrelated HNSCC, in marked contrast to the high morbidity and dismal outcomes of traditional treatment^1^. Nowadays, with clinical trials becoming increasingly complex, and with repeated biopsies simply not feasible, it is imperative to develop minimally invasive, high-throughput assays that can be used to predict response to treatment in the setting of radiation–immunotherapy. This is particularly relevant since in the context of radiation, standard imaging with CT, positron emission tomography (PET) or MRI fails to predict treatment response. In summary, we identified several potential circulatory correlates of response to the administered treatment, which can be assessed easily, and, if validated in larger trials, can serve to guide therapeutic management.

## Methods

### Ethics statement

The trial was carried out in accordance with Good Clinical Practice as required by applicable US laws and applications, including but not limited to the US Code of Federal Regulations (CFR) applicable to clinical studies (45 CFR Part 46, 21 CFR Part 50, 21 CFR Part 56, 21 CFR Part 312 and/or 21 CFR Part 812). Dr. Karam assures that no changes to the protocol took place without documented approval from the Institutional Review Board at the University of Colorado Anschutz Medical Campus. All personnel involved in the conduct of this study have completed Human Subjects Protection Training. Written, informed consent and HIPAA (Health Insurance Portability and Accountability Act) authorization were obtained from the patient before performing any protocol-related procedures, including screening evaluations. The authors affirm that human research participants provided written, informed consent for publication of the images in Fig. 1. The clinical trial protocol can be found as Supplementary Information.

### Participants

Enrolled patients were ≥18 yr in age, with a life expectancy ≥24 weeks, and diagnosed with intermediate and high-risk p16-negative, stages III and IV, nonmetastatic HNSCC cancer that was deemed resectable or borderline resectable by an Otolaryngology surgeon (NCT03635164). Patients were enrolled at three locations where the samples were collected (University of Colorado Hospital, Aurora, CO, USA, 80045; Memorial Hospital Central, Colorado Springs, CO, USA, 80909; Poudre Valley Hospital, Fort Collins, CO, USA, 80524). Twenty-one patients were enrolled. Seven patients were female, and 14 patients were male. The average age of a participant was 61 yr with a range from 43 yr to 84 yr. Patients were enrolled from November 2018 to May 2021. Diagnosis had to be confirmed either histologically or cytologically as stage III or IV HNSCC of oral cavity, hypopharynx, oropharynx or larynx. Stage II (T2 N0 M0) oral cavity cancer was also allowed. Patients also needed to have measurable disease defined as lesions that can be accurately measured in at least one dimension (longest diameter to be recorded) as >10 mm with CT scan or >10 mm with calipers by clinical exam. Other required factors for inclusion in the trial included: Eastern Cooperative Oncology Group (ECOG) performance status ≤1, body weight >30 kg, and adequate normal organ and marrow function. Adequate normal organ and marrow function was defined as hemoglobin ≥ 9.0 g dl^−1^, absolute neutrophil count ≥ 1.0 × 10^9^ per liter (≥1,000 per mm^3^), platelet count ≥ 75 × 10^9^ per liter (≥75,000 per mm^3^), serum bilirubin ≤ 1.5 × institutional upper limit of normal, and measured creatinine clearance > 40 ml min^−1^ or calculated creatinine clearance > 40 ml min^−1^ by the Cockcroft–Gault formula or by 24-h urine collection for determination of creatinine clearance. Patients were not compensated for participating in this trial.

### Trial design and treatment

This was a multi-center, prospective, single-arm phase I/Ib safety trial. Patients eligible for treatment had to be diagnosed with nonmetastatic, biopsy-proven p16-negative histology squamous cell carcinoma of the oral cavity, oropharynx, larynx or hypopharynx, and had to be eligible and amenable to surgical resection. This study enrolled using a 3 + 3 model. Patients received one dose of neoadjuvant durvalumab (1,500 mg) approximately 3–6 weeks before standard-of-care surgery given concurrently with the first dose of radiation (RT). The starting RT dose level was 6 Gy for two fractions (12 Gy total) every other day over approximately 1 week to sites of gross disease to minimize exposure to normal tissue. If toxicity developed and surgery was delayed by more than 6 weeks due to treatment toxicity (qualifying as a dose-limiting toxicity), the radiation dose was set to be dropped per protocol for the next set of patients. If this dose was tolerated, the dose was increased to 6 Gy for three fractions (18 Gy total) for the next three patients. Patients proceeded to surgical resection approximately 3–6 weeks after radiation as recommended by the Otolaryngology surgeon.

Post-operatively, pathology was reviewed at the multi-disciplinary head and neck conference, and the need for adjuvant therapy was discussed. For the first eight patients, all patients were given adjuvant therapy based on presenting features. However, after patient 8, adjuvant therapy was dictated based on high-risk pathologic features as per the National Comprehensive Cancer Network (NCCN) guidelines and treating physician recommendations. Adjuvant radiation included intensity-modulated radiation therapy of 60 Gy in 2 Gy once-daily fraction size (total of 30 fractions). If indicated, adjuvant systemic therapy included cisplatin or other cytotoxic chemotherapy or targeted biologics (cetuximab) per physician discretion.

All patients received adjuvant durvalumab to be initiated approximately 6–12 weeks post-surgery. It was given as 1,500 mg intravenously once every 4 weeks for a maximum of six doses, or until progression, toxicity or withdrawal from study. This was delivered either as monotherapy or concurrently with adjuvant radiation ± systemic therapy for high-risk patients. Safety and toxicity evaluations were done throughout the study process. DLTs and adjustment of radiation doses were done during the neoadjuvant period.

### Trial outcomes and assessment

Tumor response to neoadjuvant therapy (durvalumab + SBRT) was assessed by pathology review of the surgical specimen. Response was labeled as complete pathologic remission, microscopic residual tumor (only scattered foci of residual tumor cells) or macroscopic residual tumor by two independent board-certified pathologists blinded to treatment outcome. The method of assessment of disease status at baseline was MRI of neck with and without contrast and PET/CT of skull base to mid-thigh. The baseline assessment was performed no more than 28 d before SBRT. Efficacy for all patients was assessed by objective tumor assessment by repeat MRI of neck with and without contrast after the completion of radiation therapy pre-surgery. A PET/CT of skull base to mid-thigh and an MRI of the neck with and without contrast was done again after the last infusion of adjuvant durvalumab, and response assessment was categorized as having a response or progressive disease. Long-term follow-up was done with either PET/CT and/or MRI at the discretion of the treating physician. Following confirmed progression, patients continued to be followed up for survival every 12 weeks for 18 months. Patients who achieved and maintained disease control (that is, CR) through to the end of the treatment period continued with follow-up every 12 weeks for 18 months.

### Statistical analysis for clinical data

A total of 18 patients were included in the efficacy analyses. Sixteen patients treated at MTD were evaluable for MPR and included in the primary efficacy analysis of MPR. All 18 patients were included in the analysis of PFS and overall survival analyses. All data available, based on tissue availability, were included. Data from patients 03-001 and 03-002 were excluded from the translational analysis after it was determined that they did not receive the appropriate radiation therapy. There was no randomization as all patients were in the single arm of this clinical trial. Data collection and analysis were not performed blind to the conditions of the experiment. No statistical methods were used to pre-determine sample size but our sample sizes are similar to those reported in previous publications^26,35,38^.

Median and IQR were reported for continuous variables, and frequency and percentage for categorical variables. The associations between MPR and demographic and clinical characteristics were evaluated with nonparametric Wilcoxon rank sum test for continuous variables, and with Fisher’s exact test for categorical variables. The MPR was summarized with frequency and 95% exact CI. A one-sided exact test was conducted to test against the null hypothesis of 30% MPR. The Kaplan–Meier estimator of the survival probability curves along with the 95% CI was calculated and presented for the PFS and overall survival. The median survival time with the 95% CI was calculated and reported if feasible. The stratified Kaplan–Meier plot along with the *P* value from the log-rank test was presented.

The QOL data for each patient were collected from the FACT-H&N Version 4 Questionnaires for individual questions. Subscale scores and total scores were derived for each questionnaire using the FACT-H&N Scoring Guidelines (v.4), https://www.facit.org/measures-scoring-downloads/fact-hn-scoring-downloads. Summary statistics (median and IQR) were calculated and reported for the subscale scores and total scores for all patients by event time point. Linear mixed models with random patient effect were used to test if the QOL scores changed by event time point for each of the subscale scores and the total scores, and the Wald test results were reported. Multiple comparisons were adjusted using Benjamini and Hochberg’s method^56^. For more stable and robust testing results, the scores collected at SBRT Fraction 1 & Cycle 1, and at 60-week follow-up, were excluded from the linear mixed model analysis due to lack of sample.

All statistical analyses were performed by an independent statistician to ensure unbiased data review and were conducted in R v.4.1.0. *P* values <0.05 were considered statistically significant.

### RNA sequencing

Tumor tissue collected from patients at time of initial biopsy and at time of surgery was used for RNA sequencing. For the human RNA sequencing library preparation, a TempO-Seq Human Full Transcriptome FFPE Assay 96 Sample Kit was used (BioSpyder). A pathologist reviewed hematoxylin and eosin stains of the tumor samples, and areas of tumor cellularity were identified and marked. Only the pathologist-marked areas of tumor cellularity were scraped and processed per BioSpyder kit instructions. Samples were pooled and run in two sequencing lanes using a NextSeq high-throughput sequencing instrument at the Next Generation Sequencing Core at the University of Colorado Boulder. Reads were aligned and counts were generated using the BioSpyder TempoSeqr Platform. Genes with less than 1 mean raw count or less than 1 mean counts per million (CPM) were removed from the dataset. Reads were normalized to CPM using the edgeR R package^57^. Differential expression was calculated using the voom function in the limma R package^58^. Gene set enrichment analysis was performed using the fgsea^59^ R package (v.4.1.0 (build:27) for Mac, Broad Institute), with the full list of genes sorted by log_2_-transformed fold changes as the ranking metric. For Extended Data Fig. 3a,c, significance was established (colored red) by Benjamini Hochberg-adjusted *P* < 0.05 when fgsea was performed on all Hallmark pathways.

For EnrichmentMap analyses, differential expression between indicated groups was assessed for gene ontology (GO) terms for biological processes (GO BP). Gene sets were downloaded from baderlab.org/GeneSets on October 19, 2021 for human GO BP with sets containing electronically inferred annotation. GSEA software (GSEA v.4.1.0 (build:27) for Mac, Broad Institute) was used with the GSEAPreranked module for 1,000 permutations, enrichment statistic weighted, gene set size minimum 15 and otherwise default settings. Cytoscape^60^ (v.3.8.2 for Mac) was used with add-ins for EnrichmentMap^61^, downloaded using the EnrichmentMap Pipeline Collection. An EnrichmentMap was generated with false discovery rate *Q* value cutoff 0.05 and *P* value cutoff 0.05 using the Jaccard + Overlap combined similarity metric with cutoff 0.375. Clusters were manually labeled by visualizing the GO hierarchy with NaviGO (kiharalab.org/web/navigo/views/goparent.php). We used MultiPLIER to analyze our RNA sequencing data for cell type population level data. The code for MultiPLIER is publicly available at https://github.com/greenelab/multi-plier from Taroni and colleagues^27^. We used MultiPLIER to analyze our RNA sequencing data for cell type population level data.

### VECTRA imaging

The Human Immune Monitoring Shared Resource (HIMSR core) at the University of Colorado School of Medicine performed the immunostaining of patient tumor and DLN tissue using the Perkin Elmer Vectra 3 instrument. Slides were deparaffinized and treated with antigen retrieval buffer, blocked and incubated with primary antibody. This was followed by treatment with a horseradish peroxidase (HRP)-conjugated secondary antibody polymer, and HRP-reactive OPALfluores-cent reagents. To prevent further deposition of fluorescent dyes in subsequent staining steps, slides were stripped in between each stain with heat treatment in antigen retrieval buffer. DAPI was used to stain nucleated cells. Slides were scanned using the ×20 objective with a 0.5-mm resolution^12^. Color images were processed with inForm software v.2.4 and v.2.5. Quantification was done in Akoya Phenoptoreports in R v.4.1.0 and v.4.1.1, including cell percentages, cell densities, phenotyping and spatial analysis.

### TCR sequencing

TCR sequencing was performed on blood and tumor tissue pre- and post-treatment. Blood was collected in EDTA tubes and 0.5 ml was added to a SMART tube (SMART Tube, MTS1P) with 0.5 ml of IMDM medium and 2 μl of Protein Transport Inhibitor (Invitrogen). Samples were incubated for 6 h at 37 °C, then the fixatives in the tubes were released from the glass capsules and the tubes incubated for another 10 min at 37 °C. Smart tubes were then frozen at −80 °C until further processing. The Roche High Pure DNA Kit was used to extract DNA from the SMART tubes and tumor formalin-fixed paraffin-embedded (FFPE) for TCR sequencing. DNA concentration was determined using quantitative PCR. Samples were pooled and run in four cells on a MiSEQ sequencer according to Adaptive’s sequencing protocol. Analysis of TCR samples was done on Adaptive’s IMMUNOSEQ Analyzer.

### Mass cytometry (CyTOF)

Mass cytometry (CyTOF) of both patient blood and tumor samples was performed on the Helios Mass Cytometer at the University of Colorado Denver Cancer Center Flow Cytometry Core. Blood samples were processed on the day of blood collection. A CPT tube was collected from each patient at designated translational time points. The CPT tube was spun at 1,500*g* for 20 min and stopped without the brakes on. The buffy coat was collected, and 40 ml of PBS was added and spun down at 500*g* for 10 min in a 50-ml conical. The pellet was resuspended in 20 ml of PBS and spun down again at 500*g* for 10 min. No more than 5 million cells were frozen down in 90% FBS and 10% dimethylsulfoxide. Cells were placed at −80 °C overnight and then stored long-term in liquid nitrogen. Fresh tumor samples were collected from patients at time of biopsy and at time of surgery. Samples were minced and then placed in 5 ml of dissociation buffer (500 μl Collagenase type III (Worthington) with 10 μl of DNase (40 μg ml^−1^)). The samples were then incubated for 30 min at 37 °C and agitated every 10 min. The digestion buffer was then deactivated using 20 ml of HBSS (ThermoFisher). Tumors were then filtered through a 70-μm nylon filter into a 50-ml conical tube using FA3 buffer to wash the samples through. The samples were then centrifuged at 4 °C at 400*g* for 6 min and the supernatant was removed. Then, 2.5 ml of red cell lysis buffer (InVitrogen) was added and incubated at room temperature for 3 min. The lysis buffer was then deactivated by adding 30 ml of HBSS. The samples were then centrifuged again at 4 °C at 400*g* for 6 min, and the supernatant was removed. The samples were then resuspended in FA3 buffer and pipetted into a single-cell suspension. The sample was then run through a 40-μm nylon filter and washed with FA3 buffer. Live cells were then counted using a BD cell counter that detects tryphan blue. If there were more than 1 million cells, they were stimulated with (2 μl ml^−1^) Brefeldin A and (1 μl ml^−1^) monensin for 4 h. The samples were then spun down at 4 °C at 400*g* for 6 min and washed with FA3. Then, 250 μl of 1× lyse/fix buffer (BD pharmaceuticals) diluted in PBS was added and incubated at 37 °C for 30 min. The pellets were then washed twice with PBS at 4 °C at 400*g* for 6 min. The supernatant was then removed and the pellet was stored at −80 °C until further processing.

Blood samples were stimulated with (2 μl ml^−1^) Brefeldin A and (1 μl ml^−1^) monensin for 4 h, and then both the blood and tumors were processed according to the instructions provided with the Cell-ID 20-Plex Pd Barcoding Kit (Fluidigm). Samples were run the same day that they were stained. Samples were run in four batches altogether. To account for batch effects, all of the antibodies were pooled into a master mix, both extracellular and intracellular, and frozen at −80 °C in aliquots for each batch. All the batches were run within 2 weeks. Antibodies used: Anti-Human CD45 (HI30)-89Y, Anti-Human CD3 (UCHT1)-141Pr, Anti-Human CD19 (HIB19)-142Nd, Anti-Human CD127/IL-7Ra (A019D5)-143Nd, Anti-Human IL-2 (MQ1-17H12)-144Nd, Anti-Human CD4 (RPA-T4)-145Nd, Anti-Human CD8 (RPA-T8)-146Nd, Anti-Human CD11c (Bu15)-147Sm, Anti-Human CD16 (3G8)-148Nd, Anti-Human CD25 (2A3)-149Sm, Anti-Human CD86/B7.2 (IT2.2)-150Nd, Anti-Human CD103 (Ber-ACT8)-151Eu, Anti-Human cleaved Caspase 7 (D6H1)-152Sm, Anti-Human CD62L (DREG-56)-153Eu, Anti-Human TIM-3 (F38-2E2)-154Sm, Anti-Human CD27 (L128)-155Gd, Anti-Human CD14 (HCD14)-156Gd, Anti-Human CD134/OX40 (ACT35)-158Gd, Anti-Human FoxP3 (259D/C7)-159Tb, Anti-Human CD39 (A1)-160Gd, Anti-Human/Mouse Tbet (4B10)-161Dy, Anti-Human CD69 (FN50)-162Dy, Anti-Human TGFβ (TW4- 6H10)-163Dy, Anti-Human IL-17A (N49-653)-164Dy, Anti-Human IFN-γ (B27)-165Ho, Anti-Human IL-10 (JES3-9D7)-166Er, Anti-Human CD73 (AD2)-168Er, Anti-Human CD159a/NKG2A (Z199)-169Tm, Anti-Human CD45RA (HI100)-170Er, Anti-Human CD226 (DX11)-171Yb, Anti-Human Ki-67 (B56)-172Yb, Anti-Human HLA-DR (L243)-173Yb, Anti-Human CD279/PD-1 (EH12.2H7)-174Yb, Anti-Human TNF-α (Mab11)-175Lu, Anti-Human CD56 (HCD56)-176Yb and Anti-Human TIGIT (MBSA43)-209Bi. Three antibodies were purchased and then conjugated to metals through the HIMSR core using Fluidigm conjugation kits: ephrinB2 (R&D Systems (Arg27-ALA227))-139, EphA4 (ThermoFisher (21875-1-AP))-115 and TCF1 (Biolegend (TCF6))-167Er. All antibodies were purchased from Fluidigm, validated by Fluidigm and used at the recommended concentration unless otherwise noted. Human FC block used: Human BD Fc Block (BD Pharmingen). Analysis of CyTOF data was done in FlowJo (v.10.7.1), Astrolabediagnositics.com and cytobank.com.

### Metabolomics

Metabolomics analyses were performed as extensively described in previous studies^62^. A volume of 20 μl of frozen plasma was extracted in 480 μl of methanol:acetonitrile:water (5:3:2, v/v/v)^63^. After vortexing at 4 °C for 30 min, extracts were separated from the protein pellet by centrifugation for 10 min at 10,000*g* at 4 °C and stored at −80 °C until analysis. Ultra-high-pressure liquid chromatography–mass spectrometry (UHPLC) analyses were performed using a Vanquish UHPLC system coupled online to a Q Exactive mass spectrometer (ThermoFisher)^64^. Samples were analyzed using a 5-min gradient as described^64,65^. Solvents were supplemented with 0.1% formic acid for positive-mode runs and 1 mM ammonium acetate for negative-mode runs. Mass spectrometry data acquisition, data analysis and elaboration were performed as described.

### Random forest prediction model

A random forest model was trained on the 16 patients from the trial who met the training criteria. We chose to use a random forest model because we are training on complex variables that most likely have nonlinear interactions, based to how others have previously trained on similar datasets^66^. We included all evaluable patients that received 18 Gy or 24 Gy MTD. Due to the limited amount of data, we repeated our entire experimental pipeline, which used nested cross-validation, 20 times with different random initial seedings to ensure that our results were repeatable. We implemented the following steps in our experimental pipeline. First, we split the data into training and validation sets, 75% and 25%, respectively, using stratified sampling of responders and nonresponders in equal proportion to their makeup in the overall dataset. k-nearest neighbor (KNN) imputation, which was selected for its effectiveness in similar datasets, was used to account for the missing data^67,68^. Second, we upsampled the nonresponders to balance their count relative to the responders. Third, we ran threefold cross-validation over the training dataset to select optimal hyper-parameters for the training data using a grid search over the parameter space. The parameter space we searched across included the number of estimators, maximum features per tree, depth of the trees and the requirements for splitting nodes of the individual decision trees. Finally, we evaluated the best model found via cross-validation on our validation set, which gave us our expected accuracy. We averaged performance over the 20 pipeline trials to arrive at our final accuracy and bounds. All experiments were performed with Python 3.6.3 and scikit-learn v.0.24.2 (ref. ^69^). The code for running and evaluating the model and data is available at https://github.com/adumit/phase-1-hnscc-trial-prediction.

### Statistical analysis for translational data

Statistical analysis, unless otherwise stated, was done in GraphPad (v.9.1.0). For paired analysis, a two-tailed paired *t*-test assuming Gaussian distribution was used. For nonpaired analysis, a two-tailed Student’s *t*-test was used. For the multi-variate analysis, a multiple linear regression analysis was performed. For the metabolomics analysis, graphs and statistical analyses (unpaired *t*-test) were prepared with GraphPad Prism 8.0 (GraphPad Software). Heat maps, hierarchical clustering analyses, partial least squares discriminant analyses and two-way analysis of variance were calculated and plotted with MetaboAnalyst 5.0 (ref. ^70^). All data points represent distinct samples, not repeated sampling. For continuous variables, data distribution was assumed to be normal but this was not formally tested.

### Figures

Figures 1a and 6a and Extended Data Fig. 8a,b were created using BioRender.com.

### Reporting summary

Further information on research design is available in the Nature Research Reporting Summary linked to this article.

### Supplementary information

Reporting Summary

Clinical trial protocol

### Source data

Source Data Fig. 1 Statistical source data.

Source Data Fig. 2 Statistical source data.

Source Data Fig. 3 Statistical source data.

Source Data Fig. 4 Statistical source data.

Source Data Fig. 5 Statistical source data.

Source Data Extended Data Fig. 2 Statistical source data.

Source Data Extended Data Fig. 3 Statistical source data.

Source Data Extended Data Fig. 4 Statistical source data.

Source Data Extended Data Fig. 5 Statistical source data.

Source Data Extended Data Fig. 6 Statistical source data.

Source Data Extended Data Fig. 7 Statistical source data.

Source Data Extended Data Fig. 8 Statistical source data.

## Data Availability

The clinical trial protocol is available online at clinicaltrials.gov with the following clinical trial number: NCT03635164. The metabolomics data are available at Metabolomics Workbench under the project ID PR001336 and project 10.21228/M81D70. The RNA sequencing data are available through the GEO accession number GSE210287. TCR sequencing data are publicly available on the Adaptive Biotechnologies immuneACCESS database and can be analyzed using their immunoSEQ Analyzer using 10.21417/LBD2022NC or the URL adaptivebiotech.com/pub/darragh-2022-nc. Mass cytometry data (CyTOF) data will be available upon reasonable request. Further information on research design is available in the Nature Research Reporting Summary linked to this article. Source data are provided with this paper.

## Extended data

**Extended Data Table 1:** Incidence of Treatment Related Adverse Events

AE term = >3	% who experienced the AE in all patients	% who experienced the AE in patients received 24 Gy	% who experienced the AE in patients received 18 Gy	% of the AEs related to SBRT	% of the AEs related to Durva	% of the AEs related to Combo
Mucositis oral	19%	11.1%	11.1%	100%	0%	16.7%
Dysphagia	9.5%	11.1%	0%	100%	0%	0%
Aphonia	4.8%	0%	0%	100%	0%	0%
Infections and infestations - Other	9.5%	0%	11.1%	100%	0%	0%
Weight loss	9.5%	0%	11.1%	100%	0%	0%
Dry mouth	4.8%	0%	11.1%	100%	0%	0%
Edema limbs	4.8%	0%	11.1%	0%	100%	0%
Wound infection	9.5%	11.1%	11.1%	100%	0%	0%
Wound dehiscence	4.8%	0%	11.1%	100%	0%	0%
Oral cavity fistula	4.8%	0%	11.1%	100%	0%	0%
Respiratory, thoracic and mediastinal disorders - Other	4.8%	0%	11.1%	100%	0%	100%
AE term <3						
Arthralgia	4.8%	0%	11.1%	0%	100%	0%
Dysgeusia	52.4%	44.4%	44.4%	92.9%	7.1%	14.3%
Mucositis oral	66.7%	77.8%	44.4%	91.3%	8.7%	26.1%
Dry mouth	28.6%	22.2%	22.2%	100%	0%	9.1%
Dysphagia	23.8%	44.4%	0%	100%	16.7%	0%
Dermatitis radiation	42.9%	33.3%	33.3%	100%	0%	33.3%
Fatigue	57.1%	66.7%	33.3%	84.6%	69.2%	46.2%
Weight loss	19%	11.1%	11.1%	100%	0%	0%
Edema face	14.3%	0%	11.1%	100%	0%	0%
Lymphedema	14.3%	11.1%	0%	50%	50%	50%
General disorders and administration site conditions - Other	28.6%	33.3%	22.2%	100%	0%	0%
Cough	14.3%	11.1%	11.1%	100%	0%	0%
Sore throat	14.3%	11.1%	11.1%	100%	0%	0%
Gastrointestinal disorders - Other	33.3%	33.3%	33.3%	92.9%	7.1%	7.1%
Nausea	19%	11.1%	33.3%	50%	50%	0%
Musculoskeletal and connective tissue disorder - Other	19%	22.2%	22.2%	75%	25%	25%
Trismus	14.3%	11.1%	22.2%	66.7%	33.3%	33.3%
Hypothyroidism	23.8%	44.4%	11.1%	14.3%	71.4%	42.9%

**Extended Data Table 2:** Quality of Life Assessment

Event		SBRT Fraction 1 & Cycle 1	Pre-op Clinical Assess.	Cycle 2	Cycle 3	Cycle 4	Cycle 5	Cycle 6	Cycle 7	EOT	12 Week Follow-up	24 Week Follow-up	36 Week Follow-up	48 Week Follow-up	60 Week Follow-up	72 Week Follow-up	Total	Adjusted *P* values
Total N		1	14	17	17	15	14	15	13	4	8	8	6	3	2	5	142	
PHYSICAL WELL-BEING	Median (IQR)	24.0 (24.0 to 24.0)	23.0 (20.2 to 25.5)	22.0 (16.0 to 27.0)	21.0 (15.0 to 25.0)	21.0 (17.0 to 25.5)	20.0 (19.0 to 22.8)	20.0 (16.0 to 25.5)	25.0 (21.0 to 26.0)	21.0 (18.2 to 24.0)	25.5 (22.0 to 27.2)	23.0 (21.0 to 27.0)	22.0 (18.2 to 22.8)	18.0 (17.5 to 22.0)	26.5 (26.2 to 26.8)	19.0 (18.0 to 27.0)	22.0 (18.0 to 26.0)	0.002*
SOCIAL/FAMILY WELL-BEING	Median (IQR)	27.0 (27.0 to 27.0)	25.6 (18.8 to 27.8)	24.5 (16.0 to 26.0)	22.2 (20.0 to 27.0)	24.0 (18.5 to 27.4)	22.7 (19.0 to 28.0)	25.0 (19.0 to 28.0)	24.0 (19.0 to 28.0)	24.5 (20.8 to 25.8)	25.5 (21.5 to 28.0)	25.5 (14.8 to 27.2)	25.7 (11.4 to 27.7)	21.0 (10.5 to 24.5)	5.5 (2.8 to 8.2)	17.5 (9.0 to 28.0)	24.0 (17.1 to 28.0)	0.999
EMOTIONAL WELL-BEING	Median (IQR)	21.0 (21.0 to 21.0)	19.0 (14.5 to 20.0)	20.0 (16.0 to 23.0)	20.0 (18.0 to 23.0)	19.0 (17.0 to 23.0)	19.5 (16.5 to 23.8)	20.0 (18.0 to 24.0)	20.0 (14.0 to 22.0)	19.5 (16.8 to 20.0)	21.0 (18.8 to 23.2)	20.5 (19.2 to 22.0)	21.0 (20.0 to 22.8)	19.0 (18.0 to 19.5)	14.5 (10.8 to 18.2)	18.0 (15.0 to 20.0)	20.0 (17.0 to 22.8)	0.38
FUNCTIONAL WELL-BEING	Median (IQR)	20.0 (20.0 to 20.0)	18.5 (13.2 to 20.8)	16.0 (12.0 to 19.0)	13.0 (9.0 to 19.0)	16.0 (12.5 to 19.5)	15.0 (12.0 to 17.8)	16.0 (12.0 to 24.0)	17.0 (13.0 to 24.0)	15.5 (11.8 to 19.5)	19.5 (11.0 to 23.0)	19.0 (14.8 to 20.8)	12.5 (10.2 to 15.5)	12.0 (9.0 to 12.5)	4.2 (3.9 to 4.6)	13.0 (6.0 to 14.0)	15.5 (11.0 to 20.0)	0.66
HEAD & NECK CANCER SUBSCALE	Median (IQR)	37.0 (37.0 to 37.0)	24.5 (20.2 to 31.8)	22.0 (15.0 to 30.0)	19.0 (13.0 to 23.0)	22.0 (19.0 to 27.5)	20.0 (15.5 to 24.8)	21.0 (18.4 to 29.5)	23.0 (17.0 to 33.3)	17.0 (10.2 to 23.8)	25.0 (21.5 to 30.8)	19.5 (17.2 to 26.5)	21.9 (15.7 to 27.5)	23.0 (17.0 to 26.0)	18.0 (15.0 to 21.0)	23.0 (14.0 to 25.0)	22.0 (15.0 to 28.7)	0.537
FACT-H&N Trial Outcome Index (TOI)	Median (IQR)	81.0 (81.0 to 81.0)	63.0 (58.0 to 71.1)	55.2 (48.2 to 72.2)	50.0 (42.0 to 67.9)	61.0 (49.5 to 69.0)	54.5 (45.0 to 62.0)	57.0 (43.5 to 75.5)	61.0 (56.0 to 79.0)	50.0 (42.8 to 61.2)	60.0 (58.0 to 74.8)	58.0 (57.0 to 68.5)	54.9 (49.7 to 61.0)	55.0 (48.0 to 57.0)	48.8 (46.4 to 51.1)	55.0 (45.0 to 58.0)	58.0 (47.2 to 71.3)	0.201
FACT-G total score	Median (IQR)	92.0 (92.0 to 92.0)	84.5 (70.2 to 91.8)	78.0 (70.0 to 82.0)	77.0 (65.0 to 82.0)	77.0 (70.6 to 90.9)	74.0 (68.7 to 88.2)	76.7 (65.5 to 96.5)	83.0 (72.0 to 98.0)	77.0 (68.5 to 84.7)	87.0 (71.2 to 95.5)	87.5 (75.0 to 90.8)	79.2 (59.0 to 82.6)	68.0 (60.0 to 72.5)	50.8 (50.4 to 51.1)	63.5 (52.0 to 77.0)	77.8 (65.2 to 92.0)	0.631
FACT-H&N total score	Median (IQR)	129.0 (129.0 to 129.0)	109.7 (94.0 to 116.0)	100.0 (83.5 to 115.0)	96.0 (83.0 to 112.0)	98.0 (89.1 to 116.4)	95.2 (84.0 to 111.0)	101.7 (77.7 to 122.5)	108.0 (87.0 to 124.0)	94.0 (80.2 to 107.0)	107.7 (99.2 to 119.2)	104.5 (95.8 to 112.0)	98.6 (86.8 to 103.4)	79.0 (77.0 to 92.5)	68.8 (65.4 to 72.1)	77.5 (75.0 to 104.0)	101.0 (80.5 to 115.8)	0.537

^*^Linear mixed model with random patients’ effect were used to test if the QOL scores changed by event time point for each of the sub scale scores and the total scores, and the Wald test results were reported. Multiple comparisons were adjusted using Benjamini and Hochberg’s method.

## Code availability

The code for running and evaluating the model and data is available at https://github.com/adumit/phase-1-hnscc-trial-prediction.
