# Author Correction: A phase I/Ib trial and biological correlate analysis of neoadjuvant SBRT with single-dose durvalumab in HPV-unrelated locally advanced HNSCC

**DOI:** 10.1038/s43018-022-00507-6

**Published:** 2022-12-28

**Authors:** Laurel B. Darragh, Michael M. Knitz, Junxiao Hu, Eric T. Clambey, Jennifer Backus, Andrew Dumit, Von Samedi, Andrew Bubak, Casey Greene, Timothy Waxweiler, Sanjana Mehrotra, Shilpa Bhatia, Jacob Gadwa, Thomas Bickett, Miles Piper, Kareem Fakhoury, Arthur Liu, Joshua Petit, Daniel Bowles, Ashesh Thaker, Kimberly Atiyeh, Julie Goddard, Robert Hoyer, Adrie Van Bokhoven, Kimberly Jordan, Antonio Jimeno, Angelo D’Alessandro, David Raben, Jessica D. McDermott, Sana D. Karam

**Affiliations:** 1 Radiation Oncology, University of Colorado Denver at Anschutz Medical Campus, Aurora, CO USA; 2 Department of Immunology, University of Colorado Denver at Anschutz Medical Campus, Aurora, CO USA; 3 Department of Biostatistics and Informatics, Colorado School of Public Health, University of Colorado Denver, Aurora, CO USA; 4 Department of Anesthesiology, University of Colorado Anschutz Medical Campus, Aurora, CO USA; 5 Department of Pathology, University of Colorado Denver at Anschutz Medical Campus, Aurora, CO USA; 6 Department of Neurology, University of Colorado Denver at Anschutz Medical Campus, Aurora, CO USA; 7 Department of Biochemistry and Molecular Genetics, University of Colorado Denver at Anschutz Medical Campus, Aurora, CO USA; 8 Department of Radiation Oncology, University of Colorado, Poudre Valley Hospital, Fort Collins, CO USA; 9 Division of Medical Oncology, University of Colorado School of Medicine, Aurora, CO USA; 10 Department of Radiology, University of Colorado Denver at Anschutz Medical Campus, Aurora, CO USA; 11 Department of Otolaryngology Head and Neck Surgery, University of Colorado, Memorial South Hospital, Colorado Springs, CO USA; 12 Department of Otolaryngology Head and Neck Surgery, University of Colorado Denver at Anschutz Medical Campus, Aurora, CO USA

**Keywords:** Oral cancer, Tumour immunology, Cancer, Immunotherapy

Correction to: *Nature Cancer* 10.1038/s43018-022-00450-6, published online 25 November 2022.

In the version of this article initially published, the Competing interests section was missing the disclosure “A.J. has research support from NCI R01CA149456, R01DE024371, and P50CA261605; and stock/options ownership in Suvica and Champions Oncology. A.J.’s institution has contracts with Cantargia, DebioPharm, Genentech, Iovance, Khar Biopharma, Merck, Moderna, Pfizer, Sanofi, and SQZ for trials where A.J. is the local P.I.” The error has been corrected in the HTML and PDF versions of the article.

