# Supplementary material for: A phase I/Ib trial and biological correlate analysis of neoadjuvant SBRT with single-dose durvalumab in HPV-unrelated locally advanced HNSCC
Source: Nat Cancer. 2022 Nov 25;3(11):1300–17. doi: 10.1038/s43018-022-00450-6 (PMC9701140; doi:10.1038/s43018-022-00450-6)
Supplement: Supplementary file 1 — Reporting Summary [file 43018_2022_450_MOESM1_ESM.pdf]

nature portfolio

Corresponding author(s): NATCANCER-A06133

Last updated by author(s): Sep 16, 2022

Reporting Summary

Nature Portfolio wishes to improve the reproducibility of the work that we publish. This form provides structure for consistency and transparency in reporting. For further information on Nature Portfolio policies, see our Editorial Policies and the Editorial Policy Checklist.

Statistics

For all statistical analyses, confirm that the following items are present in the figure legend, table legend, main text, or Methods section.

n/a	Confirmed	
☐	☒	The exact sample size (*n*) for each experimental group/condition, given as a discrete number and unit of measurement
☐	☒	A statement on whether measurements were taken from distinct samples or whether the same sample was measured repeatedly
☐	☒	The statistical test(s) used AND whether they are one- or two-sided<br>*Only common tests should be described solely by name; describe more complex techniques in the Methods section.*
☐	☒	A description of all covariates tested
☐	☒	A description of any assumptions or corrections, such as tests of normality and adjustment for multiple comparisons
☐	☒	A full description of the statistical parameters including central tendency (e.g. means) or other basic estimates (e.g. regression coefficient) AND variation (e.g. standard deviation) or associated estimates of uncertainty (e.g. confidence intervals)
☐	☒	For null hypothesis testing, the test statistic (e.g. *F*, *t*, *r*) with confidence intervals, effect sizes, degrees of freedom and *P* value noted<br>*Give P values as exact values whenever suitable.*
☒	☐	For Bayesian analysis, information on the choice of priors and Markov chain Monte Carlo settings
☒	☐	For hierarchical and complex designs, identification of the appropriate level for tests and full reporting of outcomes
☒	☐	Estimates of effect sizes (e.g. Cohen's *d*, Pearson's *r*), indicating how they were calculated

Our web collection on statistics for biologists contains articles on many of the points above.

Software and code

Policy information about availability of computer code

Data collection	No software was used to collect the data.
Data analysis	Prism (v. 8.0) was used for metabolomics analysis. Prism (v. 9.1.0) was used for all other analysis except for the following: Python 3.6.3 and scikit-learn version 0.24.2 was used to perform the random forest model, we used code provided publicly at https://github.com/greenelab/multi-plier for our RNA sequencing analysis with MultiPLIER, Flowjo (v. 10.7.1), Astrolabe Diagnostics, and Cytobank were used to analyze our CyTOF data, for VECTRA image analysis we used the InForm software (v. 2.4 and v. 2.5) with Akoya Phenoptoreports in R version 4.1.0 and 4.1.1, for RNA pathway analysis GSEA (v. 4.1.0), and the data and code for the random forest model is available at https://github.com/adumit/phase-1-hnscc-trial-prediction.

For manuscripts utilizing custom algorithms or software that are central to the research but not yet described in published literature, software must be made available to editors and reviewers. We strongly encourage code deposition in a community repository (e.g. GitHub). See the Nature Portfolio guidelines for submitting code & software for further information.

Data

Policy information about availability of data

All manuscripts must include a data availability statement. This statement should provide the following information, where applicable:

- Accession codes, unique identifiers, or web links for publicly available datasets
- A description of any restrictions on data availability
- For clinical datasets or third party data, please ensure that the statement adheres to our policy

The clinical trial protocol is available online at clinicaltrials.gov with the following clinical trial number: NCT03635164. The metabolomics data is available at Metabolomics Workbench under the project ID PR001336 and project DOI: 10.21228/M81D70. The RNA sequencing data is available through the GEO accession

number GSE210287. TCR sequencing data is publicly available on Adaptive Biotechnologies immuneACCESS database and can be analyzed using their immunoSEQ Analyzer. Mass cytometry data (CyTOF) data will be available upon reasonable request.

Code Availability

The code for running and evaluating the model and data is available at https://github.com/adumit/phase-1-hnscc-trial-prediction.

Field-specific reporting

Please select the one below that is the best fit for your research. If you are not sure, read the appropriate sections before making your selection.

☒ Life sciences ☐ Behavioural & social sciences ☐ Ecological, evolutionary & environmental sciences

For a reference copy of the document with all sections, see nature.com/documents/nr-reporting-summary-flat.pdf

Life sciences study design

All studies must disclose on these points even when the disclosure is negative.

Sample size

Part 1: Dose Escalation
The expectation is that 9 patients will be enrolled to the trial during part 1. This is based on the expectation that all dose levels are safe (i.e. patients will not experience DLTs at all dose levels). In this case, 3 patients will be enrolled at dose level 2 (0/3 DLTs), then dose escalating to dose level 3 and enrolling a total of 6 patients at this dose level, for a total of 9 patients. The range of patients needed will be 6–12 patients.
Part 2: Dose Expansion
The primary outcome of this study is to assess safety. An additional 8 patients will be enrolled at the DLT dosing determined during part 1 for further safety assessment. This number was chosen to answer our secondary outcome of efficacy as measured by pathologic response and cytotoxic CD8 T cell infiltration. This is determined by the binary measure of whether or not a patient has relevant CD8 infiltration, defined as a 1.5-fold increase at tumor resection as compared to biopsy. Let p represent the proportion of patients with relevant CD8 infiltration. It is expected that half (50%) of patients not receiving this treatment will have relevant CD8 infiltration. The objective is to test the following hypotheses:
H0: $p \leq 0.50$
H1: $p \geq 0.80$
The hypotheses will be tested using the exact binomial test. A total of 14 patients are needed to have at least 80% power (87.0% to be exact) to discriminate between the hypotheses stated above when testing them using a 1-sided superiority test and controlling the type 1 error rate at 0.10.
Total Sample Size
During part 1, 6 patients will be treated at the MTD. The CD8 infiltration will be assessed on these 6, leaving an additional subjects to be enrolled during part 2 in order to achieve the total sample size of 14 needed for part 2. The total sample size is thus expected to be 17 patients (9 from part 1 + 8 from part 2) as the total sample size will depend on the number of patients needed during part 1 of the trial. The total sample size would be up to 20.

Data exclusions

A few patients were excluded from the evaluation of efficacy as they were treated with the lowest dose of radiation (12 Gy). Two other patients were excluded from translational and efficacy studies as the dose of radiation was not given to the entirety of the primary tumor (03-001 and 03-002).

Replication

Experiments were not replicated as this was a clinical trial. Phase II will be conducted and will seek to replicate these results.

Randomization

There was no randomization. There was only one treatment arm in this trial.

Blinding

Investigators were not blinded to the patients group since there was only one arm to this clinical trial.

Reporting for specific materials, systems and methods

We require information from authors about some types of materials, experimental systems and methods used in many studies. Here, indicate whether each material, system or method listed is relevant to your study. If you are not sure if a list item applies to your research, read the appropriate section before selecting a response.

Materials & experimental systems

n/a	Involved in the study
☐	☒ Antibodies
☒	☐ Eukaryotic cell lines
☒	☐ Palaeontology and archaeology
☒	☐ Animals and other organisms
☐	☒ Human research participants
☐	☒ Clinical data
☒	☐ Dual use research of concern

Methods

n/a	Involved in the study
☒	☐ ChIP-seq
☐	☒ Flow cytometry
☒	☐ MRI-based neuroimaging

Antibodies

Antibodies used

Anti-Human CD45 (HI30)-89Y, Anti-Human CD3 (UCHT1)-141Pr, Anti-Human CD19 (HIB19)-142Nd, Anti-Human CD127/ IL-7Ra (A019D5)-143Nd, Anti-Human IL2 (MQ1-17H12)-144Nd, Anti-Human CD4 (RPA-T4)-145Nd, Anti-Human CD8 (RPA-T8)-146Nd, Anti-Human CD11c (Bu15)-147Sm, Anti-Human CD16 (3G8)-148Nd, Anti-Human CD25 (2A3)-149Sm, Anti-Human CD86/ B7.2 (IT2.2)-150Nd, Anti-Human CD103 (Ber-ACT8)-151Eu, Anti-Human cleaved Caspase 7 (D6H1)-152Sm, Anti-Human CD62L (DREG-56)-153Eu, Anti-Human TIM-3 (F38-2E2)-154Sm, Anti-Human CD27 (L128)-155Gd, Anti-Human CD14 (HCD14)-156Gd, Anti-Human CD134/ OX40 (ACT35)-158Gd, Anti-Human FoxP3 (259D/C7)-159Tb, Anti-Human CD39 (A1)-160Gd, Anti-Human/Mouse Tbet (4B10)-161Dy, Anti-Human CD69 (FN50)-162Dy, Anti-Human TGFbeta (TW4- 6H10)-163Dy, Anti-Human IL-17A (N49-653)-164Dy, Anti-Human IFNg (B27)-165Ho, Anti-Human IL-10 (JES3-9D7)-166Er, Anti-Human CD73 (AD2)-168Er, Anti-Human CD159a/NKG2A (Z199)-169Tm, Anti-Human CD45RA (HI100)-170Er, Anti-Human CD226 (DX11)-171Yb, Anti-Human Ki-67 (B56)-172Yb, Anti-Human HLADR (L243)-173Yb, Anti-Human CD279/ PD-1 (EH12.2H7)-174Yb, Anti-Human TNFa (Mab11)-175Lu, Anti-Human CD56 (HCD56)-176Yb, and Anti-Human TIGIT (MBSA43)-209Bi. Three antibodies were purchased and then conjugated to metals through the HIMSR core using Fluidigm conjugation kits: ephrinB2 (R&D Systems (Arg27-ALA227))-139, EphA4 (ThermoFisher (21875-1-AP))-115, and TCF1 (Biolegend (TCF6))-167Er. Human FC block used: Human BD Fc Block (BD Pharmagen).

Validation

All mass cytometry antibodies used in this study were used based on the dilutions recommended by the manufacturer (Fluidigm). All CyTOF antibodies were validated by Fluidigm. Each lot of conjugated antibody is quality control tested by CyTOF (R) analysis of stained cells using the appropiate positive and negative cell staining and/or activation controls. All VECTRA antibodies were optimized by the HIMSR core at the University of Colorado Anschutz Medical Campus.

Human research participants

Policy information about studies involving human research participants

Population characteristics

Patients eligible for treatment had to be diagnosed with non-metastatic, biopsy-proven p16-negative histology squamous cell carcinoma of the oral cavity, oropharynx, larynx, or hypopharynx, and had to be eligible and amenable to surgical resection. Patients 18 years or older were allowed to participate in the study. Both females and males were recruited for the study, but sex was not specifically recruited for. All patients were treated with SBRT and durvalumab (1500mg). The dose of radiation was escalated from 12Gy to 18Gy, with an expansion cohort getting dosimetry painting of the tumor at 24Gy as reported.

Recruitment

Patients were screened for eligibility at three different locations in Colorado. All patients that qualified were recruited from The University of Colorado Hospital, Aurora, Colorado, United States, 80045; Memorial Hospital Central, Colorado Springs, Colorado, United States, 80909; Poudre Valley Hospital, Fort Collins, Colorado, United States, 80524. Patients were enrolled from November 2018 to May 2021. No known bias for recruitment was identified.

Ethics oversight

Institutional Review Board (IRB) at the University of Colorado Anschutz Medical Campus.

Note that full information on the approval of the study protocol must also be provided in the manuscript.

Clinical data

Policy information about clinical studies

All manuscripts should comply with the ICMJE guidelines for publication of clinical research and a completed CONSORT checklist must be included with all submissions.

Clinical trial registration

NCT03635164

Study protocol

Study protocol will be provided to the journal for review by referees.

Data collection

Patient samples were collected at three different locations. atients were recruited from three locations in Colorado; University of Colorado Hospital, Aurora, Colorado, United States, 80045; Memorial Hospital Central, Colorado Springs, Colorado, United States, 80909; Poudre Valley Hospital, Fort Collins, Colorado, United States, 80524. Patients were enrolled from November 2018 to May 2021. Patients not at the Anschutz Medical Campus had samples overnighted or delivered via currier for processing.

Outcomes

Primary Endpoint
All patients who received surgery, radiation therapy and at least 1 dose of durvalumab and for whom any valid post-baseline safety data were available were included in the safety analysis set. When assessing safety and tolerability, summaries were produced based on the safety analysis set. Patients were analyzed according to the actual treatment received.

Each subject was observed for the occurrence of a DLT from the day on which the patient starts neoadjuvant durvalumab at 1500 mg up until the day of surgery. The duration of this observation period was approximately 3–6 weeks. The primary endpoint of the safety lead-in was to determine the safety and tolerability. Toxicity was assessed by CTCAE v. 4.03 criteria. Data from all cycles of treatment was combined in the presentation of safety data. AEs are listed individually by patient.

Secondary Endpoint
Overall survival (OS) will be determined from the time of enrollment to date of death due to any cause. OS was evaluated by Kaplan-Meier estimate. Summaries of the number and percentage of patients who have died, are still in survival follow-up, are lost to follow-up, are at the end of study, and have withdrawn consent was provided along with median OS.

Tumor response to neoadjuvant therapy (durvalumab + SBRT) was assessed by pathology review of the surgical specimen. Response was labeled as complete pathologic remission, microscopic residual tumor (only scattered foci of residual tumor cells) or macroscopic residual tumor. The method of assessment of disease status at baseline was MRI neck with and without contrast and PET-CT skull base to mid-thigh. The baseline assessment was performed

Flow Cytometry

Plots

Confirm that:

☒ The axis labels state the marker and fluorochrome used (e.g. CD4-FITC).

☒ The axis scales are clearly visible. Include numbers along axes only for bottom left plot of group (a 'group' is an analysis of identical markers).

☒ All plots are contour plots with outliers or pseudocolor plots.

☒ A numerical value for number of cells or percentage (with statistics) is provided.

Methodology

Sample preparation

Mass cytometry (CyTOF) of both patient blood and tumor samples was performed on the Helios Mass Cytometer at the University of Colorado Denver Cancer Center Flow Cytometry Core. Blood samples were processed on the day of blood collection. A CPT tube was collected from each patient at designated translational timepoints. The CPT tube was spun at 1500g for 20 minutes and stopped without the brakes on. The buffy coat was collected, and 40mL of PBS was added and spun down at 500g for 10 minutes in a 50mL conical. The pellet was resuspended in 20mL of PBS and spun down again at 500g for 10 minutes. No more than 5 million cells were frozen down in 90% FBS and 10% DMSO. Cells were placed at -80C overnight and then stored long-term in liquid nitrogen. Fresh tumors samples were collected from patients at time of biopsy and at time of surgery. Samples were minced and then placed in 5mL of dissociation buffer (500ul Collagenase type III (Worthington, Lakewood, New Jersey, USA) with 10ul of DNase (40ug/mL)). The samples were then incubated for 30 minutes at 30C and agitated every 10 minutes. The digestion buffer was then deactivated using 20mL of HBSS (ThermoFisher, Waltham, Massachusetts, USA). Tumors were then filtered through a 70um nylon filter into a 50mL conical tube using FA3 buffer to wash the samples through. The samples were then centrifuged at 4C at 400g for 6 minutes and the supernatant was removed. 2.5mL of red cell lysis buffer (InVitrogen, Carlsbad, California, USA) was then added and incubated at room temperature for 3 minutes. The lysis buffer was then deactivated by adding 30mL of HBSS. The samples were then centrifuged again at 4C at 400g for 6 minutes, and the supernatant was removed. The samples were then resuspended in FA3 buffer and pipetted into a single cell suspension. The sample was then run through a 40um nylon filter and washed with FA3 buffer. Live cells were then counted using a BD cell counter that detects tryphan blue. If there were more than 1 million cells, they were stimulated with (2ul/mL) Brefeldin A and (1ul/mL) monensin for 4 hours. The samples were then spun down at 4C at 400g for 6 minutes and washed with FA3. 250ul of 1X lyse/fix buffer (BD pharmaceuticals) diluted in PBS was added and then incubated at 37C for 30 minutes. The pellets were then washed twice with PBS at 4C at 400g for 6 minutes. The supernatant was then removed and the pellet was stored at -80C until further processing.

Blood samples were stimulated with (2ul/mL) Brefeldin A and (1ul/mL) monensin for 4 hours, and then both the blood and tumors were processed according to the instructions provided with the Cell-ID™ 20-Plex Pd Barcoding Kit (Fluidigm). Samples were run the same day that they were stained. Samples were run in 4 batches altogether. To account for batch effects, all of the antibodies were pooled into a master mix, both extracellular and intracellular, and frozen at -80C in aliquots for each batch. All the batches were run within two weeks. Antibodies used: Anti-Human CD45 (HI30)-89Y, Anti-Human CD3 (UCHT1)-141Pr, Anti-Human CD19 (HIB19)-142Nd, Anti-Human CD127/ IL-7Ra (A019D5)-143Nd, Anti-Human IL2 (MQ1-17H12)-144Nd, Anti-Human CD4 (RPA-T4)-145Nd, Anti-Human CD8 (RPA-T8)-146Nd, Anti-Human CD11c (Bu15)-147Sm, Anti-Human CD16 (3G8)-148Nd, Anti-Human CD25 (2A3)-149Sm, Anti-Human CD86/ B7.2 (IT2.2)-150Nd, Anti-Human CD103 (Ber-ACT8)-151Eu, Anti-Human cleaved Caspase 7 (D6H1)-152Sm, Anti-Human CD62L (DREG-56)-153Eu, Anti-Human TIM-3 (F38-2E2)-154Sm, Anti-Human CD27 (L128)-155Gd, Anti-Human CD14 (HCD14)-156Gd, Anti-Human CD134/ OX40 (ACT35)-158Gd, Anti-Human FoxP3 (259D/C7)-159Tb, Anti-Human CD39 (A1)-160Gd, Anti-Human/Mouse Tbet (4B10)-161Dy, Anti-Human CD69 (FN50)-162Dy, Anti-Human TGFbeta (TW4- 6H10)-163Dy, Anti-Human IL-17A (N49-653)-164Dy, Anti-Human IFNg (B27)-165Ho, Anti-Human IL-10 (JES3-9D7)-166Er, Anti-Human CD73 (AD2)-168Er, Anti-Human CD159a/NKG2A (Z199)-169Tm, Anti-Human CD45RA (HI100)-170Er, Anti-Human CD226 (DX11)-171Yb, Anti-Human Ki-67 (B56)-172Yb, Anti-Human HLADR (L243)-173Yb, Anti-Human CD279/ PD-1 (EH12.2H7)-174Yb, Anti-Human TNFa (Mab11)-175Lu, Anti-Human CD56 (HCD56)-176Yb, and Anti-Human TIGIT (MBSA43)-209Bi. Three antibodies were purchased and then conjugated to metals through the HIMSR core using Fluidigm conjugation kits: ephrinB2 (R&D Systems (Arg27-ALA227))-139, EphA4 (ThermoFisher (21875-1-AP))-115, and TCF1 (Biolegend (TCF6))-167Er. Human FC block used: Human BD Fc Block (BD Pharmagen).

Instrument

Fluidigm CyTOF Helios (Mass Cytometer)

Software	FlowJo.app version 10.7.1; Astrolabediagnostics.com; Cytobank.com
Cell population abundance	No cell sorting was conducted.
Gating strategy	Gating strategy for memory cells was done by Astrolabe Diagnostics.

☒ Tick this box to confirm that a figure exemplifying the gating strategy is provided in the Supplementary Information.
