# Supplementary material for: A phase I/Ib trial and biological correlate analysis of neoadjuvant SBRT with single-dose durvalumab in HPV-unrelated locally advanced HNSCC
Source: Nat Cancer. 2022 Nov 25;3(11):1300–17. doi: 10.1038/s43018-022-00450-6 (PMC9701140; doi:10.1038/s43018-022-00450-6)
Supplement: Supplementary file 2 — Clinical trial protocol [file 43018_2022_450_MOESM2_ESM.pdf]

Investigational Drug Substance(s)	Durvalumab (MEDI4736)
Study Number	ESR-17-13229
Version Number	8.0
Date	30 March 2021

Phase I/Ib trial of radiotherapy in combination with Durvalumab (MEDI4736) prior to surgical resection for HPV/p16 negative squamous cell carcinoma of the head and neck (HNSCC)

Sponsor: Sana Karam, MD, PhD

This is an investigator-initiated study. The principal investigator (PI), Sana Karam, MD, Ph.D., is conducting the study and acting as the sponsor. As the sponsor-investigator, both the legal/ethical obligations of a PI and those of a sponsor will be followed.

The trial will be carried out in accordance with Good Clinical Practice (GCP) as required by applicable United States (US) laws and applications, including but not limited to United States (US) Code of Federal Regulations (CFR) applicable to clinical studies (45 CFR Part 46, 21 CFR Part 50, 21 CFR Part 56, 21 CFR Part 312, and/or 21 CFR Part 812)

The PI will assure that no changes to the protocol will take place without documented approval from the Institutional Review Board (IRB). All personnel involved in the conduct of this study have completed Human Subjects Protection Training.

I agree to ensure that all staff members involved in the conduct of this study are informed about their obligations in meeting the above commitments.

Sponsor-Principal Investigator: ____

Print/Type Name

Signed: ____ **Date:** ___________

PROTOCOL CONTACT LIST

Principal Investigator

Sana Karam, MD, PhD
Assistant Professor
University of Colorado
Department of Medicine
Division of Radiation Oncology
1665 Aurora Court, Suite 1032
Aurora, CO 80045
Phone: 720-848-0116
Fax: 720-848-0222
Email: sana.karam@cuanschutz.edu

PROTOCOL SYNOPSIS

Clinical Protocol ESR-17-13229

**Study Title:** Phase I/Ib trial of radiotherapy in combination with Durvalumab (MEDI4736) prior to surgical resection for HPV/p16 negative squamous cell carcinoma of the head and neck (HNSCC)
**Protocol Number:** ESR-17-13229
**Clinical Phase:** Phase I/Ib
**Study Duration:** 12-24 months
**Investigational Product(s) and Reference Therapy:** Durvalumab (MEDI4736) will be supplied in glass vials containing 500 mg of MEDI4736 as a solution at a concentration of 50 mg/mL for intravenous (IV) infusion after dilution.
**Research Hypothesis:** Combination neoadjuvant durvalumab plus hypofractionated radiation plus postoperative adjuvant durvalumab in surgically resectable stage II-IVB oral cavity, stage III-IVB larynx and hypopharynx, or stage III-IVB HPV/p16 negative intermediate-high risk oropharynx head and neck cancer is safe and tolerable.
**Objectives:**<br><br>**Primary Objectives:**<br>Determine the Maximum Tolerated Dose (MTD) and Dose Limiting Toxicities (DLT) of SBRT in combination with durvalumab + postoperative adjuvant durvalumab in surgically resectable stage II-IVB oral cavity, stage III-IVB larynx and hypopharynx, or stage III-IVB HPV/p16 negative intermediate-high risk oropharynx head and neck cancer.<br><br>**Secondary Objective(s)**:<br>1. Determine pathologic response (in diagnostic biopsy and surgical specimens) to durvalumab + RT prior to surgery.<br>2. Determine clinical response (via imaging and clinical exam) at about 3 months from the end of adjuvant radiation (if given), or for those that do not receive adjuvant radiation, at about 3 months from the end of Cycle 2 adjuvant durvalumab. This includes locoregional control, distant control, and overall survival.<br>3. Evaluate biomarkers of immune response to treatment protocol in tumor and blood samples (including circulating tumor cells) and intratumoral CD8 infiltration.<br>4. Assess incidence of adverse pathologic features that constitute indications for adjuvant therapy.<br>5. Define toxicity profile associated with treatment protocol.<br>6. Assess short-term quality-of-life associated with treatment protocol.<br><br>**Exploratory Objective(s):**<br>Translational studies will require blood samples, initial biopsy, and tumor tissue at the time of surgery.

1. To establish a gene signature panel that can assess the response to RT-anti-PDL1 response and treatment outcome.
2. Compare change in anti-PDL1 and T cell infiltration pattern and intensity before and after RT and anti-PDL1 by immunohistochemistry.
3. To evaluate the impact of other mutations on immune response and treatment outcome.
4. Blood samples to evaluate biomarkers of immune response as well as for evaluation of circulating auto-antibodies. Blood will be collected prior to treatment, at time of surgery, at end of treatment and at the 6 month follow-up.
5. Tumor tissue taken at the time of initial biopsy and at time of resection will be profiled for tumor infiltrating lymphocytes; activation markers and antigen specific TCR utilization/diversity will be evaluated for additional checkpoint targets.
6. On long-term follow-up, tumor antigen specific T lymphocyte memory populations will be monitored for representation and robustness in in-vitro stimulation assays as potential biomarker of continued anti-tumor activity.

Study Design:

This is a multi-center, prospective, single-arm phase I/Ib safety trial. Patients eligible for treatment must be diagnosed with non-metastatic, biopsy-proven stage II-IVB oral cavity, stage III-IVB larynx and hypopharynx, or stage III-IVB HPV/p16 negative intermediate-high risk oropharynx head and neck cancer, and must be eligible and amenable to surgical resection.

This study will be enrolled using a 3+3 model. Patients will receive one dose of neoadjuvant durvalumab 1500 mg approximately 3-6 weeks prior to standard of care surgery. It will be given concurrently with the first dose of radiation (RT). The starting RT dose level will be given as 6 Gy for 2 fractions (12 Gy total) every other day over approximately one week to sites of gross disease (Table 1) only to minimize exposure to normal tissue. If toxicity develops and surgery is delayed by more than 8 weeks (qualifying as a DLT), the radiation dose will be dropped per protocol for the next set of patients. If this dose is tolerated, the dose will be increased to 6 Gy for 3 fractions (18 Gy total) for the next 3 patients. Patients will proceed to surgical resection approximately 3-6 weeks after radiation as recommended by the ENT surgeon.

Table 1: Dose Escalation Schedule:

Dose Level	Treatment
1 (-1)	1500 mg Durvalumab + 1 fraction of 6 Gy radiation (6 Gy total)
2 (Starting Dose)	1500 mg Durvalumab + 2 fractions of 6 Gy radiation (12 Gy total)
3 (+1)	1500 mg Durvalumab + 3 fractions of 6 Gy radiation (18 Gy total)

Post-operatively, pathology will be reviewed and the need for **adjuvant therapy** will be guided by high risk features **as per the NCCN guidelines** and treating physician recommendations. Adjuvant radiation will include intensity modulated radiation therapy consisting of 60 Gy in 2 Gy once-daily fraction size (total of 30 fractions). If indicated, adjuvant systemic therapy can include cisplatin or other cytotoxic chemotherapy or targeted biologics (Cetuximab) per physician discretion.

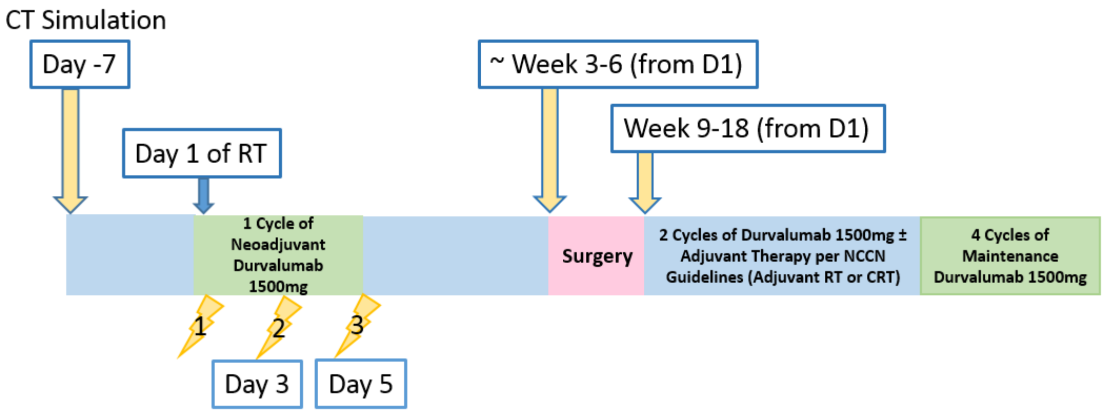

All patients will receive adjuvant durvalumab to be initiated approximately 6-12 weeks after surgery. It will be given as 1500 mg intravenously once every 4 weeks for a maximum of 6 doses, or until progression, toxicity or withdrawal from study. This will either be as monotherapy or concurrently with adjuvant radiation +/- systemic therapy for high risk patients.

Safety and toxicity evaluations will be done throughout the study process. DLTs and adjustment of radiation doses will be done during the neoadjuvant period.

Imaging evaluating response:
Patients enrolled will obtain baseline pretreatment imaging, consisting of MRI neck with and without contrast and PET-CT skull base to mid-thigh. Just prior to surgery, all patients will undergo repeat PET-CT (preferable) and/or MRI neck with and without contrast for treatment response evaluation if PET is not possible or if needed by the surgical team. At about 3 months from the end of adjuvant radiation (if given), or for those that do not receive adjuvant radiation, at about 3 months from the end of Cycle 2 adjuvant durvalumab, all patients will undergo post-treatment PET-CT skull base to mid-thigh and/or MRI neck with and without contrast. It is recommended that all radiology evaluations be done on the same machine when possible, but not mandatory.

Number of Centers: Multi-center - UCHealth Metro (University of Colorado Cancer Center), UCHealth North (Poudre Valley Hospital) and UCHealth South (Memorial Hospital).

Number of Patients: The expected maximum number of patients to be consented is 28 to allow for screen failures.

Study Population: Stage II-IVB oral cavity, stage III-IVB larynx and hypopharynx, or stage III-IVB HPV and/or p16 negative intermediate-high risk oropharynx head and neck squamous cell carcinoma cancer that is deemed resectable or borderline resectable.

Inclusion Criteria:

1. Histologically or cytologically confirmed: stage II-IVB oral cavity, stage III-IVB larynx, stage III-IVB hypopharynx, or stage III-IVB HPV and/or p16 negative intermediate-high risk oropharynx head and neck cancer (AJCC 8th edition)

2. Measurable disease defined as lesions that can be accurately measured in at least one dimension (longest diameter to be recorded) as >10 mm by CT, PET/CT or MRI or >10 mm on visual inspection by clinical exam
3. Patients who are deemed resectable by ENT surgeon without pre-existing medical conditions that could inhibit surgery following neoadjuvant therapy, and do not refuse surgery
4. Written informed consent and HIPAA authorization obtained from the patient prior to performing any protocol-related procedures, including screening evaluations
5. Age ≥ 18 years at time of study entry
6. ECOG performance status ≤ 1
7. Life expectancy ≥ 24 weeks
8. Body weight >30kg
9. Adequate normal organ and marrow function as defined below:
    - Hemoglobin ≥9.0 g/dL
    - Absolute neutrophil count (ANC) ≥ 1.0 x 10^9/L (≥ 1000 per mm^3)
    - Platelet count ≥75 x 10^9/L (≥75,000 per mm^3)
    - Serum bilirubin ≤1.5 x institutional upper limit of normal (ULN). This will not apply to patients with confirmed Gilbert's syndrome (persistent or recurrent hyperbilirubinemia that is predominantly unconjugated in the absence of hemolysis or hepatic pathology), who will be allowed only in consultation with their physician.
    - AST (SGOT)/ALT (SGPT) ≤2.5 x institutional upper limit of normal
    - Measured creatinine clearance (CL) >40 mL/min or Calculated creatinine CL>40 mL/min by the Cockcroft-Gault formula (Cockcroft and Gault 1976) or by 24-hour urine collection for determination of creatinine clearance:

    Males:

    $$\text{Creatinine CL (mL/min)} = \frac{\text{Weight (kg)} \times (140 - \text{Age})}{72 \times \text{serum creatinine (mg/dL)}}$$

    Females:

    $$\text{Creatinine CL (mL/min)} = \frac{\text{Weight (kg)} \times (140 - \text{Age}) \times 0.85}{72 \times \text{serum creatinine (mg/dL)}}$$

10. Evidence of post-menopausal status or negative urinary or serum pregnancy test for female pre-menopausal patients. Women will be considered post-menopausal if they have been amenorrheic for 12 months without an alternative medical cause. The following age-specific requirements apply:
    - Women <50 years of age would be considered post-menopausal if they have been amenorrheic for 12 months or more following cessation of exogenous hormonal treatments and if they have luteinizing hormone and follicle-stimulating hormone levels in the post-menopausal range for the institution or underwent surgical sterilization (bilateral oophorectomy or hysterectomy)
    - Women ≥50 years of age would be considered post-menopausal if they have been amenorrheic for 12 months or more following cessation of all exogenous hormonal treatments, had radiation-induced menopause with last menses >1 year ago, had chemotherapy-induced menopause with last menses >1 year

ago, or underwent surgical sterilization (bilateral oophorectomy, bilateral salpingectomy or hysterectomy)

11. Patient is willing and able to comply with the protocol for the duration of the study including undergoing treatment and scheduled visits and examinations including follow up

Exclusion Criteria:

1. Participation in another clinical study with an investigational product during the last 3 months
2. Patients with active ILD / pneumonitis or with a history of ILD/ pneumonitis requiring steroids
3. Concurrent enrolment in another clinical study, unless it is an observational (non-interventional) clinical study or during the follow-up period of an interventional study
4. Any previous treatment with a PD1 or PD-L1 inhibitor, including durvalumab, anti-PD-L2, anti-CD137, or anti-cytotoxic T-lymphocyte-associated antigen-4 (CTLA-4) antibody (including ipilimumab or any other antibody or drug specifically targeting T-cell co-stimulation or checkpoint pathways)
5. Receipt of the last dose of anticancer therapy (chemotherapy, immunotherapy, endocrine therapy, targeted therapy, biologic therapy, tumor embolization, monoclonal antibodies, other investigational agent) 30 days prior to the first dose of study drug for patients who have received prior TKIs [e.g., erlotinib, gefitinib and crizotinib] and within 6 weeks for nitrosourea or mitomycin C. (*If sufficient wash-out time has not occurred due to the schedule or PK properties of an agent, a longer wash-out period may be required.)*
6. Patients with QTc interval > 470 msec during screening
7. Current or prior use of immunosuppressive medication within 14 days before the first dose of durvalumab, with the exceptions of intranasal and inhaled corticosteroids or systemic corticosteroids at physiological doses, which are not to exceed 10 mg/day of prednisone, or an equivalent corticosteroid. The following are exceptions to this criterion:
    - Intranasal, inhaled, topical steroids, or local steroid injections (e.g., intra articular injection)
    - Systemic corticosteroids at physiologic doses not to exceed 10 mg/day of prednisone or its equivalent
    - Steroids as premedication for hypersensitivity reactions (e.g., CT scan premedication)
8. Any concurrent chemotherapy, IP, biologic, or hormonal therapy that is not part of standard NCCN indicated HNSCC adjuvant concurrent CRT. Concurrent use of hormonal therapy for non-cancer-related conditions (e.g., hormone replacement therapy) is acceptable.
9. History of allogenic organ or bone marrow transplantation
10. Active or prior documented autoimmune or inflammatory disorders (including inflammatory bowel disease [e.g., colitis or Crohn's disease], diverticulitis [with the exception of diverticulosis], systemic lupus erythematosus, Sarcoidosis syndrome, or Wegener syndrome [granulomatosis with polyangiitis, Graves' disease, rheumatoid arthritis, hypophysitis, uveitis, etc]). The following are exceptions to this criterion:
    - Patients with vitiligo or alopecia

- Patients with hypothyroidism (e.g., following Hashimoto syndrome) stable on hormone replacement
- Any chronic skin condition that does not require systemic therapy
- Patients without active disease in the last 5 years may be included but only after consultation with the study physician
- Patients with celiac disease controlled by diet alone

11. Uncontrolled intercurrent illness, including but not limited to, ongoing or active infection, symptomatic congestive heart failure, uncontrolled hypertension, unstable angina pectoris, cardiac arrhythmia, interstitial lung disease, serious chronic gastrointestinal conditions associated with diarrhea, or psychiatric illness/social situations that would limit compliance with study requirement, substantially increase risk of incurring AEs or compromise the ability of the patient to give written informed consent
12. History of another primary malignancy except for:
    - Malignancy treated with curative intent and with no known active disease ≥3 years before the first dose of IP and of low potential risk for recurrence
    - Adequately treated non-melanoma skin cancer or lentigo maligna without evidence of disease
    - Adequately treated carcinoma in situ without evidence of disease
13. History of leptomeningeal carcinomatosis
14. History of active primary immunodeficiency
15. Active infection including **tuberculosis** (clinical evaluation that includes clinical history, physical examination and radiographic findings, and TB testing in line with local practice), **hepatitis B** (known positive HBV surface antigen (HBsAg) result), **hepatitis C**, or **human immunodeficiency virus** (positive HIV 1/2 antibodies). Patients with a past or resolved HBV infection (defined as the presence of hepatitis B core antibody [anti-HBc] and absence of HBsAg) are eligible. Patients positive for hepatitis C (HCV) antibody are eligible only if polymerase chain reaction is negative for HCV RNA.
16. Receipt of live attenuated vaccine within 30 days prior to the first dose of IP. Note: Patients, if enrolled, should not receive live vaccine whilst receiving IP and up to 30 days after the last dose of IP.
17. Female patients who are pregnant or breastfeeding or male or female patients of reproductive potential who are not willing to employ highly effective birth control from screening to 90 days after the last dose of durvalumab monotherapy. Patient must have a negative serum or urine pregnancy test within 72 hours of study entry.
18. Known allergy or hypersensitivity to any of the study drugs or any of the study drug excipients
19. Prior randomisation or treatment in a previous durvalumab clinical study
20. Patients with p16-positive oropharyngeal SCCA. No verification of p16 status is needed for laryngeal cancer or oral cavity cancer.
21. Patients with sinonasal SCCAs
22. Patients with metastatic SCCA neck disease with an unknown primary tumor site
23. Patients with distant metastatic disease on initial screening imaging
24. Judgment by the investigator that the patient is unsuitable to participate in the study and the patient is unlikely to comply with study procedures, restrictions and requirements

Investigational Product(s), Dose and Mode of Administration:

Patients will receive up to seven total doses of 1500mg Durvalumab (MEDI4736) via IV infusion. One neoadjuvant dose will be administered concurrently with radiation in addition to up to six adjuvant doses administered (two doses administered as monotherapy or concurrently with adjuvant radiation +/- chemotherapy and four doses administered as maintenance treatment) unless there is unacceptable toxicity, withdrawal of consent, or another discontinuation criterion is met. (For example, if a patient's weight falls to 30kg or below the patient should receive weight-based dosing equivalent to 20 mg/kg of durvalumab Q4W until the weight improves to >30 kg, at which point the patient should start receiving the fixed dosing of durvalumab 1500 mg Q4W).

Study Assessments and Criteria for Evaluation:

Safety Assessments:

All patients who receive at least one dose of the investigational agent(s) will be included in the safety analysis. Frequency of AEs, SAEs, discontinuation of study drug due to AEs and changes from baseline laboratory parameter values will be evaluated.

- Toxicity assessments according to the NCI CTCAE v. 4.03
- Maximum tolerated dose per each cohort

Efficacy Assessments:

Tumor response to neoadjuvant therapy (durvalumab + SBRT) will be assessed by pathology review of the surgical specimen. Response will be labeled as complete pathologic remission, microscopic residual tumor (only scattered foci of residual tumor cells) or macroscopic residual tumor.

The method of assessment of disease status at baseline will be MRI neck with and without contrast *and* PET-CT skull base to mid-thigh. The baseline assessment should be performed no more than 28 days before SBRT. Efficacy for all patients will be assessed by objective tumor assessment by repeat PET-CT (preferable) and/or MRI neck with and without contrast for treatment response evaluation if PET is not possible or if MRI is needed by the surgical team. Response will be assessed on PET by comparing metabolic SUV of tumor (preferable if available) or measured reduction of gross tumor volume on clinical MRI, as determined by a neuroradiologist, if only MRI is performed. At about 3 months from the end of adjuvant radiation (if given), or for those that do not receive adjuvant radiation, at about 3 months from the end of Cycle 2 adjuvant durvalumab, all patients will undergo post-treatment PET-CT skull base to mid-thigh and/or MRI neck with and without contrast. It is recommended that all radiology evaluations be done on the same machine when possible, but not mandatory.

For patients who discontinue treatment due to toxicity in the absence of confirmed objective progression, objective tumor assessments per the schedule of study assessments should be continued.

Following confirmed progression, patients should continue to be followed up for survival every 12 weeks for 18 months.

Patients who achieve and maintain disease control through to the end of the treatment period will continue with follow-up every 12 weeks for 18 months as detailed in the Schedule of Study Assessments in section 8.1.

Statistical Methods and Data Analysis:

Study Design

Part 1 of this trial will use a traditional 3+3 design will be used for this trial (i.e. cohort sizes of 3 patients for the first and second cohort at each dose level). Dose escalation will occur as illustrated in the figure below.

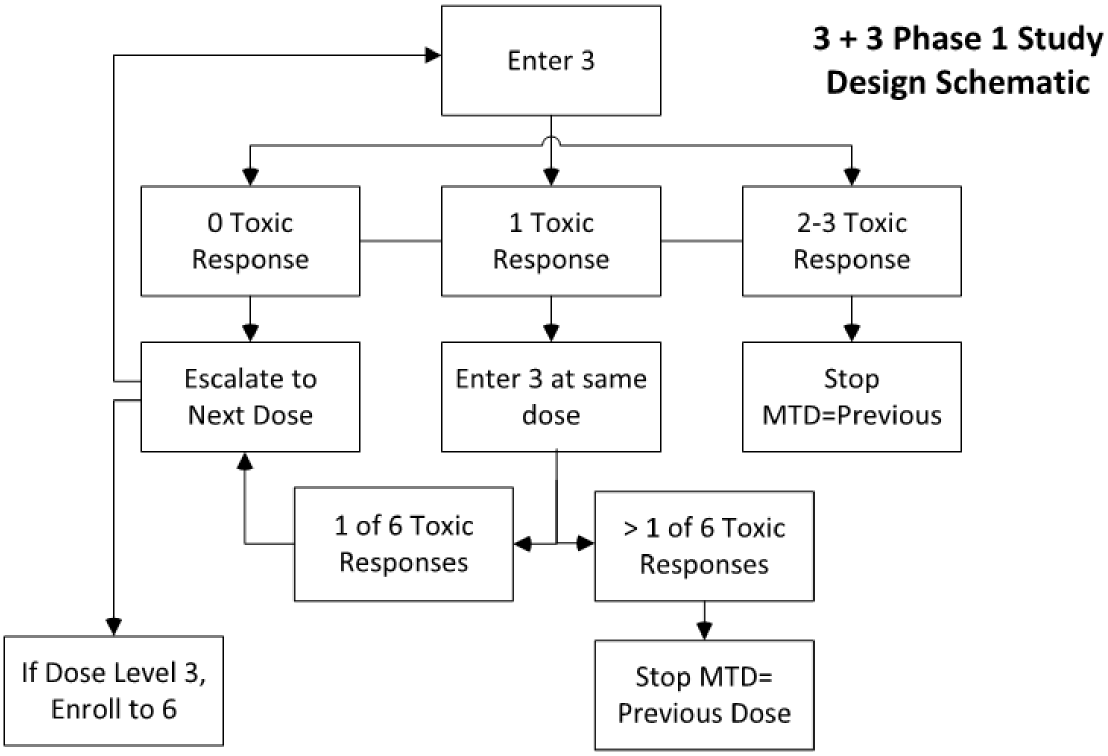

The starting dose level for the trial will be dose level 2. This will allow the trial to continue if there are 2 or 3 DLTs in the first cohort of 3 patients by de-escalating to dose level 1.

Part 2 of this trial will be an expansion cohort. A total of 8 additional patients will be enrolled at the dose level determined to be the MTD in part 1 of the study. These 8 patients will be used to confirm that the MTD is a safe combination, as well as provide additional patients to investigate the efficacy for the treatment combination. For safety purposes, DLT assessments will continue to be documented for patients enrolled in Part 2 of the trial.

Observation Period

Each subject will be observed for the occurrence of a DLT from the day on which the patient starts neoadjuvant durvalumab at 1500 mg up until the day of surgery. The expected duration of this observation period is approximately 3–6 weeks.

Interim Analyses

During part 1 of the trial, after all 3 patients in each cohort have either 1) completed the observation window, or 2) experienced a DLT, the data summarizing the primary endpoint for that cohort (number of DLTs in 3 patients) will be generated. This summary will determine what treatment the next cohort will receive. These may be considered to be interim analyses. There are no planned formal interim analyses during part 2 of the study.

Sample Size Determination:

Part 1: Dose Escalation

The expectation is that 9 patients will be enrolled to the trial during part 1. This is based on the expectation that all dose levels are safe (i.e. patients will not experience DLTs at all dose levels). In this case, 3 patients will be enrolled at dose level 2 (0/3 DLTs), then dose escalating to dose level 3 and enrolling a total of 6 patients at this dose level, for a total of 9 patients. The range of patients needed will be 6–12 patients.

Part 2: Dose Expansion

The primary outcome of this study is to assess safety. An additional 8 patients will be enrolled at the DLT dosing determined during part 1 for further safety assessment. This number was chosen to answer our secondary outcome of efficacy as measured by pathologic response and cytotoxic CD8 T cell infiltration. This is determined by the binary measure of whether or not a patient has relevant CD8 infiltration, defined as a 1.5-fold increase at tumor resection as compared to biopsy. Let p represent the proportion of patients with relevant CD8 infiltration. It is expected that half (50%) of patients not receiving this treatment will have relevant CD8 infiltration. The objective is to test the following hypotheses:

H_0: $p \leq 0.50$
H_1: $p \geq 0.80$

The hypotheses will be tested using the exact binomial test. A total of 14 patients are needed to have at least 80% power (87.0% to be exact) to discriminate between the hypotheses stated above when testing them using a 1-sided superiority test and controlling the type 1 error rate at 0.10.

Total Sample Size

During part 1, 6 patients will be treated at the MTD. The CD8 infiltration will be assessed on these 6, leaving an additional subjects to be enrolled during part 2 in order to achieve the total sample size of 14 needed for part 2. The total sample size is thus expected to be 17 patients (9 from part 1 + 8 from part 2) as the total sample size will depend on the number of patients needed during part 1 of the trial. The total sample size would be up to 20.

SCHEDULE OF STUDY ASSESSMENTS

	Screening	SBRT Fraction 1 & Cycle 1	SBRT Fraction 2	SBRT Fraction 3	Pre-op Clinical Assess[w].	Surgical Resection	Adjuvant RT ± C	Cycle 2 & 3[p]	Cycle 4-6[x]	Unsched. Visits[r]	Cycle 7 /EOT[x]	Follow up[a]
**Assessment Day/Week**	**D-28 to 0**	**D1**	**D3**	**D5**		**W3-6[v]**	**W9-18**	**W9-18**	**Q4W**			
**Window**		**+3 days**	**±3 Days**	**±3 Days**				**±2 days**	**±2 Days**		**±14 Days**	**±28 Days**
Informed Consent	X											
Review of Eligibility Criteria	X											
Review of Adverse Events	X	X			X	X	X	X	X	X	X	X
Medical History[b]	X											
Concomitant Medications[c]	X	X			X		X	X	X	X	X	X
Durvalumab Administration (neoadjuvant)		X[u]										
CT Simulation	X											
Radiation Therapy		X	X	X			X					
Surgery						X						
Durvalumab (adjuvant)								X	X		X	
Adjuvant Systemic Concurrent Therapy[n]							X					
Physical Examination[d]	X	X			X		X[o]	X	X	X	X	X
ECOG Performance Status	X	X			X		X[o]	X	X	X	X	X
Vital Signs Including Weight[e]	X	X			X		X[o]	X	X	X	X	X
Electrocardiogram[f]	X											
FACT-HN	X				X			X	X		X	X
Survival & Anti-cancer Therapy Follow-up											X	X
Hematology & Serum Chemistry[h]	X	X			X		X[o]	X	X		X	X
Pregnancy Test[i]	X	X										
Thyroid Function[j]	X				X			X	X		X	X
Urinalysis[k]	X											
Coagulation Parameters[l]	X				X							
Serology[m]	X											
Blood for Biomarker & Immunogenicity Analysis		X[s]				X					X	X[s]
Tumor Biopsy & Surgical Specimen[g]	X					X						
MRI Neck with and without contrast	X				X[y]		X[q]		X[q]			X[t]
PET/CT skull base to mid-thigh	X				X[y]		X[q]		X[q]			X[t]

[a] Follow up will be q12 weeks ± 28 days for 18 months following durvalumab therapy for those with no evidence of progressive/recurrent disease. For those with progressive/recurrent/metastatic disease found during the study, a visit or telephone call with updated Survival and Anti-cancer Therapy Follow-up can be done alone.

[b] Medical history will include surgical and cancer histories, demographic information, history of tobacco and alcohol use, all active conditions, and any condition diagnosed within the prior 10 years that are considered to be clinically significant by the Investigator.

[c] Medications taken within Day 0 of study.

[d] Full physical examination at baseline; targeted physical examination at other time points.

[e] Vital signs include temperature, resting blood pressure, pulse, and respiratory rate. For the initial infusion, vitals will be collected prior to the beginning of the infusion (measured once from approximately 30 minutes before up to 0 minutes [i.e., the beginning of the infusion]), approximately 30 minutes during the infusion (halfway through infusion), and at the end of the infusion (approximately 60 minutes ±5 minutes). If the infusion takes longer than 60 minutes, then BP and pulse measurements should follow the principles as described above or be taken more frequently if clinically indicated, and 60 minutes after the infusion for the first cycle. For subsequent infusions, vitals will be collected prior to the beginning of infusion (within 30 minutes) and then per institution standards or as clinically indicated. Weight will only be measured once at each visit, pre-treatment. Height will be collected only at screening.

[f] Electrocardiogram during screening (single), thereafter as clinically indicated.

[g] Baseline tumor biopsy is required for all patients, see section 8.3.4 for details. In addition to the baseline tumor biopsy, archival tissue should be submitted for each patient. Surgical resection specimen following neoadjuvant therapy will be used for histopathologic assessment of treatment response.

[h] See table 5 for list of hematology laboratory tests and table 6 for list of clinical chemistry laboratory tests. At screening and EOT, all labs listed in tables 5 and 6 will be collected. At the pre-op visit, treatment visits and follow-up visits, all hematology labs in table 5 will be collected and only limited chemistry labs (to include albumin, alk phos, ALT, AST, bicarbonate, calcium, chloride, creatinine, glucose, potassium, sodium, total bili, total protein and BUN) will be collected, all other chemistry labs (amylase, lipase, GGT, LDH, uric acid and magnesium) will be collected only as needed. C1D1 labs do not need to be repeated if the screening lab assessments were performed within 3 days prior to Day 1.

[i] Serum or urine pregnancy test for pre-menopausal female subjects of childbearing potential only. Screening test to be performed within 72 hours of study entry.

[j] Thyroid function will consist of TSH. If altered, total T3 and free T4 will be required.

[k] Urinalysis (specific gravity, pH, color, appearance, bilirubin, glucose, protein, ketones, and blood) performed at screening and as clinically indicated.

[l] Coagulation tests: prothrombin time, PTT and INR performed at Screening, Pre-op, and as clinically indicated.

[m] Serologies: Hepatitis B surface antigen (HbsAg), hepatitis C antibody, and HIV antibody.

[n] Systemic therapy drug, dose, and schedule per treating physician discretion.

[o] Blood work and patient assessment schedule during adjuvant standard of care RT +/- systemic therapy per treating physician discretion.

[p] For patients receiving adjuvant radiation and/or chemotherapy, the first dose of Durvalumab will be given on the same start date +/-7 days as radiation.

[q] Post-op MRI and/or PET/CT should be performed at about 3 months from the end of adjuvant radiation, if given. For those that do not receive adjuvant radiation, imaging will be performed at about 3 months from the end of Cycle 2 adjuvant durvalumab or at the discretion of the treating physician.

[r] Unscheduled visits are any standard of care visits requested by treating physician(s) or unplanned visits for symptom management that are not required by the study.

[s] Blood will be collected within 7 days prior to or on D1 of study treatment. During follow-up, blood will be collected at the 6 month (24 week) follow-up visit only.

[t] Long term follow up can be done with PET CT and/or MRI at the discretion of the treating physician.

[u] The first dose (neo-adjuvant dose) of durvalumab can be administered at any time between SBRT fractions 1-3.

[v] 3-6 weeks or at the discretion of the surgeon.

[w] Pre-Op assessment should be done within 7 days prior to surgical resection or at surgeon's discretion.

[x] Each cycle of adjuvant Durvalumab should be 4 weeks apart (± 2 days)

[y] Pre-op PET-CT is preferable. If a PET is not approved, an MRI should be ordered. Both PET and MRI can be ordered if possible. If an MRI is needed for surgical planning, then a PET can be performed first and then an MRI.

TABLE OF CONTENTS PAGE

PROTOCOL SYNOPSIS 4
SCHEDULE OF STUDY ASSESSMENTS 13
TABLE OF CONTENTS 15
ABBREVIATIONS AND DEFINITION OF TERMS 19
1. INTRODUCTION 22
1.1 Disease background 22
1.1.1 Immunotherapies 22
1.2 Durvalumab background/non-clinical and clinical experience 24
1.3 Research hypothesis 25
1.4 Rationale for conducting this study 25
1.4.1 Durvalumab monotherapy dose rationale 25
1.4.2 Rationale for fixed dosing 26
1.4.3 Rationale for hypofractionated radiation 27
1.4.4 Rationale for neoadjuvant studies 28
1.4.5 Rationale for concurrent and adjuvant treatment 28
1.4.6 Study population rationale 29
1.5 Benefit/risk and ethical assessment 29
1.5.1 Potential benefits 29
1.5.2 Overall risks 29
2. PROVIDE SUMMARY OF OVERALL BENEFIT-RISK FOR THE STUDY OBJECTIVES 30
2.1 Primary objective(s) 30
2.2 Secondary objective(s) 31
2.3 Exploratory objective(s) 31
3. STUDY DESIGN 31
3.1 Overview of study design 31
3.2 Sample size and power considerations 32
3.3 Definition of analysis populations 33
3.3.1 Full analysis set (FAS) 33
3.3.2 Evaluable analysis set (EAS) 33
3.3.3 Safety analysis set (SAS) 33
3.4 Patient disposition and characteristics 34
3.5 Planned Methods of Analysis 34
3.5.1 Analysis of primary endpoint for safety 34
3.5.2 Analysis of secondary endpoints 35
3.5.3 Analysis of exploratory endpoints 36
3.6 Interim Analyses 37
3.7 Study schema 37
3.8 Study Design 37
3.9 Study oversight for safety evaluation 38
3.10 Interim analyses 38
3.11 Clinical criteria for early trial termination 38
4. PATIENT SELECTION 38
4.1 Inclusion criteria 38
4.2 Exclusion criteria 40
4.3 Withdrawal of patients from study treatment and/or study 42
4.3.1 Permanent discontinuation of durvalumab and/or RT 42

4.3.2 Withdrawal of consent 422
4.4 Replacement of patients 43
5. INVESTIGATIONAL PRODUCT(S)..43
5.1 Durvalumab 43
5.1.1 Formulation/packaging/storage..43
5.1.2 Durvalumab doses and treatment regimens..43
5.1.3 Study drug preparation 44
5.1.4 Monitoring of dose administration..45
5.1.5 Accountability and dispensation..45
5.1.6 Disposition of unused investigational study drug 46
5.2 Standard of Care Adjuvant Therapy..46
5.2.1 Formulation/packaging/storage..46
5.2.2 Doses and treatment regimens..46
5.2.3 Product preparation 46
5.2.4 Dose administration 46
5.2.5 Monitoring of dose administration..46
5.2.6 Accountability and dispensation..46
6. TREATMENT PLAN..47
6.1 Patient enrollment 47
6.1.1 Procedures handling patients incorrectly enrolled 47
6.2 Dosage and administration 47
6.2.1 Neoadjuvant 47
6.2.2 Adjuvant 47
6.3 Dose escalation decision rules..48
6.4 Definition of DLT..48
6.5 Tracking toxicity of investigational product(s)..49
6.6 Toxicity management guidelines 49
6.6.1 Durvalumab 49
6.7 Neoadjuvant (Preoperative) Radiation Therapy..50
6.7.1 Technical factors 50
6.7.2 Set up, treatment planning and localization..50
6.7.3 Critical structures..51
6.7.4 Image Guidance for Target Localization..55
6.7.5 ADJUVANT (POSTOPERATIVE) RADIATION THERAPY 55
6.7.6 Compliance Criteria 62
6.7.7 Radiation therapy monitoring..63
6.8 Surgery..63
6.8.1 Resection of the primary tumor..63
6.8.2 Neck Dissection..64
6.8.3 Neck lymph node levels 64
6.8.4 Surgical Compliance..65
7. RESTRICTIONS DURING THE STUDY AND CONCOMITANT TREATMENT(S)..66
7.1 Restrictions during the study 66
7.2 Concomitant treatment(s) 67
7.2.1 Permitted concomitant medications 68
7.2.2 Excluded concomitant medications..68
8. STUDY PROCEDURES..69
8.1 Schedule of study procedures..69
8.1.1 Screening phase..70
8.1.2 Treatment phase 71

8.1.3 End of treatment 71
8.2 Description of study procedures 71
8.2.1 Medical history and physical examination, electrocardiogram, weight, and vital signs 71
8.2.2 Clinical laboratory tests 72
8.2.3 Patient reported outcomes (PRO) 74
8.3 Biological sampling procedures 74
8.3.1 Immunogenicity sampling and evaluation methods 74
8.3.2 Biomarker sampling and evaluation methods 75
8.3.3 Blood collection 75
8.3.4 Tissue collection 76
9. DISEASE EVALUATION AND METHODS 77
9.1.1 Efficacy Assessment 77
10. ASSESSMENT OF SAFETY 79
10.1.1 Safety parameters 79
10.1.2 Definition of serious adverse events 80
10.1.3 Definition of adverse events of special interest (AESI) 80
10.2 Assessment of safety parameters 82
10.2.1 Assessment of severity 82
10.2.2 Assessment of relationship 83
10.3 Recording of adverse events and serious adverse events 83
10.3.1 Study recording period and follow-up for adverse events and serious adverse events 84
10.3.2 Causality collection 85
10.3.3 Relationship to protocol procedures 855
10.3.4 Adverse events based on signs and symptoms 86
10.3.5 Adverse events based on examinations and tests 86
10.3.6 Hy's Law 86
10.3.7 Disease progression 86
10.3.8 Deaths 86
10.3.9 Reporting of serious adverse events 87
10.3.10 Reporting of deaths to AstraZeneca 88
10.3.11 Overdose 88
10.3.12 Hepatic function abnormality 89
10.3.13 Pregnancy 89
10.4 Medication error 90
11. STATISTICAL METHODS AND SAMPLE SIZE DETERMINATION 91
11.1 Description of analysis sets 91
11.2 Methods of statistical analyses 91
11.3 Patient disposition and characteristics 91
12. ETHICAL AND REGULATORY REQUIREMENTS 911
12.1 Ethical conduct of the study 911
12.2 Ethics and regulatory review 92
12.3 Informed consent 92
12.4 Changes to the protocol and informed consent form 92
12.5 Audits and inspections 922
13. STUDY MANAGEMENT 93
13.1 Training of study site personnel 93
13.2 Handling and Documentation of Clinical Supplies 93
13.3 Monitoring of the study 93

14. DATA MANAGEMENT 95
14.1 Study governance and oversight 95
14.2 Record Keeping and Record Retention 95
14.3 Regulatory Documentation 95
15. PROTECTION OF HUMAN SUBJECTS 965
15.1 Protection from Unnecessary Harm 965
15.2 Protection of Privacy 96
16. LIST OF REFERENCES 97

LIST OF TABLES

TABLE 1 DOSE ESCALATION SCHEDULE....5
TABLE 2 HIGHLY EFFECTIVE METHODS OF CONTRACEPTION....67
TABLE 3 SUPPORTIVE MEDICATIONS....68
TABLE 4 PROHIBITED MEDICATIONS....68
TABLE 5 HEMATOLOGY LABORATORY TESTS....73
TABLE 6 CLINICAL CHEMISTRY LABORATORY TESTS....73
TABLE 7 URINALYSIS TESTS....73
TABLE 8 VOLUME OF BLOOD TO BE DRAWN FROM EACH PATIENT....76

LIST OF FIGURES

Figure 1. 37

LIST OF APPENDICES

Appendix 1……..103
Appendix 2……..131

ABBREVIATIONS AND DEFINITION OF TERMS

The following abbreviations and special terms are used in this study Clinical Study Protocol.

Abbreviation or special term	Explanation
ADA	Anti-drug antibody
ADCC	Antibody-dependent cell mediated cytotoxicity
AE	Adverse event
AESI	Adverse event of special interest
ALP	Alkaline phosphatase
ALT	Alanine aminotransferase
APC	Antigen-presenting cells
AST	Aspartate aminotransferase
AUC	Area under the concentration-time curve
CDC	Complement-dependent cytotoxicity
CI	Confidence interval
CL	Clearance
C_{max}	Peak concentration
$C_{max,ss}$	Peak concentration at steady state
C_{min}	Trough concentration
$C_{min,ss}$	Trough concentration at steady state
CNS	Central nervous system
CR	Complete response
CT	Computed tomography
CTLA-4	Cytotoxic T-lymphocyte-associated antigen-4
DC	Disease control
DCR	Disease control rate
DLT	Dose-limiting toxicity
DNA	Deoxyribonucleic acid
DoR	Duration of response
ECG	Electrocardiogram

Abbreviation or special term	Explanation
ECOG	Eastern Cooperative Oncology Group
EDTA	Disodium edetate dihydrate
Fc	Fragment crystallizable
FFPE	Formalin fixed paraffin embedded
FSH	Follicle-stimulating hormone
FTIH	First-time-in-human
GCP	Good Clinical Practice
GMP	Good Manufacturing Practice
GLP	Good Laboratory Practice
HCl	Hydrochloride
HIV	Human immunodeficiency virus
ICF	Informed consent form
ICH	International Conference on Harmonization
IEC	Independent Ethics Committee
IFN	Interferon
IGF	Insulin-like growth factor
IgG1	Immunoglobulin G1
IgG2	Immunoglobulin G2
IGSF	Immunoglobulin superfamily
IHC	Immunohistochemistry
IL	Interleukin
irAE	Immune-related adverse event
IRB	Institutional Review Board
IV	Intravenous(ly)
MAb	Monoclonal antibody
MDSC	Myeloid-derived suppressor cells
MedDRA	Medical Dictionary for Regulatory Activities
miRNA	Micro ribonucleic acid
MRI	Magnetic resonance imaging
mRNA	Messenger ribonucleic acid
MTD	Maximum tolerated dose

Abbreviation or special term	Explanation
NCI CTCAE	National Cancer Institute Common Terminology Criteria for Adverse Events
NK	Natural killer
NOAEL	No-observed-adverse-effect level
NSCLC	Non-small cell lung cancer
OR	Objective response
ORR	Objective response rate
OS	Overall survival
PBMC	Peripheral blood mononuclear cell
PD	Progressive disease
PD-1	Programmed cell death 1
PD-L1	Programmed cell death ligand 1
PD-L2	Programmed cell death ligand 2
PFS	Progression-free survival
PK	Pharmacokinetic(s)
PR	Partial response
PRO	Patient-reported outcome
PVC	Polyvinyl chloride
Q2W	Every 2 weeks
Q3M	Every 3 months
Q3W	Every 3 weeks
Q4W	Every 4 weeks
Q12W	Every 12 weeks
QoL	Quality of life
QTc	Time between the start of the Q wave and the end of the T wave corrected for heart rate
QTcF	QT interval on ECG corrected using the Frederica’s formula
RCC	Renal cell carcinoma
RNA	Ribonucleic acid
SAE	Serious adverse event
SBRT	Stereotactic Body Radiation Therapy
SD	Stable disease

Abbreviation or special term	Explanation
SID	Subject identification
sPD-L1	Soluble programmed cell death ligand 1
SOCS3	Suppressor of cytokine signaling 3
SUSAR	Suspected unexpected serious adverse reaction
$t_{½}$	Half life
TEAE	Treatment-emergent adverse event
TIL	Tumor infiltrating lymphocyte
T_{max}	Time to peak concentration
$T_{max,ss}$	Time to peak concentration at steady state
TNF-α	Tumor necrosis factor alpha
TSH	Thyroid stimulating hormone
ULN	Upper limit of normal
USA	United States of America
WFI	Water for injection
WHO	World Health Organization

1. INTRODUCTION

Patients with HPV/p16-negative head and neck cancer (HNSCC) and heavy smoking history constitute a high-risk population with poor survival outcomes and dreadful morbidity (Ang et al., 2014). Transcriptome analysis examining the immune infiltrative landscape of HNSCC has shown that tumors harboring genetic smoking signatures had lower immune infiltration and were associated with poorer survival, suggesting these patients may benefit from immune agonist therapy (Mandal et al., 2016). The data emerging pre-clinically implicates short course RT is a powerful “firestarter” that upregulates PDL-1 expression and leads to enhanced immunogenicity when combined with anti-PD1/PDL1 inhibitors. In this trial, we propose to give radiation preoperatively in combination with anti-PDL1 immunotherapy to prime the immune system and improve the chances of a successful surgery and overall survival.

1.1 Disease background

1.1.1 Immunotherapies

It is increasingly understood that cancers are recognized by the immune system, and, under some circumstances, the immune system may control or even eliminate tumors (Dunn et al., 2004). PD-L1 is a member of the B7 family of ligands that inhibit T-cell activity through binding to the PD-1 receptor (Keir, Butte, Freeman, & Sharpe, 2008) and to CD80 (Butte, Keir, Phamduy, Freeman, & Sharpe, 2007). PD-L1 expression is an adaptive response that helps tumors evade detection and elimination by the immune system. Expression of PD-L1 protein

is induced by inflammatory signals that are typically associated with an adaptive immune response (e.g., IFNγ) and can be found on both tumor cells (TC) and tumor-infiltrating IC. The binding of PD-L1 to PD-1 on activated T cells delivers an inhibitory signal to the T cells, preventing them from killing target TC and protecting the tumor from immune elimination (Zou & Chen, 2008). PD-L1 may also inhibit T cells through binding to CD80, although the exact mechanism is still not elucidated (Butte et al., 2007; Paterson et al., 2011).

The inhibitory mechanism described above is co-opted by tumors that express PD-L1 as a way of evading immune detection and elimination. The binding of an anti–PD-L1 agent to the PD-L1 receptor inhibits the interaction of PD-L1 with the PD-1 and CD80 receptors expressed on immune cells. This activity overcomes PD-L1–mediated inhibition of antitumor immunity. While functional blockade of PD-L1 results in T-cell reactivation, this mechanism of action is different from direct agonism of a stimulatory receptor such as CD28. In vivo studies have shown that durvalumab inhibits tumor growth in xenograft models via a T cell–dependent mechanism (Stewart et al., 2015).

PD-L1 is expressed in a broad range of cancers. Based on these findings, an anti–PD-L1 antibody could be used therapeutically to enhance antitumor immune responses in patients with cancer. Results of non-clinical and clinical studies of monoclonal antibodies (mAbs) targeting the PD-L1/PD-1 pathway have shown evidence of clinical activity and a manageable safety profile, supporting the hypothesis that an anti–PD-L1 antibody could be used to therapeutically enhance antitumor immune response in cancer patients (Brahmer et al., 2012; Hirano et al., 2005; Iwai et al., 2002; Okudaira et al., 2009; Topalian et al., 2012; C. Zhang et al., 2008) with responses that tend to be more pronounced in patients with tumors that express PD-L1 (Powles et al., 2014; Rizvi et al., 2015; Segal et al., 2015). In addition, high mutational burden (e.g., in bladder carcinoma (Alexandrov et al., 2013)) may contribute to the responses seen with immune therapy.

In contrast, cytotoxic T-lymphocyte-associated antigen-4 (CTLA-4) is constitutively expressed by regulatory T cells and upregulated on activated T cells. CTLA-4 delivers a negative regulatory signal to T cells upon binding of CD80 (B7.1) or CD86 (B7.2) ligands on antigen-presenting cells (Fife & Bluestone, 2008). Blockade of CTLA-4 binding to CD80/86 by anti–CTLA-4 antibodies results in markedly enhanced T-cell activation and antitumor activity in animal models, including killing of established murine solid tumors and induction of protective antitumor immunity. Therefore, it is expected that treatment with an anti–CTLA-4 antibody will lead to increased activation of the human immune system, increasing antitumor activity in patients with solid tumors.

Pre-clinical data have now been added to with a wealth of clinical data showing that blockade of negative regulatory signals to T-cells such as cytotoxic T-lymphocyte antigen 4 (CTLA-4) and programmed death ligand 1 (PD-L1) has promising clinical activity. Ipilimumab was granted United States (US) Food and Drug Administration (FDA) approval for the treatment of metastatic melanoma and is currently under investigation for several other malignancies, whilst nivolumab and pembrolizumab, two anti–PD-1 agents, and atezolizumab, an anti–PD-L1, agent have been granted approvals by agencies such as the US FDA and the European Medicines Agency approval for the treatment of a number of malignancies including metastatic melanoma, squamous and non-squamous cell non-small-cell lung cancer and urothelial carcinoma. In addition, there are data from agents in the anti–PD-1/PD-L1class showing clinical activity in a wide range of tumor types.

1.2 Durvalumab background/non-clinical and clinical experience

The non-clinical and clinical experience is fully described in the most current version of the durvalumab Investigator's Brochure. Durvalumab is a human monoclonal antibody (mAb) of the immunoglobulin G (IgG) 1 kappa subclass that inhibits binding of PD-L1 and is being developed by AstraZeneca/MedImmune for use in the treatment of cancer (MedImmune is a wholly owned subsidiary of AstraZeneca; AstraZeneca/MedImmune will be referred to as AstraZeneca throughout this document). The proposed mechanism of action (MOA) for durvalumab is interference in the interaction of PD-L1 with PD-1 and CD80 (B7.1). Blockade of PD-L1/PD-1 and PD-L1/CD80 interactions releases the inhibition of immune responses, including those that may result in tumor elimination. *In vitro* studies demonstrate that durvalumab antagonizes the inhibitory effect of PD-L1 on primary human T cells resulting in the restored proliferation of IFN-γ (Stewart et al 2015). *In vivo* studies have shown that durvalumab inhibits tumor growth in xenograft models via a T-cell-dependent mechanism (Stewart et al 2015). Based on these data, durvalumab is expected to stimulate the patient's antitumor immune response by binding to PD-L1 and shifting the balance toward an antitumor response. Durvalumab has been engineered to reduce antibody-dependent cellular cytotoxicity and complement-dependent cytotoxicity.

To date durvalumab has been given to more than 6000 patients as part of ongoing studies either as monotherapy or in combination with other anti-cancer agents. Details on the safety profile of durvalumab monotherapy are summarized in Section 0 and Section 6.4. Refer to the current durvalumab Investigator's Brochure for a complete summary of non-clinical and clinical information including safety, efficacy and pharmacokinetics.

There are approximately 81 currently ongoing trials combining radiation and immunotherapy (Johnson & Jagsi, 2016). Many of them have shown safety of combining PD1 or PDL-1 in combination with radiation or chemoradiation, both adjuvant and neoadjuvantly, or definitively. The Phase I safety trial mentioned above (NCT02296684) for surgically resectable HNSCCs with pembrolizumab neoadjuvantly followed by adjuvant pembrolizumab, the Phase 1 safety trial has been reported to be safe and has moved to a Phase III with no DLTs encountered either in the neoadjuvant or adjuvant phase (Uppaluri et al., 2016). An FDA approved trial incorporating multimodality therapy with induction carboplatin/Nab-Paclitaxel and Durvalumab followed by surgical resection and risk-adapted adjuvant therapy for the treatment of locally-advanced and surgically resectable squamous cell carcinoma of the head and neck (NCT03174275) is currently ongoing.

Another ongoing trial in high risk oral cavity administers Nivolumab With or Without Ipilimumab neoadjuvantly in this population (NCT02919683). Personal communication with the Study PI, Dr. Jonathan Schoenfeld has relayed no toxicity since its opening in September of 2016. A current ongoing trial through the NRG is HN003, which incorporates adjuvant pembrolizumab and chemoradiation and currently completed safety check at interim analysis. Personal communication with the principal investigator, Dr. Julie Bauman, relayed that in the cohort of 12 patients had 1 DLT (unacceptable # was >3), the expansion cohort is now open, and the MTD was Pembrolizumab 200 mg IV starting one week before CRT then q 3 weeks for a total of 8 doses. Adjuvant CRT consists of cisplatinum 40 mg/m2/week and 6 weeks conventional fractionated radiation with two doses of pembrolizumab overlap with CRT. This is full dose and schedule, and no de-escalation was necessary.

In the definitive management of head and neck cancer, combination of Pembrolizumab with weekly cisplatinum is safe in a Phase I clinical trial recently presented (NCT02586207, (Powell

et al., 2017)). Combination hypofractionated radiation and anti-PD1 therapy has also been shown to be safe and effective (NCT02383212, (Papadopoulos et al., 2016). Finally, Durvalumab is being combined in multiple clinical trials neoadjuvantly and adjuvantly and in combination with radiation in a similar manner to our trial in different cancer subsites. A neoadjuvant trial combining anti-PD-L1 (Durvalumab/MEDI4736) plus anti-CTLA-4 (Tremelimumab) and radiation is currently ongoing in high risk soft-tissue sarcoma (NCT03116529). In Stage IIIA resectable non-small cell lung cancer, durvalumab is being combined with radiation neoadjuvantly in a Phase 2 clinical trial (NCT03237377). In Phase Ib/II study for esophageal and gastroesophageal junction adenocarcinoma durvalumab (MEDI4736) and chemoradiation carboplatin AUC 2/paclitaxel (NCT02962063). This study has been open for one year. Finally, a Phase Ib/II study of concurrent Durvalumab and radiation therapy followed by adjuvant Durvalumab in patients with urothelial cancer (T2-4 N0-2 M0) of the bladder is currently ongoing (NCT02891161). The trial has been open for over a year and was last updated in September, 2017 with no significant issues.

In terms of the feasibility of combining Durvalumab with Cetuximab-radiotherapy, a currently ongoing Phase 1-II trial is currently ongoing where adjuvant Durvalumab is given concurrently with Cetuximab-radiation and for 8 months adjuvantly for locally advanced HNSCCs (NCT03051906). There have been no major safety issues reported to date.

1.3 Research hypothesis

Combination of neoadjuvant durvalumab plus hypofractionated radiation plus postoperative adjuvant durvalumab in surgically resectable stage II-IVB oral cavity, stage III-IVB larynx and hypopharynx, or stage III-IVB HPV and/or p16 negative intermediate-high risk oropharynx head and neck cancer is safe and tolerable.

1.4 Rationale for conducting this study

Head and neck cancer is a disease that is known to have poor survival outcomes and response to combination chemoradiation (Ang et al., 2014). This disease is also characterized by significantly lower levels of PD-L1 expression (Concha-Benavente et al., 2016), poor tumoral infiltration of T-cells (Mandal et al., 2016; Oweida et al., 2017), and low response to immune checkpoint blockade pre-clinically and in recent clinical trials (Cohen, 2017). Our data have shown that RT overrides innate resistance to PDL1 inhibitors in orthotopic models of high risk HNSCC (Oweida et al., 2017). These data are concordant with the results of the recently publicized PACIFIC trial of administering anti-PDL1 following RT therapy in lung cancer (Antonia et al., 2017). Many clinical trials are following suit adding checkpoint inhibitors to RT either concurrently or adjuvantly (Kang, Demaria, & Formenti, 2016).

1.4.1 Durvalumab monotherapy dose rationale

A durvalumab dose of 1500 mg Q4W is supported by in vitro data, non-clinical activity, clinical PK/pharmacodynamics, biomarkers, and activity data from Study 1108 in patients with advanced solid tumors and from a Phase I trial performed in Japanese patients with advanced solid tumor (D4190C00002).

1.4.1.1 PK/Pharmacodynamic data

Based on available PK/pharmacodynamic data from ongoing Study 1108 with doses ranging from 0.1 to 10 mg/kg Q2W or 15 mg/kg Q3W, durvalumab exhibited non-linear (dose dependent) PK consistent with target-mediated drug disposition. The PK approached linearity

at ≥3 mg/kg Q2W, suggesting near complete target saturation (membrane-bound and sPD-L1), and further shows that the durvalumab dosing frequency can be adapted to a particular regimen given the linearity seen at doses higher than 3 mg/kg. The expected half-life with doses ≥3 mg/kg Q2W is approximately 21 days. A dose-dependent suppression in peripheral sPD-L1 was observed over the dose range studied, consistent with engagement of durvalumab with PD-L1. A low level of immunogenicity has been observed. No patients have experienced immune-complex disease following exposure to durvalumab (for further information on immunogenicity, please see the current IB).

Data from Study D4190C00006 (Phase I trial in NSCLC patients using the combination of durvalumab and tremelimumab) also show an approximately dose-proportional increase in PK exposure for durvalumab over the dose range of 3 to 20 mg/kg durvalumab Q4W or Q2W (for further information on PK observations in Study 006, please see the current IB). The observed durvalumab PK data from the combination study were well in line with the predicted monotherapy PK data (5th median and 95th percentiles) for a Q4W regimen. A population PK model was developed using the data from Study 1108 (doses=0.1 to 10 mg/kg Q2W or 15 mg/kg Q3W (Fairman et al., 2014). Multiple simulations indicate that a similar overall exposure is expected following both 10 mg/kg Q2W and 20 mg/kg Q4W regimens, as represented by AUC_{ss} (4 weeks). Median $C_{max,ss}$ is expected to be higher with 20 mg/kg Q4W (~1.5 fold) and median $C_{trough,}$as is expected to be higher with 10 mg/kg Q2W (~1.25 fold). Clinical activity with the 20 mg/kg Q4W dosing regimen is anticipated to be consistent with 10 mg/kg Q2W with the proposed similar dose of 20 mg/kg Q4W expected to (a) achieve complete target saturation in majority of patients; (b) account for anticipated variability in PK, pharmacodynamics, and clinical activity in diverse cancer populations; (c) maintain sufficient PK exposure in case of ADA impact; and (d) achieve PK exposure that yielded maximal antitumor activity in animal models.

Given the similar area under the plasma drug concentration-time curve (AUC) and modest differences in median peak and trough levels at steady state, the observation that both regimens maintain complete sPDL1 suppression at trough, and the available clinical data, the 20 mg/kg Q4W and 10 mg/kg Q2W regimens are expected to have similar efficacy and safety profiles, supporting further development with a dose of 20 mg/kg Q4W.

1.4.1.2 Clinical data

Refer to the current durvalumab Investigator's Brochure for a complete summary of clinical information including safety, efficacy and pharmacokinetics at the 20mg/kg Q4W regimen.

1.4.2 Rationale for fixed dosing

A population PK model was developed for durvalumab using monotherapy data from a Phase I study (study 1108; N=292; doses= 0.1 to 10 mg/kg Q2W or 15 mg/kg Q3W; solid tumors). Population PK analysis indicated only minor impact of body weight (WT) on the PK of durvalumab (coefficient of ≤0.5). The impact of body WT-based (10 mg/kg Q2W) and fixed dosing (750 mg Q2W) of durvalumab was evaluated by comparing predicted steady state PK concentrations (5th, median and 95th percentiles) using the population PK model. A fixed dose of 750 mg was selected to approximate 10 mg/kg (based on median body WT of ~75 kg). A total of 1000 patients were simulated using body WT distribution of 40–120 kg. Simulation results demonstrate that body WT-based and fixed dosing regimens yield similar median steady state PK concentrations with slightly less overall between-patient variability with fixed dosing regimen.

Similar findings have been reported by others (Narwal, Roskos, & Robbie, 2013; Ng, Lum, Gimenez, Kelsey, & Allison, 2006; D. D. Wang, Zhang, Zhao, Men, & Parivar, 2009; S. Zhang, Shi, Li, Parivar, & Wang, 2012). Wang and colleagues investigated 12 monoclonal antibodies and found that fixed and body size-based dosing perform similarly, with fixed dosing being better for 7 of 12 antibodies (D. D. Wang et al., 2009)]. In addition, they investigated 18 therapeutic proteins and peptides and showed that fixed dosing performed better for 12 of 18 in terms of reducing the between-patient variability in pharmacokinetic/pharmacodynamics parameters (S. Zhang et al., 2012).

A fixed dosing approach is preferred by the prescribing community due to ease of use and reduced dosing errors. Given expectation of similar pharmacokinetic exposure and variability, we considered it feasible to switch to fixed dosing regimens. Based on average body WT of 75 kg, a fixed dose of 1500 mg Q4W durvalumab (equivalent to 20 mg/kg Q4W) is included in the current study.

1.4.3 Rationale for hypofractionated radiation

We and others have shown that concurrent administration of anti-PDL1 with RT can significantly reduce tumor growth compared to either modality alone (Dovedi & Illidge, 2015; Oweida et al., 2017); however, the optimal RT dose and fractionation remain unknown. In the clinic, RT is delivered in multiple daily fractions of 1.8–2 Gy. This fractionation, however, can cause severe lymphopenia (Campian, Sarai, Ye, Marur, & Grossman, 2014; Verastegui, Morales, Barrera-Franco, Poitevin, & Hadden, 2003) as T-lymphocytes are radiosensitive (Manda, Glasow, Paape, & Hildebrandt, 2012), and would, therefore, eliminate any benefit from PD-L1 blockade. In contrast, hypofractionated RT involves the delivery of large doses of RT in a few fractions (1-5 fractions), providing sufficient time between doses for repopulation of T-lymphocytes, and therefore, providing an effective response to PD-L1 blockade. There is considerable variability in the literature on what constitutes a meaningful immune-stimulating dose/fractionation (Demaria, Coleman, & Formenti, 2016; Monjazeb & Schoenfeld, 2016; Young et al., 2016). Certain hypofractionated doses/fractionation have been shown to induce infiltration of immunosuppressive Tregs, MDSCs, and tumor-associated macrophages (TAMs), which can contribute to tumor progression and resistance to checkpoint blockade. A preclinical study investigating various RT schedules found that high single-doses (20Gy and 30Gy) were less immunogenic than repeated doses of 8Gy, which lead to significant improvement in tumor control when combined with anti CTLA-4 therapy (Vanpouille-Box et al., 2017). Gene-expression analysis revealed differential expression of IFN-I stimulated genes (ISGs), which were upregulated by 8Gy single and repeated doses but not by higher single doses. Inducing a highly immunogenic response with RT and mitigating the influx of immunosuppressive cells should lead to a durable tumor response.

Our data show that a 10Gy single dose of RT with anti-PDL1 leads to only a transient response in tumor growth, expression of PD-L1 and secretion of CXCL9/10 (Oweida et al., 2017). These data suggest that adaptive resistance by tumor cells can override the immune-modulatory benefits observed with single-dose RT. Using the same animal model, we have recently shown that hypofractionated RT delivered concurrently with anti-PDL1 overrides such adaptive tumor resistance in a manner that no combination checkpoint inhibition can (unpublished data, manuscript in preparation). We therefore propose to administer hypofractionated radiotherapy to the gross disease only neoadjuvantly. The proposed dose is still low at 18 Gy delivered in 3 fractions of 6 Gy each and will serve the function of enhanced antigen presentation and T-cell intratumoral infiltration.

Published data has shown that low dose neoadjuvant radiation (20Gy in 10 fractions) given concurrently with low dose cisplatinum followed by surgical resection approximately 3-6 weeks later and postoperative radiation to 50 or 60Gy based on risk has been shown to be safe and yields significant improvement in overall survival (Mücke et al., 2011). A reasonable concern is whether preoperative RT will be a significant factor in head and neck surgery and reconstruction. Lin et al., 2005, has shown that microvascular flap reconstruction can be accomplished in both irradiated and non-irradiated head and neck cancer patients, with a 99% total flap survival rate (Lin et al., 2005; Simon et al., 2011). In a Phase 2 clinical trial, Harada et al., 2013, administered 40 Gy preoperative chemotherapy preoperatively for oral cavity squamous cell carcinoma with a median interval between end of chemoradiotherapy and surgery was 22.5 days (range 13–36 days). This was shown to be a tolerable and effective approach with no serious adverse effects at 3 years (Harada et al., 2013). Finally, the design of this trial is similar to what has been proposed and approved by the FDA in a Phase I/II clinical trial testing in the HPV positive HNSCC setting (ESR 16-12538, PI: Robert Chin).

1.4.4 Rationale for neoadjuvant studies

When immune checkpoint inhibitors are administered in the setting of previously pre-treated cancer, often multifactorial resistance has developed and the tumors are less likely to respond to new therapeutics. A good example of that is the pivotal phase 3 KEYNOTE-040 trial investigating pembrolizumab anti-PD-1 therapy, in previously treated patients with recurrent or metastatic HNSCC, which did not meet its pre-specified primary endpoint of overall survival (OS) (HR, 0.82 [95% CI, 0.67-1.01]; p = 0.03). Additionally, neoadjuvant studies afford us with the ability of advancing translational research as it avoids the safety and ethical concerns of obtaining sequential tumor biopsies. By obtaining tissue before and after treatment with radiation-anti-PDL1 will allow us to analyze the early biological effect and assess predictive molecular markers of those that are likely to respond from such therapy. Finally, the availability of tissue from both the tumor and its nodal metastases will allow us to examine the differential effects of immune response in these two compartments.

1.4.5 Rationale for concurrent and adjuvant treatment

Clinical data indicate that anti–PD-1/anti-PD-L1 agents can effectively achieve disease stabilization and durable responses in HNSCC (Badoual et al., 2013; Badoual et al., 2010; Larkin et al., 2015; Lyford-Pike et al., 2013). Efficacious interventions to initiate and sustain an immune response will likely require a number of treatment sessions and possibly different agents that harness different immune mechanisms to successfully mount an immune response. The ideal schedule and optimal duration of maintenance therapy are unknown in the front-line treatment setting, but evidence supports a maintenance phase to prolong immune memory development.

A recent clinical trial using anti-PD1 therapy, pembrolizumab, in the neoadjuvant and adjuvant setting showed antitumor activity in locally advanced HPV-negative HNSCC (NCT02296684). The anti-PD1 was also given in concurrently with standard of care therapy and adjuvantly as maintenance therapy. Not only was it shown to be safe with no adverse events, but there were no local or distant failures at 1 year. Importantly, only 38% of the patients had high risk pathologic features out of the expected 80%, 43% had a pathologic response rate to neoadjuvant pembrolizumab, and 48% had clinical to pathological downstaging. These results are highly promising in this patient population known to have poor outcomes (Leeman et al., 2017). As detailed above, there are multiple ongoing studies combining systemic therapy and radiation that have to date not reported any concerning safety signals. While adjuvant immune therapy is not currently the standard of care, it is anticipated that the proposed maintenance

durvalumab will improve treatment response without adding significant adverse events based on prior studies.

1.4.6 Study population rationale

Approximately 70% of newly diagnosed SCCHN cancer patients will be diagnosed with locally advanced or local disease that is amenable to therapy with radiation, surgery, chemotherapy, or combination of the three. This study will enroll patients with newly diagnosed, resectable, locally advanced (stage II-IVB oral cavity, stage III-IVB larynx and hypopharynx, or stage III-IVB HPV/p16 negative intermediate-high risk oropharynx according to the 8th Edition of the AJCC/UICC Staging System) HNSCC. Survival rates with multimodality treatment with curative intent for locally advanced disease are still poor with 5-year survival rates of 30% to 40% (Siegel, Ma, Zou, & Jemal, 2014). Most of these patients eventually relapse with either locoregional recurrence, metastatic disease (20% to 40% of patients), or both (Vermorken & Specenier, 2010). Patients that are p16 negative (the preferred surrogate testing for HPV tumor status) have significantly worse outcomes than those that are p16 positive with nearly half the five year survival rates (Leeman, Li, Pei, 2017). An unmet medical need exists for this patient population.

1.5 Benefit/risk and ethical assessment

1.5.1 Potential benefits

The potential benefits include decreased surgical morbidity and improved response rates, both local and distant. Benefits might also include reduced need for adjuvant therapy if the response is improved.

1.5.2 Overall risks

Monoclonal antibodies directed against immune checkpoint proteins, such as programmed cell death ligand 1 (PD-L1) as well as those directed against programmed cell death-1 (PD-1) or cytotoxic T-lymphocyte antigen-4 (CTLA-4), aim to boost endogenous immune responses directed against tumor cells. By stimulating the immune system however, there is the potential for adverse effects on other tissues.

Most adverse drug reactions seen with the immune checkpoint inhibitor class of agents are thought to be due to the effects of inflammatory cells on specific tissues. These risks are generally events with a potential inflammatory or immune mediated mechanism and which may require more frequent monitoring and/or unique interventions such as immunosuppressants and/or endocrine therapy. These immune mediated effects can occur in nearly any organ system, and are most commonly seen as gastrointestinal AEs such as colitis and diarrhea, pneumonitis/interstitial lung disease (ILD), hepatic AEs such as hepatitis and liver enzyme elevations, skin events such as rash and dermatitis and endocrinopathies including hypo- and hyper-thyroidism.

1.5.2.1 Durvalumab

Risks with durvalumab include, but are not limited to, diarrhea/colitis and intestinal perforation, pneumonitis/ILD, endocrinopathies (hypo- and hyper-thyroidism, type I diabetes mellitus, hypophysitis and adrenal insufficiency) hepatitis/increases in transaminases, nephritis/increases in creatinine, pancreatitis/increases in amylase and lipase, rash/pruritus/dermatitis, myocarditis, myositis/polymyositis, other rare or less frequent

inflammatory events including neurotoxicities, infusion-related reactions, hypersensitivity reactions and infections/serious infections.

For information on all identified and potential risks with durvalumab please always refer to the current version of the durvalumab IB.

1.5.2.2 Overall benefit-risk

Currently available data supports the potential benefit of adding anti-PDL1 in the neoadjuvant setting alone (NCT02383212) and in combination with radiation and chemotherapy either adjuvantly (NCT02383212) or definitively (Antonia et al., 2017). Once started as part of the window study, patients are eligible to adjuvant treatment with the previously used anti-PDL1 to further benefit from the immunotherapy, potentially improving survival. We anticipate seeing similar neoadjuvant benefit derived from adding anti-PD1 therapy as in the Merck trial (NCT02296684).

There is a general sense that the selection of patients based on PD-L1 expression levels within the tumor microenvironment may improve the probability and/or quality of responses to PD-1 pathway-targeting agents and, therefore, may have merit as a patient enrichment tool. Data from studies with durvalumab and other agents targeting the PD-1/PD-L1 pathway suggest, as shown in a number of tumor types including HNSCCs (eg, NSCLC, renal cell carcinoma, and melanoma), that treatment with monotherapy may be associated with a higher ORR in patients who are PD-L1-positive (Segal, et al., 2015). We and others, however, have shown that one key mechanism by which radiation works is by increasing PDL1 expression (Deng et al., 2014; Stokes et al., 2017). This allows for an enhanced synergistic effect between RT and anti-PDL1 and makes the response less dependent on existing levels of baseline PDL1 expression, which tends to be low in the HPV-negative population (Mandal et al., 2016).

Some of the risks involved here might include risk of developing significant normal tissue toxicity delaying the surgical intervention. We have chosen a low dose regimen with a dose de-escalation strategy built in. It is important to note that in rectal cancer, neoadjuvant radiation with a higher dose radiation of 5 Gy delivered in 5 consecutive fractions is a standard of care guideline. We will follow strict dose constraint guidelines and given that the preoperative radiation will be given to the gross disease, and the gross tumor tissue will be surgically resected and replaced with fresh tissue, the risk of giving adjuvant radiation per guidelines (if needed) should be minimized.

2. PROVIDE SUMMARY OF OVERALL BENEFIT-RISK FOR THE STUDY OBJECTIVES

The benefits of this trial include improved resection with surgery, improved pathologic response rate, and improved progression free survival both locally and distantly. The risks involve that of surgical delay due to development of side effect to neoadjuvant anti-PDL1 radiation.

2.1 Primary objective(s)

Determine Maximum Tolerated Dose (MTD) and tolerability of combination of durvalumab + RT + postoperative adjuvant durvalumab in surgically resectable stage II-IVB oral cavity, stage III-IVB larynx and hypopharynx, or stage III-IVB HPV and/or p16 negative intermediate-high risk oropharynx head and neck cancer.

2.2 Secondary objective(s)

1. Determine pathologic response (in diagnostic biopsy and surgical specimens) to durvalumab + RT prior to surgery.
2. Determine clinical response (via imaging and clinical exam) at about 3 months from the end of adjuvant radiation (if given), or for those that do not receive adjuvant radiation, at about 3 months from the end of Cycle 2 adjuvant durvalumab. This includes locoregional control, distant control, and overall survival.
3. Evaluate biomarkers of immune response to treatment protocol in tumor and blood samples (including circulating tumor cells).
4. Assess incidence of adverse pathologic features that constitute indications for adjuvant therapy.
5. Define toxicity profile associated with treatment protocol.
6. Assess short-term quality-of-life associated with treatment protocol.

2.3 Exploratory objective(s)

Translational studies will require blood samples, initial biopsy, and tumor tissue at the time of surgery.

1. To establish a gene signature panel that can assess the response to RT-anti-PDL1 response and treatment outcome.
2. Compare change in anti-PDL1 and T cell infiltration pattern and intensity before and after RT and anti-PDL1 by immunohistochemistry.
3. To evaluate the impact of other mutations on immune response and treatment outcome.
4. Blood samples to evaluate biomarkers of immune response as well as for evaluation of circulating auto-antibodies. Blood will be collected prior to treatment, at time of surgery, at end of treatment and at the 6 month follow-up.
5. Tumor tissue taken at the time of initial biopsy and at time of resection will be profiled for tumor infiltrating lymphocytes; activation markers and antigen specific TCR utilization/diversity will be evaluated for additional checkpoint targets.
6. On long-term follow-up, tumor antigen specific T lymphocyte memory populations will be monitored for representation and robustness in in-vitro stimulation assays as potential biomarker of continued anti-tumor activity.

3. STUDY DESIGN

3.1 Overview of study design

3.1.1 Overview

This is a multi-center, prospective, single-arm phase I/Ib safety trial. Patients in this study will undergo SBRT with durvalumab neoadjuvantly followed by surgery and adjuvant durvalumab +/- adjuvant radiation and chemotherapy to ensure general safety of this combination. Phase I will follow the traditional 3+3 model. Patients eligible for this study must be diagnosed with non-metastatic, biopsy-proven squamous cell carcinoma of the oral cavity, oropharynx, larynx, or hypopharynx, and must be eligible for surgery. Eligible oropharynx cancers must be p16 negative. P16 testing is only required for cancers of oropharyngeal primary.

3.1.2 Neoadjuvant Therapy

All eligible patients will receive Durvalumab 1500 mg IV approximately 3-6 weeks prior to planned surgical resection. The first dose of durvalumab can be administered at any time between SBRT fractions 1-3. SBRT will be given as 6 Gy according to their assigned dose level given every other day over approximately one week to sites of gross disease. Please note that the SBRT can be planned as an IMRT plan.

3.1.3 Adjuvant Therapy

Adjuvant chemotherapy and radiation therapy will be dictated by surgical pathology and NCCN guidelines. If indicated, treatment will consist of intensity modulated radiation therapy of 60 Gy in 2 Gy once daily fractions (total of 30 fractions). Risk based chemotherapy (cisplatin or carboplatin based cytotoxic chemotherapy) or targeted biologics (cetuximab) will be given per investigator discretion. Adjuvant radiation +/- chemotherapy should be given 6-12 weeks after surgery.

Adjuvant Durvalumab will be given regardless of post-operative risk status. For patients receiving adjuvant radiation ± chemotherapy, the first dose of Durvalumab will be given on the same start date +/-7 days as radiation. For patients not requiring adjuvant radiation therapy, Durvalumab will start 6-12 weeks after surgery. Durvalumab in both instances will be given as 1500 mg intravenously once every 4 weeks for a maximum of 6 doses.

3.1.4 DLT Assessment

Initially 3 patients will be enrolled to receive neoadjuvant durvalumab with 2 fractions of 6Gy SBRT. DLTs are defined in section 6.4. If there are no DLTs or delay of surgery for greater than 8 weeks post neoadjuvant therapy for medical reasons, the study will continue with enrollment of 3 more patients on this schedule. If 1 DLT is established related to neoadjuvant therapy or surgery is delayed > 8 weeks for medical reasons, the radiation schedule will be decreased to 6 Gy for 1 fraction. If this is not tolerated the study will be halted. Durvalumab will not be dose decreased. Completed and ongoing studies (including the PACIFIC study and other mentioned in section 1.2) have shown good tolerance and no DLTs of adjuvant durvalumab as monotherapy and with concurrent chemo-radiation in various cancer types. Similarly, HN003 has shown that adjuvant anti-PD1 with concurrent chemoradiation and as maintenance afterwards, resulted in no adverse side effects (data presented at the NRG 2018 meeting). Therefore, we do not expect a DLT to occur during the adjuvant treatment period but will continue to record AEs as specified in the Schedule of Study Assessments.

3.2 Sample size and power considerations

Part 1: Dose Escalation

The expectation is that 9 patients will be enrolled to the trial during part 1. This is based on the expectation that all dose levels are safe (i.e. patients will not experience DLTs at all dose levels). In this case, 3 patients will be enrolled at dose level 2 (0/3 DLTs), then dose escalating to dose level 3 and enrolling a total of 6 patients at this dose level, for a total of 9 patients. The range of patients needed will be 6–12 patients.

Part 2: Dose Expansion

The primary outcome of this study is to assess safety. An additional 8-10 patients will be enrolled at the DLT dosing determined during part 1 for further safety assessment. This number was chosen to answer our secondary outcome of efficacy as measured by pathologic response and cytotoxic CD8 T cell infiltration. This is determined by binary measure of whether or not a

patient has relevant CD8 infiltration, defined as a 1.5-fold increase at tumor resection as compared to biopsy. Let p represent the proportion of patients with relevant CD8 infiltration. It is expected that half (50%) of patients not receiving this treatment will have relevant CD8 infiltration. The objective is to test the following hypotheses:

H_0: $p \leq 0.50$
H_1: $p \geq 0.80$

The hypotheses will be tested using the exact binomial test. A total of 14-17 patients are needed to have at least 80% power (87.0% to be exact) to discriminate between the hypotheses stated above when testing them using a 1-sided superiority test and controlling the type 1 error rate at 0.10.

Total Sample Size

During part 1, 6 patients will be treated at the MTD. The CD8 infiltration will be assessed on these 6, leaving an additional 8 subjects to be enrolled during part 2 in order to achieve the total sample size of 14 needed for part 2. The total sample size is thus expected to be 17 patients (9 from part 1 + 8 from part 2). As the total sample size will depend on the number of patients needed during part 1 of the trial. The total sample size would be 20.

3.3 Definition of analysis populations

3.3.1 Full analysis set (FAS)

The statistical analysis of the efficacy will include all enrolled patients (i.e., the Intent-to-Treat [ITT] population) and will classify them based on enrolled treatment, regardless of the treatment actually received. Patients who were enrolled but did not receive study treatment are included in the ITT population.

3.3.2 Evaluable analysis set (EAS)

A subset of the full analysis set (FAS), the evaluable analysis set is defined as those treated patients who are evaluable for the primary endpoint.

3.3.3 Safety analysis set (SAS)

All patients who received surgery, radiation therapy and at least 1 dose of durvalumab and for whom any valid post-baseline safety data are available will be included in the safety analysis set. When assessing safety and tolerability, summaries will be produced based on the safety analysis set. Patients are analyzed according to the actual treatment received.

Each subject will be observed for the occurrence of a DLT from the day on which the patient starts neoadjuvant durvalumab at 1500 mg up until the day of surgery. The expected duration of this observation period is approximately 3–6 weeks.

The theoretical advantages of preoperative chemoradiotherapy are downstaging of the primary tumor, increased resectability rate, and the elimination of micrometastasis. The preoperative chemoradiotherapy protocol with a radiation dose of 40 Gy might offer several therapeutic and prognostic advantages. Limiting the dose to 40 Gy before surgery has been shown to reduce the overall radiation dose for the patient and the organ-specific dose for salivary glands, facilitating the preservation of salivary gland function and resulting in less post-therapeutic xerostomia (Jellema, Doornaert, Slotman, Rene Leemans, & Langendijk, 2005; Jen, Lin,

Wang, & Wu, 2006)(Jen, Lin, Wang, & Wu, 2006)(Glanzmann & Grätz; Mendenhall, 2004) (Schultze-Mosgau et al., 2002)(Glanzmann & Grätz Furthermore, the risk of wound-healing disorders, which result from decreased vascularization of the pre-irritated recipient tissue after reconstructive surgery with free-flap transfers, is significantly reduced with a radiation dose of 40 Gy compared to radiation doses[60 Gy (Schultze-Mosgau et al., 2002). This procedure with 40 Gy appears likely to reduce the incidence of osteoradionecrosis. Furthermore, the risk of wound-healing disorders, which result from decreased vascularization of the pre-irritated recipient tissue after reconstructive surgery with free-flap transfers, is significantly reduced with a radiation dose of 40 Gy compared to radiation dose of 60 Gy.

The primary endpoint of the safety lead-in is to determine the safety and tolerability. Toxicity will be assessed by CTCAE v. 4.03 criteria. Data from all cycles of treatment will be combined in the presentation of safety data. AEs will be listed individually by patient.

Any AE occurring before treatment with investigational product (IP) will be included in the data listings but will not be included in the summary tables of AEs. Any AEs occurring within 90 days of discontinuation of IP may be included in the AE summaries, but the majority of those summaries will omit the AEs observed after a patient has received further therapy for cancer. Patients will be classified on the basis of the treatment actually received using CTCAE v 4.03 criteria. A toxicity will only be considered a DLT if it delays surgery more than 8 weeks.

3.4 Patient disposition and characteristics

A detailed description of patient disposition will be provided. It will include a summary of the number and percentage of patients entered into the study, enrolled in the study, and treated, as well as number and percentage of patients completing the study, or discontinuing (overall and by reason for discontinuation).

Patient demographic and baseline characteristics will be summarized using descriptive statistics. For continuous variables, descriptive statistics (n, mean, standard deviation, standard error, median, minimum, and maximum) will be provided. For categorical variables, patient counts and percentages will be provided. Categories for missing data will be presented if necessary. The categorical variables such as gender and race will be summarized using descriptive statistics for each variable category. These p-values are for descriptive purposes only, to aid in the assessment of baseline comparability. Therefore, they are not adjusted for multiplicity.

3.5 Planned Methods of Analysis

3.5.1 Analysis of primary endpoint for safety

Primary Endpoint

For part 1 of the study, the primary endpoint will be the binary outcome of whether or not a patient experiences the occurrence of a dose-limiting toxicity (DLT), where the occurrence of a DLTs are defined in section 6.4. AEs will be listed individually by patient. Additional patients will be enrolled at the DLT dosing determined during part 1 for further safety assessment. This number was chosen to answer our secondary outcome of efficacy as measured by pathologic response and cytotoxic CD8 T cell infiltration. This is determined by the binary measure of whether or not a patient has relevant CD8 infiltration, defined as a 1.5-fold increase at tumor resection as compared to biopsy.

Any AE occurring before treatment with investigational product (IP) will be included in the data listings but will not be included in the summary tables of AEs. Any AEs occurring within 90 days of discontinuation of IP may be included in the AE summaries, but the majority of those summaries will omit the AEs observed after a patient has received further therapy for cancer. Patients will be classified on the basis of the treatment actually received using CTCAE v 4.03 criteria.

For part 2 of the trial, the primary endpoint will be the binary measure of whether or not a patient has relevant CD8 infiltration, defined as a 1.5-fold increase at tumor resection as compared to biopsy.

Treatments

The treatments for this trial are as follows:

Dose Level	Treatment
1 (-1)	1500 mg Durvalumab + 1 fraction of 6 Gy radiation (6 Gy total)
2 (Starting Dose)	1500 mg Durvalumab + 2 fractions of 6 Gy radiation (12 Gy total)
3 (+1)	1500 mg Durvalumab + 3 fractions of 6 Gy radiation (18 Gy total)

3.5.2 Analysis of secondary endpoints

Overall survival (OS) will be determined from the time of enrollment to date of death due to any cause. OS will be evaluated by Kaplan-Meier estimate. Summaries of the number and percentage of patients who have died, are still in survival follow-up, are lost to follow-up, are at the end of study, and have withdrawn consent will be provided along with median OS.

Immune response will be determined by binary measure of whether or not a patient has relevant CD8 infiltration, defined as a 1.5-fold increase at tumor resection as compared to biopsy. Let p represent the proportion of patients with relevant cytotoxic CD8 infiltration. It is expected that half (50%) of patients not receiving this treatment will have relevant CD8 infiltration. The objective is to test the following hypotheses using the exact binomial test:

H_0: $p \leq 0.50$
H_1: $p \geq 0.80$

Tumor response to neoadjuvant therapy (durvalumab + SBRT) will be assessed by pathology review of the surgical specimen. Response will be labeled as complete pathologic remission, microscopic residual tumor (only scattered foci of residual tumor cells) or macroscopic residual tumor.

The method of assessment of disease status at baseline will be MRI neck with and without contrast and PET-CT skull base to mid-thigh. The baseline assessment should be performed

no more than 28 days before SBRT. Efficacy for all patients will be assessed by objective tumor assessment by repeat PET-CT (preferable) and/or MRI neck with and without contrast after the completion of neoadjuvant Durvalumab-radiation therapy and prior to surgery. Response will be assessed by change in metabolic SUV by PET or volumetric gross tumor change on MRI, as determined by a neuroradiologist. A PET-CT skull base to mid-thigh and MRI neck with and without IV contrast will be done again at about 3 months from the end of adjuvant radiation (if given), or for those that do not receive adjuvant radiation, at about 3 months from the end of Cycle 2 adjuvant durvalumab as detailed in the Schedule of Study Assessment in section 8.1.

Distant recurrence rate and locoregional control rate will be determined by surveillance imaging, with failure defined as the appearance of new distant disease or new locoregional disease from the date of enrollment. Kaplan-Meier estimate will be used to describe distant recurrence rate and locoregional control rate.

Rate of contralateral neck failure will be determined by surveillance imaging, with failure defined as the appearance of new cervical lymphadenopathy from the date of enrollment. Kaplan-Meier estimate will be used to describe rate of contralateral neck failure.

Rate of subclinical lymph node involvement at time of surgery will be determined by surgical pathology findings. Rate of objective radiologic response will be determined by pre-surgical MRI and/or PET-CT, as described above.

Incidence of adverse pathologic features that constitute indications for adjuvant therapy will be determined by surgical pathology findings.

Safety and tolerability will be evaluated using the CTCAE v4.03. The safety analysis set (see Section 11.3.3) will be used for safety analyses. The overall safety and tolerability will be assessed throughout the study period. All AE data will be listed individually by treatment group and patient identifier.

Short- and long-term quality of life will be obtained using FACT H&N v4 and will be assessed at baseline, with each cycle, post-SBRT, post-surgically, and throughout adjuvant therapy on a standard schedule.

3.5.3 Analysis of exploratory endpoints

Translational studies will require blood samples, initial biopsy, and tumor tissue at the time of surgery.

1. To establish a gene signature panel that can assess the response to RT-anti-PDL1 response and treatment outcome.
2. Compare change in anti-PDL1 and T cell infiltration pattern and intensity before and after RT and anti-PDL1 by immunohistochemistry.
3. To evaluate the impact of other mutations on immune response and treatment outcome.
4. Blood samples to evaluate biomarkers of immune response as well as for evaluation of circulating auto-antibodies. Blood will be collected prior to treatment, at time of surgery, at end of treatment and at the 6 month follow-up.
5. Tumor tissue taken at the time of initial biopsy and at time of resection will be profiled for tumor infiltrating lymphocytes; activation markers and antigen specific TCR utilization/diversity will be evaluated for additional checkpoint targets.
6. On long-term follow-up, tumor antigen specific T lymphocyte memory populations will

be monitored for representation and robustness in in-vitro stimulation assays as potential biomarker of continued anti-tumor activity.

3.6 Interim Analyses

During part 1 of the trial, after all 3 patients in each cohort have either 1) completed the observation window, or 2) experienced a DLT, the data summarizing the primary endpoint for that cohort (number of DLTs in 3 patients) will be generated. This summary will determine what treatment the next cohort will receive. These may be considered to be interim analyses. There are no planned formal interim analyses during part 2 of the study.

A flexible interim monitoring approach based on predicted intervals will be used [42]. We will predict the confidence interval of PFSR at the end of study condition on the data observed thus far and an assumption that the observed trend continues in the remainder of the study. Predicted interval plots will be created to facilitate the decision of terminating the trial or not [43].

3.7 Study schema

Figure 1.

3.8 Study Design

Part 1 of this trial will use a traditional 3+3 design will be used for this trial (i.e. cohort sizes of 3 patients for the first and second cohort at each dose level). Dose escalation will occur as illustrated in the figure below.

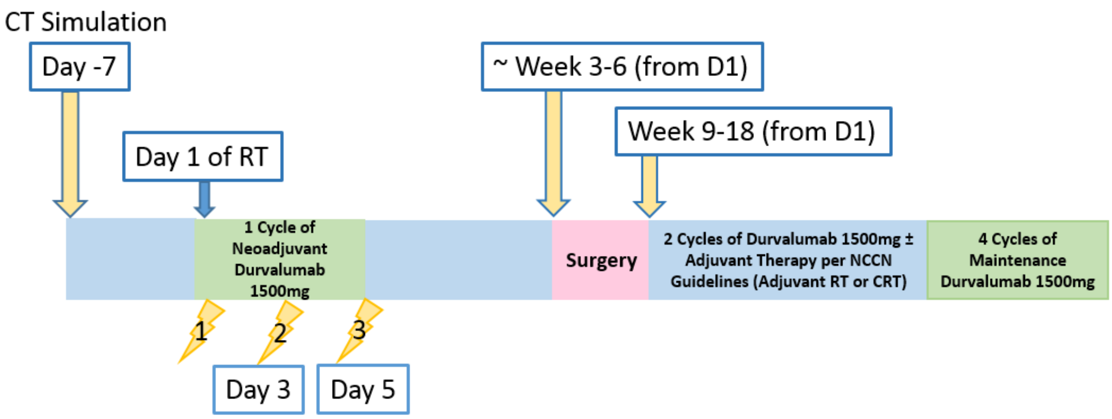

The starting dose level for the trial will be dose level 2. This will allow the trial to continue if there are 2 or 3 DLTs in the first cohort of 3 patients by de-escalating to dose level 1.

Part 2 of this trial will be an expansion cohort. A total of 8 additional patients will be enrolled at the dose level determined to be the MTD in part 1 of the study. These 8 patients will be used to confirm that the MTD is a safe combination, as well as provide additional patients to investigate the efficacy for the treatment combination. For safety purposes, DLT assessments will continue to be documented for patients enrolled in Part 2 of the trial.

3.9 Study oversight for safety evaluation

All adverse events, whether or not unexpected, and whether or not considered associated within the use of the drug will be submitted to the University of Colorado Data Safety Monitoring Board (DSMB) and Institutional Review Board (IRB).

3.10 Interim analyses

During part 1 of the trial, after all 3 patients in each cohort have either 1) completed the observation window, or 2) experienced a DLT, the data summarizing the primary endpoint for that cohort (number of DLTs in 3 patients) will be generated. This summary will determine what treatment the next cohort will receive. These may be considered to be interim analyses. There are no planned formal interim analyses during part 2 of the study.

3.11 Clinical criteria for early trial termination

Early trial termination will be the result of the criteria specified below:

1. Quality of data recording is inaccurate or incomplete
2. Poor adherence to protocol and regulatory requirements
3. Incidence or severity of adverse drug reaction in this or other studies indicates a potential health hazard to subjects
4. Plans to modify or discontinue the development of the study drug
5. Poor treatment efficacy leading to increased locoregional failures resulting in futility at meeting study primary objectives on interim analysis

In the event of an AstraZeneca decision to no longer supply study drug, ample notification will be provided so that appropriate adjustments to subject treatment can be made.

4. PATIENT SELECTION

Each patient must meet all of the inclusion criteria and none of the exclusion criteria for this study. Under no circumstances will there be exceptions to this rule. Re-screening will be allowed if patient met a reversible exclusion criterion that has resolved.

4.1 Inclusion criteria

For inclusion in the study patients must fulfill all of the following criteria:

1. Histologically or cytologically confirmed stage II-IVB oral cavity, stage III-IVB larynx, stage III-IVB hypopharynx, or stage III-IVB HPV and/or p16 negative intermediate-high risk oropharynx head and neck cancer (AJCC 8th edition)
2. Measurable disease defined as lesions that can be accurately measured in at least one dimension (longest diameter to be recorded) as >10 mm with CT, PET/CT, or MRI or >10 mm on visual inspection by clinical exam
3. Patients who are medically operable as determined by ENT surgeon without pre-existing medical conditions that could inhibit surgery following neoadjuvant therapy, and do not refuse surgery
4. Written informed consent and HIPAA authorization obtained from the patient prior to performing any protocol-related procedures, including screening evaluations

5. Age ≥ 18 years at time of study entry
6. ECOG performance status ≤ 1
7. Life expectancy ≥ 24 weeks
8. Body weight >30kg
9. Adequate normal organ and marrow function as defined below:
   - Hemoglobin ≥9.0 g/dL
   - Absolute neutrophil count (ANC) ≥ 1.0 x 10^9/L (≥ 1000 per mm^3)
   - Platelet count ≥75 x 10^9/L (≥75,000 per mm^3)
   - Serum bilirubin ≤1.5 x institutional upper limit of normal (ULN). This will not apply to patients with confirmed Gilbert's syndrome (persistent or recurrent hyperbilirubinemia that is predominantly unconjugated in the absence of hemolysis or hepatic pathology), who will be allowed only in consultation with their physician.
   - AST (SGOT)/ALT (SGPT) ≤2.5 x institutional upper limit of normal
   - Measured creatinine clearance (CL) >40 mL/min or Calculated creatinine CL>40 mL/min by the Cockcroft-Gault formula (Cockcroft and Gault 1976) or by 24-hour urine collection for determination of creatinine clearance:

     Males:

     $$\text{Creatinine CL (mL/min)} = \frac{\text{Weight (kg)} \times (140 - \text{Age})}{72 \times \text{serum creatinine (mg/dL)}}$$

     Females:

     $$\text{Creatinine CL (mL/min)} = \frac{\text{Weight (kg)} \times (140 - \text{Age})}{72 \times \text{serum creatinine (mg/dL)}} \times 0.85$$

10. Evidence of post-menopausal status or negative urinary or serum pregnancy test for female pre-menopausal patients. Women will be considered post-menopausal if they have been amenorrheic for 12 months without an alternative medical cause. The following age-specific requirements apply:
    - Women <50 years of age would be considered post-menopausal if they have been amenorrheic for 12 months or more following cessation of exogenous hormonal treatments and if they have luteinizing hormone and follicle-stimulating hormone levels in the post-menopausal range for the institution or underwent surgical sterilization (bilateral oophorectomy or hysterectomy)
    - Women ≥50 years of age would be considered post-menopausal if they have been amenorrheic for 12 months or more following cessation of all exogenous hormonal treatments, had radiation-induced menopause with last menses >1 year ago, had chemotherapy-induced menopause with last menses >1 year ago, or underwent surgical sterilization (bilateral oophorectomy, bilateral salpingectomy or hysterectomy)

11. Patient is willing and able to comply with the protocol for the duration of the study including undergoing treatment and scheduled visits and examinations including follow up

4.2 Exclusion criteria

Patients should not enter the study if any of the following exclusion criteria are fulfilled:

1. Participation in another clinical study with an investigational product during the last 3 months
2. Patients with active ILD / pneumonitis or with a history of ILD/ pneumonitis requiring steroids
3. Concurrent enrolment in another clinical study, unless it is an observational (non-interventional) clinical study or during the follow-up period of an interventional study
4. Any previous treatment with a PD1 or PD-L1 inhibitor, including durvalumab, anti-PD-L2, anti-CD137, or anti-cytotoxic T-lymphocyte-associated antigen-4 (CTLA-4) antibody (including ipilimumab or any other antibody or drug specifically targeting T-cell co-stimulation or checkpoint pathways)
5. Receipt of the last dose of anticancer therapy (chemotherapy, immunotherapy, endocrine therapy, targeted therapy, biologic therapy, tumor embolization, monoclonal antibodies, other investigational agent) 30 days prior to the first dose of study drug for patients who have received prior TKIs [e.g., erlotinib, gefitinib and crizotinib] and within 6 weeks for nitrosourea or mitomycin C. (*If sufficient wash-out time has not occurred due to the schedule or PK properties of an agent, a longer wash-out period may be required.)*
6. Patients with QTc interval > 470 msec during screening
7. Current or prior use of immunosuppressive medication within 14 days (use 28 days if combining durvalumab with a novel agent) before the first dose of durvalumab, with the exceptions of intranasal and inhaled corticosteroids or systemic corticosteroids at physiological doses, which are not to exceed 10 mg/day of prednisone, or an equivalent corticosteroid. The following are exceptions to this criterion:
   - Intranasal, inhaled, topical steroids, or local steroid injections (e.g., intra articular injection)
   - Systemic corticosteroids at physiologic doses not to exceed 10 mg/day of prednisone or its equivalent
   - Steroids as premedication for hypersensitivity reactions (e.g., CT scan premedication)
8. Any concurrent chemotherapy, IP, biologic, or hormonal therapy that is not part of standard NCCN indicated HNSCC adjuvant concurrent CRT. Concurrent use of hormonal therapy for non-cancer-related conditions (e.g., hormone replacement therapy) is acceptable.
9. History of allogenic organ or bone marrow transplantation
10. Active or prior documented autoimmune or inflammatory disorders (including inflammatory bowel disease [e.g., colitis or Crohn's disease], diverticulitis [with the exception of diverticulosis], systemic lupus erythematosus, Sarcoidosis syndrome, or Wegener syndrome [granulomatosis with polyangiitis, Graves' disease,

rheumatoid arthritis, hypophysitis, uveitis, etc]). The following are exceptions to this criterion:

- Patients with vitiligo or alopecia
- Patients with hypothyroidism (e.g., following Hashimoto syndrome) stable on hormone replacement
- Any chronic skin condition that does not require systemic therapy
- Patients without active disease in the last 5 years may be included but only after consultation with the study physician
- Patients with celiac disease controlled by diet alone

11. Uncontrolled intercurrent illness, including but not limited to, ongoing or active infection, symptomatic congestive heart failure, uncontrolled hypertension, unstable angina pectoris, cardiac arrhythmia, interstitial lung disease, serious chronic gastrointestinal conditions associated with diarrhea, or psychiatric illness/social situations that would limit compliance with study requirement, substantially increase risk of incurring AEs or compromise the ability of the patient to give written informed consent
12. History of another primary malignancy except for:
    - Malignancy treated with curative intent and with no known active disease ≥3 years before the first dose of IP and of low potential risk for recurrence
    - Adequately treated non-melanoma skin cancer or lentigo maligna without evidence of disease
    - Adequately treated carcinoma in situ without evidence of disease
13. History of leptomeningeal carcinomatosis.
14. History of active primary immunodeficiency
15. Active infection including **tuberculosis** (clinical evaluation that includes clinical history, physical examination and radiographic findings, and TB testing in line with local practice), **hepatitis B** (known positive HBV surface antigen (HBsAg) result), **hepatitis C**, or **human immunodeficiency virus** (positive HIV 1/2 antibodies). Patients with a past or resolved HBV infection (defined as the presence of hepatitis B core antibody [anti-HBc] and absence of HBsAg) are eligible. Patients positive for hepatitis C (HCV) antibody are eligible only if polymerase chain reaction is negative for HCV RNA.
16. Receipt of live attenuated vaccine within 30 days prior to the first dose of IP. Note: Patients, if enrolled, should not receive live vaccine whilst receiving IP and up to 30 days after the last dose of IP.
17. Female patients who are pregnant or breastfeeding or male or female patients of reproductive potential who are not willing to employ highly effective birth control from screening to 90 days after the last dose of durvalumab monotherapy. Patient must have a negative serum or urine pregnancy test within 72 hours of study entry.
18. Known allergy or hypersensitivity to any of the study drugs or any of the study drug excipients
19. Prior randomisation or treatment in a previous durvalumab clinical study

20. Patients with p16-positive oropharyngeal SCCA. No verification of p16 status is needed for laryngeal cancer or oral cavity cancer.
21. Patients with sinonasal SCCAs
22. Patients with metastatic SCCA neck disease with an unknown primary tumor site
23. Patients with distant metastatic disease on initial screening imaging
24. Judgment by the investigator that the patient is unsuitable to participate in the study and the patient is unlikely to comply with study procedures, restrictions and requirements

Procedures for withdrawal of incorrectly enrolled patients are presented in Section 4.3 If a patient withdraws from participation in the study, then his or her enrollment/randomization code cannot be reused. Withdrawn patients can be replaced.

4.3 Withdrawal of patients from study treatment and/or study

4.3.1 Permanent discontinuation of durvalumab and/or RT

An individual patient will not receive any further investigational product if any of the following occur in the patient in question:

1. Withdrawal of consent or lost to follow-up
2. Adverse event that, in the opinion of the investigator or the sponsor, contraindicates further dosing
3. Patient is determined to have met one or more of the exclusion criteria for study participation at study entry and continuing investigational therapy might constitute a safety risk
4. Pregnancy or intent to become pregnant
5. Any AE that meets criteria for discontinuation as defined in Section 6.6
6. Adverse event related to durvalumab or RT with the exception of toxicities that do not meet the criteria for discontinuation as defined in Section 6.6
7. Dose-limiting toxicity (See Section 6.4 for definition of DLT)
8. Grade ≥3 infusion reaction
9. Patient noncompliance that, in the opinion of the investigator or sponsor, warrants withdrawal; e.g., refusal to adhere to scheduled visits
10. Initiation of alternative anticancer therapy including another investigational agent
11. Confirmation of PD and investigator determination that the patient is no longer benefiting from treatment with durvalumab
12. Patients who are permanently discontinued from receiving investigational product will be followed for safety per Section 10.3.1, including the collection of any protocol-specified blood specimens, unless consent is withdrawn or the patient is lost to follow-up or enrolled in another clinical study. All patients will be followed for survival. Patients who decline to return to the site for evaluations will be offered follow-up by phone every 3 months as an alternative.

4.3.2 Withdrawal of consent

Patients are free to withdraw from the study at any time (IP and assessments) without prejudice to further treatment. Patients who withdraw consent for further participation in the study will not receive any further IP or further study observation, with the exception of follow-up for survival, which will continue until the end of the study unless the patient has expressly withdrawn their

consent to survival follow-up. Note that the patient may be offered additional tests or tapering of treatment to withdraw safely. A patient who withdraws consent will always be asked about the reason(s) for withdrawal and the presence of any AE. The Investigator will follow up AEs outside of the clinical study.

If a patient withdraws consent, they will be specifically asked if they are withdrawing consent to:

- all further participation in the study including any further follow up (e.g., survival contact telephone calls)
- withdrawal of consent to the use of their study generated data
- withdrawal to the use of any samples

4.4 Replacement of patients

A patient incorrectly enrolled will be withdrawn from the study. This patient may be replaced with another patient at the same and/or correct dose and regimen. Additionally, patients who do not comply with the protocol as outlined and are not considered evaluable for analysis by the PI, may be replaced.

5. INVESTIGATIONAL PRODUCT(S)

5.1 Durvalumab

The Investigational Products Supply section of AstraZeneca/MedImmune will supply durvalumab (MEDI4736) to the investigator as a 500-mg vial solution for infusion after dilution.

5.1.1 Formulation/packaging/storage

Durvalumab (MEDI4736) will be supplied by AstraZeneca as a 500-mg vial solution for infusion after dilution. The solution contains 50 mg/mL durvalumab (MEDI4736), 26 mM histidine/histidinehydrochloride, 275 mM trehalose dihydrate, and 0.02% weight/volume (w/v) polysorbate 80; it has a pH of 6.0. The nominal fill volume is 10.0 mL. Investigational product vials are stored at 2°C to 8°C (36°F to 46°F) and must not be frozen. Drug product should be kept in secondary packaging until use to prevent excessive light exposure.

5.1.2 Durvalumab doses and treatment regimens

5.1.2.1 Neoadjuvant

All enrolled patients will receive 1500 mg durvalumab via IV infusion for 1 dose neoadjuvantly approximately 3-6 weeks prior to standard of care surgery. The first dose of durvalumab can be administered at any time between SBRT fractions 1-3. RT will be 6 Gy according to dose level assignment. It will be given every other day over approximately one week to sites of gross disease only to minimize exposure to normal tissue. The SBRT can be planned as an IMRT. If a dose limiting toxicity develops and/or surgery is delayed by > 8 weeks, this will be considered a DLT. The individual patient will be withdrawn from the study and future patients will be dropped down to the next radiation dose level per protocol. Following neoadjuvant treatment, the patient will proceed to surgical resection as directed by the ENT surgeon.

5.1.2.2 Adjuvant

Adjuvant chemotherapy and radiation therapy will be dictated by surgical pathology and NCCN guidelines. Post-operatively, adjuvant Durvalumab will be given regardless of risk status starting concurrently with radiation if indicated or between 6-12 weeks post-op if no standard adjuvant therapy is needed. Durvalumab will be given as 1500 mg, intravenously once every 4 weeks for a maximum of 6 doses (including two concurrent with radiation or chemoradiation if indicated).

The duration of infusion will be approximately 1 hour. A 60 minutes observation period is required after the first infusion of durvalumab. If no significant reactions are observed during or after the first cycle, subsequent infusion observation periods can be at the physician discretions.

When durvalumab is given in combination with adjuvant chemotherapy, durvalumab will be given first followed by chemotherapy. It is recommended that there be 30 minutes between the end of the durvalumab administration and the start of the chemotherapy administration.

5.1.3 Study drug preparation

Patients in the durvalumab (MEDI4736) monotherapy treatment group will receive 1500mg durvalumab (MEDI4736) via IV infusion for one dose neoadjuvantly with RT and then 1500 mg via IV infusion Q4W for a maximum of 6 doses adjuvantly unless there is unacceptable toxicity, withdrawal of consent, or another discontinuation criterion is met. (If a patient's weight falls to 30kg or below the patient should receive weight-based dosing equivalent to 20 mg/kg of durvalumab Q4W until the weight improves to >30 kg, at which point the patient should start receiving the fixed dosing of durvalumab 1500mg Q4W).

5.1.3.1 Preparation of durvalumab doses for administration with an IV bag

The dose of durvalumab (MEDI4736) for administration must be prepared by the Investigator's or site's designated IP manager using aseptic technique. Total time from needle puncture of the durvalumab (MEDI4736) vial to the start of administration should not exceed:

- 24 hours at 2°C to 8°C (36°F to 46°F)
- 4 hours at room temperature

Infusion solution must be allowed to equilibrate to room temperature prior to commencement of administration.

A dose of 1500mg (for patients >30kg in weight) will be administered using an IV bag containing 0.9% (w/v) saline or 5% (w/v) dextrose, with a final durvalumab (MEDI4736) concentration ranging from 1 to 20 mg/mL, and delivered through an IV administration set with a 0.2- or 0.22-µm in-line filter. Add 30.0 mL of durvalumab (MEDI4736) (ie, 1500mg of durvalumab [MEDI4736]) to the IV bag. The IV bag size should be selected such that the final concentration is within 1 to 20 mg/mL. Mix the bag by gently inverting to ensure homogeneity of the dose in the bag.

If weight falls to ≤ 30 kg weight-based dosing at 20 mg/kg will be a administered using an IV bag containing 0.9% (w/v) saline or 5% (w/v) dextrose, with a final durvalumab (MEDI4736) concentration ranging from 1 to 20 mg/mL, and delivered through an IV administration set with

a 0.2- or 0.22-μm in-line filter. The appendix includes an example of a weight-based dose calculation.

Standard infusion time is 1 hour. In the event that there are interruptions during infusion, the total allowed infusion time should not exceed 8 hours at room temperature.

Do not co-administer other drugs through the same infusion line.

The IV line will be flushed with a volume of IV diluent equal to the priming volume of the infusion set used after the contents of the IV bag are fully administered, or complete the infusion according to institutional policy to ensure the full dose is administered and document if the line was not flushed.

If either preparation time or infusion time exceeds the time limits a new dose must be prepared from new vials. Durvalumab (MEDI4736) does not contain preservatives, and any unused portion must be discarded.

5.1.4 Monitoring of dose administration

Patients will be monitored before, during and after the infusion with assessment of vital signs at the times specified in the Schedule of Study Assessments during the first infusion. Patients are monitored every 30 minutes during the infusion period (including times where infusion rate is slowed or temporarily stopped). For subsequent infusions, vital signs will be done prior to infusion and then only as clinically indicated.

In the event of a ≤Grade 2 infusion-related reaction, the infusion rate of study drug may be decreased by 50% or interrupted until resolution of the event (up to 4 hours) and re-initiated at 50% of the initial rate until completion of the infusion. For patients with a ≤Grade 2 infusion related- reaction, subsequent infusions may be administered at 50% of the initial rate. Acetaminophen and/or an antihistamine (e.g., diphenhydramine) or equivalent medications per institutional standard may be administered at the discretion of the investigator. If the infusion related- reaction is Grade 3 or higher in severity, study drug will be discontinued. The standard infusion time is one hour, however if there are interruptions during infusion, the total allowed time from infusion start to completion of infusion should not exceed 4 hours at room temperature, with maximum total time at room temperature not exceeding 4 hours (otherwise requires new infusion preparation). For management of patients who experience an infusion reaction, please refer to the toxicity and management guidelines in Appendix 1.

As with any antibody, allergic reactions to dose administration are possible. Appropriate drugs and medical equipment to treat acute anaphylactic reactions must be immediately available, and study personnel must be trained to recognize and treat anaphylaxis. The study site must have immediate access to emergency resuscitation teams and equipment in addition to the ability to admit patients to an intensive care unit if necessary.

5.1.5 Accountability and dispensation

The study drug provided for this study will be used only as directed in the study protocol. The study personnel will account for all study drugs.

Drug accountability should be performed until the patient stops study treatment completely. Study site personnel will account for all study drugs received at the site, for all unused study

drugs, and for appropriate destruction of study drugs. Certificates of delivery, destruction, and return should be signed.

Study drug will not be distributed to the study site until the contract is concluded between the study site and AstraZeneca. The research pharmacist is responsible for managing the study drug from receipt by the study site until the return of all unused study drug to AstraZeneca. AstraZeneca will provide the study documents "Procedures for drug accountability" and "Procedures for drug storage," which describe the specific requirements.

5.1.6 Disposition of unused investigational study drug

The site will account for all investigational study drug dispensed and also for appropriate destruction. Certificates of delivery and destruction must be signed.

5.2 Standard of Care Adjuvant Therapy

Post-operatively, pathology will be reviewed for risk factors (including positive margins, extracapsular nodal spread, pT4, pN2 or pN3 disease) suggesting the need for adjuvant radiation +/- chemotherapy by NCCN guidelines. Final decision for adjuvant therapy will be per treating physician decision. Adjuvant systemic therapy to be given with radiation will be per treating medical oncologist discretion. Preferred regimens will contain either platinum cytotoxic chemotherapy (cisplatin or carboplatin) or cetuximab.

5.2.1 Formulation/packaging/storage

Formulation, package, and storage will be per local institution usual practice for these standard of care drugs.

5.2.2 Doses and treatment regimens

Dose and schedule of concurrent standard of care systemic therapy will be per treating physician discretion. This data will be recorded in the patient record.

5.2.3 Product preparation

The product preparation will be determinant upon systemic therapy chosen and usual practise of the local institution pharmacy personnel.

5.2.4 Dose administration

The dose of the chosen systemic therapy will be administered per local institution standard infusion center protocol.

5.2.5 Monitoring of dose administration

The chosen systemic therapy will be monitored during and post-infusion per local institution standard infusion center protocol.

5.2.6 Accountability and dispensation

Drug accountability should be performed until the patient stops treatment completely. Delivery, destruction, and return should be documented per institutional standards.

6. TREATMENT PLAN

6.1 Patient enrollment

Before recruitment of patients into the study, written IRB approval of the protocol, informed consents, and any additional patient information must be obtained. The investigator will maintain a patient log for all screened (including patients that failed screening) patients.

At screening, the investigator or appropriately trained delegate will:

1. Obtain informed consent
2. Determine patient eligibility
3. Obtain a unique case enrollment number. This case enrollment number is the patient's unique identifier and is used to identify the patient on the case report forms.

Study-related procedures must not commence before obtaining consent. However, results from assessments performed before obtaining informed consent that are considered "routine standard of care" (e.g. laboratory results, CT scans, etc.) may be used to determine eligibility. The physician in charge of the patient is responsible for verifying that the patient is eligible before requesting enrollment. If any of the inclusion criteria are not met or any of the exclusion criteria are met, that patient should not be enrolled. This is an open-label, unblinded study. Treatment assignment will be known by the investigator and the patient. Once enrolled in the study, the patient will be only identified by initials and the assigned patient number. Patients will start treatment preferably within 28 days of enrollment.

6.1.1 Procedures handling patients incorrectly enrolled

Patients who fail to meet the eligibility criteria will not be enrolled or receive study medication. Patients who are enrolled but found not to meet all eligibility criteria will not be initiated on treatment and will be withdrawn from the study as a screen failure. The withdrawn subjects will be replaced with another subject.

6.2 Dosage and administration

6.2.1 Neoadjuvant

All patients will receive 1500mg durvalumab (MEDI4736) via IV infusion for one dose neoadjuvantly with RT as described in section 5. If a patient's weight falls to 30kg or below the patient should receive weight-based dosing equivalent to 20 mg/kg of durvalumab Q4W until the weight improves to >30 kg, at which point the patient should start receiving the fixed dosing of durvalumab 1500 mg Q4W. Otherwise, there is not a planned escalation or de-escalation dose, but Durvalumab will be discontinued with any drug-related AE with grade 3 or greater.

Radiation will be given as 2 fractions of 6 Gray every other day over approximately one week. If a dose limiting toxicity due to radiation develops and/or surgery is delayed by more than 8 weeks, the radiation dose will be de-escalated. RT dose can be adjusted from 2 fractions of 6 Gray a fraction to 1 fraction of 6 Gray a fraction.

6.2.2 Adjuvant

All patients will receive adjuvant Durvalumab (either alone or concurrently with adjuvant radiation +/- chemotherapy) within 6-12 weeks post-operatively as described in section 5. Durvalumab will be given as 1500 mg via IV infusion Q4W for a maximum of 6 doses.

Adjuvant radiation and systemic therapy will be guided by surgical pathology, NCCN guidelines, and treating physician discretion for high risk patients. This will be started within 6-12 weeks post-operatively if necessary and will be given concurrently with Durvalumab.

6.3 Dose escalation decision rules

Doses of Durvalumab will not be escalated. Radiation fractions will be increased by 3+3 study design. If patients tolerate 6 Gy of SBRT for 2 fractions with no DLTs, the next cohort will be escalated to 6 Gy of SBRT for 3 fractions.

6.4 Definition of DLT

Dose-limiting toxicities (DLTs) will be evaluated during the entirety of part 1 (dose escalation) of the trial. The period for evaluating DLTs will be from the time of first administration of durvalumab until the day of surgery. Patients who do not remain on the study up to this time for reasons other than DLT will be replaced with another patient at the same dose level. Grading of adverse effects (AEs) will follow the guidelines provided in the Common Terminology Criteria for Adverse Events (CTCAE) version 4.03.

A DLT will be defined as any Grade 3 or higher toxicity that occurs during the DLT evaluation period (from initiation of treatment until the day of surgery). Toxicity that is clearly and directly related to the gross disease or to another etiology is excluded from this definition. Only toxicity that delays surgery by > 8 weeks will be considered a DLT. The following will be DLTs related to Durvalumab and/or SBRT:

- Any Grade 4 rash or dermatitis
- Any ≥Grade 3 colitis and/or diarrhea that does not resolve to a ≤Grade 1 or baseline within 14 days
- Any Grade 3 or 4 noninfectious pneumonitis irrespective of duration
- Any Grade 2 pneumonitis that does not resolve to ≤Grade 1 within 3 days of the initiation of maximal supportive care
- Grade 4 anemia regardless of duration
- Grade 4 neutropenia lasting more than 7 days
- Grade 4 thrombocytopenia regardless of duration. Liver transaminase elevation > 8 × ULN or total bilirubin > 5 × ULN
- Grade 3 and 4 increase in creatinine (>3x ULN). In patients with baseline creatinine above ULN, DLT is creatinine increase >3x baseline
- Any Grade 3 irAE, excluding colitis or pneumonitis, that does not downgrade to Grade 2 within 3 days after onset of the event despite optimal medical management including systemic corticosteroids or does not downgrade to ≤Grade 1 or baseline within 14 days
- Any other Grade 4 irAE regardless of duration
- Grade 3 radiation induced mucositis or dermatitis
- Any ≥Grade 3 non-irAE, except for the exclusions listed below

The definition excludes the following conditions:

- Grade 3 fatigue lasting ≤7 days

- Grade 3 endocrine disorder (thyroid, pituitary, and/or adrenal insufficiency) that is managed with or without systemic corticosteroid therapy and/or hormone replacement therapy and the patient is asymptomatic
- Grade 3 inflammatory reaction attributed to a local antitumor response (e.g., inflammatory reaction at sites of metastatic disease, lymph nodes, etc)
- Concurrent vitiligo or alopecia of any AE grade
- Grade 3 infusion-related reaction (first occurrence and in the absence of steroid prophylaxis) that resolves within 6 hours with appropriate clinical management
- Grade 3 or 4 neutropenia that is not associated with fever or systemic infection that improves by at least 1 grade within 3 days. Grade 3 or Grade 4 febrile neutropenia will be a DLT regardless of duration or reversibility
- Grade 3 or 4 lymphopenia
- Grade 3 thrombocytopenia that is not associated with clinically significant bleeding that requires medical intervention, and improves by at least 1 grade within 3 days
- Isolated Grade 3 electrolyte abnormalities that are not associated with clinical signs or symptoms and are reversed with appropriate maximal medical intervention within 3 days

Immune-related AEs are defined as AEs of an immune nature (i.e., inflammatory) in the absence of a clear alternative etiology. In the absence of a clinically significant abnormality, repeat laboratory testing will be conducted to confirm significant laboratory findings prior to designation as a DLT.

6.5 Tracking toxicity of investigational product(s)

In addition to the above AEs and AESIs, following surgery, patients will also be monitored for the following and will be reported as delays possibly due to immunotherapy and/or SBRT toxicity:

- Patients remaining as an inpatient after post-op day 21

6.6 Toxicity management guidelines

6.6.1 Durvalumab

Guidelines for the management of immune-mediated reactions, infusion-related reactions, and non-immune-mediated reactions for durvalumab are provided in the Dosing Modification and Toxicity Management Guidelines in Appendix 1. Patients should be thoroughly evaluated and appropriate efforts should be made to rule out neoplastic, infectious, metabolic, toxin, or other etiologic causes of the imAE. Serologic, immunologic, and histologic (biopsy) data, as appropriate, should be used to support an imAE diagnosis. In the absence of a clear alternative etiology, events should be considered potentially immune related.

In addition, there are certain circumstances in which durvalumab should be permanently discontinued (see section 4.3.1 of this protocol and the Dosing Modification and Toxicity Management Guidelines in Appendix 1).

Dose reductions are not permitted except for if weight drops to < 30 kg as specified in section 6.2.1. In case of doubt, the Investigator should consult with the Principal Investigator.

Patients should be thoroughly evaluated and appropriate efforts should be made to rule out neoplastic, infectious, metabolic, toxin, or other etiologic causes of the imAE. Serologic, immunologic, and histologic (biopsy) data, as appropriate, should be used to support an imAE

diagnosis. In the absence of a clear alternative etiology, events should be considered potentially immune related.

All toxicities will be graded according to NCI CTCAE, Version 4.03.

6.7 Neoadjuvant (Preoperative) Radiation Therapy

Total Prescribed Dose

In the neoadjuvant setting, patients will receive radiation therapy according to assigned dose level to the gross disease. The course of radiation therapy will start on Day 1. Patients will be treated every other day (Monday – Friday) over the course of 1 week. Radiation will occur approximately 3-6 weeks prior to surgery.

Dose Coverage

The prescription dose should aim to cover a minimum of 90-95% of the planning target volume (PTVSBRT). A consideration for minimum Dmax is 138%-140% and maximum Dmax of 160%. Please note that the SBRT can be planned as an IMRT with similar constraints.

PTV expansion:
The planning target volume (PTV_{SBRT}) will then be generated with an expansion of 2-3mm in all directions from the GTV_{SBRT}.

Minimum Dose

The minimum dose within the PTV_{SBRT} is recommended least 90-95% of the prescribed dose. However, this requirement is not mandatory.

High density material
If there is high density/metal (fillings) in the PTV, it should be excluded on the PTV eval.

6.7.1 Technical factors

Targeting and Treatment

This protocol will require treatments to be performed with an image-guided technique with the use of a 3-D coordinate system defined by 3D volumetric data or orthogonal 2D images.

Image guided radiation therapy (IGRT) will be used for target localization. See section 6.7.5 for a list of techniques.

6.7.2 Set up, treatment planning and localization

Patient Set-up

Patients will be positioned supine in a stable and comfortable position allowing for accurate reproducibility. The immobilization system will use thermoplastic head and neck mask that will conform to the patient' external contours allowing for setup reliability.

Treatment Planning

Computed Tomography (CT)

CT will be the primary image platform for treatment planning. The simulation CT scan should be performed in the supine treatment position. Axial cuts of 3mm or less will be acquired from the level of the top of the head to the carina. It is advised that no mouth piece is used at the time of sim so a better fusion of the PET and MRI can be done for planning purposes.

Contrast

IV contrast can be used at the physician's discretion.

Image fusion

Fusion of the diagnostic PET/CT and MRI neck (and any other available imaging) will be generated to obtain the gross tumor volume (GTV_{SBRT}). GTV_{SBRT} represents the region judged to contain gross tumor based on clinical and endoscopic examinations, and imaging techniques (CT, PET/CT and MRI).

Planning

For patients with N1-N2b disease, two separate radiation plans and two separate PTVs will be formulated with 2 separate isocenter points. For those with 2 separate radiation plans (gross disease and neck), both radiation plans will be delivered on the same day. Patients will be treated every other day (Monday-Friday) over the course of 1 week.

Localization

IGRT techniques will be used for localization to ensure accurate beam positioning.

6.7.3 Critical structures

Definition of normal tissue structures/organs at risk (OARs)

The spinal cord will be contoured based on the bony limits of the spinal canal and should be contoured starting at the cranial-cervical junction (i.e., the top of the C1 vertebral body). The inferior border of the spinal cord should be below the lowest slice level that has PTV on it. The spinal cord shall be defined based on the treatment planning CT scan. In addition, however, a Planning Risk Volume (PRV) spinal cord shall be defined. The PRVcord = cord + 5 mm in each dimension.

The brainstem will be contoured starting at the level of the top of the posterior clinoid (superior most portion) extending to the cranial-cervical junction where it meets the spinal cord (inferior most portion). The brainstem shall be defined based on the treatment planning CT scan. In addition, the PRVbrainstem = brainstem + 3 mm in each dimension

The oral cavity will be contoured as a composite structure consisting of the anterior 1/2 to 2/3 of the oral tongue/floor of mouth, buccal mucosa, and palate. The oral cavity OAR should not overlap any PTV_{SBRT}.

The lips will be contoured as a separate structure from the oral cavity. The goal is to keep the lip dose much lower than the oral cavity dose.

The parotid glands will be contoured as a composite structure consisting of both deep and superficial lobes. The objective is to limit the mean dose to less than < 12 Gy; alternatively, at least 20 cc of the combined volume of both parotid glands to < 10 Gy or at least 50% of the ipsi parotid gland to < 10 Gy.

The pharynx will be contoured as the "uninvolved" posterior pharyngeal wall plus adjacent constrictor muscles. This extends from the superior constrictor region (the inferior pterygoid plates level) to the cricopharyngeal inlet (posterior cricoid cartilage level). This should not overlap with the PTVs.

The cervical esophagus will be contoured as a tubular structure that starts at the bottom of the pharynx and extends to the thoracic inlet.

The glottis/supraglottic larynx (GSL) will be contoured as a "triangular prism shaped" volume that begins just inferior to the hyoid bone and extends to the cricoid cartilage inferiorly and extends from the anterior commissure to include the arytenoids. This includes the infrahyoid but not suprahyoid epiglottis.

The mandible will be contoured as the entire bony structure of the mandible from TMJ through the symphysis. It is recognized that in many cases, this may overlap with PTVs.

Brachial plexus contouring can be delineated as outlined by Hall 2008 [44]. It comprises of linear structures of 5 mm thickness that extend from the neural foramina of C5 through T1 to the small space between the anterior and middle scalene muscles. For CT slices where no neural foramen is present, one can contour only the space between the anterior and middle scalene muscles. If one follows the space between these muscles inferiorly; one will find the cords of the brachial plexus posterior to subclavian neurovascular bundle. They are the non-enhanced structures posterior to the enhanced subclavian vein. These cords extent laterally along the axillary vein into the axilla.

The brain, cochlea, globes, lens, optic nerves, and optic chiasm will be contoured for all cases.

Radiation dosimetry constraints

Dose constraints guidelines according to AAPM Task Group 101 for SBRT in 2-3 fractions.

Two-three fractions:

Serial Tissue	Volume	Volume Max (Gy)	Max Point Dose (Gy)**	Endpoint (≥Grade 3)
Optic Pathway	<0.2 cc	15.3 Gy	17.4 Gy	Neuritis
Cochlea			14.4 Gy	hearing loss
Brainstem (not medulla)	<0.5 cc	15.9 Gy	23.1 Gy	cranial neuropathy
Spinal Cord and medulla	<0.35 cc<br><1.2 cc	15.9 Gy<br>12.3 Gy	22.5 Gy	myelitis

Serial Tissue	Volume	Volume Max (Gy)	Max Point Dose (Gy)**	Endpoint (≥Grade 3)
Spinal Cord Subvolume (5-6 mm above and below level treated per Ryu)	<10% of subvolume	18 Gy	22.5 Gy	myelitis
Cauda Equina	<5 cc	21.9 Gy	25.5 Gy	Neuritis
Sacral Plexus	<5 cc	22.5 Gy	24 Gy	neuropathy
Esophagus*	<5 cc	17.7 Gy	25.2 Gy	stenosis/fistula
Brachial Plexus	<3 cc	22 Gy	26 Gy	neuropathy
Heart/Pericardium	<15 cc	24 Gy	30 Gy	pericarditis
Great vessels	<10 cc	39 Gy	45 Gy	aneurysm
Trachea and Large Bronchus*	<5 cc	25.8 Gy	30 Gy	stenosis/fistula
Bronchus- smaller airways	<0.5 cc	18.9 Gy	23.1 Gy	stenosis with atelectasis
Rib	<5 cc	40 Gy	50 Gy	Pain or fracture
Skin	<10 cc	31 Gy	33 Gy	ulceration
Stomach	<5 cc	22.5 Gy	30 Gy	ulceration/fistula
Bile duct			36 Gy	stenosis
Duodenum*	<5 cc<br><10 cc	15.6 Gy<br>12.9 Gy	22.2 Gy	ulceration
Jejunum/Ileum*	<30 cc	17.4 Gy	27 Gy	enteritis/obstruction
Colon*	<20 cc	24 Gy	30 Gy	colitis/fistula
Rectum*	<20 cc	27.5 Gy	35 Gy	proctitis/fistula
Ureter			40 Gy	stenosis
Bladder wall	<15 cc	17 Gy	33 Gy	cystitis/fistula
Penile bulb	<3 cc	25 Gy		impotence
Femoral Heads	<10 cc	24 Gy		necrosis
Renal hilum/vascular trunk	15 cc	19.5 Gy		malignant hypertension
**Parallel Tissue**	**Critical Volume (cc)**	**Critical Volume Dose Max (Gy)**		**Endpoint (≥Grade 3)**
Lung (Right & Left)	1500 cc	10.5 Gy		Basic Lung Function
Lung (Right & Left)	1000 cc	11.4 Gy	V-11Gy<37%	Pneumonitis
Liver	700 cc	17.1 Gy		Basic Liver Function
Renal cortex (Right & Left)	200 cc	15 Gy		Basic renal function

Single fraction:

Serial Tissue	Volume	Volume Max (Gy)	Max Point Dose (Gy)**	Endpoint (≥Grade 3)
Optic Pathway	<0.2 cc	8 Gy	10 Gy	Neuritis
Cochlea			9 Gy	hearing loss

Serial Tissue	Volume	Volume Max (Gy)	Max Point Dose (Gy)**	Endpoint (≥Grade 3)
Brainstem (not medulla)	<0.5 cc	10 Gy	15 Gy	cranial neuropathy
Spinal Cord and medulla	<0.35 cc<br><1.2 cc	10 Gy<br>7 Gy	14 Gy	myelitis
Spinal Cord Subvolume (5-6 mm above and below level treated per Ryu)	<10% of subvolume	10 Gy	14 Gy	myelitis
Cauda Equina	<5 cc	14 Gy	16 Gy	Neuritis
Sacral Plexus	<5 cc	14.4 Gy	16 Gy	neuropathy
Esophagus*	<5 cc	11.9 Gy	15.4 Gy	stenosis/fistula
Brachial Plexus	<3 cc	13.6 Gy	16.4 Gy	neuropathy
Heart/Pericardium	<15 cc	16 Gy	22 Gy	pericarditis
Great vessels	<10 cc	31 Gy	37 Gy	aneurysm
Trachea and Large Bronchus*	<4 cc	17.4 Gy	20.2 Gy	stenosis/fistula
Bronchus- smaller airways	<0.5 cc	12.4 Gy	13.3 Gy	stenosis with atelectasis
Rib	<5 cc	28 Gy	33 Gy	Pain or fracture
Skin	<10 cc	25.5 Gy	27.5 Gy	ulceration
Stomach	<5 cc	17.4 Gy	22 Gy	ulceration/fistula
Bile duct			30 Gy	stenosis
Duodenum*	<5 cc<br><10 cc	11.2 Gy<br>9 Gy	17 Gy	ulceration
Jejunum/Ileum*	<30 cc	12.5 Gy	22 Gy	enteritis/obstruction
Colon*	<20 cc	18 Gy	25 Gy	colitis/fistula
Rectum*	<20 cc	22 Gy	30 Gy	proctitis/fistula
Ureter			35 Gy	stenosis
Bladder wall	<15 cc	12 Gy	25 Gy	cystitis/fistula
Penile bulb	<3 cc	16 Gy		impotence
Femoral Heads	<10 cc	15 Gy		necrosis
Renal hilum/vascular trunk	15 cc	14 Gy		malignant hypertension
**Parallel Tissue**	**Critical Volume (cc)**	**Critical Volume Dose Max (Gy)**		**Endpoint (≥Grade 3)**
Lung (Right & Left)	1500 cc	7 Gy		Basic Lung Function
Lung (Right & Left)	1000 cc	7.6 Gy	V-8Gy <37%	Pneumonitis
Liver	700 cc	11 Gy		Basic Liver Function
Renal cortex (Right & Left)	200 cc	9.5 Gy		Basic renal function

<u>Dose constraints to other normal structures</u>

Lips: Reduce the dose as much as possible. The mean dose should be < 10 Gy.

Oral Cavity: Reduce the dose as much as possible unless oral cavity is the target. The mean dose should be < 15 Gy if feasible. Efforts should be made to avoid hot spots (> 30 Gy) within the oral cavity.

Parotid Glands: The goal is to keep the mean dose to at least one parotid gland to < 12 Gy. Additional planning goals may include: 1) At least 50% of one parotid will receive < 15 Gy; and/or 2) At least 20 cc of parotid tissue (from the combination of both glands) will receive < 10 Gy.

Pharynx*:* Reduce the dose as much as possible. Some recommended (but not mandatory) treatment goals include: 1) No more than 33% of the pharynx exceeds 25 Gy; 2) Mean dose < 20 Gy; 3) No more than 10% of the pharynx exceeds 30 Gy.

Glottic and supraglottic larynx (GSL): Reduce the dose as much as possible. It is recommended that the dose to the larynx should be kept < 20 Gy whenever feasible.

Mandible: Reduce the dose as much as possible. It is recognized that particularly for these cancers, portions of the mandible will overlap the CTVs and/or PTVs; however, hot spots within the mandible should be avoided. It is recommended that maximum dose within the mandible be < 30 Gy whenever possible.

6.7.4 Image Guidance for Target Localization

After the patient is setup on the treatment table, image guidance will be used to align the patient with treatment machine based on the treatment plan. The alignment will be evaluated and approved by the treating radiation oncologist prior to treatment. Alignment should prioritize matching to the PTV(s). The alignment data will be recorded.

Image guided radiation therapy (IGRT) will be used to ensure optimal alignment. The specific IGRT techniques are listed below in section 6.7.5. CT images or on-board MRI will be taken prior to radiation therapy.

6.7.5 ADJUVANT (POSTOPERATIVE) RADIATION THERAPY

Dose Specifications and Technical factors

The prescribed radiotherapy dose, postoperatively, will be 60 Gy in 2 Gy once-daily fraction size (total of 30 fractions). The daily dose of 2 Gy will be prescribed such that 95% of the PTV60 volume receives at least 60 Gy. PTV56 is also used, and PTV66 (given as an integrated boost) may be optionally defined. All patients will be treated with IMRT.

Localization, Simulation, and Immobilization

Patients must have an immobilization device (e.g., aquaplast mask) made prior to treatment planning CT scan. The treatment planning CT scan can be performed with IV contrast at the physician’s discretion so that the major vessels of the neck are easily visualized. The treatment planning CT scan must be performed with the immobilization device and in the treatment position. Slice thickness should be 0.3 cm.

Dose-limiting normal tissue constraints are listed. The spinal cord dose may not exceed 48 Gy to any volume larger than 0.03 cc.

Technical Factors

Treatment Planning/Delivery: Megavoltage energy photon beam irradiation is required. Any treatment planning and delivery system that has been credentialed for head and neck IMRT.

Image Guidance for IGRT: Daily image guidance of IMRT may be achieved using any one or more of the following techniques:

- Orthogonal kilovoltage (KV) images, e.g. ExacTrac;
- Linear-accelerator mounted kV and MV conebeam CT images;
- Linear-accelerator mounted MV CT images (e.g., Tomotherapy);
- Other mechanism, after discussion with the Study PI and Medical Physics Co-chair.

The institution's procedure to register treatment day imaging dataset with the reference dataset should comply with the following recommendations:

- Region-of-Interest (ROI) or "clip box" for fusion should be set to encompass the high dose PTV and adjacent spinal cord; if the supraclavicular region is a part of the target volume the ROI should extend to the C6 level;
- If the fusion software allows the user to create an irregular ROI (e.g., ExacTrac), treatment room objects seen on in-room X-rays should be excluded from the registration;
- Both manual (e.g., based on bony anatomy) and automatic (e.g., based on mutual information) types of registration can be used; the result of the fusion must be visually checked for the alignment of the bony anatomy, such as vertebral bodies and applicable soft tissue structures (e.g., optic nerves and/or optic chiasm).

Following the registration, the translational and (if the appropriate technology is available) rotational corrections should be applied to the treatment couch. If all the variances are less than 2.5 mm (this typically corresponds to one half of the usual PRV margin), the treatment can proceed without correction (however, the physician/team may elect to perform adjustments even for a variance < 2.5 mm). If one or more corrections are 2.5-5 mm, adjustment is necessary prior to treatment; however, re-imaging is not mandatory. If one or more of the corrections are larger than 5 mm, the imaging must be repeated in addition to performing table/positioning adjustments.

Management of Radiation Dose to the Patient from IGRT

Radiation doses from IGRT are small enough dose contributions that if there is only one imaging study done per treatment session, the dose does not need to be incorporated into treatment planning and is not expected to have any clinical relevance to the patient. However, the imaging dose to the patient may become significant if repeated studies are done for patients with severe set up problems (e.g., requiring frequent corrections of more than 5 mm). It is recommended that patients demonstrating severe set up problems during the first week of treatment be moved to a treatment with larger margins.

Target and Normal Tissue Volume Definitions

Definition of Target Volumes

<u>CTV60:</u> This volume will receive 2 Gy per day. CTV60 will include the primary tumor bed (based on preoperative imaging, preoperative physical exam/endoscopy, operative findings,

pathologic findings) plus region(s) of grossly involved lymphadenopathy. It is recognized that after surgery, there can be considerable distortion of normal anatomy. If possible, map preoperative GTV(s) onto the postoperative radiation therapy planning CT scan, and add appropriate margins for microscopic spread (1.5-2 cm).

CTV60 also will include the ipsilateral pathologically positive hemineck (if both sides of the neck are proven pathologically positive, CTV60 will include both sides). This generally means encompassing nodal levels 2a, 3, and 4 for all cases. Nodal levels 1, 2b, 5a, and 5b are included in CTV60 in selected circumstances. For example, level 1 must be included for oral cavity cancer but is not mandatory for larynx cancer. Level 5a can be included for oropharynx cancer but is not mandatory for larynx cancer.

CTV45-56: This will include all other regions felt to be at risk for harboring microscopic cancer that do not meet the criteria for CTV60. For example, this would apply to the contralateral hemineck being irradiated electively for base of tongue cancer. This volume should not approach the skin < 5 mm. This volume will receive approximately 1.65-1.85 Gy per day.

CTV66-70 Optional: This may be defined at the discretion of the treating radiation oncologist. This would include a region or regions felt to be at especially high risk for recurrence (e.g., an area of very close margin of resection). Note: This area will be receiving a daily fraction size of 1.8-2.2 Gy and thus, the volume of CTV66 should be kept as small as possible.

Planning Target Volumes (PTVs): In general, the PTV should not go outside of the skin surface; if it does exceed the skin surface, the application of bolus material over this portion of the PTV may be considered but is generally not recommended. It is also allowable to define two PTV's for a given CTV: 1) PTV Planning, which extends beyond the skin surface and is used for planning treatment segments; and 2) PTV Evaluation, which does not reach the skin surface within 2 mm and is used for evaluation of the dose volume histogram to determine if treatment goals have been met. Dental artifacts or other artifacts can be subtracted.

PTV Expansion

The minimum CTV-to-PTV expansion is 2.5 mm (a larger expansion may be necessary for a target volume subject to significant intra-fraction variability, such as the non-immobilized oral tongue). In general, the CTV-to-PRV expansion (with IGRT) should not exceed 5 mm.

Treatment Planning and Delivery

The entire clinical target volume (CTV) [upper and lower neck and primary tumor bed] is irradiated with IMRT. There is no match line between upper and lower portions of the regions at risk. In this technique, limiting radiotherapy dose to organs at risk (OARs), e.g., the cervical esophagus, is entirely achieved by inverse treatment planning via IMRT algorithms. This technique in general is appropriate for irradiation of cancers of the larynx and/or oral/pharyngeal cancers that involve the hypopharynx.

Dose to Supraclavicular Nodal Region

Regardless of whether technique 1 (Match) or technique 2 (No Match) is used, the dose to the supraclavicular nodal region may be limited to 50 Gy if level 4 nodes were dissected and found to be negative or in the case of oral cavity cancer with level 3 nodes dissected and found to be negative.

IMRT Dose Prescription to PTVs

The starting radiotherapy dose will be 60 Gy in 2 Gy once-daily fraction size 5 days a week. For inverse planning IMRT, the goal is for 95% of the PTV60 to receive ≥ 2 Gy with a minimum dose (cold spot) of no less than 45 Gy. It is recognized that portions of the PTV60 close to the skin may receive significantly less than 56 Gy. If negative margins are not achieved, the PTV dose to the high risk region could be increases to a range between 66Gy and 70 Gy in 2 to 2.2 Gy per fraction at the discretion of the treating radiation oncologist.

Definition of Normal Tissues/Organs at Risk (OARs)

Spinal Cord: The cord begins at the cranial-cervical junction (i.e., the top of the C1 vertebral body). Superior to this is brainstem and inferior to this is cord. The inferior border of the spinal cord is at approximately T3-4 (i.e., just below the lowest slice level that has PTV on it). The spinal cord shall be defined based on the treatment planning CT scan. In addition, however, a Planning Risk Volume (PRV) spinal cord shall be defined. The PRVcord = cord + 5 mm in each dimension. This is irrespective of whether or not IGRT is used.

Brainstem: The inferior most portion of the brainstem is at the cranial-cervical junction where it meets the spinal cord. For the purposes of this study, the superior most portion of the brainstem is approximately at the level of the top of the posterior clinoid. The brainstem shall be defined based on the treatment planning CT scan. In addition, however, a Planning Risk Volume (PRV) brainstem shall be defined. The PRV brainstem = brainstem + 3 mm in each dimension.

Lips and Oral Cavity: These should be contoured as 2 separate structures as the goal is to keep the lip dose much lower than the oral cavity dose. The definition of lips is self-explanatory. For non-oral cavity cancers, the oral cavity will be defined as a composite structure consisting of the anterior ½ to 2/3 of the oral tongue/floor of mouth, buccal mucosa, and palate. For oral cavity cancers, the oral cavity will be defined as the subset of this composite structure that does not overlap with PTV.

Parotid Glands: Parotid glands will be defined based on the treatment planning CT scan. Parotid gland volume will not include any portion of any of the CTVs, although they can overlap the PTVs.

OARpharynx: This will be defined as the "uninvolved" posterior pharyngeal wall plus adjacent constrictor muscles. This extends from the superior constrictor region (the inferior pterygoid plates level) to the cricopharyngeal inlet (posterior cricoid cartilage level). This should not overlap the PTVs.

Cervical Esophagus: This will be defined as a tubular structure that starts at the bottom of OARpharynx and extends to the thoracic inlet.

Glottic/Supraglottic Larynx (GSL): Obviously, for patients who have had a total laryngectomy, this structure is not applicable. This will be defined as a "triangular prism shaped" volume that begins just inferior to the hyoid bone and extends to the cricoid cartilage inferiorly and extends from the anterior commissure to include the arytenoids. This includes the infrahyoid but not suprahyoid epiglottis.

Mandible: This includes the entire bony structure of the mandible from TMJ through the symphysis. It is recognized that for oral cavity cancers, this may overlap with CTVs and PTVs.

Unspecified Tissue Outside the Targets: This will be defined as tissue located between the skull base and thoracic inlet that is not included in either the target volumes or the normal tissues described above.

IMRT Dose Constraints to Normal Structures

Spinal Cord: The PRVcord (as defined above) should not exceed 48 Gy to any volume in excess of 0.03 cc (approximately 3 mm x 3 mm x 3 mm). The spinal cord PRV should not exceed 50 Gy to any volume in excess of 0.01 cc. In treatment planning, the spinal cord PRV should be given the highest priority.

Brainstem: The PRVbrainstem (as defined above) should not exceed 52 Gy to any volume in excess of 0.03 cc (approximately 3 mm x 3 mm x 3 mm). In treatment planning, the PRV brainstem should be given less priority than the PRVcord but more priority than the other critical structures listed below.

Lips: Reduce the dose as much as possible. The mean dose should be < 20 Gy. For non-oral cavity cancers, the maximum dose will be < 30 Gy. For oral cavity cancers, the maximum dose will be < 50 Gy.

Oral Cavity: Reduce the dose as much as possible if non-oral cavity primary. For non-oral cavity cancers, the mean dose should be < 30 Gy. Efforts should be made to avoid hot spots (> 66 Gy) within the oral cavity, particularly for non-oral cavity cancers.

Parotid Glands: In most cases, it will be easier to spare one parotid than the other. The treatment planning goal will be for this individual parotid gland to receive a mean dose of < 26 Gy. Additional planning goals may include: 1) At least 50% of one parotid will receive < 30 Gy; and/or 2) At least 20 cc of parotid tissue (from the combination of both glands) will receive < 20 Gy.

OARpharynx: Reduce the dose as much as possible. Some recommended (but not mandatory) treatment goals include: 1) No more than 33% of the OARpharynx exceeds 50 Gy; 2) Mean dose < 45 Gy; 3) No more than 15% of the OARpharynx exceeds 60 Gy.

Cervical Esophagus: Reduce the dose as much as possible. For oral or oropharyngeal cancer, some recommended (but not mandatory) treatment goals include: 1) No more than 33% of the esophagus exceeds 45 Gy; 2) Mean dose < 35 Gy; 3) No more than 15% of the esophagus exceeds 54 Gy. For larynx cancer, higher doses are expected and permitted. Some recommended doses (but not mandatory) treatment goals include: 1) No more than 33% of the esophagus exceeds 50 Gy; 2) Mean dose < 45 Gy; 3) No more than 15% of the esophagus exceeds 60 Gy.

Glottic and Supraglottic larynx (GSL): Reduce the dose as much as possible. In patients with resected oral or oropharyngeal carcinoma, it is recommended that the dose to the larynx should be kept < 45 Gy whenever feasible.

Mandible: Reduce the dose as much as possible. It is recognized that particularly for oral cavity cancers, significant portions of the mandible will overlap the CTVs and/or PTVs; however, hot spots within the mandible should be avoided. It is recommended that maximum dose within the mandible be < 70 Gy.

Cochlea: Reduce the dose as much as possible; maximum dose < 45 Gy.

Unspecified Tissue Outside the Targets: For the typical case in which there is no CTV66, no more than 5% of unspecified tissue can receive greater than 58 Gy and no more than 1% or 1cc of unspecified tissue can receive 64 Gy or more. When a boost is used to increase the dose to high risk regions to as much as 66 Gy, these numbers can be increased. In this case, no more than 5% of the unspecified dose should exceed the level of the boost dose, and no more than 1% or 1 cc should exceed the boost dose value plus 10%.

Prioritization for IMRT Planning

1. Spinal Cord
2. Brainstem
3. PTV60
4. PTV56 (if applicable)
5. PTV66 (if applicable)
6. a. OAR pharynx
   b. Parotid gland contralateral to primary tumor site
7. a. GSL
   b. Esophagus
8. a. Lips
   b. Oral Cavity
9. a. Parotid gland ipsilateral to primary tumor site
   b. Mandible
10. Unspecified tissue outside the targets

Critical Structures

Standard Name	Description	Detailed Description
CTV_6000	Primary Tumor Bed plus involved nodes. **Required**	
CTV_5600	At risk regions **Required when applicable**	
CTV_6600	Optional higher dose region; should be kept small as possible. **Required when applicable**	
PTV_6000	CTV-PTV 5 mm margin without IGRT; 2.5 mm with Daily IGRT. **Required**	
PTV_6000-08	PTV_6000, not including portion of PTV_6000 near (<8 mm) skin	

The following table outlines the naming of the various normal and critical structures.

	**Required**	
PTV_5600	CTV-PTV 5 mm margin without IGRT; 2.5 mm with Daily IGRT. **Required when applicable**	
PTV_5600-08	PTV_5600, not including portion of PTV_5600 near (<8 mm) skin **Required when applicable**	
PTV_6600	CTV-PTV 5 mm margin without IGRT; 2.5 mm with Daily IGRT. **Required when applicable**	
PTV_6600-08	PTV_6600, not including portion of PTV_6600 near (<8 mm) skin **Required when applicable**	
NonPTV6000	External minus PTV_6000 **Required**	
SpinalCord	Spinal Cord **Required**	
SpinalCord_05	Planning risk Volume of 5 mm **Required**	
BrainStem	Brain Stem **Required**	
BrainStem_03	Planning Risk Volume of 3 mm **Required**	
Parotid_L	Left Parotid **Required**	
Parotid_R	Right Parotid **Required**	
OralCavity	Oral Cavity **Required**	
Lips	Lips **Required**	
Mandible	Mandible **Required**	
Pharynx	Uninvolved posterior pharyngeal wall plus adjacent constrictor muscles; should not include PTVs **Required**	
Esophagus_Up	Cervical Esophagus **Required**	
LarynxGSL	Glottic/Supraglottic larynx **Required**	
External	External border of patient used to define Unspecified Tissue. **Required**	

	Per Protocol	Minor Variation	Major Variation
Total RT dose to PTV_6000 (to 95%) of PTV_6000)	60-64 Gy	58-60 or 64-66 Gy	<58 or > 66 Gy
Minimum dose ("cold spot" within PTV_6000, not including portion of PTV_6000 near (<8 mm) skin)	56-60 Gy	54-56 Gy	< 54 Gy
Maximum dose ("hot spot") within PTV_6000*	< 70 Gy	70-72 Gy	> 72 Gy
Maximum dose ("hot spot" outside of PTV_6000)	< 66 Gy	66-70 Gy	> 70 Gy
Total RT dose to PTV_5600 (to 95%) of PTV_5600)	56-60	54-56 or 60-62	<54 or >62
Minimum dose ("cold spot" within PTV_5600, not including portion of PTV near (<8 mm) skin)	52-56	50-52	<50
Total RT dose to PTV_6600 (to 95%) of PTV_6600)	66-70	64-66 or 70-72	<64 or >72
Minimum dose ("cold spot" within PTV_6600, not including portion of PTV near (<8 mm) skin)	62-66	60-62	<60
Definition of CTV_6000	Based on case review by study chair		
Definition of PTV_6000	Based on case review by study chair.		
Total RT dose to SpinalCord_05(0.03 cc)	< 48 Gy	48-50 Gy	> 50 Gy
Total RT dose to SpinalCord_05(0.01 cc)	< 50 Gy	50-52 Gy	> 52 Gy
Definition of SpinalCord_05	Based on case review by study chair		
Overall RT treatment time	< 45 days	46-50 days (without a medically appropriate indication for delay).	> 50 days (without a medically appropriate indication for delay).
Non-Medically Indicated Treatment Interruptions	0-2	2-4	>4

*Not including the region of PTV60 that falls within PTV66 (if applicable)

6.7.6 Compliance Criteria

Treatment breaks must be clearly indicated in the treatment record along with the reason(s) for the treatment break(s). Treatment breaks, if necessary, ideally should not exceed five treatment days at a time and ten treatment days total. Treatment breaks should be allowed only for resolution of severe acute toxicity and/or for intercurrent illness and not for social or logistical reasons. Any treatment break(s) exceeding two treatment days for reasons other than toxicity/illness will be considered a protocol deviation.

6.7.7 Radiation therapy monitoring

All patients will be seen weekly by their radiation oncologist during therapy. Any observations regarding radiation reactions will be recorded.

The descriptions and grading scales found in the revised NCI Common Terminology Criteria for Adverse Events (CTCAE), version 4.03 will be utilized for grading all adverse events. All appropriate treatment areas should have access to a copy of the CTCAE, v. 4.03. A copy of the CTCAE, v. 4.03 can be downloaded from the CTEP web site (http://ctep.cancer.gov).

Grade 3 therapy-induced mucositis and/or dysphagia are expected to develop in about one third to one half of patients. Nutritional evaluation prior to the initiation of therapy for a prophylactic gastrostomy (PEG) tube placement is highly recommended. Placement of a feeding tube should be recorded on the appropriate case report form (see Section 12.1), as should use of a feeding tube during and after treatment (e.g., greater than or less than 50% of nutrition by tube). Other common radiation adverse events include: fatigue, weight loss, regional alopecia, xerostomia, hoarseness, transient ear discomfort, dysgeusia, and skin erythema and desquamation within the treatment fields.

Less common long-term treatment adverse events include: hypothyroidism, loss of hearing, chronic swallowing dysfunction requiring permanent feeding tube, and cervical fibrosis. Much less common radiation adverse events include: mandibular osteoradionecrosis (< 5% incidence with attention to the dental recommendations provided in Appendix IV), and cervical myelopathy (< 1% with restriction of spinal cord dose to ≤ 45 Gy). Only DLTs that result in surgical delay beyond the 8 weeks will constitute a DLT.

6.8 Surgery

Following neoadjuvant durvalumab with SBRT, patients will receive surgery consistent with the NCCN guidelines.

6.8.1 Resection of the primary tumor

Surgeons performing procedures must be board certified otolaryngologist-head and neck surgeons at their operating hospitals. The surgery should, when possible, provide complete removal of the primary lesion with negative gross margins. If not possible, and an R1 or R2 resection is done, then adjuvant radiation dosing will be modified as described under the radiation section. Documentation of margins by cryosection intraoperatively is required. En bloc resection is the preferred surgical approach. Surgeons should aim to achieve a 1 cm gross visual margin of normal tissue around tumor. Goal for final pathologic negative margin designation is 3 mm.

Standard terminology to designate margins by cryosection will consist of, at minimum, a deep margin, and four quadrants: anterior, posterior, medial, lateral. Intraoperatively, surgeons must submit oriented specimens consisting of the 4 quadrants named above, plus a deep margin. A positive intraoperative cryosection margin is defined as invasive SCC or carcinoma in situ (CIS) at the margin of resection. Additional submitted margin tissue (i.e. a new margin) found to be histopathologically free of disease on new cryosection will be considered a pathologically negative margin on intraoperative cryosection analysis.

Final pathologic analysis of margins may identify a positive margin found on permanent histopathologic sections after cryosection analysis was read as negative. This will be designated as a close margin negative resection.

For SCC of the tonsil, simple tonsillectomy will not be performed. The extent of En bloc resection will include a margin of soft palate, constrictor muscle, anterior and posterior tonsil pillars, base of tongue with similar goals of 1cm gross visual negative margin and 2 mm microscopic margin.

Intraoperatively, surgeons must submit oriented specimens consisting of 4 quadrant margins plus deep margin to the pathologist for cryosection. A positive margin found on final pathologic analysis after negative frozen sections would be classified as a close negative margin resection (R0). It is recommended to perform photo documentation of the specimen by the pathologist after these procedures, using high-resolution digital photography with annotation. If the surgeon obtains additional margins from the patient, the "new" margins should refer back to the geometric orientation of the resected tumor specimen. Additionally, the final surgical pathology report should point out that this "new" margin represents the final margin of resection in addition to its histologic status.

Generally accepted techniques should be utilized in the reconstruction and closure of surgical defects at the discretion of the surgeon, including the decisions regarding the need for free tissue transfer.

6.8.2 Neck Dissection

Neck dissection involves removal of lymph nodes from affected, or potentially affected, areas of the neck based upon the location of primary tumor. Anatomic levels of the neck are described below in section 6.10.4. The levels of the neck at risk for metastatic nodal involvement is well accepted based upon primary tumor site and embryology of lymphatic drainage. Surgical intervention in this study will include neck dissection as deemed appropriate based upon tumor site and stage per the operating surgeon. The neck dissection should be oriented or separately partitioned for pathologic submission in order to identify levels of lymph nodes encompassed by the dissection.

Pathologic assessment of extracapsular extension (ECE):

- Lymph nodes ≤ 2 cm in greatest dimension are considered adequately assessed with one section.
- Multiple lymph nodes ≤ 2 cm in greatest dimension may be submitted in a single cassette.
- Lymph nodes > 2 cm in greatest dimension require multiple sections (and possibly multiple cassettes); specifically, one section per 1 cm should be submitted. Sections should include the edge of the lymph node that interfaces with surrounding fibroadipose tissue.

6.8.3 Neck lymph node levels

Neck lymph node (LN) levels are defined as per the American Head and Neck Society and the American Academy of Otolaryngology-Head and Neck Surgery [45].
Submental (sublevel IA): This group consists of LNs within the triangular boundary of the anterior belly of the digastric muscles and the hyoid bone.

Submandibular (sublevel IB): This group consists of LNs within the boundaries of the anterior and posterior belly of the digastric muscles, the stylohyoid muscle, and the body of the mandible. It includes the preglandular and the postglandular nodes and the prevascular and postvascular nodes. The submandibular gland is included in the specimen when the lymph nodes within the triangle are removed.

Upper jugular (level IIA and IIB): This group consists of LNs located around the upper third of the internal jugular vein and adjacent spinal accessory nerve (SAN) extending from the level of the skull base (above) to the level of the inferior border of the hyoid bone (below). The anterior (medial) boundary is the stylohyoid muscle (the radiologic correlate is the vertical plane defined by the posterior surface of the submandibular gland) and the posterior (lateral) boundary is the posterior border of the sternocleidomastoid muscle (SCM). Sublevel IIA nodes are located anterior (medial) to the vertical plane defined by the SAN. Sublevel IIB nodes are located posterior (lateral) to the vertical plane defined by the SAN.

Middle jugular (level III): This group consists of LNs located around the middle third of the internal jugular vein extending from the inferior border of the hyoid bone (above) to the inferior border of the cricoid cartilage (below). The anterior (medial) boundary is the lateral border of the sternohyoid muscle, and the posterior (lateral) boundary is the posterior border of the SCM. Included in this group is the jugulo-omohyoid node, which lies immediately above the superior belly of the omohyoid muscle as it crosses the internal jugular vein.

Lower jugular (level IV): This group consists of LNs located around the lower third of the internal jugular vein extending from the inferior border of the cricoid cartilage (above) to the clavicle (below). The anterior (medial) boundary is the lateral border of the sternohyoid muscle, and the posterior (lateral) boundary is the posterior border of the SCM.

Posterior triangle group (includes sublevels VA and VB): This group is composed predominantly of LNs located along the lower half of the SAN and the transverse cervical artery. The supraclavicular nodes are also included in posterior triangle group. The superior boundary is the apex formed by convergence of the SCM and trapezius muscles, the inferior boundary is the clavicle, the anterior (medial) boundary is the posterior border of the SCM, and the posterior (lateral) boundary is the anterior border of the trapezius muscle. Sublevel VA is separated from sublevel VB by a horizontal plane marking the inferior border of the anterior cricoid arch. Sublevel VA includes the SAN, whereas sublevel VB includes the nodes following the transverse cervical vessels and the supraclavicular nodes with the exception of the Virchow node, which is located in level IV.

The surgical landmark that defines the lateral boundary of levels II, III, and IV and the corresponding medial boundary of the posterior triangle (level V) is the plane that parallels the sensory branches of the cervical plexus.

Anterior compartment group (level VI): LNs in this compartment include the pretracheal and paratracheal nodes, precricoid (Delphian) node, and the perithyroidal nodes including the LNs along the recurrent laryngeal nerves. The superior boundary is the hyoid bone, the inferior boundary is the suprasternal notch, and the lateral boundaries are the common carotid arteries.

6.8.4 Surgical Compliance

Deviations deemed unacceptable include those that affect patient safety/outcome, which will result in study suspension from further patient recruitment include:

- Positive margin rate exceeding 20% (assessed every 5 cases accrued)
- Inadequate nodal dissection (<5 nodes removed) rate exceeding 20% (assessed every 5 cases accrued)
- Postoperative bleeding requiring return to the operating room for control, exceeding 20% of submitted patients (assessed every 5 patients accrued)
- Failure to ligate feeding arteries at time of surgery

7. RESTRICTIONS DURING THE STUDY AND CONCOMITANT TREATMENT(S)

7.1 Restrictions during the study

The following restrictions apply while the patient is receiving study treatment and for the specified times before and after:

Female patient of child-bearing potential

- Females of childbearing potential who are sexually active with a non-sterilized male partner must use at least 1 **highly** effective method of contraception (Table 2) from the time of screening and must agree to continue using such precautions for 180 days after the last dose of durvalumab + any drug combination therapy or 90 days after the last dose of durvalumab monotherapy. Non-sterilized male partners of a female patient of childbearing potential must use male condom plus spermicide throughout this period. Female patients should also refrain from breastfeeding throughout this period.

Male patients with a female partner of childbearing potential

- Non-sterilized males who are sexually active with a female partner of childbearing potential must use a male condom plus spermicide from screening through 180 days after receipt of the final dose of durvalumab + any drug combination therapy or 90 days after receipt of the final dose of durvalumab monotherapy. Not engaging in sexual activity is an acceptable practice; however, occasional abstinence, the rhythm method, and the withdrawal method are not acceptable methods of contraception. Male patients should refrain from sperm donation throughout this period.
- Female partners (of childbearing potential) of male patients must also use a highly effective method of contraception throughout this period (Table 2).

Females of childbearing potential are defined as those who are not surgically sterile (i.e., bilateral salpingectomy, bilateral oophorectomy, or complete hysterectomy) or post-menopausal.
Women will be considered post-menopausal if they have been amenorrheic for 12 months without an alternative medical cause. The following age-specific requirements apply:

- Women <50 years of age would be considered post-menopausal if they have been amenorrheic for 12 months or more following cessation of exogenous hormonal treatments and if they have luteinizing hormone and follicle-stimulating hormone levels in the post-menopausal range for the institution or underwent surgical sterilization (bilateral oophorectomy or hysterectomy).

- Women ≥50 years of age would be considered post-menopausal if they have been amenorrheic for 12 months or more following cessation of all exogenous hormonal treatments, had radiation-induced menopause with last menses >1 year ago, had chemotherapy-induced menopause with last menses >1 year ago, or underwent surgical sterilization (bilateral oophorectomy, bilateral salpingectomy or hysterectomy).

Highly effective methods of contraception, defined as one that results in a low failure rate (i.e., less than 1% per year) when used consistently and correctly are described in Table 2. Note that some contraception methods are not considered highly effective (e.g., male or female condom with or without spermicide; female cap, diaphragm, or sponge with or without spermicide; non-copper containing intrauterine device; progestogen-only oral hormonal contraceptive pills where inhibition of ovulation is not the primary mode of action [excluding Cerazette/desogestrel which is considered highly effective]; and triphasic combined oral contraceptive pills).

Table 2. Highly Effective Methods of Contraception (<1% Failure Rate)

• Barrier/Intrauterine methods	• Hormonal Methods
• Copper T intrauterine device<br>• Levonorgestrel-releasing intrauterine system (e.g., Mirena®)[a]	• Implants: Etonogestrel-releasing implants: e.g. Implanon® or Norplant®<br>• Intravaginal: Ethinylestradiol/etonogestrel-releasing intravaginal devices: e.g. NuvaRing®<br>• Injection: Medroxyprogesterone injection: e.g. Depo-Provera®<br>• Combined Pill: Normal and low dose combined oral contraceptive pill<br>• Patch: Norelgestromin/ethinylestradiol-releasing transdermal system: e.g. Ortho Evra®<br>• Minipillc: Progesterone based oral contraceptive pill using desogestrel: Cerazette® is currently the only highly effective progesterone-based

[a] This is also considered a hormonal method

Blood donation

Patients should not donate blood while participating in this study, or for at least 90 days following the last infusion of durvalumab or 90 days after receipt of the final dose of durvalumab.

7.2 Concomitant treatment(s)

The Principal Investigator must be informed as soon as possible about any medication taken from the time of screening until the end of the clinical phase of the study (final treatment visit). Any concomitant medication(s), including herbal preparations, taken during the study, with the exception of those documented during the surgical resection hospitalization, will be recorded in the CRF.

Restricted, prohibited, and permitted concomitant medications are described in the following tables.

7.2.1 Permitted concomitant medications

Table 3. Supportive Medications

Supportive medication/class of drug:	Usage:
Concomitant medications or treatments (e.g., acetaminophen or diphenhydramine) deemed necessary to provide adequate prophylactic or supportive care, except for those medications identified as "prohibited," as listed above	To be administered as prescribed by the Investigator
Best supportive care (including antibiotics, nutritional support, correction of metabolic disorders, optimal symptom control, and pain management [including palliative radiotherapy to non-target lesions, etc])	Should be used, when necessary, for all patients
Inactivated viruses, such as those in the influenza vaccine	Permitted
Concurrent chemotherapy given adjuvantly per investigator choice and related supportive medications	Permitted

7.2.2 Excluded concomitant medications

Table 4. Prohibited Concomitant Medications

Prohibited medication/class of drug:	Usage:
Any investigational anticancer therapy other than those under investigation in this study	Should not be given concomitantly whilst the patient is on study treatment
mAbs against CTLA-4, PD-1, or PD-L1 other than those under investigation in this study	Should not be given concomitantly whilst the patient is on study treatment
Any concurrent chemotherapy, radiotherapy, immunotherapy, or biologic or hormonal therapy for cancer treatment other than those under investigation in this study	Should not be given concomitantly whilst the patient is on study treatment. (Concurrent use of hormones for non-cancer-related conditions [e.g., insulin for diabetes and hormone replacement therapy] is acceptable. Local treatment of isolated lesions, excluding target lesions, for palliative intent is acceptable [e.g., by local surgery or radiotherapy])

Prohibited medication/class of drug:	Usage:
Immunosuppressive medications including, but not limited to, systemic corticosteroids at doses exceeding 10 mg/day of prednisone or equivalent, methotrexate, azathioprine, and tumor necrosis factor-α blockers	Should not be given concomitantly, or used for premedication prior to the durvalumab infusions. The following are allowed exceptions:<br>• Use of immunosuppressive medications for the management of IP-related AEs,<br>• Short-term premedication for patients receiving adjuvant concurrent systemic therapy where the prescribing information for the agent requires the use of steroids for documented hypersensitivity reactions or emesis prevention<br>• Use in patients with contrast allergies.<br>• In addition, use of inhaled, topical, and intranasal corticosteroids is permitted.<br>*A temporary period of steroids will be allowed if clinically indicated and considered to be essential for the management of* non-immunotherapy related events experienced by *the patient (e.g., chronic obstructive pulmonary disease, radiation, nausea, etc).*
Drugs with laxative properties and herbal or natural remedies for constipation	Should be used with caution through to 90 days after the last dose of study treatment
EGFR TKIs	Should not be given concomitantly.<br>Should be used with caution in the 90 days post last dose of durvalumab.<br>Increased incidences of pneumonitis (with third generation EGFR TKIs) and increased incidence of transaminase increases (with 1st generation EGFR TKIs) has been reported when durvalumab has been given concomitantly.
Live attenuated vaccines	Should not be given through 30 days after the last dose of IP (including SoC)
Herbal and natural remedies which may have immune-modulating effects	Should not be given concomitantly unless agreed by the sponsor

8. STUDY PROCEDURES

8.1 Schedule of study procedures

Before study entry, throughout the study, and following study drug discontinuation, various clinical and diagnostic laboratory evaluations are outlined. The purpose of obtaining these detailed measurements is to ensure adequate safety and tolerability assessments. Clinical evaluations and laboratory studies may be repeated more frequently if clinically indicated. The Schedule of Study Assessments during the screening and treatment period is provided following the Protocol Synopsis.

All other scheduled assessments must be performed relative to the start of the dosing cycle such that all laboratory procedures, etc. required for dosing should be performed on the day of dosing.

For durvalumab adjuvant therapy

Patients may delay dosing under certain circumstances.

- Dosing may be delayed per Toxicity Management Guidelines, due to either an immune or a non-immune-related AE.
- If dosing must be delayed for reasons other than treatment-related toxicity, dosing will resume as soon as feasible
- Dosing intervals of subsequent cycles may be shortened as clinically feasible in order to gradually align treatment cycles with the schedule of tumor efficacy and PRO assessments. Subsequent time between 2 consecutive doses cannot be less than 22 days, based on the half-life of durvalumab (see current Investigator Brochures for durvalumab.)

8.1.1 Screening phase

Screening procedures will be performed up to 28 days before Day 1, unless otherwise specified. All patients must first read, understand, and sign the IRB/REB/IEC-approved ICF before any study-specific screening procedures are performed. After signing the ICF, completing all screening procedures, and being deemed eligible for entry, patients will be enrolled in the study. Procedures that are performed prior to the signing of the ICF (including imaging and pre-treatment biopsy) and are considered standard of care may be used as screening assessments if they fall within the 28-day screening window.

The following procedures will be performed during the Screening Visit:

- Informed consent
- Review of eligibility criteria
- Review of adverse events
- Medical history and demographics
- Complete physical exam
- ECOG Performance Status
- Vitals signs, including weight and height
- 12-lead ECG
- FACT-HN
- Pre-treatment tumor biopsy
- Review of prior/concomitant medications
- CT Simulation
- MRI neck with and without contrast
- PET/CT skull base to mid-thigh
- Clinical laboratory tests for:
    - Hematology (see Table 5)
    - Clinical chemistry (see Table 6)
    - TSH (if TSH is abnormal, total T3 and free T4 will be required)
    - Coagulation (PT, PTT, INR)
    - Creatinine Clearance

- Urine or serum pregnancy test (for women of childbearing potential only, to be performed within 72 hours of study entry)
- Hepatitis & HIV serologies
- Urinalysis (see Table 7)
- Biomarker & Immunogenicity analysis (to be performed within 7 days prior to or on D1 of study treatment)

8.1.2 Treatment phase

Procedures to be conducted during the treatment phase of the study are presented in the Schedule of Study Assessments.

On all treatment visits:

- Targeted physical exam
- ECOG Performance Status
- Review of concomitant medications
- Review of adverse events
- Vital Signs including weight

Patients will receive the first fraction of radiation on Day 1. Subsequent fractions of SBRT will be given on days 3 and 5. Subjects will receive the first dose (neo-adjuvant dose) of durvalumab between SBRT fractions 1 through 3. Patients will undergo surgical resection on or about week 3-6. Following surgery, patients will receive adjuvant radiation +/- concurrent systemic therapy if indicated. They will also receive adjuvant durvalumab q 4 weeks for up to 6 cycles or until progression, toxicity, or withdrawal from study. Study related blood draws, tumor assessments, and imaging will be collected as indicated on the Schedule of Study Assessments.

8.1.3 End of treatment

End of treatment is defined as the last planned dosing visit with Durvalumab. For patients who discontinue durvalumab prior to 6th adjuvant dose, end of treatment is considered the last visit where the decision is made to discontinue treatment. All required procedures may be completed within ±14 days of the end of treatment visit.

All patients treated with Durvalumab will be followed for survival up to 18 months from the end of treatment regardless of further treatments, or until the sponsor ends the study.

8.2 Description of study procedures

8.2.1 Medical history and physical examination, electrocardiogram, weight, and vital signs

Findings from medical history (obtained at screening) and physical examination shall be given a baseline grade according to the procedure for AEs. Increases in severity of pre-existing conditions during the study will be considered AEs, with resolution occurring when the grade returns to the pre-study grade or below.

Smoking status will be recorded for each patient. They will be categorized as either a Never Smoker (< 100 cigarettes in entire lifetime or never smoked cigarettes), Smoker ≤ 10 pack years, Smoker > 10 pack years.

Physical examinations will be performed on study days noted in the Schedule of Study Assessments. At screening, a complete physical examination will be performed and will include an assessment of the following (as clinically indicated): general appearance, respiratory, cardiovascular, abdomen, skin, head and neck (including ears, eyes, nose and throat), lymph nodes, thyroid, musculoskeletal (including spine and extremities), genital/rectal, and neurological systems and height. During subsequent study visits a directed physical exam will be completed.

Resting 12-lead ECGs will be recorded at screening and as clinically indicated throughout the study. ECGs should be obtained after the patient has been in a supine position for 5 minutes and recorded while the patient remains in that position. ECGs will be recorded singularly. In case of clinically significant ECG abnormalities, including a QTcF value >470 ms, 2 additional 12-lead ECGs should be obtained over a brief period (eg, 30 minutes) to confirm the finding. Situations in which ECG results should be reported as AEs are described in Section 10.1.1.

Vital signs (blood pressure [BP], pulse, temperature, and respiration rate) will be evaluated according to the Schedule of Study Assessments. Body weight will also be recorded once (pre-treatment) at each visit. Height will be collected at screening.

First infusion

On the first infusion day of durvalumab patients will be monitored and vital signs collected/recorded prior to, during and after infusion of IP as presented in the bulleted list below):

- Prior to the beginning of the infusion (measured once from approximately 30 minutes before up to 0 minutes [i.e., the beginning of the infusion])
- Approximately 30 minutes during the infusion (**halfway** through infusion)
- At the end of the infusion (approximately 60 minutes ±5 minutes)
- If the infusion takes longer than 60 minutes, then BP and pulse measurements should follow the principles as described above or be taken more frequently if clinically indicated. A 1-hour observation period is recommended after the first infusion of durvalumab.

Subsequent infusions

BP, pulse and other vital signs should be measured, collected/recorded prior to the start of the infusion. Patients should be carefully monitored, and BP and other vital signs should be measured during and post infusion as per institution standard and as clinically indicated.

8.2.2 Clinical laboratory tests

The following clinical laboratory tests will be performed (see the Schedule of Study Assessments for the time points of each test):

Table 5. Hematology Laboratory Tests

Basophils	Mean corpuscular volume
Eosinophils	Monocytes
Hematocrit	Neutrophils
Hemoglobin	Platelet count
Lymphocytes	Red blood cell count
Mean corpuscular hemoglobin	Total white cell count
Mean corpuscular hemoglobin concentration	

Table 6. Clinical Chemistry (Serum or Plasma) Laboratory Tests

Albumin	Glucose
Alkaline phosphatase	Lactate dehydrogenase
Alanine aminotransferase	Lipase[b]
Amylase[b]	Magnesium
Aspartate aminotransferase	Potassium
Bicarbonate	Sodium
Calcium	Total bilirubin[a]
Chloride	Total protein
Creatinine	Urea or blood urea nitrogen, depending on local practice
Gamma glutamyltransferase	Uric acid

[a] Tests for ALT, AST, alkaline phosphatase, and total bilirubin must be conducted and assessed concurrently. If total bilirubin is ≥2 × upper limit of normal (and no evidence of Gilbert's syndrome) then fractionate into direct and indirect bilirubin.

[b] It is preferable that both amylase and lipase parameters are assessed. For sites where only 1 of these parameters is routinely measured then either lipase or amylase is acceptable.

Table 7. Urinalysis Tests[a]

Bilirubin	pH
Blood	Protein
Glucose	Specific gravity
Ketones	Colour and appearance

[a] Microscopy should be used as appropriate to investigate white blood cells and use the high-power field for red blood cells

If a patient shows an AST or ALT ≥3xULN together with total bilirubin ≥2xULN, refer to Appendix 1 for further instructions on cases of increases in liver biochemistry and evaluation of Hy's Law. These cases should be reported as SAEs if, after evaluation, they meet the criteria for a Hy's law case or if any of the individual liver test parameters fulfill any of the SAE criteria. All patients should have further chemistry profiles performed at each follow-up assessment (12 weeks ± 2 weeks)

Any clinically significant abnormal laboratory values should be repeated as clinically indicated and recorded on the eCRF. Situations in which laboratory safety results should be reported as AEs are described in Section 10.3.5.

All patients with Grade 3 or 4 laboratory values at the time of completion or discontinuation from IP must have further tests performed until the laboratory values have returned to Grade 1 or 2, unless these values are not likely to improve because of the underlying disease.

8.2.3 Patient reported outcomes (PRO)

8.2.3.1 FACT H&N v4

The functional assessment of cancer therapy-head and neck version 4 (FACT H&N v4) instrument will be used to evaluate quality of life of patients treated in this study. The FACT H&N v4 includes an 11-item subscale of head and neck cancer specific problems. Internal consistency of these items has been validated and it has demonstrated sensitivity to change in clinical status. This is a self-administered instrument and will be given to enrolled patients by clinic nurses or other appropriately trained personnel. It will be administered to patients prior to treatment and as noted in the Schedule of Study Assessments.

8.3 Biological sampling procedures

8.3.1 Immunogenicity sampling and evaluation methods

Translational studies will require blood samples, initial biopsy, and tumor tissue at the time of surgery.

1. To establish a gene signature panel that can assess the response to RT-anti-PDL1 response and treatment outcome. This will be done using fresh tissue (multi-spec flow), formalin fixed tissue (RNA seq and multi-spec immunofluorescence), and peripheral blood flow cytometry and plasma/serum cytokines.
2. Compare change in anti-PDL1 and T cell infiltration pattern and intensity before and after RT and anti-PDL1 by immunohistochemistry (formalin fixed tissue).
3. To evaluate the impact of other mutations on immune response and treatment outcome (DNA seq from formalin fixed tissue or frozen tissue or circulating tumor DNA).
4. Blood samples to evaluate biomarkers of immune response as well as for evaluation of circulating auto-antibodies. Blood will be collected prior to treatment, at time of surgery, at end of treatment, and at the 6 month follow-up.
5. Tumor tissue taken at the time of initial biopsy and at time of resection will be profiled for tumor infiltrating lymphocytes; activation markers and antigen specific TCR utilization/diversity will be evaluated for additional checkpoint targets. This will be done using fresh tissue (multi-spec flow), formalin fixed tissue (RNA seq and multi-spec immunofluorescence),
6. On long-term follow-up, tumor antigen specific T lymphocyte memory populations will

be monitored for representation and robustness in in-vitro stimulation assays and ex vivo TCR alpha and beta chain gene sequencing as potential biomarker of continued anti-tumor activity. This will be conducted on peripheral blood samples.

8.3.2 Biomarker sampling and evaluation methods

Analysis of gene expression of RNA levels and entire genome sequences. We will employ multiplexed-gene expression profiling to conduct paired-end RNA-sequencing on tumor biopsies/samples before and after treatment with RT and anti-PD-L1 using the BioSpyder platform. Such gene expression analysis of RNA levels incorporates a large amount of data that can assist in the identification of prognostic and predictive relevance and can be used to characterize both tumor and immune cells. Sequencing of whole exomes (WES) and entire genome (WGS) using next generation sequencing (NGS) will also be performed on tumor biopsies to evaluate tumor somatic mutation load and assess whether high tumor mutation load predicts response to RT+anti-PD-L1. The germline DNA isolated from blood will be used to filter out genomic variants.

***Phenotypic analysis of T cells and assessment of intracellular and circulating cytokines*.** We will use multiplex mass flow cytometry to analyze phenotypic changes, functional response of cytokine production, and activation status of tumor infiltrating lymphocytes (TILs), circulating T cells (especially CD8 and PD-1 expression), and peripheral blood mononuclear cells (PBMCs). This will employ the use of protein activation cocktail stimulation, assessment by phospho-flow, and intracellular cytokine flow. In addition to mass cytometry for assessment of intracellular and circulating cytokines, we will also utilize cytokine mesoscale assay on protein lysates of fresh tumor tissue and plasma samples. We will utilize TCR gene expression analyses to determine changes in the repertoire of alpha and beta chains.

***Examination of intratumoral immune cell infiltration*.** Tumor infiltrating cells will be characterized using a number of markers (CD3, CD4, CD25, CD8, CD20, CD68 and FoxP3) by multiplex IF (VECTRA; Perkin Elmer Vectra 3) on formalin-fixed paraffin embedded (FFPE) tumor tissues. The new Multiplexed Ion Beam Imaging Platform (MIBI) will further strengthen these tissue analyses. The MIBI allows for simultaneous multiplex detection of up to 100 analytes, including proteins and specific mRNAs, through secondary ion mass spectrometry and metal isotope labeled antibodies. To maximize tissue usage, we will utilize tissue microarrays (TMAs), which can include tissue punches from many different patients on one slide and/or several punches from one tissue block. The HIMSR core laboratory will perform the high parameter multiplex slide staining, image acquisition, and analysis.

Tissue and blood sample repository
As part of the study, a tissue and blood sample repository will be created. The objective of this tissue sample repository will be to provide material for future evaluations of other relevant biomarkers that may be associated with clinical outcomes. A written informed consent will be obtained from patients enrolled in this study so that these remaining samples may be analyzed in the future for biomarkers not described in this protocol. Banking of these remaining samples will be optional. If a subject withdraws consent to the use of donated samples, the samples will be disposed of/destroyed, and the action documented.

8.3.3 Blood collection

The total volume of blood that will be drawn from each patient in this study is as follows:

Table 8. Volume of Blood to be Drawn from Each Patient

Assessment		Sample volume (mL)	No. of samples	Total volume (mL)
**Safety**	**Clinical chemistry**	1	1/patient/time point	10
	**Hematology**	1	1/patient/time point	10
Immune Monitoring; Cytokine Analysis; biomarker[1]		30-40	1/patient/time point	30-40
[1]Total 3-4 tubes per patient. See laboratory collection forms for tube details.				

We will collect the plasma and peripheral blood. Plasma will be collected to measure circulating cytokines. Whole blood will be stimulated with LPS or cytokines and preserved using Smart Tube system. Immune cell distribution, phenotypes, and cytokine production will be analyzed by mass cytometry (cyTOF) and compared to unstimulated controls.

Participants will be asked to provide blood prior to initiating treatment, during treatment, and after completion of treatment to evaluate changes in immune cell frequency and activation status by cyTOF. Peripheral blood samples for CyTOF will be cryopreserved, with samples subjected to a barcoding approach at time of study completion, to allow simultaneous assessment of immune cell frequencies across patient samples and to avoid batch effects of analysis.

Blood will be collected at scheduled intervals according to the Schedule of Study Assessments table, using three to four (3 to 4) vacutainer tubes for plasma, buffy coat, and blood cells separation and storage. Samples for immune assays will be de-identified and forwarded to the HIMSR core laboratory to be processed within 24 hours of collection. Any samples remaining after the described experiments are completed will be stored at the Biorepository Core Facility.

8.3.4 Tissue collection

Tissue analysis:
We will analyze tissue from the baseline biopsy and from surgery with CyTOF, RNA -seq, NGS, and PDL1 expression and T-cell infiltration by IHC. FFPE tissue will be used for immunohistochemical/ immunofluorescence analysis. FFPE tissue will be used for RNA isolation and RNA-seq. Fresh tissue will be collected for cell harvesting for flow cytometric analysis of intratumoral immune infiltrates and activation status. Fresh tissue analysis should be prioritized as a minimum of a large marble size piece of tissue is needed (~12x12mm). If and only if adequate tissue is collected for fresh tissue shall tissue by other methods be collected.

Fresh tumor biopsies:
For baseline biopsy samples, fresh and formalin tissue will be collected. In addition, archival tissue from the patient’s initial diagnosis will be requested and submitted. Baseline tissue

samples from the primary tumor will be collected for histopathological examination, evaluation of CD8+ tumor infiltrating lymphocytes, and comprehensive immune-profiling. Additional lymph node metastasis should be sampled in cases where it was grossly involved by cancer at the presentation (preoperatively). Every effort will be made to obtain adequate tissue from the tumor to allow direct comparison with the surgical specimen. Samples of the primary tumor will be obtained by an outpatient punch-biopsy or large-cup forceps biopsy performed under local anesthesia, preferably with a *minimum* of 1cm diameter (marble size). Ultrasound-guided core biopsy will be performed to sample metastatic lymph nodes if possible. The procedure is done in the outpatient setting using small core needles (18 or 20 gauges). The specimens should be collected and processed according to the study laboratory manual.

The specimens resected during surgery will be used for routine histopathological diagnosis in accordance with Institutional Standard Operating Procedures. The recommended procedure for obtaining the tissue specimens is as follows: the primary tumor should be identified at the time of surgery. Procurement of tissue for research is done ex vivo. FFPE blocks from the formalin sample collected for diagnostic purposes will also be requested following completion of pathology review.

The surgical specimens will be evaluated for pathologic staging (pTNM) according to the AJCC 8th edition (or per pathology report), pathologic complete response, quantification of percentage of viable tumor cells, status of resection margins, number of lymph nodes removed at each level, number of lymph nodes positive for cancer at each level, presence of extra- capsular nodal spread, perineural and vascular invasion. To determine the pathologic response, specimens will be grossed and evaluated by the head and neck pathologist. The entire specimen measurement and the size of visible residual tumor will be documented. At least 1 block/1 cm of tumor will be submitted (average of 5-10 blocks/patient). All slides prepared from blocks taken from each specimen will be reviewed by the head and neck pathologist, as well as slides from each dissected lymph node.

If a patient withdraws consent to the use of donated samples, the samples will be disposed of/destroyed, and the action documented. As collection of the biological samples is an integral part of the study, then the patient is withdrawn from further study participation. The Principal Investigator:

- Ensures that biological samples from that patient that are stored in the Biorepository Core Facility, are immediately identified, disposed of /destroyed, and the action documented.
- Ensures that the patient is informed about the sample disposal.

9. DISEASE EVALUATION AND METHODS

9.1.1 Efficacy Assessment

Tumor response to neoadjuvant therapy (durvalumab + SBRT) will be assessed by pathology review of the surgical specimen. Response will be labeled as complete pathologic remission, microscopic residual tumor (only scattered foci of residual tumor cells) or macroscopic residual tumor.

The method of assessment of disease status at baseline will be MRI neck with and without contrast *and* PET-CT skull base to mid-thigh. The baseline assessment should be performed no more than 28 days before SBRT. Efficacy for all patients will be assessed by objective tumor assessment by a repeat PET-CT scan (preferable) and/or an MRI neck with and without contrast for treatment response evaluation if a PET is not possible or if an MRI is needed by the surgical team. Response will be assessed based on either the PET by examining the metabolic SUV signal of the tumor (preferable if available) or gross tumor volume changes by MRI as dictated by neuroradiology if only an MRI is done. At about 3 months from the end of adjuvant radiation (if given), or for those that do not receive adjuvant radiation, at about 3 months from the end of Cycle 2 adjuvant durvalumab, all patients will undergo post-treatment PET-CT skull base to mid-thigh and MRI neck to mid-thigh with and without contrast. It is recommended that all radiology evaluations be done on the same machine when possible.

For patients who discontinue treatment due to toxicity in the absence of confirmed objective progression, objective tumor assessments per the scheduled study assessments should be continued every 12 weeks for 18 months.

Following confirmed progression, patients should continue to be followed up for survival every 12 weeks for 18 months

Patients who achieve and maintain disease control (CR) through to the end of the treatment period will continue with follow-up every 12 weeks for 18 months as detailed in the Schedule of Study Assessments.

Confirmation of progression guidelines are set for the following reasons:

- For patient management and treatment decisions
- In the absence of significant clinical deterioration on follow up, to promote the collection of additional scans after the first radiologic progression, preferably by PET-CT (MRI will be accepted if PET-CT cannot be obtained). Suspected clinical progression in the follow-up period after neoadjuvant Durva-RT will be confirmed at the time of surgery by tissue assessment. Suspected clinical progression in the adjuvant period after surgery and during Durva or Durva-RT or Durva alone, or on long-term follow up, that is detected by PET-CT or MRI must be confirmed with tissue biopsy that can demonstrate evidence of malignancy. The metabolic signal on the PET as defined by a board-certified radiologist and/or the MRI reading by a neuroradiologist, or evidence of gross disease on physical exam by a trial investigator will serve as the definition of clinical progression.

In the absence of significant clinical deterioration, treatment with study drug may continue between the initial assessment of progression and the scan to confirm progression. If the confirmation scan confirms progression, then the date of the prior scan with PD should be declared as the date of progression.

If progression is not confirmed, in the absence of significant clinical deterioration, then the patient should continue study drug and on-treatment assessments until the next PD which will also require a follow-up confirmation scan. **If the first PD is not confirmed by the immediate next scan, then the Investigator should not change the PD assessment of the first scan.**

If a patient discontinues treatment (and/or receives a subsequent anticancer therapy) prior to radiologic progression, then the patient should still continue to be followed until confirmed objective disease progression.

Following confirmed progression, patients should continue to be followed up for survival every 3 months (12 weeks) for up to 18 months as outlined for follow-up in the Schedule of Study Assessments.

10. ASSESSMENT OF SAFETY

Assessments will consist of monitoring and recording of AEs and serious AEs, physical examination, measurement of protocol-specific laboratory variables and vital signs, as well as other tests deemed important for this protocol. Circumstances in which these assessments should be reported as AEs are described below. All patients who have received at least one exposure to study drug will be evaluated for safety of the study drug. The Principal Investigator is responsible for ensuring that all staff involved in the study is familiar with the content of this section.

10.1.1 Safety parameters

10.1.1.1 Definition of adverse events

The International Conference on Harmonization (ICH) Guideline for Good Clinical Practice (GCP) E6(R1) defines an AE as:

Any untoward medical occurrence in a patient or clinical investigation patient administered a pharmaceutical product and which does not necessarily have a causal relationship with this treatment. An AE can therefore be any unfavorable and unintended sign (including an abnormal laboratory finding), symptom, or disease temporally associated with the use of a medicinal product, whether or not considered related to the medicinal product.

An AE includes but is not limited to any clinically significant worsening of a patient's preexisting condition. An abnormal laboratory finding (including ECG finding) that requires an action or intervention by the investigator, or a finding judged by the investigator to represent a change beyond the range of normal physiologic fluctuation, should be reported as an AE.

Adverse events may be treatment emergent (i.e., occurring after initial receipt of investigational product) or nontreatment emergent. A nontreatment-emergent AE is any new sign or symptom, disease, or other untoward medical event that begins after written informed consent has been obtained but before the patient has received investigational product.

Elective treatment or surgery or preplanned treatment or surgery (that was scheduled prior to the patient being enrolled into the study) for a documented pre-existing condition, that did not worsen from baseline, is not considered an AE (serious or nonserious). An untoward medical event occurring during the prescheduled elective procedure or routinely scheduled treatment should be recorded as an AE or SAE.

The term AE is used to include both serious and non-serious AEs.

10.1.2 Definition of serious adverse events

A serious adverse event is an AE occurring during any study phase (i.e., screening, run-in, treatment, wash-out, follow-up), at any dose of the study drugs that fulfils one or more of the following criteria:

- Results in death
- Is immediately life-threatening
- Requires in-patient hospitalization or prolongation of existing hospitalization
- Results in persistent or significant disability or incapacity
- Is a congenital abnormality or birth defect in offspring of the patient
- Is an important medical event that may jeopardize the patient or may require medical intervention to prevent one of the outcomes listed above.
    - Medical or scientific judgment should be exercised in deciding whether expedited reporting is appropriate in this situation. Examples of medically important events are intensive treatment in an emergency room or at home for allergic bronchospasm, blood dyscrasias, or convulsions that do not result in hospitalizations; or development of drug dependency or drug abuse.

The causality of SAEs (their relationship to all study treatment/procedures) will be assessed by the investigator(s) and communicated to AstraZeneca.

10.1.3 Definition of adverse events of special interest (AESI)

An adverse event of special interest (AESI) is one of scientific and medical interest specific to understanding of the Investigational Product and may require close monitoring. An AESI may be serious or non-serious.

AESIs for durvalumab include but are not limited to events with a potential inflammatory or immune-mediated mechanism and which may require more frequent monitoring and/or interventions such as steroids, immunosuppressants and/or hormone replacement therapy. These AESIs may require close monitoring in clinical studies with durvalumab monotherapy and durvalumab combination therapy. An immune-mediated adverse event (imAE) is defined as an AESI that is associated with drug exposure and is consistent with an immune-mediated mechanism of action and where there is no clear alternate etiology. Serologic, immunologic, and histologic (biopsy) data, as appropriate, should be used to support an imAE diagnosis. Appropriate efforts should be made to rule out neoplastic, infectious, metabolic, toxin, or other etiologic causes of the imAE.

If the Investigator has any questions in regard to an event being an imAE, the Investigator should promptly contact the Principal Investigator.

AESIs observed with durvalumab include:

- Diarrhea / Colitis and intestinal perforation
- Pneumonitis / ILD
- hepatitis / transaminase increases

- Endocrinopathies (i.e. events of hypophysitis/hypopituitarism, adrenal insufficiency, hyper- and hypothyroidism and type I diabetes mellitus)
- Rash / Dermatitis
- Nephritis / Blood creatinine increases
- Pancreatitis / serum lipase and amylase increases
- Myocarditis
- Myositis / Polymyositis
- Neuropathy / neuromuscular toxicity (e.g. Guillain-Barr√©, and myasthenia gravis)
- Other inflammatory responses that are rare / less frequent with a potential immune-mediated aetiology include, but are not limited to, pericarditis, sarcoidosis, uveitis and other events involving the eye, skin, haematological and rheumatological events, vasculitis, non-infectious meningitis and non-infectious encephalitis.

In addition, infusion-related reactions and hypersensitivity/anaphylactic reactions with a different underlying pharmacological aetiology are also considered AESIs.

Further information on these risks (e.g. presenting symptoms) can be found in the current version of the durvalumab Investigator's Brochures. More specific guidelines for their evaluation and treatment are described in detail in the Dosing Modification and Toxicity Management Guidelines (please see Appendix 1). These guidelines have been prepared by the sponsor to assist the Investigator in the exercise of his/her clinical judgment in treating these types of toxicities. These guidelines apply to AEs considered causally related to the study drug/study regimen by the reporting investigator.

If new or worsening pulmonary symptoms (e.g. dyspnea) or radiological abnormality suggestive of pneumonitis/interstitial lung disease is observed, toxicity management as described in detail in the Dosing Modification and Toxicity Management Guidelines (see Appendix 1) will be applied. The results of the full diagnostic workup (including high-resolution computed tomography (HRCT), blood and sputum culture, hematological parameters etc) will be captured in the eCRF. It is strongly recommended to perform a full diagnostic workup, to exclude alternative causes such as lymphangitic carcinomatosis, infection, allergy, cardiogenic edema, or pulmonary hemorrhage. In the presence of confirmatory HRCT scans where other causes of respiratory symptoms have been excluded, a diagnosis of pneumonitis (ILD) should be considered and the Dosing Modification and Toxicity Management Guidelines should be followed.

Pneumonitis (ILD) investigation

The following assessments, and additional assessments if required, will be performed to enhance the investigation and diagnosis of potential cases of pneumonitis. The results of the assessment will be collected.

- Physical examination
  - Signs and symptoms (cough, shortness of breath and pyrexia, etc.) including auscultation for lung field will be assessed.
- SpO2
  - Saturation of peripheral oxygen (SpO2)
- Other items

- When pneumonitis (ILD) is suspected during study treatment, the following markers should be measured where possible:
  (i) ILD Markers (KL-6, SP-D) and β-D-glucan
  (ii) Tumor markers: Particular tumor markers which are related to disease progression.

Additional Clinical chemistry: CRP, LDH

Only serious (as determined by the PI) Grade 3 or Grade 4 AESIs will be reported in an expedited manner in accordance with section 10.3.4 to AstraZeneca. All non-serious AESIs will be documented and entered in the eCRF.

10.2 Assessment of safety parameters

10.2.1 Assessment of severity

Assessment of severity is one of the responsibilities of the investigator in the evaluation of AEs and SAEs. Severity will be graded according to the NCI CTCAE v4.03. The determination of severity for all other events not listed in the CTCAE should be made by the investigator based upon medical judgment and the severity categories of Grade 1 to 5 as defined below.

Grade 1 (mild)	An event that is usually transient and may require only minimal treatment or therapeutic intervention. The event does not generally interfere with usual activities of daily living.
Grade 2 (moderate)	An event that is usually alleviated with additional specific therapeutic intervention. The event interferes with usual activities of daily living, causing discomfort but poses no significant or permanent risk of harm to the patient.
Grade 3 (severe)	An event that requires intensive therapeutic intervention. The event interrupts usual activities of daily living, or significantly affects the clinical status of the patient.
Grade 4 (life-threatening)	An event, and/or its immediate sequelae, that is associated with an imminent risk of death or with physical or mental disabilities that affect or limit the ability of the patient to perform activities of daily living (eating, ambulation, toileting, etc).
Grade 5 (fatal)	Death (loss of life) as a result of an event.

It is important to distinguish between serious criteria and severity of an AE. Severity is a measure of intensity whereas seriousness is defined by the criteria in Section 10.1.2. A Grade 3 AE need not necessarily be considered an SAE. For example, a Grade 3 headache that persists for several hours may not meet the regulatory definition of an SAE and would be considered a nonserious event, whereas a Grade 2 seizure resulting in a hospital admission would be considered an SAE.

10.2.2 Assessment of relationship

The relationship to study drug and/or radiation therapy should be assessed by the PI or designee using the following definitions:

- **Not Related:** Evidence exists that the adverse event has an etiology other than the study drug (e.g., pre-existing condition, underlying disease, intercurrent illness, or concomitant medication). This includes events that are considered remotely or unlikely related to study drug.
- **Related:** A temporal relationship exists between the event onset and administration of the study drug. It cannot be readily explained by the patient's clinical state, intercurrent illness, or concomitant therapies. In case of cessation or reduction of the dose, the event abates or resolves and reappears upon re-challenge. It should be emphasized that ineffective study drug treatment should not be considered as causally related in the context of adverse event reporting. This includes events that are considered possibly, probably, or definitely related to study drug.

10.3 Recording of adverse events and serious adverse events

Adverse events will be recorded in the eCRF using a recognized medical term or diagnosis that accurately reflects the event. Adverse events will be assessed by the investigator for severity, relationship to the investigational product, possible etiologies, and whether the event meets criteria of an SAE and therefore requires immediate notification to AstraZeneca/MedImmune Patient Safety.

The following variables will be collected for each AE:

- AE (verbatim)
- The date when the AE started and stopped
- The maximum CTCAE grade reported
- Changes in CTCAE grade
- Whether the AE is serious or not
- Investigator causality rating against the IPs (yes or no)
- Action taken with regard to IPs
- Administration of treatment for the AE
- Outcome

In addition, the following variables will be collected for SAEs:

- Protocol number/ESR tracking number
- Name of reporting investigator/study site

- Subject study identification number
- Subject age at time of onset of AE
- Subject gender
- Study drug dose, start & stop date
- Date the AE met criteria for SAE
- Date the Investigator became aware of the SAE
- Seriousness criteria fulfilled
- Date of hospitalization
- Date of discharge
- Probable cause of death
- Date of death
- Whether an autopsy was performed
- Causality assessment in relation to study procedure(s)
- Causality assessment in relation to other medication, as explained in Section 10.3.2
- Description of the SAE

The grading scales found in the revised NCI CTCAE version 4.03 will be utilized for all events with an assigned CTCAE grading. For those events without assigned CTCAE grades, the recommendation in the CTCAE criteria that converts mild, moderate, and severe events into CTCAE grades should be used. A copy of the CTCAE version 4.03 can be downloaded from the Cancer Therapy Evaluation Program website (http://ctep.cancer.gov).

Events which are unequivocally due to disease progression should not be reported as an AE during the study.

10.3.1 Study recording period and follow-up for adverse events and serious adverse events

Adverse events and serious adverse events will be recorded from time of signature of informed consent, throughout the treatment period and including the follow-up period 90 days after the last dose of durvalumab.

During the course of the study all AEs and SAEs should be proactively followed up for each patient. Every effort should be made to obtain a resolution for all events, even if the events continue after discontinuation/study completion.

If a patient discontinues from treatment for reasons other than disease progression, and therefore continues to have tumor assessments, drug or procedure-related SAEs must be

captured until the patient is considered to have confirmed PD and will have no further tumor assessments.

The investigator is responsible for following all SAEs until resolution, until the patient returns to baseline status, or until the condition has stabilized with the expectation that it will remain chronic, even if this extends beyond study participation.

Follow-up of unresolved adverse events

Any AEs that are unresolved at the subject's last visit in the study are followed up by the investigator for as long as medically indicated, but without further recording. After 90 days, only patients with ongoing investigational product-related SAEs will continue to be followed for safety.

AstraZeneca/Medimmune retains the right to request additional information for any subject with ongoing AE(s)/SAE(s) at the end of the study, if judged necessary.

Post study events

After the subject has been permanently withdrawn from the study, there is no obligation for the investigator to actively report information on new AE or SAEs occurring in former study subjects after the 90-day safety follow-up period for patients treated with durvalumab.
However, if an investigator learns of any SAEs, including death, at any time after the subject has been permanently withdrawn from study, and he/she considers there is a reasonable possibility that the event is related to study treatment, the investigator should notify the study sponsor and AstraZeneca/Medimmune Drug Safety.

10.3.2 Causality collection

The Investigator will assess causal relationship between the IPs and each AE and answer "yes" or "no" to the question "Do you consider that there is a reasonable possibility that the event may have been caused by the investigational product?"

For SAEs causal relationship will also be assessed for other medication and study procedures. Note that for SAEs that could be associated with any study procedure, the causal relationship is implied as "yes."

10.3.3 Relationship to protocol procedures

The Investigator is also required to provide an assessment of the relationship of SAEs to protocol procedures on the SAE report form. This includes both non-treatment–emergent (i.e., SAEs that occur prior to the administration of IP) and treatment-emergent SAEs. A protocol-related SAE may occur as a result of a procedure or intervention required during the study (e.g., blood collection). The following guidelines should be used by Investigators to assess the relationship of SAEs to the protocol:

- Protocol related: The event occurred due to a procedure or intervention that was described in the protocol for which there is no alternative etiology present in the patient's medical record.

- Not protocol related: The event is related to an etiology other than the procedure or intervention that was described in the protocol. The alternative etiology must be documented in the study patient's medical record.

10.3.4 Adverse events based on signs and symptoms

All AEs spontaneously reported by the patient or reported in response to the open question from the study personnel: “Have you had any health problems since the previous visit/you were last asked?” or revealed by observation will be collected and recorded in the eCRF. When collecting AEs, the recording of diagnoses is preferred, when possible, to recording a list of signs and symptoms. However, if a diagnosis is known and there are other signs or symptoms that are not generally part of the diagnosis, the diagnosis and each sign or symptom will be recorded separately.

10.3.5 Adverse events based on examinations and tests

Deterioration as compared to baseline in protocol-mandated laboratory values and vital signs should therefore only be reported as AEs if they fulfill any of the SAE criteria or are the reason for discontinuation of treatment with the IPs.

If deterioration in a laboratory value or vital sign is associated with clinical signs and symptoms, the sign or symptom will be reported as an AE and the associated laboratory result or vital sign will be considered as additional information. Whenever possible, the reporting Investigator should use the clinical rather than the laboratory term (e.g., anemia versus low hemoglobin value). In the absence of clinical signs or symptoms, clinically relevant deteriorations in nonmandated parameters should be reported as AEs.

Deterioration of a laboratory value that is unequivocally due to disease progression should not be reported as an AE/SAE.

Any new or aggravated clinically relevant abnormal medical finding at a physical examination as compared with the baseline assessment will be reported as an AE.

10.3.6 Hy’s Law

Cases where a patient shows elevations in liver biochemistry may require further evaluation and occurrences of AST or ALT ≥3 × ULN together with total bilirubin ≥2 × ULN may need to be reported as SAEs. Please refer to Appendix 1 for further instruction on cases of increases in liver biochemistry and evaluation of Hy’s law.

10.3.7 Disease progression

Disease progression can be considered as a worsening of a patient’s condition attributable to the disease for which the IP is being studied. It may be an increase in the severity of the disease under study and/or increases in the symptoms of the disease. The development of new or progression of existing metastasis to the primary cancer under study should be considered as disease progression and not an AE. Events that are unequivocally due to disease progression should not be reported as an AE during the study.

10.3.8 Deaths

All deaths that occur during the study treatment period, or within the protocol-defined follow-up period after the administration of the last dose of study drug, must be reported as follows:

- Death clearly resulting from disease progression should be reported to the Sponsor at the next monitoring visit and should be documented in the eCRF. It should not be reported as an SAE.
- Where death is not due (or not clearly due) to progression of the disease under study, the AE causing the death must be reported to the Sponsor and AstraZeneca as an SAE within 24 hours. It should also be documented in the eCRF. The report should contain a comment regarding the co involvement of PD, if appropriate, and should assign main and contributory causes of death.
- Deaths with an unknown cause should always be reported to the Sponsor and AstraZeneca as an SAE. It should also be documented in the eCRF. A post mortem may be helpful in the assessment of the cause of death, and if performed, a copy of the post-mortem results should be forwarded to AstraZeneca Patient Safety or its representative within the usual timeframes.

Deaths occurring after the protocol defined safety follow up period after the administration of the last dose of study drug should be documented in the eCRF. If the death occurred as a result of an event that started after the defined safety follow up period and the event is considered to be due to a late onset toxicity to study drug, then it should also be reported as an SAE.

AstraZeneca/MedImmune retains the right to request additional information for any patient with ongoing AE(s)/SAE(s) at the end of the study, if judged necessary.

10.3.9 Reporting of serious adverse events

All SAEs will be reported, whether or not considered causally related to the investigational product, or to the study procedure(s). The reporting period for SAEs is the period immediately following the time that written informed consent is obtained through 90 days after the last dose of durvalumab or until the initiation of alternative anticancer therapy. The investigator and/or sponsor are responsible for informing the Ethics Committee and/or the Regulatory Authority of the SAE as per local requirements.

The investigator must inform the Sponsor, via a MedWatch form, of any serious or unexpected adverse events ***within 24 hours of awareness of the event***, and will concurrently forward all such reports to AstraZeneca.

* A ***cover page*** should accompany the ***MedWatch*** form indicating the following:

"Notification from an Investigator Sponsored Study"

The investigator's name and address

The trial name/title and AstraZeneca ISS reference number (ESR-17-13229)

***** Investigator must also indicate, either in the SAE report or the cover page, the ***causality*** of events ***in relation to all study medications*** and if the SAE is ***related to disease progression***, as determined by the investigator.

**** Send SAE report and accompanying cover page by way of email to AstraZeneca's <u>designated mailbox:</u>*** AEMailboxClinicalTrialTCS@astrazeneca.com

SAE reports may also be faxed to 1-302-886-4114, although, the preferred method of reporting is via the AE mailbox.

SAE reports should also be emailed to the Sponsor designated mailboxes: CPDM.IIT@cuanschutz.edu, DSMC@ucdenver.edu, and Sana.Karam@cuanschutz.edu

If a non-serious AE becomes serious, this initial and other relevant follow-up information must also be provided to AstraZeneca and the Sponsor.

10.3.10 Reporting of deaths to AstraZeneca

All deaths that occur during the study, or within the protocol defined 90-day post last dose of durvalumab safety follow-up period must be reported to the Sponsor and AstraZeneca as follows:

- Death that is clearly the result of disease progression should be documented in the eCRF, but should not be reported as an SAE.
- Where death is not due (or not clearly due) to progression of the disease under study, the AE causing the death must be reported to the Sponsor and AstraZeneca as a SAE within ***24 hours of knowledge of the event*** (see Section 10.3.10 for further details). The report should contain a comment regarding the coinvolvement of progression of disease, if appropriate, and should assign main and contributory causes of death.
- Deaths with an unknown cause should always be reported as a SAE.

Deaths that occur following the protocol-defined 90-day post-last-dose of durvalumab safety follow-up period will be documented as events for survival analysis, but will not be reported as an SAE. However, if an investigator learns of any SAEs, including death, at any time after the patient has been permanently withdrawn from study, and he/she considers there is a reasonable possibility that the event is related to study treatment, the investigator should notify the Sponsor and AstraZeneca/MedImmune Drug Safety.

10.3.11 Overdose

An overdose is defined as a patient receiving a dose of durvalumab in excess of that specified in the Investigator's Brochure, unless otherwise specified in this protocol.

Any overdose of a study patient with durvalumab, with or without associated AEs/SAEs, is required to be reported within ***24 hours of knowledge of the event*** to the Sponsor and AstraZeneca/MedImmune Patient Safety or designee using the designated Safety e-mailbox (see Section 10.3.10 for details). If the overdose results in an AE, the AE must also be recorded as an AE (see Section 10.3). Overdose does not automatically make an AE serious, but if the consequences of the overdose are serious, for example death or hospitalization, the event is serious and must be recorded and reported as an SAE (see Section 10.1.2 and Section 10.3.10). There is currently no specific treatment in the event of an overdose of durvalumab.

The investigator will use clinical judgment to treat any overdose.

10.3.12 Hepatic function abnormality

Hepatic function abnormality that fulfills the biochemical criteria of a potential Hy's Law case in a study patient, with or without associated clinical manifestations, is required to be reported as "hepatic function abnormal" ***within 24 hours of knowledge of the event*** to the Sponsor and AstraZeneca Patient Safety using the designated Safety e-mailbox (see Section 10.3.10 for details) unless a definitive underlying diagnosis for the abnormality (e.g., cholelithiasis or bile duct obstruction) that is unrelated to investigational product has been confirmed. The criteria for a potential Hy's Law case is Aspartate Aminotransferase (AST) or Alanine Aminotransferase (ALT) ≥3x Upper Limit of Normal (ULN) together with Total Bilirubin (TBL) ≥2xULN at any point during the study following the start of study medication irrespective of an increase in Alkaline Phosphatase (ALP).

- If the definitive underlying diagnosis for the abnormality has been established and is unrelated to investigational product, the decision to continue dosing of the study patient will be based on the clinical judgment of the investigator.

- If no definitive underlying diagnosis for the abnormality is established, dosing of the study patient must be interrupted immediately. Follow-up investigations and inquiries must be initiated by the investigational site without delay.

Each reported event of hepatic function abnormality will be followed by the investigator and evaluated by the Sponsor and AstraZeneca/MedImmune.

10.3.13 Pregnancy

Maternal exposure

If a patient becomes pregnant during the course of the study, the IPs should be discontinued immediately.

Pregnancy itself is not regarded as an AE unless there is a suspicion that the IP under study may have interfered with the effectiveness of a contraceptive medication. Congenital abnormalities or birth defects and spontaneous miscarriages should be reported and handled as SAEs. Elective abortions without complications should not be handled as AEs. The outcome of all pregnancies (spontaneous miscarriage, elective termination, ectopic pregnancy, normal birth, or congenital abnormality) should be followed up and documented even if the patient was discontinued from the study.

If any pregnancy occurs in the course of the study, then the Investigator or other site personnel should inform the Sponsor and appropriate AstraZeneca representatives within 1 day, i.e., immediately, but ***no later than 24 hours*** of when he or she becomes aware of it.

The designated AstraZeneca representative will work with the Investigator to ensure that all relevant information is provided to the AstraZeneca Patient Safety data entry site within 1 to 5 calendar days for SAEs and within 30 days for all other pregnancies.

The same timelines apply when outcome information is available.

Paternal exposure

Male patients should refrain from fathering a child or donating sperm during the study and for 180 days after the last dose of durvalumab + any drug combination therapy or 90 days after the last dose of durvalumab monotherapy, whichever is the longer time period.

Pregnancy of the patient's partner is not considered to be an AE. However, the outcome of all pregnancies (spontaneous miscarriage, elective termination, ectopic pregnancy, normal birth, or congenital abnormality) occurring from the date of the first dose until 180 days after the last dose of durvalumab + any drug combination therapy or 90 days after the last dose of durvalumab monotherapy, whichever is the longer time period should, if possible, be followed up and documented.

Where a report of pregnancy is received, prior to obtaining information about the pregnancy, the Investigator must obtain the consent of the patient's partner. Therefore, the local study team should adopt the generic ICF template in line with local procedures and submit it to the relevant Ethics Committees (ECs)/Institutional Review Boards (IRBs) prior to use.

10.4 Medication error

For the purposes of this clinical study a medication error is an unintended failure or mistake in the treatment process for an AstraZeneca study drug that either causes harm to the patient or has the potential to cause harm to the patient.

A medication error is not lack of efficacy of the drug, but rather a human or process related failure while the drug is in control of the study site staff or patient.

Medication error includes situations where an error:

- Occurred
- Was identified and intercepted before the patient received the drug
- Did not occur, but circumstances were recognized that could have led to an error

Examples of events to be reported in clinical studies as medication errors:

- Drug name confusion
- Dispensing error e.g. medication prepared incorrectly, even if it was not actually given to the patient
- Drug not administered as indicated, for example, wrong route or wrong site of administration
- Drug not taken as indicated e.g. tablet dissolved in water when it should be taken as a solid tablet
- Drug not stored as instructed e.g. kept in the fridge when it should be at room temperature
- Wrong patient received the medication
- Wrong drug administered to patient (excluding IVRS/IWRS errors)

Examples of events that **do not** require reporting as medication errors in clinical studies:

- Errors related to or resulting from IVRS/IWRS - including those that lead to one of the above listed events that would otherwise have been a medication error
- Patient accidentally missed drug dose(s) e.g. forgot to take medication
- Accidental overdose (will be captured as an overdose)
- Patient failed to return unused medication or empty packaging
- Errors related to background and rescue medication, or standard of care medication in open label studies, even if an AZ product

Medication errors are not regarded as AEs but AEs may occur as a consequence of the medication error.

If a medication error occurs in the course of the study, then the Investigator or other site personnel informs the Sponsor and appropriate AstraZeneca representatives within 1 day i.e., immediately but ***no later than 24 hours*** of when he or she becomes aware of it.

The designated AstraZeneca representative works with the Investigator to ensure that all relevant information is completed within 1 or 5 calendar days if there is an SAE associated with the medication error (see Section 10.3.9) and within 30 days for all other medication errors.

11. STATISTICAL METHODS AND SAMPLE SIZE DETERMINATION

11.1 Description of analysis sets

See section 3.3 for details regarding the FAS, EAS and SAS. Efficacy analysis is discussed in section 9.1.1.

11.2 Methods of statistical analyses

See section 3 for discussion of objectives, study design and sample size justification.

11.3 Patient disposition and characteristics

A detailed description of patient disposition will be provided. It will include a summary of the number and percentage of patients entered into the study, enrolled in the study, and treated, as well as number and percentage of patients completing the study, or discontinuing (overall and by reason for discontinuation).

Patient demographic and baseline characteristics will be summarized using descriptive statistics. For continuous variables, descriptive statistics (n, mean, standard deviation, standard error, median, minimum, and maximum) will be provided. For categorical variables, patient counts and percentages will be provided. Categories for missing data will be presented if necessary. The categorical variables such as gender and race will be summarized using descriptive statistics for each variable category. These p-values are for descriptive purposes only, to aid in the assessment of baseline comparability. Therefore, they are not adjusted for multiplicity.

12. ETHICAL AND REGULATORY REQUIREMENTS

12.1 Ethical conduct of the study

The study will be performed in accordance with ethical principles that have their origin in the Declaration of Helsinki and are consistent with ICH/Good Clinical Practice, and applicable regulatory requirements Patient data protection.

12.2 Ethics and regulatory review

Before initiating this trial, the Investigator will have written and dated approval from the Institutional Review Board for the protocol, written informed consent form, subject recruitment materials, and any other written information to be provided to subjects before any protocol related procedures are performed on any subjects.

The clinical investigation will not begin until either FDA has determined that the study under the Investigational Drug Application (IND) is allowed to proceed or the Investigator has received a letter from FDA stating that the study is exempt from IND requirements.

The Investigator must comply with the applicable regulations in Title 21 of the Code of Federal Regulations (21 CFR §50, §54, and §312), GCP/ICH guidelines, and all applicable regulatory requirements. The IRB must comply with the regulations in 21 CFR §56 and applicable regulatory requirements.

The protocol, the proposed informed consent form, and all forms of participant information related to the study will be reviewed and approved by the University of Colorado Institutional Review Board (IRB), University of Colorado Protocol Review and Monitoring System (PRMS) and Data Safety Monitoring Board (DSMB). The initial protocol and all protocol amendments must be approved by the IRB prior to implementation.

12.3 Informed consent

All participants must be provided a consent form describing the study with sufficient information for each participant to make an informed decision regarding their participation. Participants must sign the IRB-approved informed consent form prior to participation in any study specific procedure. The participant must receive a copy of the signed and dated consent document. The original signed copy of the consent document must be retained in the medical record or research file.

12.4 Changes to the protocol and informed consent form

Once the protocol has been approved by the University of Colorado IRB (COMIRB) and other oversight committees as noted in section 12.2, any changes to the protocol must be documented in the form of an amendment. The amendment must be signed by the Investigator and approved by IRB prior to implementation.

If it becomes necessary to alter the protocol to eliminate an immediate hazard to patients, an amendment may be implemented prior to IRB approval. In this circumstance, however, the investigator must then notify COMIRB in writing within five (5) working days after implementation.

12.5 Audits and inspections

All records and documents pertaining to the study will be maintained in appropriate permanent files as per the ICH guidelines for Essential Documents for the Conduct of a Clinical Trial and 21 CFR 11, and will be available for inspection by the PI, PI designee, AstraZeneca, the FDA, or any other designated review body at any time.

13. STUDY MANAGEMENT

13.1 Training of study site personnel

Investigational staff including physicians, clinicians, coordinators, etc. involved in the study will need to review the protocol and any amendments and provide written documentation of study training.

13.2 Handling and Documentation of Clinical Supplies

The University of Colorado Principal Investigator will maintain complete records showing the receipt, dispensation, return, or other disposition of all investigational drugs. The date, quantity and batch or code number of the drug, and the identification of patients to whom study drug has been dispensed by patient number and initials will be included. The sponsor-investigator will maintain written records of any disposition of the study drug.

The Principal Investigator shall not make the investigational drug available to any individuals other than to qualified study patients. Furthermore, the Principal Investigator will not allow the investigational drug to be used in any manner other than that specified in this protocol.

13.3 Monitoring of the study

Monitoring and Oversight

The sponsor investigator will be responsible for monitoring the trial per the trial monitoring plan, in addition to overseeing the safety and efficacy of the trial including any specimens collected, executing the data and safety monitoring (DSM) plan, and complying with all reporting requirements to local and federal authorities. This oversight will be accomplished through additional oversight from the Data and Safety Monitoring Committee (DSMC) at the University of Colorado Cancer Center (CU Cancer Center). The DSMC is responsible for ensuring data quality and study participant safety for all clinical studies at the CU Cancer Center, which is the coordinating institution of this trial. A summary of the DSMC's activities is as follows:

• Conduct of internal audits

• Ongoing review of all serious adverse events (SAEs) and unanticipated problems (UAPs)

• May submit recommendations for corrective actions to the CU Cancer Center's Executive Committee

Per the CU Cancer Center Institutional DSM Plan, SAEs and UAPs are reported to the DSMC, IRB and the sponsor investigator per protocol. All SAEs and UAPs are to be reported to the DSMC within 7 (for fatal or life-threatening events) or 15 (non-life-threatening events) calendar days of the sponsor investigator receiving notification of the occurrence.

Each subject's treatment outcomes will be discussed by the site PI and appropriate staff at regularly scheduled meetings. Data regarding number of subjects, significant toxicities, dose modifications, and treatment responses will be discussed and documented in the meeting's minutes.

The sponsor investigator is responsible for organizing and conducting regularly scheduled teleconferences with all participating sites. The sponsor investigator will also be responsible for including data from all the participating sites to include the minutes from these regularly scheduled teleconferences between the sponsor investigator and the sites within the overall trial's DSM progress report.

The sponsor investigator will provide a DSM progress report to the CU Cancer Center DSMC on a recurring basis (either every six or twelve months based on DSMC vote). The DSM report will include a protocol summary, current enrollment numbers, summary of toxicity data to include specific SAEs, UAPs and AEs, any dose modifications, all protocol deviations, and protocol amendments. The DSM report submitted to the DSMC will also include, if applicable, the results of any efficacy data analysis conducted. Results and recommendations from the review of this progress report by the DSMC will then be provided to the sponsor investigator in a DSMC review letter. The sponsor investigator is then responsible for ensuring this letter is submitted to the site's IRB of record at the time of IRB continuing review.

Quality Control and Quality Assurance
Site monitoring visits will be performed by the sponsor investigator's authorized representative on a regular basis, pursuant to the Monitoring Plan. During these visits, information recorded on the CRFs will be verified against source documents. Additional computer programs that identify selected protocol deviations, out-of-range data, and other data errors within the electronic data entry may also be used to help monitor the study. As necessary, requests for data clarification or correction will be sent to the appropriate site PI.
Independent auditors from the sponsor investigator's authorized representative will be allowed by the site's PI to audit. In addition, audits may be conducted at any time by appropriate regulatory authorities and/or the IRB.

The Principal Investigator and/or his/her designee will prepare and maintain adequate and accurate participant case histories with observations and data pertinent to the study. Study specific Case Report Forms (CRFs) will document safety and treatment outcomes for safety monitoring and data analysis. All study data will be entered into the medical record via standardized CRFs in accordance with the study calendar, using single data entry with a secure access account. The Clinical Research Coordinator (CRC) will complete the CRFs as soon as possible upon completion of the study visit; the Investigator will review and approve the completed CRFs.

The information collected on CRFs shall be identical to that appearing in original source documents. Source documents will be found in the patient's medical records maintained by University of Colorado personnel. All source documentation should be kept in separate research folders for each patient.

In accordance with federal regulations, the Investigator is responsible for the accuracy and authenticity of all clinical and laboratory data entered onto CRFs. The PI will approve all completed CRFs to attest that the information contained on the CRFs is true and accurate.

All source documentation and data will be available for review/monitoring by University of Colorado regulatory and oversight committees and regulatory agencies.

The Principal Investigator will be responsible for ensuring the accurate capture of study data. At study completion, when the CRFs have been declared to be complete and accurate, the database will be locked. Any changes to the data entered into the CRFs after that time can only be made by joint written agreement among the Study Chair, the Trial Statistician, and the Protocol Project Manager.

14. DATA MANAGEMENT

14.1 Study governance and oversight

The safety of all AstraZeneca clinical studies is closely monitored on an ongoing basis by AstraZeneca representatives in consultation with Patient Safety. Issues identified will be addressed; for instance, this could involve amendments to the study protocol and letters to Investigators.

14.2 Record Keeping and Record Retention

The Principal Investigator is required to maintain adequate records of the disposition of the drug, including dates, quantity, and use by subjects, as well as written records of the disposition of the drug when the study ends.

The Principal Investigator is required to prepare and maintain adequate and accurate case histories that record all observations and other data pertinent to the investigation on each individual administered the investigational drug or employed as a control in the investigation. Case histories include the case report forms and supporting data including, for example, signed and dated consent forms and medical records including, for example, progress notes of the physician, the individual's hospital chart(s), and the nurses' notes. The case history for each individual shall document that informed consent was obtained prior to participation in the study.

Study documentation includes all CRFs, data correction forms or queries, source documents, Sponsor-Investigator correspondence, monitoring logs/letters, and regulatory documents (e.g., protocol and amendments, CHR correspondence and approval, signed patient consent forms).

Source documents include all recordings of observations or notations of clinical activities and all reports and records necessary for the evaluation and reconstruction of the clinical research study.

In accordance with FDA regulations, the investigator shall retain records for a period of 2 years following the date a marketing application is approved for the drug for the indication for which it is being investigated; or, if no application is to be filed or if the application is not approved for such indication, until 2 years after the investigation is discontinued and FDA is notified.

14.3 Regulatory Documentation

Prior to implementing this protocol at University of Colorado, the protocol, informed consent form, HIPAA authorization and any other information pertaining to participants must be approved by the University of Colorado oversight and regulatory committees as applicable.

15. PROTECTION OF HUMAN SUBJECTS

15.1 Protection from Unnecessary Harm

Each clinical site is responsible for protecting all subjects involved in human experimentation. This is accomplished through the review mechanisms of the Colorado Institutional Review Board and Data Safety Monitoring Board (DSMB) and through the process of informed consent. The Colorado regulatory and oversight committees will review all proposed studies involving human experimentation and ensures that the subject’s rights and welfare are protected and that the potential benefits and/or the importance of the knowledge to be gained outweigh the risks to the individual. The University of Colorado regulatory and oversight committees also reviews the informed consent document associated with each study in order to ensure that the consent document accurately and clearly communicates the nature of the research to be done and its associated risks and benefits.

15.2 Protection of Privacy

Patients will be informed of the extent to which their confidential health information generated from this study may be used for research purposes. Following this discussion, they will be asked to sign the HIPAA form and informed consent documents. The original signed document will become part of the patient’s medical records, and each patient will receive a copy of the signed document. The use and disclosure of protected health information will be limited to the individuals described in the informed consent document.

16. LIST OF REFERENCES

Alexandrov, L. B., Nik-Zainal, S., Wedge, D. C., Aparicio, S. A. J. R., Behjati, S., Biankin, A. V., . . . Stratton, M. R. (2013). Signatures of mutational processes in human cancer. *Nature, 500*(7463), 415-421. doi:10.1038/nature12477

Ang, K. K., Zhang, Q., Rosenthal, D. I., Nguyen-Tan, P. F., Sherman, E. J., Weber, R. S., . . . Axelrod, R. S. (2014). Randomized phase III trial of concurrent accelerated radiation plus cisplatin with or without cetuximab for stage III to IV head and neck carcinoma: RTOG 0522. *J Clin Oncol, 32*(27), 2940-2950. doi:10.1200/JCO.2013.53.5633

Antonia, S. J., Villegas, A., Daniel, D., Vicente, D., Murakami, S., Hui, R., . . . Investigators, P. (2017). Durvalumab after Chemoradiotherapy in Stage III Non-Small-Cell Lung Cancer. *N Engl J Med*. doi:10.1056/NEJMoa1709937

Ayers, M., Lunceford, J., Nebozhyn, M., Murphy, E., Loboda, A., Kaufman, D. R., . . . McClanahan, T. K. (2017). IFN-γ–related mRNA profile predicts clinical response to PD-1 blockade. *The Journal of Clinical Investigation, 127*(8), 2930-2940. doi:10.1172/JCI91190

Badoual, C., Hans, S., Merillon, N., Van Ryswick, C., Ravel, P., Benhamouda, N., . . . Tartour, E. (2013). PD-1–Expressing Tumor-Infiltrating T Cells Are a Favorable Prognostic Biomarker in HPV-Associated Head and Neck Cancer. *Cancer Research, 73*(1), 128-138. doi:10.1158/0008-5472.can-12-2606

Badoual, C., Sandoval, F., Pere, H., Hans, S., Gey, A., Merillon, N., . . . Tartour, E. (2010). Better understanding tumor–host interaction in head and neck cancer to improve the design and development of immunotherapeutic strategies. *Head & Neck, 32*(7), 946-958. doi:10.1002/hed.21346

Brahmer, J. R., Horn, L., Gandhi, L., Spigel, D. R., Antonia, S. J., Rizvi, N. A., . . . Gettinger, S. N. (2014). Nivolumab (anti-PD-1, BMS-936558, ONO-4538) in patients (pts) with advanced non-small-cell lung cancer (NSCLC): Survival and clinical activity by subgroup analysis. *Journal of Clinical Oncology, 32*(15_suppl), 8112-8112. doi:10.1200/jco.2014.32.15_suppl.8112

Brahmer , J. R., Tykodi , S. S., Chow , L. Q. M., Hwu , W.-J., Topalian , S. L., Hwu , P., . . . Wigginton , J. M. (2012). Safety and Activity of Anti–PD-L1 Antibody in Patients with Advanced Cancer. *New England Journal of Medicine, 366*(26), 2455-2465. doi:10.1056/NEJMoa1200694

Butte, M. J., Keir, M. E., Phamduy, T. B., Freeman, G. J., & Sharpe, A. H. (2007). PD-L1 interacts specifically with B7-1 to inhibit T cell proliferation. *Immunity, 27*(1), 111-122. doi:10.1016/j.immuni.2007.05.016

Campian, J. L., Sarai, G., Ye, X., Marur, S., & Grossman, S. A. (2014). Association between severe treatment-related lymphopenia and progression-free survival in patients with newly diagnosed squamous cell head and neck cancer. *Head Neck, 36*(12), 1747-1753. doi:10.1002/hed.23535

Cohen, E. E. (2017). ESMO 2017 Press Release: KEYNOTE-040 Evaluates Pembrolizumab in Head and Neck Cancer [Press release]. Retrieved from http://www.esmo.org/Press-Office/Press-Releases/KEYNOTE-040-Evaluates-Pembrolizumab-in-Head-and-Neck-Cancer

Concha-Benavente, F., Srivastava, R. M., Trivedi, S., Lei, Y., Chandran, U., Seethala, R. R., . . . Ferris, R. L. (2016). Identification of the Cell-Intrinsic and -Extrinsic Pathways Downstream of EGFR and IFNgamma That Induce PD-L1 Expression in Head and Neck Cancer. *Cancer Res, 76*(5), 1031-1043. doi:10.1158/0008-5472.CAN-15-2001

Demaria, S., Coleman, C. N., & Formenti, S. C. (2016). Radiotherapy: Changing the Game in Immunotherapy. *Trends Cancer, 2*(6), 286-294. doi:10.1016/j.trecan.2016.05.002

Deng, L., Liang, H., Burnette, B., Beckett, M., Darga, T., Weichselbaum, R. R., & Fu, Y.-X. (2014). Irradiation and anti–PD-L1 treatment synergistically promote antitumor immunity in mice. *The Journal of Clinical Investigation, 124*(2), 687-695. doi:10.1172/JCI67313

Dovedi, S. J., & Illidge, T. M. (2015). The antitumor immune response generated by fractionated radiation therapy may be limited by tumor cell adaptive resistance and can be circumvented by PD-L1 blockade. *Oncoimmunology, 4*(7), e1016709. doi:10.1080/2162402X.2015.1016709

Drake, C. G., McDermott, D. F., Sznol, M., Choueiri, T. K., Kluger, H. M., Powderly, J. D., . . . Atkins, M. B. (2013). Survival, safety, and response duration results of nivolumab (Anti-PD-1; BMS-936558; ONO-4538) in a phase I trial in patients with previously treated metastatic renal cell carcinoma (mRCC): Long-term patient follow-up. *Journal of Clinical Oncology, 31*(15_suppl), 4514-4514. doi:10.1200/jco.2013.31.15_suppl.4514

Dunn, G. P., Old, L. J., & Schreiber, R. D. (2004). The Three Es of Cancer Immunoediting. *Annual Review of Immunology, 22*(1), 329-360. doi:10.1146/annurev.immunol.22.012703.104803

Ellis, S., Carroll, K. J., & Pemberton, K. (2008). Analysis of duration of response in oncology trials. *Contemporary Clinical Trials, 29*(4), 456-465. doi:https://doi.org/10.1016/j.cct.2007.10.008

Fairman, D., Narwal, R., Liang, M., Robbins, P. B., Schneider, A., Chavez, C., . . . Roskos, L. (2014). Pharmacokinetics of MEDI4736, a fully human anti-PDL1 monoclonal antibody, in patients with advanced solid tumors. *Journal of Clinical Oncology, 32*(15_suppl), 2602-2602. doi:10.1200/jco.2014.32.15_suppl.2602

Fife, B. T., & Bluestone, J. A. (2008). Control of peripheral T-cell tolerance and autoimmunity via the CTLA-4 and PD-1 pathways. *Immunological Reviews, 224*(1), 166-182. doi:10.1111/j.1600-065X.2008.00662.x

Glanzmann, C., & Grätz, K. W. Radionecrosis of the mandibula: a retrospective analysis of the incidence and risk factors. *Radiotherapy and Oncology, 36*(2), 94-100. doi:10.1016/0167-8140(95)01583-3

Harada, H., Omura, K., Tomioka, H., Nakayama, H., Hiraki, A., Shinohara, M., . . . Shintani, S. (2013). Multicenter phase II trial of preoperative chemoradiotherapy with S-1 for locally advanced oral squamous cell carcinoma. *Cancer Chemotherapy and Pharmacology, 71*(4), 1059-1064. doi:10.1007/s00280-013-2101-5

Herbst, R. S., Gordon, M. S., Fine, G. D., Sosman, J. A., Soria, J.-C., Hamid, O., . . . Hodi, F. S. (2013). A study of MPDL3280A, an engineered PD-L1 antibody in patients with locally advanced or metastatic tumors. *Journal of Clinical Oncology, 31*(15_suppl), 3000-3000. doi:10.1200/jco.2013.31.15_suppl.3000

Hirano, F., Kaneko, K., Tamura, H., Dong, H., Wang, S., Ichikawa, M., . . . Chen, L. (2005). Blockade of B7-H1 and PD-1 by Monoclonal Antibodies Potentiates Cancer Therapeutic Immunity. *Cancer Research, 65*(3), 1089-1096.

Hodi, F. S., Sznol, M., Kluger, H. M., McDermott, D. F., Carvajal, R. D., Lawrence, D. P., . . . Sosman, J. A. (2014). Long-term survival of ipilimumab-naive patients (pts) with advanced melanoma (MEL) treated with nivolumab (anti-PD-1, BMS-936558, ONO-4538) in a phase I trial. *Journal of Clinical Oncology, 32*(15_suppl), 9002-9002. doi:10.1200/jco.2014.32.15_suppl.9002

Iwai, Y., Ishida, M., Tanaka, Y., Okazaki, T., Honjo, T., & Minato, N. (2002). Involvement of PD-L1 on tumor cells in the escape from host immune system and tumor immunotherapy by PD-L1 blockade. *Proceedings of the National Academy of Sciences of the United States of America, 99*(19), 12293-12297. doi:10.1073/pnas.192461099

Jellema, A. P., Doornaert, P., Slotman, B. J., Rene Leemans, C., & Langendijk, J. A. (2005). Does radiation dose to the salivary glands and oral cavity predict patient-rated xerostomia and sticky saliva in head and neck cancer patients treated with curative radiotherapy? *Radiotherapy and Oncology, 77*(2), 164-171. doi:https://doi.org/10.1016/j.radonc.2005.10.002

Jen, Y.-M., Lin, Y.-C., Wang, Y.-B., & Wu, D.-M. (2006). Dramatic and prolonged decrease of whole salivary secretion in nasopharyngeal carcinoma patients treated with radiotherapy. *Oral Surgery, Oral Medicine, Oral Pathology, Oral Radiology, and Endodontology, 101*(3), 322-327. doi:https://doi.org/10.1016/j.tripleo.2005.01.011

Johnson, C. B., & Jagsi, R. (2016). The Promise of the Abscopal Effect and the Future of Trials Combining Immunotherapy and Radiation Therapy. *International Journal of Radiation Oncology*Biology*Physics, 95*(4), 1254-1256. doi:https://doi.org/10.1016/j.ijrobp.2016.02.067

Kang, J., Demaria, S., & Formenti, S. (2016). Current clinical trials testing the combination of immunotherapy with radiotherapy. *J Immunother Cancer, 4*, 51. doi:10.1186/s40425-016-0156-7

Keir, M. E., Butte, M. J., Freeman, G. J., & Sharpe, A. H. (2008). PD-1 and Its Ligands in Tolerance and Immunity. *Annual Review of Immunology, 26*(1), 677-704. doi:10.1146/annurev.immunol.26.021607.090331

Lan, K. K. G., & DeMets, D. L. (1983). Discrete Sequential Boundaries for Clinical Trials. *Biometrika, 70*(3), 659-663. doi:10.2307/2336502

Larkin, J., Chiarion-Sileni, V., Gonzalez, R., Grob, J. J., Cowey, C. L., Lao, C. D., . . . Wolchok, J. D. (2015). Combined Nivolumab and Ipilimumab or Monotherapy in Untreated Melanoma. *New England Journal of Medicine, 373*(1), 23-34. doi:10.1056/NEJMoa1504030

Leeman, J. E., Li, J. G., Pei, X., Venigalla, P., Zumsteg, Z. S., Katsoulakis, E., . . . Riaz, N. (2017). Patterns of Treatment Failure and Postrecurrence Outcomes Among Patients With Locally Advanced Head and Neck Squamous Cell Carcinoma After Chemoradiotherapy Using Modern Radiation Techniques. *JAMA Oncol*. doi:10.1001/jamaoncol.2017.0973

Lin, S., Dutra, J., Keni, J., Dumanian, G. A., Fine, N., & Pelzer, H. (2005). Preoperative radiation therapy and its effects on outcomes in microsurgical head and neck reconstruction. *Otolaryngology-Head and Neck Surgery, 132*(6), 845-848. doi:10.1016/j.otohns.2005.03.006

Lyford-Pike, S., Peng, S., Young, G. D., Taube, J. M., Westra, W. H., Akpeng, B., . . . Pai, S. I. (2013). Evidence for a role of the PD-1:PD-L1 pathway in immune resistance of HPV-associated head and neck squamous cell carcinoma. *Cancer Research, 73*(6), 1733-1741. doi:10.1158/0008-5472.CAN-12-2384

Manda, K., Glasow, A., Paape, D., & Hildebrandt, G. (2012). Effects of ionizing radiation on the immune system with special emphasis on the interaction of dendritic and T cells. *Front Oncol, 2*, 102. doi:10.3389/fonc.2012.00102

Mandal, R., Şenbabaoğlu, Y., Desrichard, A., Havel, J. J., Dalin, M. G., Riaz, N., . . . Morris, L. G. T. (2016). The head and neck cancer immune landscape and its immunotherapeutic implications. *JCI Insight, 1*(17). doi:10.1172/jci.insight.89829

Mendenhall, W. M. (2004). Mandibular Osteoradionecrosis. *Journal of Clinical Oncology, 22*(24), 4867-4868. doi:10.1200/jco.2004.09.959

Monjazeb, A. M., & Schoenfeld, J. D. (2016). Radiation dose and checkpoint blockade immunotherapy: unanswered questions. *Lancet Oncol, 17*(1), e3-4. doi:10.1016/S1470-2045(15)00541-0

Mücke, T., Konen, M., Wagenpfeil, S., Kesting, M. R., Wolff, K.-D., & Hölzle, F. (2011). Low-Dose Preoperative Chemoradiation Therapy Compared with Surgery Alone with

or Without Postoperative Radiotherapy in Patients with Head and Neck Carcinoma. *Annals of Surgical Oncology, 18*(10), 2739-2747. doi:10.1245/s10434-011-1643-1

Narwal, R., Roskos, L. K., & Robbie, G. J. (2013). Population Pharmacokinetics of Sifalimumab, an Investigational Anti-Interferon-α Monoclonal Antibody, in Systemic Lupus Erythematosus. *Clinical Pharmacokinetics, 52*(11), 1017-1027. doi:10.1007/s40262-013-0085-2

Ng, C. M., Lum, B. L., Gimenez, V., Kelsey, S., & Allison, D. (2006). Rationale for Fixed Dosing of Pertuzumab in Cancer Patients Based on Population Pharmacokinetic Analysis. *Pharmaceutical Research, 23*(6), 1275-1284. doi:10.1007/s11095-006-0205-x

Okudaira, K., Hokari, R., Tsuzuki, Y., Okada, Y., Komoto, S., Watanabe, C., . . . Miura, S. (2009). Blockade of B7-H1 or B7-DC induces an anti-tumor effect in a mouse pancreatic cancer model. *International Journal of Oncology, 35*(4), 741-749. doi:10.3892/ijo_00000387

Oweida, A., Lennon, S., Calame, D., Korpela, S., Bhatia, S., Sharma, J., . . . Karam, S. D. (2017). Ionizing radiation sensitizes tumors to PD-L1 immune checkpoint blockade in orthotopic murine head and neck squamous cell carcinoma. *OncoImmunology, 6*(10), e1356153. doi:10.1080/2162402X.2017.1356153

Papadopoulos, K. P., Crittenden, M. R., Johnson, M. L., Lockhart, A. C., Moore, K. N., Falchook, G. S., . . . Lowy, I. (2016). A first-in-human study of REGN2810, a monoclonal, fully human antibody to programmed death-1 (PD-1), in combination with immunomodulators including hypofractionated radiotherapy (hfRT). *Journal of Clinical Oncology, 34*(15_suppl), 3024-3024. doi:10.1200/JCO.2016.34.15_suppl.3024

Pardoll, D. M. (2012). The blockade of immune checkpoints in cancer immunotherapy. *Nature reviews. Cancer, 12*(4), 252-264. doi:10.1038/nrc3239

Paterson, A. M., Brown, K. E., Keir, M. E., Vanguri, V. K., Riella, L. V., Chandraker, A., . . . Sharpe, A. H. (2011). The PD-L1:B7-1 pathway restrains diabetogenic effector T cells in vivo. *Journal of immunology (Baltimore, Md. : 1950), 187*(3), 1097-1105. doi:10.4049/jimmunol.1003496

Powell, S. F., Gitau, M. M., Sumey, C. J., Reynolds, J. T., Lohr, M., McGraw, S., . . . Spanos, W. C. (2017). Safety of pembrolizumab with chemoradiation (CRT) in locally advanced squamous cell carcinoma of the head and neck (LA-SCCHN). *Journal of Clinical Oncology, 35*(15_suppl), 6011-6011. doi:10.1200/JCO.2017.35.15_suppl.6011

Powles, T., Eder, J. P., Fine, G. D., Braiteh, F. S., Loriot, Y., Cruz, C., . . . Vogelzang, N. J. (2014). MPDL3280A (anti-PD-L1) treatment leads to clinical activity in metastatic bladder cancer. *Nature, 515*, 558. doi:10.1038/nature13904

Rizvi, N. A., Brahmer, J. R., Ou, S.-H. I., Segal, N. H., Khleif, S., Hwu, W.-J., . . . Antonia, S. J. (2015). Safety and clinical activity of MEDI4736, an anti-programmed cell death-ligand 1 (PD-L1) antibody, in patients with non-small cell lung cancer (NSCLC). *Journal of Clinical Oncology, 33*(15_suppl), 8032-8032. doi:10.1200/jco.2015.33.15_suppl.8032

Schadendorf, D., Hodi, F. S., Robert, C., Weber, J. S., Margolin, K., Hamid, O., . . . Wolchok, J. D. (2015). Pooled Analysis of Long-Term Survival Data From Phase II and Phase III Trials of Ipilimumab in Unresectable or Metastatic Melanoma. *Journal of Clinical Oncology, 33*(17), 1889-1894. doi:10.1200/JCO.2014.56.2736

Schultze-Mosgau, S., Grabenbauer, G. G., Radespiel-Troöger, M., Wiltfang, J., Ries, J., Neukam, F. W., & Roödel, F. (2002). Vascularization in the transition area between free grafted soft tissues and pre-irradiated graft bed tissues following preoperative

radiotherapy in the head and neck region. *Head & Neck, 24*(1), 42-51. doi:10.1002/hed.10012

Segal, N. H., Ou, S.-H. I., Balmanoukian, A. S., Fury, M. G., Massarelli, E., Brahmer, J. R., . . . Butler, M. O. (2015). Safety and efficacy of MEDI4736, an anti-PD-L1 antibody, in patients from a squamous cell carcinoma of the head and neck (SCCHN) expansion cohort. *Journal of Clinical Oncology, 33*(15_suppl), 3011-3011. doi:10.1200/jco.2015.33.15_suppl.3011

Siegel, R., Ma, J., Zou, Z., & Jemal, A. (2014). Cancer statistics, 2014. *CA: A Cancer Journal for Clinicians, 64*(1), 9-29. doi:10.3322/caac.21208

Simon, C., Bulut, C., Federspil, P. A., Münter, M. W., Lindel, K., Bergmann, Z., . . . Plinkert, P. K. (2011). Assessment of peri- and postoperative complications and Karnofsky-performance status in head and neck cancer patients after radiation or chemoradiation that underwent surgery with regional or free-flap reconstruction for salvage, palliation, or to improve function. *Radiation Oncology (London, England), 6*, 109-109. doi:10.1186/1748-717X-6-109

Stewart, R., Morrow, M., Hammond, S. A., Mulgrew, K., Marcus, D., Poon, E., . . . McCourt, M. (2015). Identification and Characterization of MEDI4736, an Antagonistic Anti–PD-L1 Monoclonal Antibody. *Cancer Immunology Research, 3*(9), 1052-1062. doi:10.1158/2326-6066.cir-14-0191

Stokes, W. A., Binder, D. C., Jones, B. L., Oweida, A. J., Liu, A. K., Rusthoven, C. G., & Karam, S. D. (2017). Impact of immunotherapy among patients with melanoma brain metastases managed with radiotherapy. *Journal of Neuroimmunology, 313*(Supplement C), 118-122. doi:https://doi.org/10.1016/j.jneuroim.2017.10.006

Tarhini, A. A., & Kirkwood, J. M. (2008). Tremelimumab (CP-675,206): a fully human anticytotoxic T lymphocyte-associated antigen 4 monoclonal antibody for treatment of patients with advanced cancers. *Expert Opinion on Biological Therapy, 8*(10), 1583-1593. doi:10.1517/14712598.8.10.1583

Topalian, S. L., Hodi, F. S., Brahmer, J. R., Gettinger, S. N., Smith, D. C., McDermott, D. F., . . . Sznol, M. (2012). Safety, Activity, and Immune Correlates of Anti–PD-1 Antibody in Cancer. *The New England journal of medicine, 366*(26), 2443-2454. doi:10.1056/NEJMoa1200690

Topalian, S. L., Sznol, M., McDermott, D. F., Kluger, H. M., Carvajal, R. D., Sharfman, W. H., . . . Hodi, F. S. (2014). Survival, Durable Tumor Remission, and Long-Term Safety in Patients With Advanced Melanoma Receiving Nivolumab. *Journal of Clinical Oncology, 32*(10), 1020-1030. doi:10.1200/jco.2013.53.0105

Uppaluri, R., Zolkind, P., Lin, T., Nussenbaum, B., Paniello, R., Rich, J., . . . Adkins, D. (2016). Immunotherapy with pembrolizumab in surgically resectable head and neck squamous cell carcinoma. *Journal of Clinical Oncology, 34*(15_suppl), TPS6110-TPS6110. doi:10.1200/JCO.2016.34.15_suppl.TPS6110

Vanpouille-Box, C., Alard, A., Aryankalayil, M. J., Sarfraz, Y., Diamond, J. M., Schneider, R. J., . . . Demaria, S. (2017). DNA exonuclease Trex1 regulates radiotherapy-induced tumour immunogenicity. *Nat Commun, 8*, 15618. doi:10.1038/ncomms15618

Verastegui, E. L., Morales, R. B., Barrera-Franco, J. L., Poitevin, A. C., & Hadden, J. (2003). Long-term immune dysfunction after radiotherapy to the head and neck area. *Int Immunopharmacol, 3*(8), 1093-1104. doi:10.1016/S1567-5769(03)00013-4

Vermorken, J. B., & Specenier, P. (2010). Optimal treatment for recurrent/metastatic head and neck cancer. *Annals of Oncology, 21*(suppl_7), vii252-vii261. doi:10.1093/annonc/mdq453

Wang, D. D., Zhang, S., Zhao, H., Men, A. Y., & Parivar, K. (2009). Fixed Dosing Versus Body Size—Based Dosing of Monoclonal Antibodies in Adult Clinical Trials. *The Journal of Clinical Pharmacology, 49*(9), 1012-1024. doi:10.1177/0091270009337512

Wang, E., Kang, D., Bae, K.-S., Marshall, M. A., Pavlov, D., & Parivar, K. (2014). Population pharmacokinetic and pharmacodynamic analysis of tremelimumab in patients with metastatic melanoma. *The Journal of Clinical Pharmacology, 54*(10), 1108-1116. doi:10.1002/jcph.309

Wolchok , J. D., Kluger , H., Callahan , M. K., Postow , M. A., Rizvi , N. A., Lesokhin , A. M., . . . Sznol , M. (2013). Nivolumab plus Ipilimumab in Advanced Melanoma. *New England Journal of Medicine, 369*(2), 122-133. doi:10.1056/NEJMoa1302369

Young, K. H., Baird, J. R., Savage, T., Cottam, B., Friedman, D., Bambina, S., . . . Crittenden, M. R. (2016). Optimizing Timing of Immunotherapy Improves Control of Tumors by Hypofractionated Radiation Therapy. *PLoS One, 11*(6), e0157164. doi:10.1371/journal.pone.0157164

Yuan, J., Adamow, M., Ginsberg, B. A., Rasalan, T. S., Ritter, E., Gallardo, H. F., . . . Gnjatic, S. (2011). Integrated NY-ESO-1 antibody and CD8(+) T-cell responses correlate with clinical benefit in advanced melanoma patients treated with ipilimumab. *Proceedings of the National Academy of Sciences of the United States of America, 108*(40), 16723-16728. doi:10.1073/pnas.1110814108

Zhang, C., Wu, S., Zhang, C., Wu, S., Xue, X., Li, M., . . . Zhang, Y. (2008). Anti-tumor immunotherapy by blockade of the PD-1/PD-L1 pathway with recombinant human PD-1–IgV. *Cytotherapy, 10*(7), 711-719. doi:https://doi.org/10.1080/14653240802320237

Zhang, S., Shi, R., Li, C., Parivar, K., & Wang, D. D. (2012). Fixed Dosing Versus Body Size—Based Dosing of Therapeutic Peptides and Proteins in Adults. *The Journal of Clinical Pharmacology, 52*(1), 18-28. doi:10.1177/0091270010388648

Zou, W., & Chen, L. (2008). Inhibitory B7-family molecules in the tumour microenvironment. *Nature Reviews Immunology, 8*, 467. doi:10.1038/nri2326

APPENDIX 1

Dosing Modification and Toxicity Management Guidelines for Immune-Mediated, Infusion-Related, and Non-Immune–Mediated Reactions (MEDI4736 Monotherapy or Combination Therapy With Tremelimumab or Tremelimumab Monotherapy) 17 October 2019 Version (CTCAE v4.03)

General Considerations Regarding Immune-Medicated Reactions

Dose Modifications	Toxicity Management
Drug administration modifications of study drug/study regimen will be made to manage potential immune-related AEs based on severity of treatment-emergent toxicities graded per NCI CTCAE v4.03 (unless indicated otherwise).<br>In addition to the criteria for permanent discontinuation of study drug/study regimen based on CTC grade/severity (table below), permanently discontinue study drug/study regimen for the following conditions:<br>• Inability to reduce corticosteroid to a dose of ≤10 mg of prednisone per day (or equivalent) **within 12 weeks** of the start of the immune-mediated adverse event (imAE)<br>• Grade 3 recurrence of a previously experienced treatment-related imAE following resumption of dosing<br>**Grade 1** No dose modification<br>**Grade 2** Hold study drug/study regimen dose until Grade 2 resolution to Grade ≤1.<br>If toxicity worsens, then treat as Grade 3 or Grade 4.<br>Study drug/study regimen can be resumed once event stabilizes to Grade ≤1 after completion of steroid taper.<br>Patients with endocrinopathies who may require prolonged or continued steroid replacement can be retreated with study drug/study regimen on the following conditions:<br>1. The event stabilizes and is controlled.<br>2. The patient is clinically stable as per Investigator or treating physician's clinical judgement.<br>3. Doses of prednisone are at ≤10 mg/day or equivalent.<br>**Grade 3** Depending on the individual toxicity, study drug/study regimen may be permanently discontinued. Please refer to guidelines below.<br>**Grade 4** Permanently discontinue study drug/study regimen.<br>Note: For asymptomatic amylase or lipase levels of >2.0×ULN, hold study drug/study regimen, and if complete work up shows no evidence of pancreatitis, study drug/study regimen may be continued or resumed.	It is recommended that management of immune-mediated adverse events (imAEs) follows the guidelines presented in this table:<br>– It is possible that events with an inflammatory or immune mediated mechanism could occur in nearly all organs, some of them not noted specifically in these guidelines.<br>– Whether specific immune-mediated events (and/or laboratory indicators of such events) are noted in these guidelines or not, patients should be thoroughly evaluated to rule out any alternative etiology (e.g., disease progression, concomitant medications, and infections) to a possible immune-mediated event. In the absence of a clear alternative etiology, all such events should be managed as if they were immune related. General recommendations follow.<br>– Symptomatic and topical therapy should be considered for low-grade (Grade 1 or 2, unless otherwise specified) events.<br>– For persistent (>3 to 5 days) low-grade (Grade 2) or severe (Grade ≥3) events, promptly start prednisone 1 to 2 mg/kg/day PO or IV equivalent.<br>– Some events with high likelihood for morbidity and/or mortality – e.g., myocarditis, or other similar events even if they are not currently noted in the guidelines – should progress rapidly to high dose IV corticosteroids (methylprednisolone at 2 to 4 mg/kg/day) even if the event is Grade 2, and if clinical suspicion is high and/or there has been clinical confirmation. Consider, as necessary, discussing with the study physician, and promptly pursue specialist consultation.<br>– If symptoms recur or worsen during corticosteroid tapering (28 days of taper), increase the corticosteroid dose (prednisone dose [e.g., up to 2 to 4 mg/kg/day PO or IV equivalent]) until stabilization or improvement of symptoms, then resume corticosteroid tapering at a slower rate (>28 days of taper).<br>– More potent immunosuppressives such as TNF inhibitors (e.g., infliximab; also refer to the individual sections of the imAEs for specific type of immunosuppressive) should be considered for events not responding to

APPENDIX 1

Dosing Modification and Toxicity Management Guidelines for Immune-Mediated, Infusion-Related, and Non-Immune–Mediated Reactions (MEDI4736 Monotherapy or Combination Therapy With Tremelimumab or Tremelimumab Monotherapy) 17 October 2019 Version (CTCAE v4.03)

General Considerations Regarding Immune-Medicated Reactions

Dose Modifications	Toxicity Management
Note: Study drug/study regimen should be permanently discontinued in Grade 3 events with high likelihood for morbidity and/or mortality – e.g., myocarditis, or other similar events even if they are not currently noted in the guidelines. Similarly, consider whether study drug/study regimen should be permanently discontinued in Grade 2 events with high likelihood for morbidity and/or mortality – e.g., myocarditis, or other similar events even if they are not currently noted in the guidelines – when they do not rapidly improve to Grade <1 upon treatment with systemic steroids and following full taper<br>Note: There are some exceptions to permanent discontinuation of study drug for Grade 4 events (i.e., hyperthyroidism, hypothyroidism, Type 1 diabetes mellitus).	systemic steroids. Progression to use of more potent immunosuppressives should proceed more rapidly in events with high likelihood for morbidity and/or mortality – e.g., myocarditis, or other similar events even if they are not currently noted in the guidelines – when these events are not responding to systemic steroids.<br>– With long-term steroid and other immunosuppressive use, consider need for *Pneumocystis jirovecii* pneumonia (PJP, formerly known as *Pneumocystis carinii* pneumonia) prophylaxis, gastrointestinal protection, and glucose monitoring.<br>– Discontinuation of study drug/study regimen is not mandated for Grade 3/Grade 4 inflammatory reactions attributed to local tumor response (e.g., inflammatory reaction at sites of metastatic disease and lymph nodes). Continuation of study drug/study regimen in this situation should be based upon a benefit-risk analysis for that patient.

AE Adverse event; CTC Common Toxicity Criteria; CTCAE Common Terminology Criteria for Adverse Events; imAE immune-mediated adverse event; IV intravenous; NCI National Cancer Institute; PO By mouth.

Pediatric Considerations

Dose Modifications	Toxicity Management
The criteria for permanent discontinuation of study drug/study regimen based on CTC grade/severity is the same for pediatric patients as it is for adult patients, as well as to permanently discontinue study drug/study regimen if unable to reduce corticosteroid ≤ a dose equivalent to that required for corticosteroid replacement therapy **within 12 weeks** after last dose of study drug/study regimen	– All recommendations for specialist consultation should occur with a pediatric specialist in the specialty recommended.<br>– The recommendations for dosing of steroids (i.e., mg/kg/day) and for IV IG and plasmapheresis that are provided for adult patients should also be used for pediatric patients.

Pediatric Considerations	
**Dose Modifications**	**Toxicity Management**
	– The infliximab 5 mg/kg IV dose recommended for adults is the same as recommended for pediatric patients ≥ 6 years old. For dosing in children younger than 6 years old, consult with a pediatric specialist.<br>– For pediatric dosing of mycophenolate mofetil, consult with a pediatric specialist.<br>– With long-term steroid and other immunosuppressive use, consider need for PJP prophylaxis, gastrointestinal protection, and glucose monitoring.

Specific Immune-Mediated Reactions

Adverse Events	Severity Grade of the Event (NCI CTCAE version 4.03)	Dose Modifications	Toxicity Management
**Pneumonitis/Interstitial Lung Disease (ILD)**	**Any Grade**	**General Guidance**	**For Any Grade:**<br>– Monitor patients for signs and symptoms of pneumonitis or ILD (new onset or worsening shortness of breath or cough). Patients should be evaluated with imaging and pulmonary function tests, including other diagnostic procedures as described below.<br>– Initial work-up may include clinical evaluation, monitoring of oxygenation via pulse oximetry (resting and exertion), laboratory work-up, and high- resolution CT scan.
	**Grade 1**<br>(asymptomatic, clinical or diagnostic observations only; intervention not indicated)	No dose modifications required. However, consider holding study drug/study regimen dose as clinically appropriate and during diagnostic work-up for other etiologies.	**For Grade 1 (radiographic changes only):**<br>– Monitor and closely follow up in 2 to 4 days for clinical symptoms, pulse oximetry (resting and exertion), and laboratory work-up and then as clinically indicated.<br>– Consider Pulmonary and Infectious disease consult.
	**Grade 2**<br>(symptomatic; medical intervention indicated; limiting instrumental ADL)	Hold study drug/study regimen dose until Grade 2 resolution to Grade ≤1.<br>• If toxicity worsens, then treat as Grade 3 or Grade 4.<br>• If toxicity improves to Grade ≤1, then the decision to reinitiate study drug/study regimen will be based upon treating physician's clinical judgment and after completion of steroid taper.	**For Grade 2 (mild to moderate new symptoms):**<br>– Monitor symptoms daily and consider hospitalization.<br>– Promptly start systemic steroids (e.g., prednisone 1 to 2 mg/kg/day PO or IV equivalent).<br>– Reimage as clinically indicated.<br>– If no improvement within 3 to 5 days, additional workup should be considered and prompt treatment with IV methylprednisolone 2 to 4 mg/kg/day started<br>– If still no improvement within 3 to 5 days despite IV methylprednisolone at 2 to 4 mg/kg/day, promptly start immunosuppressive therapy such as TNF inhibitors (e.g., infliximab at 5 mg/kg every 2 weeks). Caution: It is

			important to rule out sepsis and refer to infliximab label for general guidance before using infliximab.<br>– Once the patient is improving, gradually taper steroids over ≥28 days and consider prophylactic antibiotics, antifungals, or anti-PJP treatment (refer to current NCCN guidelines for treatment of cancer-related infections [Category 2B recommendation])[a]<br>– Consider pulmonary and infectious disease consult.<br>– Consider, as necessary, discussing with study physician.
	**Grade 3 or 4**<br>(Grade 3: severe symptoms; limiting self-care ADL; oxygen indicated)<br><br>(Grade 4: life-threatening respiratory compromise; urgent intervention indicated [e.g., tracheostomy or intubation])	Permanently discontinue study drug/study regimen.	**For Grade 3 or 4 (severe or new symptoms, new/worsening hypoxia, life-threatening):**<br>– Promptly initiate empiric IV methylprednisolone 1 to 4 mg/kg/day or equivalent.<br>– Obtain Pulmonary and Infectious disease consult; consider, as necessary, discussing with study physician.<br>– Hospitalize the patient.<br>– Supportive care (e.g., oxygen).<br>– If no improvement within 3 to 5 days, additional workup should be considered and prompt treatment with additional immunosuppressive therapy such as TNF inhibitors (e.g., infliximab at 5 mg/kg every 2 weeks' dose) started. Caution: rule out sepsis and refer to infliximab label for general guidance before using infliximab.<br>– Once the patient is improving, gradually taper steroids over ≥28 days and consider prophylactic antibiotics, antifungals, and, in particular, anti-PJP treatment (refer to current NCCN guidelines for treatment of cancer-related infections [Category 2B recommendation]).[a]
**Diarrhea/Colitis**	**Any Grade**	**General Guidance**	**For Any Grade:**<br>– Monitor for symptoms that may be related to diarrhea/enterocolitis (abdominal pain, cramping, or changes in bowel habits such as increased frequency over baseline or

		blood in stool) or related to bowel perforation (such as sepsis, peritoneal signs, and ileus).<br>– Patients should be thoroughly evaluated to rule out any alternative etiology (e.g., disease progression, other medications, or infections), including testing for clostridium difficile toxin, etc.<br>– Steroids should be considered in the absence of clear alternative etiology, even for low-grade events, in order to prevent potential progression to higher grade event.<br>– Use analgesics carefully; they can mask symptoms of perforation and peritonitis.
**Grade 1**<br>(Diarrhea: stool frequency of <4 over baseline per day)<br>(Colitis: asymptomatic; clinical or diagnostic observations only)	No dose modifications.	**For Grade 1:**<br>– Monitor closely for worsening symptoms.<br>– Consider symptomatic treatment, including hydration, electrolyte replacement, dietary changes (e.g., American Dietetic Association colitis diet), and loperamide. Use probiotics as per treating physician's clinical judgment.
**Grade 2**<br>(Diarrhea: stool frequency of 4 to 6 over baseline per day)<br>(Colitis: abdominal pain; mucus or blood in stool)	Hold study drug/study regimen until resolution to Grade ≤1<br>• If toxicity worsens, then treat as Grade 3 or Grade 4.<br>• If toxicity improves to Grade ≤1, then study drug/study regimen can be resumed after completion of steroid taper.	**For Grade 2:**<br>– Consider symptomatic treatment, including hydration, electrolyte replacement, dietary changes (e.g., American Dietetic Association colitis diet), and loperamide and/or budesonide.<br>– Promptly start prednisone 1 to 2 mg/kg/day PO or IV equivalent.<br>– If event is not responsive within 3 to 5 days or worsens despite prednisone at 1 to 2 mg/kg/day PO or IV equivalent, GI consult should be obtained for consideration of further workup, such as imaging and/or colonoscopy, to confirm colitis and rule out perforation, and prompt treatment with IV methylprednisolone 2 to 4 mg/kg/day started.

		– If still no improvement within 3 to 5 days despite 2 to 4 mg/kg IV methylprednisolone, promptly start immunosuppressives such as infliximab at 5 mg/kg once every 2 weeks[a]. **Caution:** it is important to rule out bowel perforation and refer to infliximab label for general guidance before using infliximab.<br>– Consider, as necessary, discussing with study physician if no resolution to Grade ≤1 in 3 to 4 days.<br>– Once the patient is improving, gradually taper steroids over ≥28 days and consider prophylactic antibiotics, antifungals, and anti-PJP treatment (refer to current NCCN guidelines for treatment of cancer-related infections [Category 2B recommendation]).[a]
**Grade 3 or 4**<br>(Grade 3 diarrhea: stool frequency of ≥7 over baseline per day; Grade 4 diarrhea: life threatening consequences)<br>(Grade 3 colitis: severe abdominal pain, change in bowel habits, medi-cal intervention indi-cated, peritoneal signs;<br>Grade 4 colitis: life-threatening consequences, urgent intervention indicated)	**Grade 3**<br>Permanently discontinue study drug/study regimen for Grade 3 if toxicity does not improve to Grade ≤1 within 14 days; study drug/study regimen can be resumed after completion of steroid taper.<br><br>**Grade 4**<br>Permanently discontinue study drug/study regimen.	**For Grade 3 or 4:**<br>– Promptly initiate empiric IV methylprednisolone 2 to 4 mg/kg/day or equivalent.<br>– Monitor stool frequency and volume and maintain hydration.<br>– Urgent GI consult and imaging and/or colonoscopy as appropriate.<br>– If still no improvement within 3 to 5 days of IV methylprednisolone 2 to 4 mg/kg/day or equivalent, promptly start further immunosuppressives (e.g., infliximab at 5 mg/kg once every 2 weeks). **Caution**: Ensure GI consult to rule out bowel perforation and refer to infliximab label for general guidance before using infliximab.<br>– Once the patient is improving, gradually taper steroids over ≥28 days and consider prophylactic antibiotics, antifungals, and anti-PJP treatment (refer to current NCCN guidelines for treatment of cancer-related infections [Category 2B recommendation]).[a]

**Hepatitis (elevated LFTs)**<br>Infliximab should not be used for management of immune-related hepatitis.<br><br>**PLEASE SEE shaded area immediately below this section to find guidance for management of "Hepatitis (elevated LFTS)" in HCC patients**	**Any Grade**	**General Guidance**	**For Any Grade:**<br>– Monitor and evaluate liver function test: AST, ALT, ALP, and TB.<br>– Evaluate for alternative etiologies (e.g., viral hepatitis, disease progression, concomitant medications).
	**Grade 1**<br>(AST or ALT >ULN and ≤3.0×ULN and/or TB > ULN and ≤1.5×ULN)	• No dose modifications.<br>• If it worsens, then treat as Grade 2 event.	**For Grade 1:**<br>– Continue LFT monitoring per protocol.
	**Grade 2**<br>(AST or ALT >3.0×ULN and ≤5.0×ULN and/or TB >1.5×ULN and ≤3.0×ULN)	• Hold study drug/study regimen dose until Grade 2 resolution to Grade ≤1.<br>• If toxicity worsens, then treat as Grade 3 or Grade 4.<br>• If toxicity improves to Grade ≤1 or baseline, resume study drug/study regimen after completion of steroid taper.	**For Grade 2:**<br>– Regular and frequent checking of LFTs (e.g., every 1 to 2 days) until elevations of these are improving or resolved.<br>– If no resolution to Grade ≤1 in 1 to 2 days, consider, as necessary, discussing with study physician.<br>– If event is persistent (>3 to 5 days) or worsens, promptly start prednisone 1 to 2 mg/kg/day PO or IV equivalent.<br>– If still no improvement within 3 to 5 days despite 1 to 2 mg/kg/day of prednisone PO or IV equivalent, consider additional work up and start prompt treatment with IV methylprednisolone 2 to 4 mg/kg/day.<br>– If still no improvement within 3 to 5 days despite 2 to 4 mg/kg/day of IV methylprednisolone, promptly start immunosuppressives (i.e., mycophenolate mofetil).[a] Discuss with study physician if mycophenolate mofetil is not available. **Infliximab should NOT be used.**<br>– Once the patient is improving, gradually taper steroids over ≥28 days and consider prophylactic antibiotics, antifungals, and anti-PJP treatment (refer to current NCCN guidelines for treatment of cancer-related infections [Category 2B recommendation]).[a]

	**Grade 3 or 4**<br>(Grade 3: AST or ALT >5.0×ULN and ≤20.0×ULN and/or TB >3.0×ULN and ≤10.0×ULN)<br><br>(Grade 4: AST or ALT >20×ULN and/or TB >10×ULN)	**For Grade 3:**<br>For elevations in transaminases ≤8 × ULN, or elevations in bilirubin ≤5 × ULN:<br>• Hold study drug/study regimen dose until resolution to Grade ≤1 or baseline<br>• Resume study drug/study regimen if elevations downgrade to Grade ≤1 or baseline within 14 days and after completion of steroid taper.<br>• Permanently discontinue study drug/study regimen if the elevations do not downgrade to Grade ≤1 or baseline within 14 days<br>For elevations in transaminases >8 × ULN or elevations in bilirubin >5 × ULN, discontinue study drug/study regimen.<br>Permanently discontinue study drug/study regimen for any case meeting Hy's law criteria (AST and/or ALT >3 × ULN + bilirubin >2 × ULN without initial findings of cholestasis (i.e., elevated alkaline P04) and in the absence of any alternative cause.[b]<br><br>**For Grade 4:**<br>Permanently discontinue study drug/study regimen.	**For Grade 3 or 4:**<br>– Promptly initiate empiric IV methylprednisolone at 1 to 4 mg/kg/day or equivalent.<br>– If still no improvement within 3 to 5 days despite 1 to 4 mg/kg/day methylprednisolone IV or equivalent, promptly start treatment with immunosuppressive therapy (i.e., mycophenolate mofetil). Discuss with study physician if mycophenolate is not available. **Infliximab should NOT be used.**<br>– Perform hepatology consult, abdominal workup, and imaging as appropriate.<br>– Once the patient is improving, gradually taper steroids over ≥28 days and consider prophylactic antibiotics, antifungals, and anti-PJP treatment (refer to current NCCN guidelines for treatment of cancer-related infections [Category 2B recommendation]).[a]

<table>
<tr>
<td rowspan="3">Hepatitis (elevated LFTs)
Infliximab should not be used for management of immune-related hepatitis.

THIS shaded area is guidance only for management of "Hepatitis (elevated LFTs)" in HCC patients

See instructions at bottom of shaded area if transaminase rise is not isolated but (at any time) occurs in setting of either increasing bilirubin or signs of DILI/liver decompensation</td>
<td>Any Grade</td>
<td>General Guidance</td>
<td>For Any Grade:
– Monitor and evaluate liver function test: AST, ALT, ALP, and TB.
– Evaluate for alternative etiologies (e.g., viral hepatitis, disease progression, concomitant medications, worsening of liver cirrhosis [e.g., portal vein thrombosis]).
– For HBV+ patients: evaluate quantitative HBV viral load, quantitative HBsAg, or HBeAg
– For HCV+ patients: evaluate quantitative HCV viral load
– Consider consulting hepatologist/Infectious disease specialist regarding change/implementation in/of antiviral medications for any patient with an elevated HBV viral load >2000 IU/ml
– Consider consulting hepatologist/Infectious disease specialist regarding change/implementation in/of antiviral HCV medications if HCV viral load increased by ≥2-fold
– For HCV+ with HBcAB+: Evaluate for both HBV and HCV as above</td>
</tr>
<tr>
<td>Grade 1
(Isolated AST or ALT >ULN and ≤5.0×ULN, whether normal or elevated at baseline)</td>
<td>• No dose modifications.
• If ALT/AST elevations represents significant worsening based on investigator assessment, then treat as Grade 2 event.
For all grades, see instructions at bottom of shaded area if transaminase rise is not isolated but (at any time) occurs in setting of either increasing bilirubin or signs of DILI/liver decompensation</td>
<td></td>
</tr>
<tr>
<td>Grade 2
(Isolated AST or ALT >5.0×ULN and</td>
<td>• Hold study drug/study regimen dose until Grade 2 resolution to Grade ≤1 or baseline.</td>
<td>For Grade 2:
– Regular and frequent checking of LFTs (e.g., every 1 to 3 days) until elevations of these are improving or resolved.</td>
</tr>
</table>

	≤8.0×ULN, if normal at baseline)<br><br>(Isolated AST or ALT >2.0×baseline and ≤12.5×ULN, if elevated >ULN at baseline)	• If toxicity worsens, then treat as Grade 3 or Grade 4.<br>If toxicity improves to Grade ≤1 or baseline, resume study drug/study regimen after completion of steroid taper.	– Recommend consult hepatologist; consider abdominal ultrasound, including Doppler assessment of liver perfusion.<br>– Consider, as necessary, discussing with study physician.<br>– If event is persistent (>3 to 5 days) or worsens, and investigator suspects toxicity to be immune-mediated AE, recommend to start prednisone 1 to 2 mg/kg/day PO or IV equivalent.<br>– If still no improvement within 3 to 5 days despite 1 to 2 mg/kg/day of prednisone PO or IV equivalent, consider additional workup and treatment with IV methylprednisolone 2 to 4 mg/kg/day.<br>– If still no improvement within 3 to 5 days despite 2 to 4 mg/kg/day of IV methylprednisolone, consider additional abdominal workup (including liver biopsy) and imaging (i.e., liver ultrasound), and consider starting immunosuppressives (i.e., mycophenolate mofetil).[a] Discuss with study physician if mycophenolate mofetil is not available. **Infliximab should NOT be used.**
	**Grade 3**<br>(Isolated AST or ALT >8.0×ULN and ≤20.0×ULN, if normal at baseline)<br><br>(Isolated AST or ALT >12.5×ULN and ≤20.0×ULN, if elevated >ULN at baseline)	• Hold study drug/study regimen dose until resolution to Grade ≤1 or baseline<br>• Resume study drug/study regimen if elevations downgrade to Grade ≤1 or baseline within 14 days and after completion of steroid taper.<br>• Permanently discontinue study drug/study regimen if the elevations do not downgrade to Grade ≤1 or baseline within 14 days<br>Permanently discontinue study drug/study regimen for any case meeting	**For Grade 3:**<br>– Regular and frequent checking of LFTs (e.g., every 1-2 days) until elevations of these are improving or resolved.<br>– Consult hepatologist (unless investigator is hepatologist); obtain abdominal ultrasound, including Doppler assessment of liver perfusion; and consider liver biopsy.<br>– Consider, as necessary, discussing with study physician.<br>– If investigator suspects toxicity to be immune-mediated, promptly initiate empiric IV methylprednisolone at 1 to 4 mg/kg/day or equivalent.<br>– If no improvement within 3 to 5 days despite 1 to 4 mg/kg/day methylprednisolone IV or equivalent, obtain liver biopsy (if it has not been done already) and promptly start treatment with immunosuppressive therapy

		Hy's law criteria, in the absence of any alternative cause.[b]	(mycophenolate mofetil). Discuss with study physician if mycophenolate is not available. **Infliximab should NOT be used.**<br>– Once the patient is improving, gradually taper steroids over ≥28 days and consider prophylactic antibiotics, antifungals, and anti-PCP treatment (refer to current NCCN guidelines for treatment of cancer-related infections [Category 2B recommendation]).[a]
	**Grade 4**<br>(Isolated AST or ALT >20×ULN, whether normal or elevated at baseline)	Permanently discontinue study drug/study regimen.	**For Grade 4:**<br>**Same as above**<br>**(except would recommend obtaining liver biopsy early)**
**If transaminase rise is not isolated but (at any time) occurs in setting of either increasing total/direct bilirubin (≥1.5×ULN, if normal at baseline; or 2×baseline, if >ULN at baseline) or signs of DILI/liver decompensation (e.g., fever, elevated INR):**<br>- **Manage dosing for Grade 1 transaminase rise as instructed for Grade 2 transaminase rise**<br>- **Manage dosing for Grade 2 transaminase rise as instructed for Grade 3 transaminase rise**<br>- **Grade 3-4: Permanently discontinue study drug/study regimen**			
**Nephritis or renal dysfunction**<br>(elevated serum creatinine)	**Any Grade**	**General Guidance**	**For Any Grade:**<br>– Consult with nephrologist.<br>– Monitor for signs and symptoms that may be related to changes in renal function (e.g., routine urinalysis, elevated serum BUN and creatinine, decreased creatinine clearance, electrolyte imbalance, decrease in urine output, or proteinuria).<br>– Patients should be thoroughly evaluated to rule out any alternative etiology (e.g., disease progression or infections).

			– Steroids should be considered in the absence of clear alternative etiology even for low-grade events (Grade 2), in order to prevent potential progression to higher grade event.
	**Grade 1**<br>(Serum creatinine > 1 to 1.5 × baseline; > ULN to 1.5 × ULN)	No dose modifications.	**For Grade 1:**<br>– Monitor serum creatinine weekly and any accompanying symptoms.<br>• If creatinine returns to baseline, resume its regular monitoring per study protocol.<br>• If creatinine worsens, depending on the severity, treat as Grade 2, 3, or 4.<br>– Consider symptomatic treatment, including hydration, electrolyte replacement, and diuretics.
	**Grade 2**<br>(serum creatinine >1.5 to 3.0 × baseline; >1.5 to 3.0 × ULN)	Hold study drug/study regimen until resolution to Grade ≤1 or baseline.<br>• If toxicity worsens, then treat as Grade 3 or 4.<br>• If toxicity improves to Grade ≤1 or baseline, then resume study drug/study regimen after completion of steroid taper.	**For Grade 2:**<br>– Consider symptomatic treatment, including hydration, electrolyte replacement, and diuretics.<br>– Carefully monitor serum creatinine every 2 to 3 days and as clinically warranted.<br>– Consult nephrologist and consider renal biopsy if clinically indicated.<br>– If event is persistent (>3 to 5 days) or worsens, promptly start prednisone 1 to 2 mg/kg/day PO or IV equivalent.<br>– If event is not responsive within 3 to 5 days or worsens despite prednisone at 1 to 2 mg/kg/day PO or IV equivalent, additional workup should be considered and prompt treatment with IV methylprednisolone at 2 to 4 mg/kg/day started.<br>– Once the patient is improving, gradually taper steroids over ≥28 days and consider prophylactic antibiotics, antifungals, and anti-PJP treatment (refer to current NCCN guidelines for treatment of cancer-related infections [Category 2B recommendation]).[a]

			– When event returns to baseline, resume study drug/study regimen and routine serum creatinine monitoring per study protocol.
	**Grade 3 or 4**<br>(Grade 3: serum creatinine >3.0 × baseline; >3.0 to 6.0 × ULN;<br><br>Grade 4: serum creatinine >6.0 × ULN)	Permanently discontinue study drug/study regimen.	**For Grade 3 or 4:**<br>– Carefully monitor serum creatinine on daily basis.<br>– Consult nephrologist and consider renal biopsy if clinically indicated.<br>– Promptly start prednisone 1 to 2 mg/kg/day PO or IV equivalent.<br>– If event is not responsive within 3 to 5 days or worsens despite prednisone at 1 to 2 mg/kg/day PO or IV equivalent, additional workup should be considered and prompt treatment with IV methylprednisolone 2 to 4 mg/kg/day started.<br>– Once the patient is improving, gradually taper steroids over ≥28 days and consider prophylactic antibiotics, antifungals, and anti-PJP treatment (refer to current NCCN guidelines for treatment of cancer-related infections [Category 2B recommendation]).[a]
**Rash**<br>(excluding bullous skin formations)	**Any Grade**<br>(refer to NCI CTCAE v 4.03 for definition of severity/grade depending on type of skin rash)	**General Guidance**	**For Any Grade:**<br>– Monitor for signs and symptoms of dermatitis (rash and pruritus).<br>– IF THERE IS ANY BULLOUS FORMATION, THE STUDY PHYSICIAN SHOULD BE CONTACTED **AND STUDY DRUG DISCONTINUED.**
	**Grade 1**	No dose modifications.	**For Grade 1:**<br>– Consider symptomatic treatment, including oral antipruritics (e.g., diphenhydramine or hydroxyzine) and topical therapy (e.g., urea cream).
	**Grade 2**	For persistent (>1 to 2 weeks) Grade 2 events, hold scheduled study drug/study	**For Grade 2:**<br>– Obtain dermatology consult.

		regimen until resolution to Grade ≤1 or baseline.<br>• If toxicity worsens, then treat as Grade 3.<br>• If toxicity improves to Grade ≤1 or baseline, then resume drug/study regimen after completion of steroid taper.	– Consider symptomatic treatment, including oral antipruritics (e.g., diphenhydramine or hydroxyzine) and topical therapy (e.g., urea cream).<br>– Consider moderate-strength topical steroid.<br>– If no improvement of rash/skin lesions occurs within 3 to 5 days or is worsening despite symptomatic treatment and/or use of moderate strength topical steroid, consider, as necessary, discussing with study physician and promptly start systemic steroids such as prednisone 1 to 2 mg/kg/day PO or IV equivalent.<br>– Consider skin biopsy if the event is persistent for >1 to 2 weeks or recurs.
	**Grade 3 or 4**	**For Grade 3:**<br>Hold study drug/study regimen until resolution to Grade ≤1 or baseline.<br>If temporarily holding the study drug/study regimen does not provide improvement of the Grade 3 skin rash to Grade ≤1 or baseline within 30 days, then permanently discontinue study drug/study regimen.<br><br>**For Grade 4:**<br>Permanently discontinue study drug/study regimen.	**For Grade 3 or 4:**<br>– Consult dermatology.<br>– Promptly initiate empiric IV methylprednisolone 1 to 4 mg/kg/day or equivalent.<br>– Consider hospitalization.<br>– Monitor extent of rash [Rule of Nines].<br>– Consider skin biopsy (preferably more than 1) as clinically feasible.<br>– Once the patient is improving, gradually taper steroids over ≥28 days and consider prophylactic antibiotics, antifungals, and anti-PJP treatment (refer to current NCCN guidelines for treatment of cancer-related infections [Category 2B recommendation]).[a]<br>– Consider, as necessary, discussing with study physician.
**Endocrinopathy**<br>(e.g., hyperthyroidism, hypothyroidism, Type 1 diabetes mellitus, hypophysitis,	**Any Grade**<br>(depending on the type of endocrinopathy, refer to NCI CTCAE	**General Guidance**	**For Any Grade:**<br>– Consider consulting an endocrinologist for endocrine events.<br>– Consider, as necessary, discussing with study physician.<br>– Monitor patients for signs and symptoms of endocrinopathies. Non-specific symptoms include headache, fatigue, behavior

hypopituitarism, and adrenal insufficiency; exocrine event of amylase/lipase increased also included in this section)	v4.03 for defining the CTC grade/severity)		changes, changed mental status, vertigo, abdominal pain, unusual bowel habits, polydipsia, polyuria, hypotension, and weakness.<br>– Patients should be thoroughly evaluated to rule out any alternative etiology (e.g., disease progression including brain metastases, or infections).<br>– Depending on the suspected endocrinopathy, monitor and evaluate thyroid function tests: TSH, total T3 and free T4 and other relevant endocrine and related labs (e.g., blood glucose and ketone levels, HgA1c).<br>– For modest asymptomatic elevations in serum amylase and lipase, corticosteroid treatment is not indicated as long as there are no other signs or symptoms of pancreatic inflammation.<br>– If a patient experiences an AE that is thought to be possibly of autoimmune nature (e.g., thyroiditis, pancreatitis, hypophysitis, or diabetes insipidus), the investigator should send a blood sample for appropriate autoimmune antibody testing.
	**Grade 1**	No dose modifications.	**For Grade 1 (including those with asymptomatic TSH elevation):**<br>– Monitor patient with appropriate endocrine function tests.<br>– For suspected hypophysitis/hypopituitarism, consider consultation of an endocrinologist to guide assessment of early-morning ACTH, cortisol, TSH and free T4; also consider gonadotropins, sex hormones, and prolactin levels, as well as cosyntropin stimulation test (though it may not be useful in diagnosing early secondary adrenal insufficiency).<br>– If TSH < 0.5 × LLN, or TSH >2 × ULN, or consistently out of range in 2 subsequent measurements, include free T4 at subsequent cycles as clinically indicated and consider consultation of an endocrinologist.<br>–

**Grade 2**	For Grade 2 endocrinopathy other than hypothyroidism and Type 1 diabetes mellitus, hold study drug/study regimen dose until patient is clinically stable.<br>• If toxicity worsens, then treat as Grade 3 or Grade 4.<br>Study drug/study regimen can be resumed once event stabilizes and after completion of steroid taper.<br>Patients with endocrinopathies who may require prolonged or continued steroid replacement (e.g., adrenal insufficiency) can be retreated with study drug/study regimen on the following conditions:<br>1. The event stabilizes and is controlled.<br>2. The patient is clinically stable as per investigator or treating physician's clinical judgement.<br>3. Doses of prednisone are ≤10 mg/day or equivalent.	**For Grade 2 (including those with symptomatic endocrinopathy):**<br>– Consult endocrinologist to guide evaluation of endocrine function and, as indicated by suspected endocrinopathy and as clinically indicated, consider pituitary scan.<br>– For all patients with abnormal endocrine work up, except those with isolated hypothyroidism or Type 1 DM, and as guided by an endocrinologist, consider short-term corticosteroids (e.g., 1 to 2 mg/kg/day methylprednisolone or IV equivalent) and prompt initiation of treatment with relevant hormone replacement (e.g., hydrocortisone, sex hormones).<br>– Isolated hypothyroidism may be treated with replacement therapy, without study drug/study regimen interruption, and without corticosteroids.<br>– Isolated Type 1 diabetes mellitus (DM) may be treated with appropriate diabetic therapy, without study drug/study regimen interruption, and without corticosteroids.<br>– Once patients on steroids are improving, gradually taper immunosuppressive steroids (as appropriate and with guidance of endocrinologist) over ≥28 days and consider prophylactic antibiotics, antifungals, and anti-PJP treatment (refer to current NCCN guidelines for treatment of cancer-related infections [Category 2B recommendation]).[a]<br>– For patients with normal endocrine workup (laboratory assessment or MRI scans), repeat laboratory assessments/MRI as clinically indicated.
**Grade 3 or 4**	For Grade 3 or 4 endocrinopathy other than hypothyroidism and Type 1 diabetes mellitus, hold study drug/study regimen dose until endocrinopathy symptom(s) are controlled.	**For Grade 3 or 4:**<br>– Consult endocrinologist to guide evaluation of endocrine function and, as indicated by suspected endocrinopathy and as clinically indicated, consider pituitary scan. Hospitalization recommended.<br>– For all patients with abnormal endocrine work up, except those with isolated hypothyroidism or Type 1 DM, and as

		Study drug/study regimen can be resumed once event stabilizes and after completion of steroid taper.<br>Patients with endocrinopathies who may require prolonged or continued steroid replacement (e.g., adrenal insufficiency) can be retreated with study drug/study regimen on the following conditions:<br>1. The event stabilizes and is controlled.<br>2. The patient is clinically stable as per investigator or treating physician's clinical judgement.<br>3. Doses of prednisone are ≤10 mg/day or equivalent.	guided by an endocrinologist, promptly initiate empiric IV methylprednisolone 1 to 2 mg/kg/day or equivalent, as well as relevant hormone replacement (e.g., hydrocortisone, sex hormones).<br>– For adrenal crisis, severe dehydration, hypotension, or shock, immediately initiate IV corticosteroids with mineralocorticoid activity.<br>– Isolated hypothyroidism may be treated with replacement therapy, without study drug/study regimen interruption, and without corticosteroids.<br>– Isolated Type 1 diabetes mellitus may be treated with appropriate diabetic therapy, without study drug/study regimen interruption, and without corticosteroids.<br>– Once patients on steroids are improving, gradually taper immunosuppressive steroids (as appropriate and with guidance of endocrinologist) over ≥28 days and consider prophylactic antibiotics, antifungals, and anti-PJP treatment (refer to current NCCN guidelines for treatment of cancer-related infections [Category 2B recommendation]).[a]
**Neurotoxicity**<br>(to include but not be limited to limbic encephalitis and autonomic neuropathy, excluding Myasthenia Gravis and Guillain-Barre)	**Any Grade**<br>(depending on the type of neurotoxicity, refer to NCI CTCAE v4.03 for defining the CTC grade/severity)	**General Guidance**	**For Any Grade:**<br>– Patients should be evaluated to rule out any alternative etiology (e.g., disease progression, infections, metabolic syndromes, or medications).<br>– Monitor patient for general symptoms (headache, nausea, vertigo, behavior change, or weakness).<br>– Consider appropriate diagnostic testing (e.g., electromyogram and nerve conduction investigations).<br>– Perform symptomatic treatment with neurological consult as appropriate.<br>–
	**Grade 1**	No dose modifications.	**For Grade 1:**<br>– See "Any Grade" recommendations above.

	**Grade 2**	For acute motor neuropathies or neurotoxicity, hold study drug/study regimen dose until resolution to Grade ≤1.<br>For sensory neuropathy/neuropathic pain, consider holding study drug/study regimen dose until resolution to Grade ≤1.<br>If toxicity worsens, then treat as Grade 3 or 4.<br>Study drug/study regimen can be resumed once event improves to Grade ≤1 and after completion of steroid taper.	**For Grade 2:**<br>– Consider, as necessary, discussing with the study physician.<br>– Obtain neurology consult.<br>– Sensory neuropathy/neuropathic pain may be managed by appropriate medications (e.g., gabapentin or duloxetine).<br>– Promptly start systemic steroids prednisone 1 to 2 mg/kg/day PO or IV equivalent.<br>– If no improvement within 3 to 5 days despite 1 to 2 mg/kg/day prednisone PO or IV equivalent, consider additional workup and promptly treat with additional immunosuppressive therapy (e.g., IV IG).
	**Grade 3 or 4**	**For Grade 3:**<br>Hold study drug/study regimen dose until resolution to Grade ≤1.<br>Permanently discontinue study drug/study regimen if Grade 3 imAE does not resolve to Grade ≤1 within 30 days.<br>**For Grade 4:**<br>Permanently discontinue study drug/study regimen.	**For Grade 3 or 4:**<br>– Consider, as necessary, discussing with study physician.<br>– Obtain neurology consult.<br>– Consider hospitalization.<br>– Promptly initiate empiric IV methylprednisolone 1 to 2 mg/kg/day or equivalent.<br>– If no improvement within 3 to 5 days despite IV corticosteroids, consider additional workup and promptly treat with additional immunosuppressants (e.g., IV IG).<br>– Once stable, gradually taper steroids over ≥28 days.
**Peripheral neuromotor syndromes**<br>(such as Guillain-Barre and myasthenia gravis)	**Any Grade**	**General Guidance**	**For Any Grade:**<br>– The prompt diagnosis of immune-mediated peripheral neuromotor syndromes is important, since certain patients may unpredictably experience acute decompensations that can result in substantial morbidity or in the worst case, death. Special care should be taken for certain sentinel symptoms that may predict a more severe outcome, such as prominent

		dysphagia, rapidly progressive weakness, and signs of respiratory insufficiency or autonomic instability.<br>– Patients should be evaluated to rule out any alternative etiology (e.g., disease progression, infections, metabolic syndromes or medications). It should be noted that the diagnosis of immune-mediated peripheral neuromotor syndromes can be particularly challenging in patients with underlying cancer, due to the multiple potential confounding effects of cancer (and its treatments) throughout the neuraxis. Given the importance of prompt and accurate diagnosis, it is essential to have a low threshold to obtain a neurological consult.<br>– Neurophysiologic diagnostic testing (e.g., electromyogram and nerve conduction investigations, and "repetitive stimulation" if myasthenia is suspected) are routinely indicated upon suspicion of such conditions and may be best facilitated by means of a neurology consultation.<br>– It is important to consider that the use of steroids as the primary treatment of Guillain-Barre is not typically considered effective. Patients requiring treatment should be started with IV IG and followed by plasmapheresis if not responsive to IV IG.
**Grade 1**	No dose modifications.	**For Grade 1:**<br>– Consider, as necessary, discussing with the study physician.<br>– Care should be taken to monitor patients for sentinel symptoms of a potential decompensation as described above.<br>– Obtain a neurology consult.
**Grade 2**	Hold study drug/study regimen dose until resolution to Grade ≤1.<br>Permanently discontinue study drug/study regimen if it does not resolve to Grade ≤1 within 30 days or if there are	**For Grade 2:**<br>– Consider, as necessary, discussing with the study physician.<br>– Care should be taken to monitor patients for sentinel symptoms of a potential decompensation as described above.<br>– Obtain a neurology consult

	signs of respiratory insufficiency or autonomic instability.	– Sensory neuropathy/neuropathic pain may be managed by appropriate medications (e.g., gabapentin or duloxetine).<br>*MYASTHENIA GRAVIS:*<br>○ Steroids may be successfully used to treat myasthenia gravis. It is important to consider that steroid therapy (especially with high doses) may result in transient worsening of myasthenia and should typically be administered in a monitored setting under supervision of a consulting neurologist.<br>○ Patients unable to tolerate steroids may be candidates for treatment with plasmapheresis or IV IG. Such decisions are best made in consultation with a neurologist, taking into account the unique needs of each patient.<br>○ If myasthenia gravis-like neurotoxicity is present, consider starting AChE inhibitor therapy in addition to steroids. Such therapy, if successful, can also serve to reinforce the diagnosis.<br>*GUILLAIN-BARRE:*<br>○ It is important to consider here that the use of steroids as the primary treatment of Guillain-Barre is not typically considered effective.<br>○ Patients requiring treatment should be started with IV IG and followed by plasmapheresis if not responsive to IV IG.
**Grade 3 or 4**	**For Grade 3:**<br>Hold study drug/study regimen dose until resolution to Grade ≤1.<br>Permanently discontinue study drug/study regimen if Grade 3 imAE does not resolve to Grade ≤1 within 30	**For Grade 3 or 4 (severe or life-threatening events):**<br>– Consider, as necessary, discussing with study physician.<br>– Recommend hospitalization.<br>– Monitor symptoms and obtain neurological consult.<br>*MYASTHENIA GRAVIS:*

		days or if there are signs of respiratory insufficiency or autonomic instability.<br><br>**For Grade 4:**<br>Permanently discontinue study drug/study regimen.	○ Steroids may be successfully used to treat myasthenia gravis. They should typically be administered in a monitored setting under supervision of a consulting neurologist.<br>○ Patients unable to tolerate steroids may be candidates for treatment with plasmapheresis or IV IG.<br>○ If myasthenia gravis-like neurotoxicity present, consider starting AChE inhibitor therapy in addition to steroids. Such therapy, if successful, can also serve to reinforce the diagnosis.<br>*GUILLAIN-BARRE:*<br>○ It is important to consider here that the use of steroids as the primary treatment of Guillain-Barre is not typically considered effective.<br>○ Patients requiring treatment should be started with IV IG and followed by plasmapheresis if not responsive to IV IG.
**Myocarditis**	**Any Grade**	**General Guidance**<br>Discontinue drug permanently if biopsy-proven immune-mediated myocarditis.	**For Any Grade:**<br>– The prompt diagnosis of immune-mediated myocarditis is important, particularly in patients with baseline cardiopulmonary disease and reduced cardiac function.<br>– Consider, as necessary, discussing with the study physician.<br>– Monitor patients for signs and symptoms of myocarditis (new onset or worsening chest pain, arrhythmia, shortness of breath, peripheral edema). As some symptoms can overlap with lung toxicities, simultaneously evaluate for and rule out pulmonary toxicity as well as other causes (e.g., pulmonary embolism, congestive heart failure, malignant pericardial effusion). A Cardiology consultation should be obtained early, with prompt assessment of whether and when to complete a cardiac biopsy, including any other diagnostic procedures.

		– Initial work-up should include clinical evaluation, BNP, cardiac enzymes, ECG, echocardiogram (ECHO), monitoring of oxygenation via pulse oximetry (resting and exertion), and additional laboratory work-up as indicated. Spiral CT or cardiac MRI can complement ECHO to assess wall motion abnormalities when needed.<br>– Patients should be thoroughly evaluated to rule out any alternative etiology (e.g., disease progression, other medications, or infections)
**Grade 1**<br>(asymptomatic with laboratory (e.g., BNP) or cardiac imaging abnormalities)	No dose modifications required unless clinical suspicion is high, in which case hold study drug/study regimen dose during diagnostic work-up for other etiologies. If study drug/study regimen is held, resume after complete resolution to Grade 0.	**For Grade 1 (no definitive findings):**<br>- Monitor and closely follow up in 2 to 4 days for clinical symptoms, BNP, cardiac enzymes, ECG, ECHO, pulse oximetry (resting and exertion), and laboratory work-up as clinically indicated.<br>- Consider using steroids if clinical suspicion is high.
**Grade 2, 3 or 4**<br>(Grade 2: Symptoms with mild to moderate activity or exertion)<br>(Grade 3: Severe with symptoms at rest or with minimal activity or exertion; intervention indicated)<br>(Grade 4: Life-threatening consequences; urgent intervention indicated (e.g., continuous IV therapy or mechanical	- If Grade 2 -- Hold study drug/study regimen dose until resolution to Grade 0. If toxicity rapidly improves to Grade 0, then the decision to reinitiate study drug/study regimen will be based upon treating physician's clinical judgment and after completion of steroid taper. If toxicity does not rapidly improve, permanently. discontinue study drug/study regimen.<br>If Grade 3-4, permanently discontinue study drug/study regimen.	**For Grade 2-4:**<br>– Monitor symptoms daily, hospitalize.<br>– Promptly start IV methylprednisolone 2 to 4 mg/kg/day or equivalent after Cardiology consultation has determined whether and when to complete diagnostic procedures including a cardiac biopsy.<br>– Supportive care (e.g., oxygen).<br>– If no improvement within 3 to 5 days despite IV methylprednisolone at 2 to 4 mg/kg/day, promptly start immunosuppressive therapy such as TNF inhibitors (e.g., infliximab at 5 mg/kg every 2 weeks). Caution: It is important to rule out sepsis and refer to infliximab label for general guidance before using infliximab.<br>– Once the patient is improving, gradually taper steroids over ≥28 days and consider prophylactic antibiotics, antifungals, or anti-PJP treatment (refer to current NCCN guidelines for

	hemodynamic support))		treatment of cancer-related infections [Category 2B recommendation]).[a]
**Myositis/Polymyositis (“Poly/myositis”)**	**Any Grade**	**General Guidance**	**For Any Grade:**<br>– Monitor patients for signs and symptoms of poly/myositis. Typically, muscle weakness/pain occurs in proximal muscles including upper arms, thighs, shoulders, hips, neck and back, but rarely affects the extremities including hands and fingers; also difficulty breathing and/or trouble swallowing can occur and progress rapidly. Increased general feelings of tiredness and fatigue may occur, and there can be new-onset falling, difficulty getting up from a fall, and trouble climbing stairs, standing up from a seated position, and/or reaching up.<br>– If poly/myositis is suspected, a Neurology consultation should be obtained early, with prompt guidance on diagnostic procedures. Myocarditis may co-occur with poly/myositis; refer to guidance under Myocarditis. Given breathing complications, refer to guidance under Pneumonitis/ILD. Given possibility of an existent (but previously unknown) autoimmune disorder, consider Rheumatology consultation.<br>– Consider, as necessary, discussing with the study physician.<br>– Initial work-up should include clinical evaluation, creatine kinase, aldolase, LDH, BUN/creatinine, erythrocyte sedimentation rate or C-reactive protein level, urine myoglobin, and additional laboratory work-up as indicated, including a number of possible rheumatological/antibody tests (i.e., consider whether a rheumatologist consultation is indicated and could guide need for rheumatoid factor, antinuclear antibody, anti-smooth muscle, antisynthetase [such as anti-Jo-1], and/or signal-recognition particle antibodies). Confirmatory testing may include electromyography, nerve conduction studies, MRI of the muscles, and/or a muscle biopsy. Consider Barium swallow for evaluation of dysphagia or dysphonia.

		Patients should be thoroughly evaluated to rule out any alternative etiology (e.g., disease progression, other medications, or infections).
**Grade 1**<br>(mild pain)	- No dose modifications.	**For Grade 1:**<br>– Monitor and closely follow up in 2 to 4 days for clinical symptoms and initiate evaluation as clinically indicated.<br>– Consider Neurology consult.<br>– Consider, as necessary, discussing with the study physician.
**Grade 2**<br>(moderate pain associated with weakness; pain limiting instrumental activities of daily living [ADLs])	Hold study drug/study regimen dose until resolution to Grade ≤1.<br>- Permanently discontinue study drug/study regimen if it does not resolve to Grade ≤1 within 30 days or if there are signs of respiratory insufficiency.	**For Grade 2:**<br>– Monitor symptoms daily and consider hospitalization.<br>– Obtain Neurology consult, and initiate evaluation.<br>– Consider, as necessary, discussing with the study physician.<br>– If clinical course is rapidly progressive (particularly if difficulty breathing and/or trouble swallowing), promptly start IV methylprednisolone 2 to 4 mg/kg/day systemic steroids along with receiving input from Neurology consultant<br>– If clinical course is *not* rapidly progressive, start systemic steroids (e.g., prednisone 1 to 2 mg/kg/day PO or IV equivalent); if no improvement within 3 to 5 days, continue additional work up and start treatment with IV methylprednisolone 2 to 4 mg/kg/day<br>– If after start of IV methylprednisolone at 2 to 4 mg/kg/day there is no improvement within 3 to 5 days, consider start of immunosuppressive therapy such as TNF inhibitors (e.g., infliximab at 5 mg/kg every 2 weeks). Caution: It is important to rule out sepsis and refer to infliximab label for general guidance before using infliximab.<br>– Once the patient is improving, gradually taper steroids over ≥28 days and consider prophylactic antibiotics, antifungals, or anti-PJP treatment (refer to current NCCN guidelines for treatment of cancer-related infections [Category 2B recommendation]).[a]

	**Grade 3 or 4**<br>(pain associated with severe weakness; limiting self-care ADLs)	**For Grade 3:**<br>Hold study drug/study regimen dose until resolution to Grade ≤1.<br>Permanently discontinue study drug/study regimen if Grade 3 imAE does not resolve to Grade ≤1 within 30 days or if there are signs of respiratory insufficiency.<br><br>**For Grade 4:**<br>- Permanently discontinue study drug/study regimen.	**For Grade 3 or 4 (severe or life-threatening events):**<br>– Monitor symptoms closely; recommend hospitalization.<br>– Obtain Neurology consult, and complete full evaluation.<br>– Consider, as necessary, discussing with the study physician.<br>– Promptly start IV methylprednisolone 2 to 4 mg/kg/day systemic steroids along with receiving input from Neurology consultant.<br>– If after start of IV methylprednisolone at 2 to 4 mg/kg/day there is no improvement within 3 to 5 days, consider start of immunosuppressive therapy such as TNF inhibitors (e.g., infliximab at 5 mg/kg every 2 weeks). Caution: It is important to rule out sepsis and refer to infliximab label for general guidance before using infliximab.<br>– Consider whether patient may require IV IG, plasmapheresis.<br>– Once the patient is improving, gradually taper steroids over ≥28 days and consider prophylactic antibiotics, antifungals, or anti-PJP treatment (refer to current NCCN guidelines for treatment of cancer-related infections [Category 2B recommendation]).[a]

[a]ASCO Educational Book 2015 “Managing Immune Checkpoint Blocking Antibody Side Effects” by Michael Postow MD.
[b]FDA Liver Guidance Document 2009 Guidance for Industry: Drug Induced Liver Injury – Premarketing Clinical Evaluation.
AChE Acetylcholine esterase; ADL Activities of daily living; AE Adverse event; ALP Alkaline phosphatase test; ALT Alanine aminotransferase; AST Aspartate aminotransferase; BUN Blood urea nitrogen; CT Computed tomography; CTCAE Common Terminology Criteria for Adverse Events; ILD Interstitial lung disease; imAE immune-mediated adverse event; IG Immunoglobulin; IV Intravenous; GI Gastrointestinal; LFT Liver function tests; LLN Lower limit of normal; MRI Magnetic resonance imaging; NCI National Cancer Institute; NCCN National Comprehensive Cancer Network; PJP *Pneumocystis jirovecii* pneumonia (formerly known as *Pneumocystis carinii* pneumonia); PO By mouth; T3 Triiodothyronine; T4 Thyroxine; TB Total bilirubin; TNF Tumor necrosis factor; TSH Thyroid-stimulating hormone; ULN Upper limit of normal.

Infusion-Related Reactions

Severity Grade of the Event (NCI CTCAE version 4.03)	Dose Modifications	Toxicity Management
**Any Grade**	General Guidance	**For Any Grade:**<br>– Manage per institutional standard at the discretion of investigator.<br>– Monitor patients for signs and symptoms of infusion-related reactions (e.g., fever and/or shaking chills, flushing and/or itching, alterations in heart rate and blood pressure, dyspnea or chest discomfort, or skin rashes) and anaphylaxis (e.g., generalized urticaria, angioedema, wheezing, hypotension, or tachycardia).
**Grade 1 or 2**	**For Grade 1:**<br>The infusion rate of study drug/study regimen may be decreased by 50% or temporarily interrupted until resolution of the event.<br>**For Grade 2:**<br>The infusion rate of study drug/study regimen may be decreased 50% or temporarily interrupted until resolution of the event.<br>Subsequent infusions may be given at 50% of the initial infusion rate.	**For Grade 1 or 2:**<br>– Acetaminophen and/or antihistamines may be administered per institutional standard at the discretion of the investigator.<br>– Consider premedication per institutional standard prior to subsequent doses.<br>– Steroids should not be used for routine premedication of Grade ≤2 infusion reactions.
**Grade 3 or 4**	**For Grade 3 or 4:**<br>Permanently discontinue study drug/study regimen.	**For Grade 3 or 4:**<br>– Manage severe infusion-related reactions per institutional standards (e.g., IM epinephrine, followed by IV diphenhydramine and ranitidine, and IV glucocorticoid).

CTCAE Common Terminology Criteria for Adverse Events; IM intramuscular; IV intravenous; NCI National Cancer Institute.

Non–Immune-Mediated Reactions

Severity Grade of the Event (NCI CTCAE version 4.03)	Dose Modifications	Toxicity Management
**Any Grade**	Note: Dose modifications are not required for AEs not deemed to be related to study treatment (i.e., events due to underlying disease) or for laboratory abnormalities not deemed to be clinically significant.	Treat accordingly, as per institutional standard.
**Grade 1**	No dose modifications.	Treat accordingly, as per institutional standard.
**Grade 2**	Hold study drug/study regimen until resolution to ≤Grade 1 or baseline.	Treat accordingly, as per institutional standard.
**Grade 3**	Hold study drug/study regimen until resolution to ≤Grade 1 or baseline.<br>For AEs that downgrade to ≤Grade 2 within 7 days or resolve to ≤Grade 1 or baseline within 14 days, resume study drug/study regimen administration. Otherwise, discontinue study drug/study regimen.	Treat accordingly, as per institutional standard.
**Grade 4**	Discontinue study drug/study regimen (Note: For Grade 4 labs, decision to discontinue should be based on accompanying clinical signs/symptoms, the Investigator's clinical judgment, and consultation with the Sponsor.).	Treat accordingly, as per institutional standard.

Note: As applicable, for early phase studies, the following sentence may be added: "Any event greater than or equal to Grade 2, please discuss with Study Physician."
AE Adverse event; CTCAE Common Terminology Criteria for Adverse Events; NCI National Cancer Institute.

Appendix 2. Durvalumab Dose Calculations

Durvalumab Dosing

The durvalumab dosing should be done depending on patient weight (if patient is ≤ 30kg):

1. Cohort dose: X mg/kg
2. Patient weight: Y kg
3. Dose for patient: XY mg = X (mg/kg) × Y (kg)
4. Dose to be added into infusion bag:

    Dose (mL) = XY mg / 50 (mg/mL)
    Where 50 mg/mL is durvalumab nominal concentration
    The corresponding volume of durvalumab should be rounded to the nearest tenth mL (0.1 mL). Dose adjustments for each cycle only needed for greater than 10% change in weight.

5. The theoretical number of vials required for dose preparation is the next greatest whole number of vials from the following formula:

    Number of vials = Dose (mL) / 10 (mL/vial)

Example:

1. Cohort dose: 20 mg/kg
2. Patient weight: 30 kg
3. Dose for patient: 600 mg = 20 (mg/kg) × 30 (kg)
4. Dose to be added into infusion bag:

    Dose (mL) = 600 mg / 50 (mg/mL) = 12.0 mL

5. The theoretical number of vials required for dose preparation:

    Number of vials = 12.0 (mL) / 10.0 (mL/vial) = 2 vials
